# Aridity drives plant biogeographical sub regions in the Caatinga, the largest tropical dry forest and woodland block in South America

**DOI:** 10.1371/journal.pone.0196130

**Published:** 2018-04-27

**Authors:** Augusto C. Silva, Alexandre F. Souza

**Affiliations:** 1 Programa de Pós-Graduação em Ecologia, CB, Universidade Federal do Rio Grande do Norte, Campus Universitário, Lagoa Nova, Natal, Rio Grande do Norte, Brazil; 2 Departamento de Ecologia, CB, Universidade Federal do Rio Grande do Norte, Campus Universitário, Lagoa Nova, Natal, Rio Grande do Norte, Brazil; Chinese Academy of Forestry, CHINA

## Abstract

Our aims were to quantify and map the plant sub regions of the the Caatinga, that covers 844,453 km^2^ and is the largest block of seasonally dry forest in South America. We performed spatial analyses of the largest dataset of woody plant distributions in this region assembled to date (of 2,666 shrub and tree species; 260 localities), compared these distributions with the current phytogeographic regionalizations, and investigated the potential environmental drivers of the floristic patterns in these sub regions. Phytogeographical regions were identified using quantitative analyses of species turnover calculated as Simpson dissimilarity index. We applied an interpolation method to map NMDS axes of compositional variation over the entire extent of the Caatinga, and then classified the compositional dissimilarity according to the number of biogeographical sub regions identified a priori using k-means analysis. We used multinomial logistic regression models to investigate the influence of contemporary climatic productivity, topographic complexity, soil characteristics, climate stability since the last glacial maximum, and the human footprint in explaining the identified sub regions. We identified nine spatially cohesive biogeographical sub regions. Current productivity, as indicated by an aridity index, was the only explanatory variable retained in the best model, explaining nearly half of the floristic variability between sub regions. The highest rates of endemism within the Caatinga were in the Core and Periphery Chapada Diamantina sub regions. Our findings suggest that the topographic complexity, soil variation, and human footprint in the Caatinga act on woody plant distributions at local scales and not as determinants of broad floristic patterns. The lack of effect of climatic stability since the last glacial maximum probably results from the fact that a single measure of climatic stability does not adequately capture the highly dynamic climatic shifts the region suffered during the Pleistocene. There was limited overlap between our results and previous Caatinga classifications.

## Introduction

Delineating biogeographical regions is an important step in understanding spatial organization of biological diversity and has been involved in both generating and testing hypotheses since the 19^th^ century [1–3]. Indeed, in order to test the mechanisms driving regional diversity, we need to define the biotic regions and quantify the diversity within them [4]. For example, identifying spatially distinct assemblages bound by environmental conditions has led to novel ecological interpretations of the fossil record [5], including greater determinism or inertia of long-term community dynamics than predicted by neutral expectations [5,6]. Likewise, biogeographical regions imply predictable species associations, therefore, define sets of species with either similar ecological requirements or non-neutral interactions [7]. Bioregionalization is also a prerequisite for producing stratified random samples, as it allows relatively homogeneous biotas to be compared in a reproducible way [8]. For example, bioregionalization makes it possible to determine whether differences in species abundance are due to real change rather than background noise. Additionally, bioregionalization is often an essential first step in conservation planning and management [9,10]. For example, bioregionalization can be used to choose priority areas for conservation within delineated biogeographical regions, which could optimize the conservation of unique biotas, and assess the effects of prioritizing species richness or endemism in conservation planning [11]. Clearly recognizing plant biogeographical regions is also important due to their role as habitat templates for animal species distribution and life-history evolution [8,12–14]. Increased data availability and the development of new analytical methods have allowed the recognition of biogeographical regions at distinct spatial scales with precision that was unimaginable a few decades ago [3,10]. For instance, the WWF Ecoregions [9] have been regarded as prominent global schemes based on the principle of biogeographic representation [11], but the internal structure of most of their units have not yet been established for different taxa.

Several non-exclusive hypotheses have been proposed to explain the current distribution of species and whole biotas. From an evolutionary point of view, the geographic size and the unique evolutionary history of each region may explain current compositional patterns better than contemporary environmental factors [15,16]. Such idiosyncratic and broad-scale effects have been called region effects and have been found to be important determinants of local species richness and composition [17–19]. The Neotropics have accumulated more species than tropical Africa and Asia due to higher speciation and extinction rates since the Eocene (ca. 60 million years BP, [20]). Many extant dry forest species originated in a continual manner since the late Eocene/early Oligocene until the Pleistocene [21,22]. The Tertiary speciation was mainly driven by paleogeographic reorganizations linked to continental drift such as the Andean orogeny, the closure of the Panama Isthmus, and the flooding of the Orinoco and Amazon basins by epicontinental seas [21,23]. From the Pleistocene on, vicariance has been mediated by disturbance, due to the alternation of glacial and inter-glacial periods that produced oscillating climatic cooling and warming. These cycles seem to have caused downward altitudinal migrations and the spread of cool-adapted species during glacial phases, as well as fragmentation and isolation of populations of species in warm inter-glacial phases, resulting in adaptive radiation and allopatric/parapatric speciation [22–24].

During Pleistocene glacial periods, dry forests may have formed a continuous expanse across much of the South America’s ‘dry diagonal’ of open vegetation, including the dry vegetation of the north-eastern Caatinga, the Cerrado savannas, the Chaco ecoregions, and even the Amazon region [25,26]. Pleistocene expansion-retraction-fragmentation dynamics not only promoted a great portion of current taxon diversity [21,22], but also widespread genetic and compositional structure between current dry forest populations, communities, and ecoregions [24,27–30]. In north-eastern South America, large tracks of the Caatinga region remained as nuclei of stable dry forest since the last glacial maximum [25,26,31]. However, in contrast to the more general increased aridity recognized in most other lowland areas of Brazil during the last glacial maximum, wetter conditions characterized glacial maxima of the now semiarid north-eastern Brazil, which was a contact zone between the Amazon and Atlantic rain forests [31–33]. This happened because distant climatic anomalies displaced the Intertropical Convergence Zone rain belt southwards several times during the late Pleistocene [34]. These wet intervals were dynamic and recent. In the last ~18,000 years several distinct climatic changes occurred, including cooler and wetter phases in which species from neighbouring Cerrado savannas and Amazon/Atlantic rain forests occupied the region [32,33,35]. The current semiarid conditions only established within the last 4,500 years [33]. Such conditions still promote evolutionary radiations in xeric plant lineages [36], the range expansion of dry forest species from xeric refugia [29,30,32], and the formation of forest refugia in wetter mountain ranges [35]. Herein, we refer to the historical rationale for regional floristic patterns as the Historical Stability Hypothesis.

Despite the importance of historical events for speciation events, their relative importance for current community assembly at local and regional scales remains uncertain, and a model combining annual energy input with water supply and topographic complexity has been able to predict the distribution of global centres of plant richness [19]. Some of the main ecological determinants of the past and present species distribution include current habitat productivity, climatic stability throughout millennia, mountain ranges, and the impact of human activities. Increased habitat productivity and the temporal stability of productivity, mainly due to thermal and rainfall regimes, are both positively related to species richness [17,19]. This is because the more energy available within a region allows species to maintain larger population sizes, increasing their speciation and reducing their extinction probabilities [4,12]. In dry forest ecosystems, small variations in productivity due to changes in rainfall, temperature, or soil nutrition—a known mediator of drought sensitivity in plants—can lead to significant changes in species composition [37–40] and ecological strategies [41–44]. In the tropics, ridges and mountain ranges promote productivity because they attenuate the effects of reduced rainfall due to their reduced temperatures [19]. Mountain ranges have also been recognized as promotors of evolutionary divergence among populations due to gene flow reduction in rough topographies, the creation of different soil and climatic gradients on their surface that favour adaptive divergence, and regional effects on climate (i.e. rain shadows) [22,30,45,46]. In tropical regions, relatively small tropical mountains may act as allopatric barriers for lowland populations due to the lack of adaptations of tropical species to colder seasonal temperatures [47]. These effects seem to have been amplified during phases of climatic change, when mountain ranges acted as refuges for lineages adapted to cooler or wetter conditions [19,22,45,48]. Finally, disturbances produced by humans in dry forest ecosystems (i.e. fire, cattle grazing, and fragmentation) are known to impact distributions and community structure of dry forest species [40,49,50] and may heavily distort patterns observed in biogeographical data [51]. We refer to these contemporary rationales for regional floristic patterns as the Current Productivity, Mountain Ranges, and Human Footprint Hypotheses.

Despite growing efforts to uncover the historical and geographical assembly of different biotic regions, Europe and North America are still the most studied continents, while more diverse tropical regions are highly underrepresented, and studies with a small grain but covering a large spatial extent are extremely scarce [19,51,52]. In this paper we focus on the Caatinga, which covers 11% of the Brazilian territory (844,453 km^2^) in north-eastern South America. The Caatinga is the largest unit of seasonally dry forest biome in the Neotropics, and is distributed in fragments from Mexico to Argentina throughout the Caribbean [53]. These disjunct areas occur on fertile soils and suffer severe dry seasons in the winter or strong summer droughts for at least 5 months [37,54,55]. They vary in physiognomy and floristic composition, but share many common species and genera [55]. In South America, the Caatinga is the largest area of seasonally dry forest and has remained relatively isolated at the north-eastern part of the ‘dry diagonal’ of open vegetation [39]. The Caatinga is highly diverse, harbouring 4,657 seed plant species, of which 913 (19.7%) are endemic species [56] mainly concentrated in the Diamantina and Araripe mountain ranges [57,58]. It has the highest number of endemic genera amongst the Neotropical seasonally dry forest and woodlands [39]. The Caatinga and the south-western Bolivia, Paraguay, and northern Argentina region comprise the most extensive area of contiguous dry forest worldwide [59], but between 1990 and 2010 the Caatinga has lost 37,068 Km^2^ of tree and scrub cover [60]. Conservation parks currently make up only ca. 1% of the Caatinga [61]. Given their high biodiversity and exposure to threats including climate change, fragmentation, deforestation, and degradation through fire and grazing, these forests have been regarded as priority areas of conservation. Fish, amphibian, and lizard distribution maps or bioregions have been proposed for the region [62], but natural plant bioregions are still lacking. Furthermore, increasing the network of protected areas in the Caatinga has been urgently advised [59,63], as well as efforts to increase scientific knowledge on biodiversity distribution [63].

Since the von Martius expedition of 1824, the Caatinga has been recognized as a distinct biogeographic unit through physiognomic, floristic, or zoological criteria of at least 15 classification schemes (reviews in [54,64,65]). Broad-scale biogeographic units frequently contain substantial internal structure, and plant regions tend to display finer structure than animal ones [16]. Internal structures have been proposed for the Caatinga based on drought severity [64,66], floristic distinctiveness [54,66], and congruence of soil, geomorphology, plant, and animal species [67]. Eisenlohr & Oliveira-Filho [68] suggested that a purely physiognomic classification captured most of the floristic variation in large regions and Fernandes [66] proposed a physiognomic classification as a proxy for floristic variation in the Caatinga. However, these proposals are qualitative [64,66,69] or based on subjective expert opinion [67,69]. They depend on the experience and judgement of the proponents and rely upon the user’s intuition in interpreting observed patterns on the basis of personal experience, and are thus not reproducible nor amenable to scientific inference [8]. The only quantitative cluster analysis was carried out by Moro et al. [54]. However, their work did not aim to establish plant biogeographical regions, but searched for broad relationships between vegetation physiognomies. The floristic clusters they found were subjectively delimited, cohesive regions were not delimited, and they did not evaluate hypotheses involving environmental determinants.

Our aims were to quantify and map the plant sub regions of the Caatinga by spatially analysing the largest dataset of woody plant distributions in this region assembled to date, compare these distributions with the current phytogeographic regionalizations, and investigate the potential environmental drivers of the floristic patterns in these sub regions. Specifically, we tested the 1) Historical Stability, 2) Current Productivity, 3) Mountain Ranges, and 4) Human Footprint non-exclusive hypotheses regarding drivers of floristic sub regions.

## Materials and methods

### Study area

**Fig 1:**
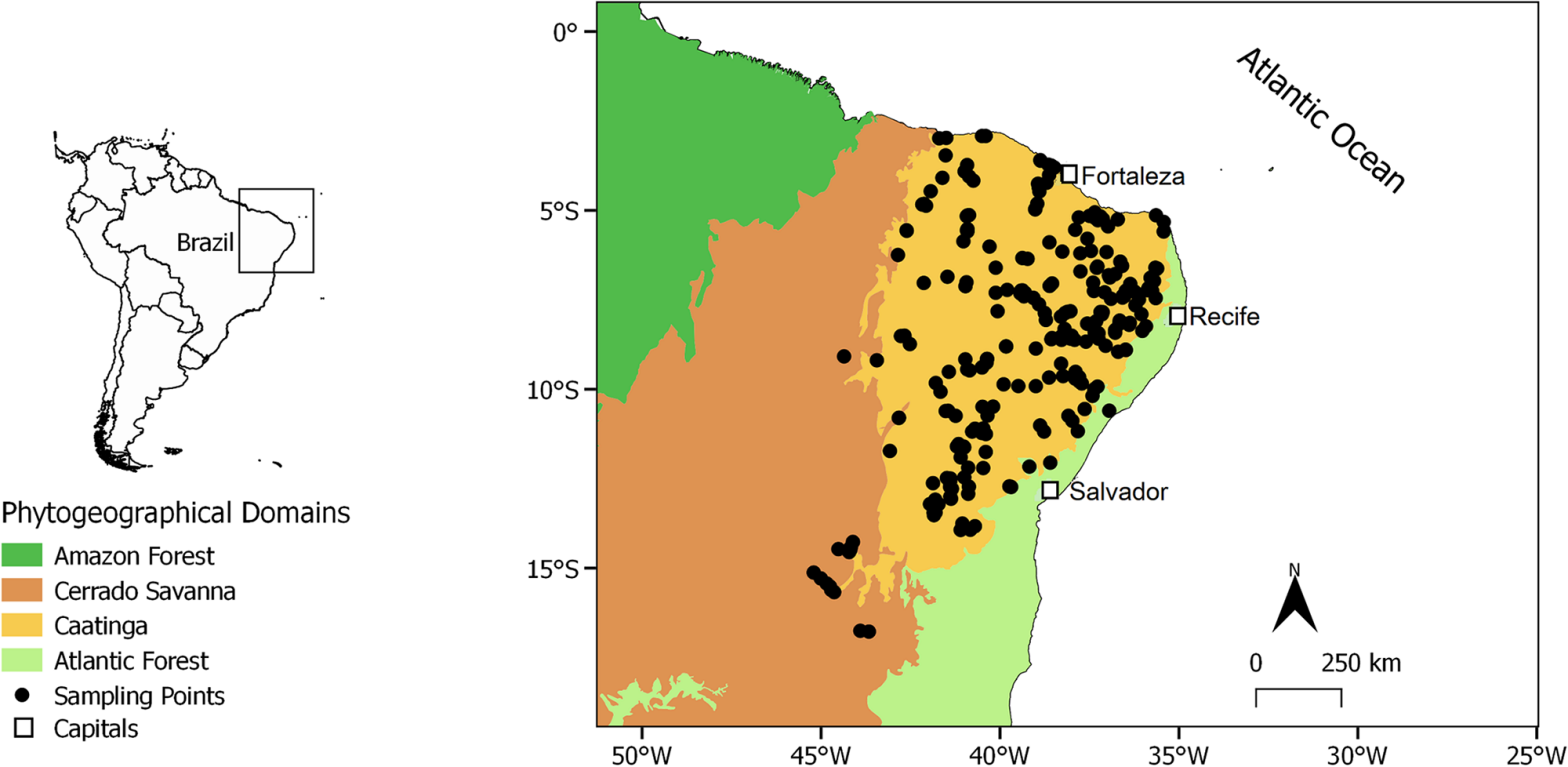
The distribution of 260 studied localities. The limits of the Caatinga dry vegetation, the Cerrado savannah, the Amazon and the Atlantic Forest dominions are shown. The inset map shows the location of the location of the studied region in South America. Note that a few studied localities were located just outside the Caatinga limits. They were included in the analyzes due to small-scale uncertainties regarding the exact location of the Caatinga limits ([67], for instance, adopt somewhat distinct southern and north-western limits), and because they shared the typical thorn dry forest physiognomy and seasonally dry climate that predominate in most of the Caatinga dominion. Their exclusion did not alter the results significantly (data not shown).

The Caatinga is located in north-eastern South America (Olson et al. [9], Fig 1) and lies on top of Pre-Cambrian granitic and gneissic basement exposed by erosion, that during the Tertiary formed nutrient-rich but stony and shallow soils referred to as crystalline terrains (reviews in [54] and [39]). Topography is relatively uniform with altitudes varying around 400 m. The main mountain ranges are the Borborema, Ibiapaba, Araripe, with altitudes that reach over 800 m and Chapada Diamantina (capped by horizontal strata of sandstone), with altitudes that reach over 1200 m (Supporting information Fig A in S1 Text). Temperatures are constant and high (ca. 28°C) in the northern and eastern parts of the domain and lower (ca. 19°C) and more seasonal to the south. The Caatinga limits mostly match the 1000 mm rainfall isohyet [70]. Annual rainfall varies greatly between years [71], and is higher in the northwest transition of Cerrado savannah and Amazon rainforest and on the windward slopes of the main mountain chains, but is less than 400 mm in the driest regions. The most arid areas form a north-eastern-southwestern belt through the region. According to Köppen, most of the domain is ’s semi-arid (Bs) or tropical with dry summer (As) climate type. Peripheral areas display tropical climates with dry winter (Aw) and a mosaic of humid subtropical with dry winter (Cw) with no dry season (Cf), and Aw climates in the Chapada Diamantina highlands [72]. Although the Caatinga has been labelled the largest area of seasonally tropical dry forest in South America [73], most plant species are shrubs and herbs [39,54,56]. The physiognomy is highly variable, including shrublands, open savannas, bushy grasslands, as well as both broadleaved and stiff-leaved deciduous and semideciduous forests and dwarf forests ([53,74], Fig B in S1 Text), and is better labelled as a seasonally dry tropical forest and woodland [39].

### Floristic and environmental data

We obtained woody floristic data from 260 locations using both literature and herbarium specimens (Table A in S1 Text). Data from the literature included 174 floristic surveys and ecological inventories published in journal articles, books, technical reports, and theses. Herbarium data was obtained using the SpeciesLink tool [75] by entering the names of all north-eastern Brazilian states and recording localities with coordinates inside the Caatinga limits that had at least 30 specimens. We excluded specimens lacking species identifications, as well as cultivated, herbaceous, epiphytic, parasitic, liana, and fern species. We did not consider any infra-specific taxa in the data set. Localities with less than five species were not included in the dataset. Coordinates of the 86 records were refined using Google Earth^™^ [76] to improve the geographical reference and increase the precision and accuracy of the maps. The Taxonomic Name Resolution Service v1.1 (http://tnrs.iplantcollaborative.org) was used to revise family, genus, and species names. Accepted species names and synonyms followed the most recently updated taxonomic resources found in the Flora do Brasil project (http://floradobrasil.jbrj.gov.br/). Synonymous species were merged with the accepted species and invalid species were discarded, totaling 571 name corrections, fusions, or exclusions. We used a high resolution to keep spatial data structure. Each site in the final data set represented woody plant assemblages at the resolution of 2.5 arc-min (ca. 5 km^2^).

Environmental data included variables representing current and historical climates, soil, and human impact. Data regarding current climate were obtained from the World-Clim project v. 1.4 at a 30″ (~ 1 km^2^) spatial resolution (Hijmans et al. [77], http://www.worldclim.org/). We defined aridity as the long-term water deficit produced by the imbalance between rainfall and temperature-driven evapotranspiration, which is more relevant to our understanding of plant ecology than either rainfall or temperature alone [78]. Due to this we calculated the Köppen aridity index (AI = MAP/(MAT + 33), where MAP is mean annual precipitation and MAT is mean annual temperature), and used it instead of temperature and rainfall variables as a more accurate and precise measurement of water availability and proxy to local productivity [79]. We measured historical variation in climate using three variables (Fig C in S1 Text). The first of these variables was the historical difference in the Köppen aridity index (HAI, measured as the difference in the aridity index between current climate and climate during the last glacial maximum). This variable indicated the historical change in aridity, i.e., conjoint change in water availability and energy input. The current and historical aridity indices showed significant but weak negative correlation (*r* = -0.37, *P* < 0.05). Two other variables accounted for historical variation in overall hydric and thermal conditions. The first was historical variation in hydric conditions. It captured the variation in the overall ‘hydric envelope’ in a region, and included annual precipitation, precipitation seasonality, and precipitation across wettest/driest/warmest/coldest seasons. The second was the historical variation in thermal conditions, which captured the overall variation in the ‘thermal envelope’ and included changes in annual mean temperature, isothermality, temperature seasonality, and temperature across warmest/coldest/wettest/driest seasons. We measured these two variables as the multivariate distance from each grid cell to the origin of the multidimensional ordination space, representing the covariation between all hydric or thermal variables. For this, we first calculated the difference between current and last glacial maximum conditions for each one of the 19 bioclimatic variables available at the WorldClim database, generating 19 ‘Δ-bioclimatic’ variables. These 19 ‘Δ-bioclimatic’ variables were separated into one group containing 11 ‘Δ-bioclimatic’ variables related to temperature (Bio1 to Bio11), and a second group including eight ‘Δ-bioclimatic’ variables related to precipitation (Bio12 to Bio19). For each group of ‘Δ-bioclimatic’ variables, we performed a principal component analysis (PCA) and extracted the PCA scores for each grid cell in each PCA axis. The Euclidean distance from each grid cell to the multidimensional ordination space origin was used as a measure of the ‘Δ-bioclimatic’ value in that cell and, therefore, was the overall hydric or thermal change in the last 21,000 years. To account for uncertainties in the estimation of historic climate conditions, we calculated four measurements of historical variation in climate (historical difference in rainfall and temperature, and the overall difference in hydric and thermal conditions) using three general circulation models for the last glacial maximum (CCSM4, MIROC-ESM, and MPI-ESM-P). We obtained final consensus values for each variable by averaging the historical variables obtained for each circulation model (Fig C in S1 Text).

Soil variables relevant to plant growth were downloaded from www.soilgrids.org [80] in 250 m^2^ resolution and were upscaled to match bioclimatic variables. We used average values of soil variables for the 0, 5, and 15 cm depths (Fig A in S1 Text). We used the revised Human Footprint map [81] to measure human impact across the Caatinga domain. The map conveys a standardized human footprint index in which data from the extent of built environments, crop land, pasture land, human population density, night-time lights, railways, roads, and navigable waterways were weighed according to estimates of their relative levels (Fig A in S1 Text). Variables were tested for multicollinearity by examining Pearson correlations based on 260 localities. Only one variable from any pair of highly cross-correlated variables (*r* > 0.75) was included in further analyses based on the potential biological relevance to floristic patterns and ease of interpretation. In total, 11 variables were retained for analyses, including elevation, three current climate variables, four historic climate variables, two soil variables, and the human footprint (Table B in S1 Text).

### Data analysis

#### Biogeographical regionalization

Recent biogeographical regionalization approaches are based on regular and continuous grids containing species-by-site data, which are subjected to hierarchical cluster analyses and generate spatially cohesive biogeographical regions [2]. Alternatively, clusters may be identified among discontinuous sample sites, while no cohesive biogeographical regions are established (e.g., [82]). Since we aimed to identify and map cohesive biogeographical regions, we followed the analytical framework proposed by Rueda et al. [13] and Moura et al. [3] to produce a spatially contiguous estimation of floristic dissimilarity and then performed regionalization based on this contiguous surface, as summarized below.

#### Interpolation procedure

This step was based on unconstrained community-level modelling of the compositional dissimilarity [83,84]. We applied an interpolation technique to model the compositional dissimilarity of unsurveyed sites and obtained a spatially contiguous representation of floristic compositional dissimilarity across the Caatinga domain. All calculations were performed in R 3.3.2 (R Core Team 2016). We first used the recluster.dist function of the ‘recluster’ package [85] to produce a dissimilarity matrix with the Simpson index. This index is recommended for biogeographical regionalization purposes [2,82] due to its independence of richness variation [86]. We then represented this dissimilarity matrix into a few dimensions, which produces colour-ramp maps that represent compositional variation [2,84]. We did this by using the metaMDS function in the ‘vegan’ package [87] to perform a three-dimension non-metric multidimensional scaling (NMDS) on the Simpson dissimilarity matrix, which reduced dimensionality while retaining as much information as possible about floristic relationships among sites. We used the NMDS scores of each site to interpolate floristic dissimilarities over the entire extent of the Caatinga. This was achieved using the inverse distance weighting technique, after examining the NMDS axes for spatial autocorrelation with Moran correlograms (see S1 Text for further details, Figs D and E in S1 Text).

We used colour combinations to visualize floristic similarities between interpolated cells. Each cell’s position on the NMDS axes was translated into an RGB colour by assigning cell positions for each of the three ordination axes to intensities of red, green, and blue [88]. We applied the same translation of axis position to colour intensity to all axes simultaneously, so that the variation shown by each of the colours was proportional to the variation explained by its respective axis. We combined the red, green, and blue components of each cell to create RGB colours that we then mapped. This method of mapping community composition allows a greater portion of community variation to be depicted, as compared to displaying each ordination axis at a time.

#### Regionalization procedure

We used K-means partitioning to produce a single partition of the interpolated axes of compositional variation (the interpolated NMDS scores) that optimized within-group homogeneity. Cells were clustered based on their species composition with no regard to spatial dependence to avoid unjustified cohesion of clusters by the actual species distributions [13]. However, in K-means the number of clusters (*k*) must be defined before the partitioning. Because K-means is an Euclidean-based analysis, and neither the NMDS axes scores nor the Simpson distance matrix are Euclidean metric (*sensu* [89]), we used a principal coordinate analysis (PCoA) to identify the best number of floristic sub-regions in which the Caatinga floristic variation should be divided. We ran a PCoA with Cailliez correction for negative eigenvalues to project the Simpson distance matrix into a multivariate Euclidean space that reproduces the exact original observed distances [7]. The PCoA was obtained with the cmdscale function of the ‘stats’ R base package. The PCoA scores were then used as input data in the Euclidean-based cluster analysis used to identify the number of clusters. We identified the optimum number of clusters using the L-method proposed by Salvador & Chan [90]. This method consists of performing a piecewise regression of the evaluation metric (here, the within-group sum of squares) against the number of clusters and finding the ‘knee’ in which two regression lines minimize the root of the mean squared error (RMSE) in the scatterplot. However, the maximum number of groups entered *a priori* in the piecewise regression as the range of the x-axis influences the identification of the optimal breakpoints, and provides different optimal number of groups in the piecewise regression [90]. We solved this problem following [3] and finding the optimal k for each possible value of maximum number of groups from 4 to n_sites_ -1. This procedure comprised 262 K-means partitioning analyses, each with 100 iterations and 50 random starting points. Because we performed piecewise regressions with distinct degrees of freedom (different maximum *k*), we used the residual standard error (RSE) instead of RMSE to identify the optimal breakpoint. We obtained 262 values of optimal *k* (including repeated values) and used the most frequent among them as the optimal number of clusters. Calculations were performed using the ‘vegan’ package and the kmeans function of the ‘stats’ R base package.

### Environmental correlates and comparison between classification systems

Following Banda-R et al. [53], we investigated relationships among the floristic groups identified by the overall clustering analyses (nine floristic groups; see below). We pooled the species lists for each group into a single list and conducted hierarchical clustering analyses on a species × floristic group matrix using the Simpson dissimilarity and UPGMA linkage method as recommended by Kreft & Jetz [2]. We used the recluster.boot function of the ‘recluster’ package (option tr = 100) to determine node significance with 1000 bootstrapped trees. In order to test our hypotheses and determine the environmental correlates of Caatinga biogeographical sub-regions, which constitutes a categorical variable with multiple levels, we used multinomial logistic regression run with the multinom function of the ‘nnet’ package [91]. Following Zuur et al. [92], we built a full model including the variable sets of soil (sand content + cation exchange capacity), topography (elevation and elevation CV), current climate (aridity index), historical climate (historical aridity index and historical variation in overall hydric and thermal conditions), and the human footprint variables. We then used model selection to identify the smallest set of variables explaining the deviance in the Caatinga floristic regions. We built models using single-predictors, each predictor set, and each combination of current and historical predictor sets. Model selection was based on the smallest value of Akaike’s information criterion corrected for small sample sizes (AICc). We used Akaike weights (wAICc) to evaluate model selection uncertainty. wAICc vary from 0 (no support) to 1 (complete support) and may be interpreted as the probability that any given model is the best model expected for the sampling situation considered [93]. The deviance of the model with the lowest AICc was partitioned to obtain the unique and shared contribution of different predictor sets to explain floristic regions.

We examined the spatial structure in the multionomial logistic regression residuals of the best supported model through spatial correlograms of Moran’s I coefficients using 14 geographical distance classes. Since we detected significant autocorrelation in the residuals of preliminary model runs, we used Moran’s Eigenvector Maps (MEMs) [94] to control for spatial autocorrelation in the model. Positive and significant (P < 0.05) MEM eigenfunctions were included as explanatory variables alongside the environmental variables in the model. MEM eigenfunctions were calculated following Borcard et al. [95] and using the ‘spacemakeR’ package [96].

To match the resolution of the species assemblage data used in the multinomial logistic regression, we calculated the mean, range, or coefficient of variation of the predictor variables listed in Table B in S1 Text using a buffer of 10-km radius centered on geographical coordinates of each site using the ‘raster’ package [97]. The Pearson correlations among the final set of environmental variables ranged from 0.007 to 0.672 and variance inflation factor (VIF) was < 3.25 for all variables, indicating low multicollinearity [98].

In order to evaluate the effectiveness of different classification systems in explaining floristic variation in the Caatinga, we classified each of our 260 localities to the fifth level of the physiognomic classification system for vegetation types in extra-Andean tropical and subtropical South America proposed by Oliveira Filho [99]. We also attributed each locality to one of the Caatinga ecoregions proposed by Velloso et al. [67] and to one of the floristic groups proposed by Moro et al. [54]. We compared the explanatory power of the different classifications using the adonis function of the ‘vegan’ package to run three two-way PERMANOVAs [100] using the Simpson distance matrix and 1000 randomizations. In each PERMANOVA, the physiognomic classification and one of the other three classification systems, including ours, were included as explanatory factors.

## Results

**Fig 2:**
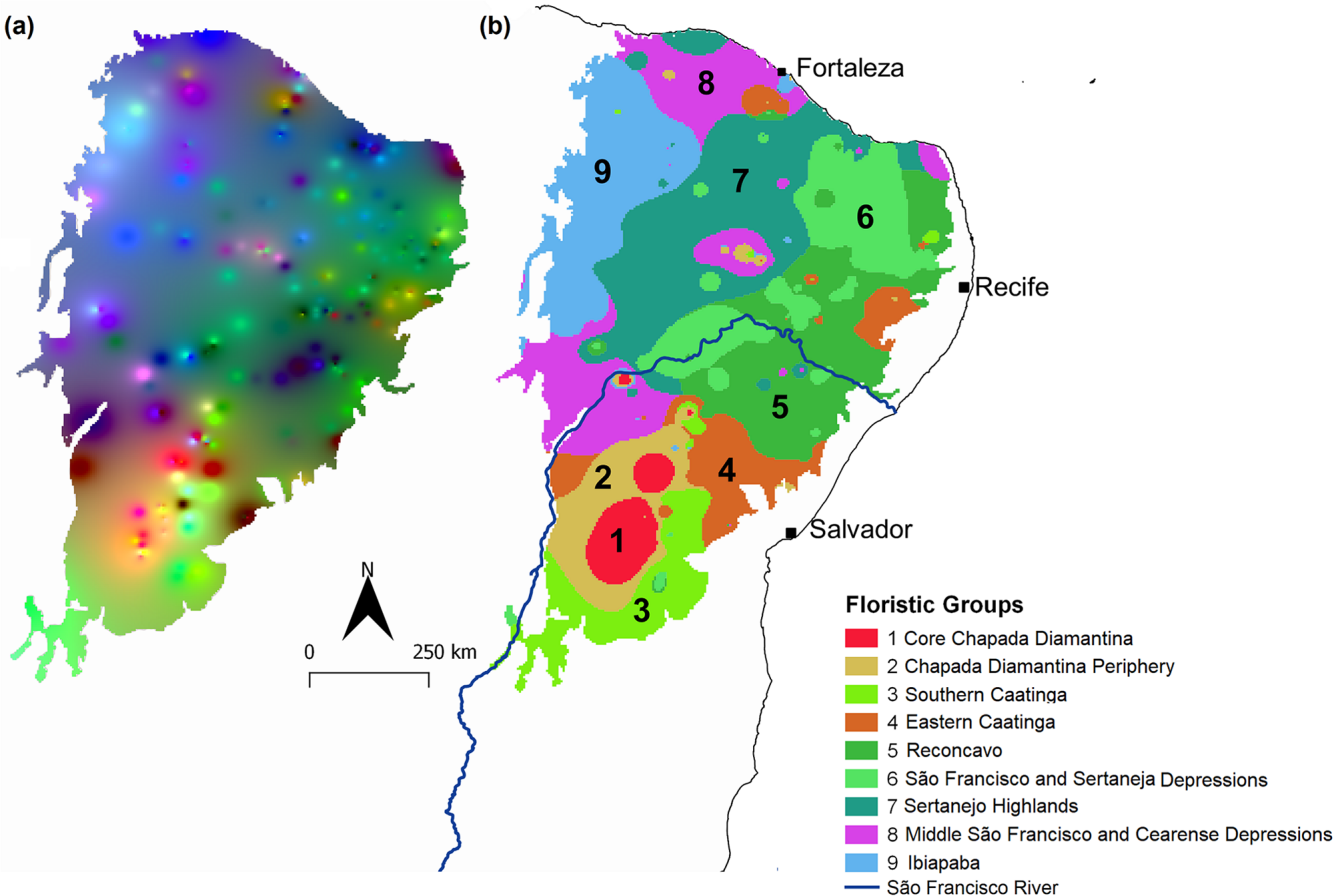
Tree and shrub floristic variation in the Caatinga. (a) Quantitative representation of beta diversity as interpolated dissimilarity based on NMDS axes. The colours of the map have no absolute meaning—only the colour differences between locations within the same study site are meaningful. (b) Regionalization of woody plants into nine biogeographical subregions based on the K-means partitioning of the interpolated values of NMDS axes. Maps drawn in 2.5 arc-min resolution.

We found a total of 2,666 shrub and tree species belonging to 778 genera and 143 botanical families in the 260 localities (Table A in S2 Text). Species restricted to a single locality totalled 1411 (52.9% of the total). The average number of species per locality was 47.0 (SD = 40.9, median = 33; range: 5–216). The three-dimensional NMDS produced high congruence between the observed and ordinated distances (non-metric fit R^2^ = 0.97, linear fit R^2^ = 0.83) and a stress value of 17.3 (Fig F in S1 Text). The floristic variation captured by the NMDS axes was depicted in a beta diversity map (Fig 2a), where cells of similar colour contained similar woody communities (lower Simpson dissimilarity), providing a visual interpretation of the turnover between any two cells and the total floristic variation. The K-means piecewise regression L-method identified nine floristic groups as the best solution for our dataset (Fig G in S1 Text). The subsequent K-means clustering of the interpolated NMDS identified the following floristic biogeographical regions: (1) Core Chapada Diamantina, (2) Chapada Diamantina Periphery, (3) sSouthern Caatinga, (4) Eastern Caatinga, (5) Reconcavo, (6) São Francisco and Sertaneja Depressions, (7) Sertanejo Highlands, (8) Middle São Francisco and Cearense Depressions, and (9) Ibiapaba (Fig 2b and Table C in S1 Text). The Core Chapada Diamantina was the smallest region, but contained the highest number of species and nearly five times the number of exclusive species than the other regions. Although we did not let spatial dependency influence the clustering of neighbouring cells, the groups showed strong spatial cohesiveness, although disjunctions of relatively small sizes were common. The shapefile of the groups described above is available in S1 File–Shapefile floristic groups of the Caatinga.

**Fig 3:**
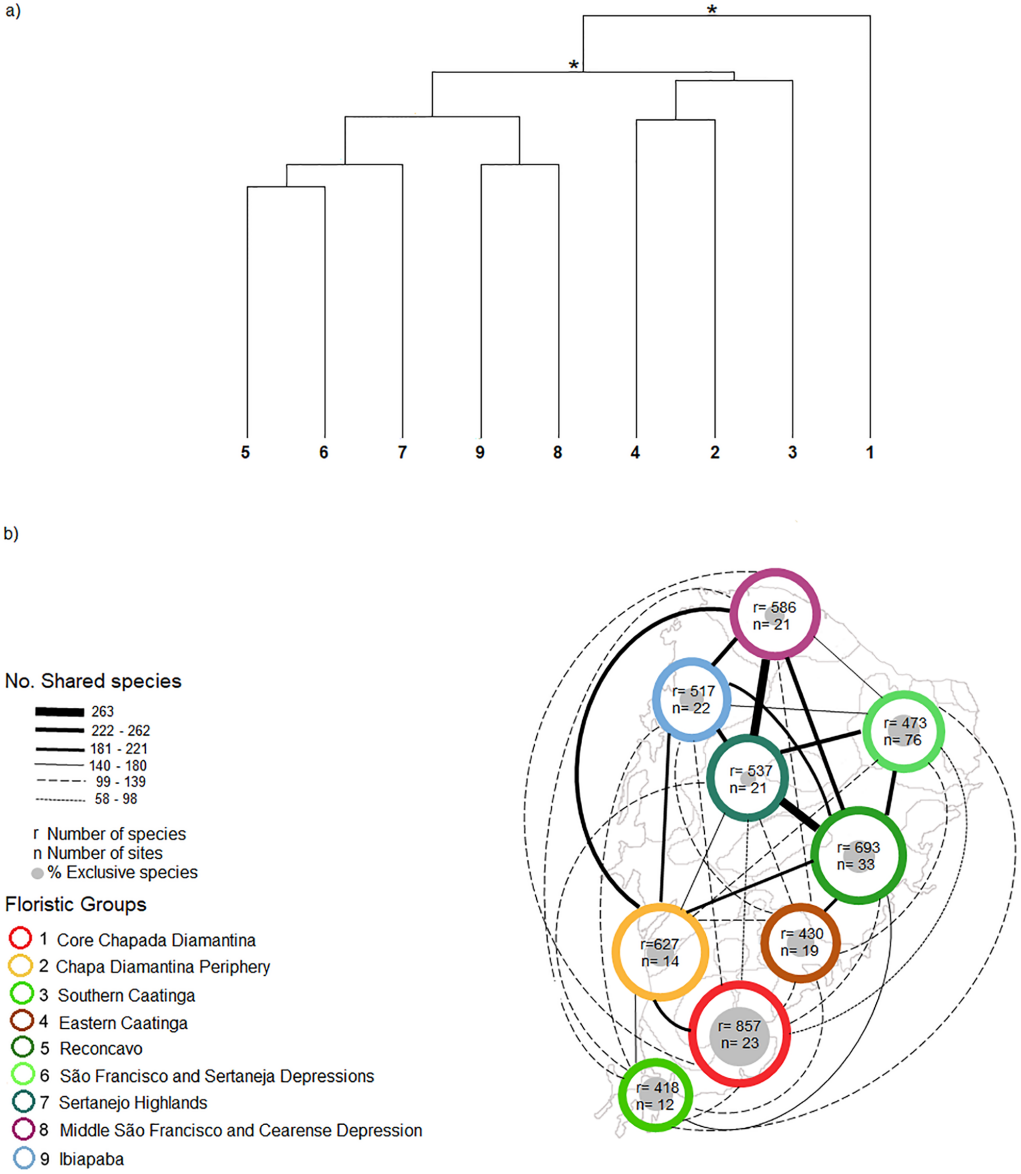
Floristic groups of the Caatinga. (a) Hierarchical classification of the 9 Caatinga floristic groups using Simpson dissimilarity and UPGMA as the linkage method. This analysis highlights the relationships between the 9 floristic groups identified by the K-means analysis and identified higher-level clusters of groups. Asterisks correspond to the statistically significant (*P* < 0.05) nodes obtained using 1000 iterations of bootstrap resampling. Horizontal bars indicate the corresponding higher-level clusters of groups. (b) Geographical patterns of species turnover among the nine Caatinga biogeographical sub-regions. The size of colored circles is proportional to the total number of species and of gray circles to the number of exclusive species per sub-region. The species turnover among areas is described by line widths proportional to the shared number of species (values from Table D in S1 Text).

The hierarchal UPGMA pooling all sites in each of the nine floristic groups, complemented by a nonmetric multidimensional scaling (NMDS) ordination, recognized three higher-level clusters of groups (Fig 3a and H-I in S1 Text). The Core Chapada Diamantina group was floristically distinct from all other groups. It formed a separate branch in the dendrogram and its localities clustered at the lower end of the third axis of the NMDS. The distinctiveness of this sub-region was due to the reduced number of species this group shared with the other groups (Fig 3b and Table D in S1 Text). The remaining groups formed two higher-order clusters, which split the Caatinga woody flora into two broad clusters (Fig 3a and J in S1 Text). The southern cluster comprised the Chapada Diamantina Periphery, the Southern Caatinga, and the Eastern Caatinga groups. These groups shared relatively few species (Fig 3b and Table D in S1 Text). The northern cluster comprised the remaining groups and presented the widest aridity intensities, including the São Francisco and Sertaneja Depressions and the Ibiapaba groups. The sub-regions in this northern higher-order group formed a floristic gradient along the first axis of the NMDS (Fig H in S1 Text). Despite this, these groups showed the strongest connections, sharing the highest number of species among all pairwise comparisons (Fig 3b and Table D in S1 Text).

The nine biogeographical sub-regions we identified explained a larger fraction of the floristic variability in the Caatinga (27.7%) than the floristic classification employed by Moro et al. [54] (18.4%) and the soil-relief-plant-animal sub-regions based on expert opinions of Velloso et al. [67] (18.7%)(PERMANOVA, Table E in S1 Text). Variation in vegetation physiognomy also explained a significant portion of floristic variation, and the interaction between floristic sub-region and physiognomy was significant in all three PERMANOVAs. This means that there were species occurring preferentially in specific combinations of our biogeographical sub-regions and vegetation types, like the semi-arid deciduous stiff-leaved scrub in the Eastern Caatinga floristic sub-region. The inclusion of floristic sub-regions doubled or tripled (our sub-regions) the portion of floristic variance explained relative to only physiognomy in all cases.

Spatial structure in floristic variation was captured by eight positive MEMs, which effectively controlled for spatial autocorrelation in the model residuals and were included in all tested models (Fig J in S1 Text). Floristic variation among the nine biogeographical groups was explained mostly by variation in current aridity. The model with only the aridity index was the best supported model, including current and historic environmental conditions, as well as the human footprint (Table F in S1 Text). Explained deviance was highest for the full model (59%), but most of the variables contributed weakly and the model with only the aridity index was the best supported (lowest AICc and highest wAICc), explaining 47.63% of the variability in the biogeographical sub-regions. The unique contribution of aridity accounted for the largest fraction of this variability in the sub-regions (78.06% of the explained variability), while the spatial structures captured in the MEMs accounted for 21.94% of the explained variability.

## Discussion

### The determinants of the Caatinga floristic sub regions act through direct and indirect effects

We confirmed the Current Productivity hypothesis since the woody flora of the Caatinga was spatially organized by variation in aridity. As an integrative measure, aridity is a proxy to local productivity because it reflects the balance between heat load and hydrological dynamics [38,79]. In north-eastern South America, aridity poses severe constraints on plant growth and biomass accumulation, not only due to seasonal drought coupled with consistently high year-round temperatures, but also because of high year-to-year variation in annual rainfall, which can cause several dry years in a row [71]. Arid environments are known to act as biogeographical filters that select species with particular ecological strategies. Increased semiarid conditions select for stress-tolerant strategies that favour resource maintenance over resource acquisition [41,43]. Morpho-physiological traits that increase survivorship in drought-prone tropical environments include slow growth, xylem that is resistant to drought-induced cavitation, high sapwood capacitance that protects xylem from critically low water potentials, deciduousness, photosynthetic stems that have the potential to assimilate carbon with greater water-use efficiency than leaves, deep roots, regulation of gas exchange to reduce leaf water loss or to maintain photosynthesis at low leaf water potential, and low cuticular conductance of exposed tissues during extended drought [42]. Many of these traits have been found in the Caatinga flora [54,64,66], but our regionalization makes certain sub regions more likely to present such traits than others, mainly the São Francisco and Sertaneja Depressions, Reconcavo, and the Middle São Francisco and Cearense Depression.

The effects of the human footprint, elevation, and historic climate stability did not hold when aridity was included in the analyses. However, these exclusions may have distinct meanings. The impact of human activities as fire, cattle grazing, and vegetation destruction and fragmentation are known to change species distributions and community structure in the Caatinga [49,101]. However, our analyses suggest that these impacts have a rather homogeneous distribution across the region. Their effects on species distributions, as strong as they may be, seem to be local and did not influence the broad-scale floristic patterns we detected, which agrees with findings from other regions [5,13]. This also agrees with recent findings that floristic gradients along the South American dry diagonal respond to climatic changes more so than to human impacts like fragmentation and isolation [40]. The exclusion of elevation variation indicates that the main mountain ranges of the Caatinga (Chapada Diamantina, Araripe, Borborema, Ibiapaba, Fig A in S1 Text) act indirectly on floristic patterns through increased productivity because they attenuate aridity through reduced temperatures [19]. In arid and semiarid ecosystems, small variations in productivity can lead to significant changes in species composition [37,38]. This occurs in part because productivity increases shift the relative advantage of distinct sets of traits and ecological strategies from more arid-stress tolerance to competitive resource acquisition [41,44]. Furthermore, aridity and vegetation biomass/physiognomy are spatially structured by orographic rain and rain shadows produced in mountain ranges and inselbergs by the strong easterly trade winds that bring moisture from the Atlantic ocean, as well as forest refugia and semideciduous forests that cover eastern slopes and mountain tops and deciduous and thorn vegetation that cover the drier western slopes [46]. The main mountain ranges of the Caatinga have been recognized as historic forest refugia for plant lineages adapted to wetter conditions [35].

The environmental complexity that mountain ranges create is known to reduce plant species migration and colonization in the tropics due to the narrow temperature ranges that tropical species tolerate [47], as well as to the niche diversity that the heterogenous mountain environment creates [45]. Therefore, such indirect elevation effects were strong enough to produce two distinctive plant sub regions (Core and Periphery of the Chapada Diamantina) and northern disjunct areas in the Borborema, Araripe, and Ibiapaba mountain ranges. The two Chapada Diamantina sub regions had a higher concentration of restricted species, agreeing with the estimation made by Manhães et al. [57]. The Chapada Diamantina represents one end of the larger Espinhaço mountain range that runs southwards into Minas Gerais state, where it is covered by the Atlantic forest and Cerrado savannah. Therefore, it is possible that a portion of the distinctive diversity of the Chapada Diamantina we registered represents the northern ranges of southern rainforest and savannah species. Many regional differences in species diversity that have been classically attributed to historical factors can also be predicted by contemporary differences in the environment [19]. However, the Chapada Diamantina has been recognized as a centre of endemism, with hundreds of unique genera and species [58,67]. This has been attributed to evolutionary divergence promoted by gene flow reduction caused by barriers as valleys, rifts, and steep walls [22,30,45] and is reinforced by the separation of the Core Chapada Diamantina biogeographical sub region from all other sub regions in our UPGMA analysis. Even though large rivers may act as geographical barriers for Cactaceae in Eastern Brazil [36], the Caatinga biogeographical sub regions we detected were not delimited by major rivers like the São Francisco or the Jaguaribe, suggesting that such rivers did not represent barriers to seed movement for most of the plant species.

Our estimates of the Caatinga paleoclimate and historic aridity and climatic stability portrayed a much more arid climate 21,000 years ago than today’s climate. The lack of an effect of historic climate stability on current Caatinga floristic sub regions may indicate that current floristic patterns do not carry any significant historic signatures. Yet, this is unlikely since vegetation is known to be spatially organized by historic factors to a large extent [17–19]. The highly biodiverse species pool in South American has evolved more or less continuously since the Tertiary [20], driven mainly by geographic and tectonic forces and then by climatic changes during the Pleistocene [21,22]. Such climatic changes were quite frequent and extensive, with alternating dry and wet periods with different intensities and durations throughout the last couple million years until very recently [26,31–33]. These changes have produced genetic signals of range expansion for dry forest and woodland species from xeric refugia [29,30,32], as well as for recent divergence in xeric plant lineages [36]. Our snapshot of the past climatic conditions probably did not capture the long and complex cumulative effects of environmental change that may explain much of the deviance unexplained by current aridity in our model, mainly in the eastern parts of the Caatinga [26,31].

### Biogeographical relationships of the Caatinga sub regions

Most plant lineages found in the Caatinga drylands came from Mesoamerican seasonally dry forest and woodland communities, with subsequent in situ diversification in the Caatinga in pre-Pleistocenic times [39]. This is attributed to the fact that plant lineages of this biome are strongly shaped by niche conservatism and dispersal limitations, and because Caatinga’s harsh climatic conditions pose severe limits to the establishment of immigrant species which are not pre-adapted to long and erratic dry seasons [27,39]. At the same time, the Caatinga is bordered by a diverse array of biogeographical provinces, including the Atlantic and Amazon rain forests and the Cerrado Savannah. The proximity of these diverse vegetation types must have contributed to the recruitment of Caatinga lineages and elevated diversity [53]. Our UPGMA analysis clustered biogeographical sub regions as Chapada Diamantina Periphery, Southern Caatinga, and Eastern Caatinga. All these sub regions are closer to the eastern and northeastern portions of the Atlantic forest complex, and were repeatedly covered by Atlantic forest expansions during the Pleistocene [26,31]. Thus, aside from the Chapada Diamantinga being a major diversification centre [39], these sub regions most likely also include floristic transitions to distinct Atlantic forest floristic sub regions, most likely the Pernambuco and Bahia rainforests (Eastern Caatinga) and the Interior semideciduous forests (Chapada Diamantina Periphery and Southern Caatinga) [102]. The floristic influence of the Cerrado is probably determined by the distinction of the Middle São Francisco and Cearense Depression sub region, where the southwestern portion borders the Cerrado domain and the northeastern disjunction coincides with a Cerrado physiognomic disjunction in Rio Grande do Norte state (Fig B in S1 Text).

The cluster of the Middle São Francisco and Cearense Depression sub regions with the other northern sub regions in the UPGMA suggests a pervasive presence of Cerrado species in central and northern Caatinga areas. This is compatible with the fossil record, which registers repeated savannah expansions into northeastern Brazil in the past 200,000 years [32]. The northwestern Ibiapaba sub region was distinguished by two transitions. The first was a climatic transition to a wetter climate, since this was the least drought-prone region. This means increased productivity and change in the suite of advantageous ecological strategies [44]. Additionally, this sub region borders the ecotone of the Amazon rainforest and the northernmost part of the Cerrado savannah in the Piaui state, known as Campo Maior complex [54,67]. Indeed, it is likely that both the Ibiapaba and its eastern neighbor Sertanejo Highlands sub region include transitional zones between the xeric flora of the São Francisco and Sertaneja Depressions and Reconcavosub regions and the northern Cerrado and eastern Amazon floras, mediated by decreasing aridity and topographic complexity. This interpretation is supported by the elevated beta-diversity estimated by Manhães et al. [57] for the northern Caatinga, especially its northwestern portion. It is worth noting that the few bioregions tentatively proposed for animal groups share with the plant bioregions we found the pivotal role of the Chapada Diamantina as a center of diversity and endemism [62]. Apart from this animal bioregions in the Caatinga seem to be much broader and cohesive than plant bioregions. This pattern repeats findings made in other continents and probably results from the restricted migration hability of plants, coupled with their increased frequency of sympatric speciation [16].

### Comparison with earlier subdivision attempts and practical implications

The plant biogeographical sub regions we identified contrasted with previous regionalization attempts for the Caatinga in three important ways. First, they highlighted the importance of studying plant species separately from geomorphological and animal distributions. Previous bioregionalizations proposed for the Caatinga were based on geomorphological units as well as on plant and animal distributions (e.g., [67]). Such synthetic units may hide important differences in the bioregionalization of broad areas, as recently seen for several contrasting plant, bird, mammal, and reptile bioregionalizations [5,13,16]. Second, although expert consensus may be valuable to support conservation and management decisions when there is a lack of detailed distribution data, quantitative analyses of distributional data are much more accurate. Distributional data can distinguish precise bioregion borders and delineate subdivisions like the nested spatial structure in the Chapada Diamantina, with a core and more distinctive sub region surrounded by a significantly different peripheral sub region with distinct floristic affinities. Third, they emphasized the value of a data-driven approach instead of constraining the analyses to previously postulated biogeographical units (e.g., [54,69]). For highly biodiverse biotas, most assumed regionalizations will yield significant floristic differences, but these are not necessarily the strongest or more natural ones.

We attribute these causes to the fact that the sub regions we identified did not confirm previous proposals, although there were a few exceptions. We confirmed the Chapada Diamantina as a distinctive floristic unit in the Caatinga, as in Velloso et al. [67] and Moro et al. [54], although distinguished its core area separate from its periphery. Other mountain ranges like the Borborema, Ibiapaba, and Araripe did not form particular sub regions as suggested by Velloso et al. [67]. The Eastern Caatinga sub region corresponded with the agreste floristic unit proposed by Rizzini [64], Fernandes [66], and Moro et al. [54], which constitutes a transition to the Atlantic forest, although it is not as continuous or extensive as previously thought. We found that the northern and southern Sertaneja depressions of Velloso et al. [67] were much more complex and subdivided floristically, while their Raso da Catarina semiarid unit was mostly included in our Reconcavo and São Francisco and Sertaneja Depressions sub regions. The assumed distinction between floras of sedimentary versus crystalline terrains [39,54,67,69] was not confirmed. Floristic sub regions did not match the distribution of sandy or crystalline soils nor were soil variables included in the best model selected to explain the relationship between biogeographical units and past climate, current environmental, or human footprint variables. This is not to say that soil factors do not influence community structure and species distributions in the Caatinga. There is evidence that they do [54] but our results indicate that they are likely to be more determinant for floristic structure on smaller spatial scales than at the Caatinga sub region scale. Our analyses were based on the Simpson dissimilarity index and thus emphasize species turnover and not community nestedness [86]. Some of the floristic patterns previously ascribed to human activities, geomorphological, soil, and physiognomic variation, may correspond to patterns of community nestedness within the distinct biogeographical sub regions, which is a future step for understanding the Caatinga biome.

Our results also highlight the distinction between floristic and physiognomic variation. The official Brazilian vegetation classification is the product of a great effort to map the highly complex physiognomic variation [74], and improvements in this classification have occurred [99]. However, the Caatinga floristic sub regions present different physiognomic units (i.e., seasonally dry forests, open scrub, and short thorn forests) that are overlapped (Fig 2 and B in S1 Text), as for European plant bioregions [13]. Commonly recognized vegetation types like *Restinga* along coastal sandy plains, rainforests on mountainous refugia, and thorn scrubs in the semiarid Raso da Catarina did not comprise separate floristic units. Importantly, the deciduous forest patches found in western Bahia and parts of the Chapada Diamantina did not form separate biogeographical units. Therefore, they can hardly be regarded as valid biogeographical disjunctions of the Atlantic forest, as defined by the Brazilian government decree that established the Atlantic forest limits [103]. This has two important implications. First, that phenotypic plasticity is strong enough in the region so that species can express distinct habits and trait values across different vegetation types, to a large extent driven by local resource variation [43,104]. Second, although physiognomic variation captures a significant portion of changes in species composition [68], we should not use vegetation types as proxies for floristic composition because they masks the main biogeographical patterns in plant species distributions. Rather, vegetation types should be used as a classification scheme complimentary to recognized biogeographical sub regions.

In summary, we identified the main plant biogeographical sub regions of the biodiversity-rich Caatinga nucleus in the Neotropical dry forest and woodland biome, based on a rigorous quantitative analysis of a large data set. Our results help refine the current terrestrial ecoregions [9], and create a more accurate taxonomy for Brazil’s phytogeographical regions and sub-regions, which are still entirely based on physiognomic variation [74]. The biogeographical regionalization we proposed, which is available as shape files in the Supplementary Material, can be used for strategic conservation and management planning, for measuring and modelling change, and to test hypotheses about the ecology and evolution of dry forest biotas [4,5,6,8,10], including animal species [12,14]. Our regionalization also allows regional applications to be aggregated into regional-wide assessments, facilitating the growing demand for coherent ecological data to assist policy and assess of the state of the global environment. Furthermore, a greater understanding of the changes in morpho-physiological traits and ecological strategies that accompany sub region shifts will enable a broader understanding of the adaptive history of species and their potential for adaptation in the face of human induced climate change [38].

## Supporting information

S1 File Shapefile of the floristic groups of the Caatinga. (ZIP)

S1 Text Fig A. Distribution of current environmental variables at the Caatinga biogeographical province at 2.5 arc-min (ca. 5 km2) resolution. Fig B. Caatinga physiognomies according to the categories proposed by Oliveira-Filho [99]. Table A. General characterization of the studied localities in the Caatinga vegetation domain. Fig C. Estimated variation in the geographic distribution of abiotic variables in the Caatinga province between the last glacial maximum (ca. 22000 years before present) and the present. Table B. Selected environmental variables and their descriptive statistics. Fig D. Moran’s spatial correlograms for the NMDS ordination axes using the Simpson dissimilarity matrix. Fig E. Interpolated scores of the non-metric muldimensional scaling (NMDS) ordination based on Simpson β-diversity distances. Fig F. Shepard diagram for the non-metric muldimensional scaling (NMDS). Fig G. K-means test. Table C. Description of Caatinga forest floristic groups: Fig H. NMDS ordination plots in three dimensions of Caatinga biogeographical sub-regions with 260 localities. Fig I. NMDS ordination plots in two dimensions of the nine Caatinga biogeographical sub-regions. Table D. Shared species among Caatinga floristic groups. Table E. Comparison of classification schemes. Fig J. Moran’s correlograms. Table F. Multinomial logistic regression models used to investigate the influence of current and historical environmental conditions as well as the human footprint in explaining the biogeographical sub-regions for woody plants in the Caatinga.
(DOCX)

S2 Text Table A. Woody plant species recorded after compilation of 260 inventories in the Caatinga.
(DOCX)
