# Supplementary material for: Aridity drives plant biogeographical sub regions in the Caatinga, the largest tropical dry forest and woodland block in South America
Source: PLoS One. 2018 Apr 27;13(4):e0196130. doi: 10.1371/journal.pone.0196130 (PMC5922524; doi:10.1371/journal.pone.0196130)
Supplement: S1 Text — Fig A. Distribution of current environmental variables at the Caatinga biogeographical province at 2.5 arc-min (ca. 5 km2) resolution. Fig B. Caatinga physiognomies according to the categories proposed by Oliveira-Filho [99]. Table A. General characterization of the studied localities in the Caatinga vegetation domain. Fig C. Estimated variation in the geographic distribution of abiotic variables in the Caatinga province between the last glacial maximum (ca. 22000 years before present) and the present. Table B. Selected environmental variables and their descriptive statistics. Fig D. Moran’s spatial correlograms for the NMDS ordination axes using the Simpson dissimilarity matrix. Fig E. Interpolated scores of the non-metric muldimensional scaling (NMDS) ordination based on Simpson β-diversity distances. Fig F. Shepard diagram for the non-metric muldimensional scaling (NMDS). Fig G. K-means test. Table C. Description of Caatinga forest floristic groups: Fig H. NMDS ordination plots in three dimensions of Caatinga biogeographical sub-regions with 260 localities. Fig I. NMDS ordination plots in two dimensions of the nine Caatinga biogeographical sub-regions. Table D. Shared species among Caatinga floristic groups. Table E. Comparison of classification schemes. Fig J. Moran’s correlograms. Table F. Multinomial logistic regression models used to investigate the influence of current and historical environmental conditions as well as the human footprint in explaining the biogeographical sub-regions for woody plants in the Caatinga. (DOCX) [file pone.0196130.s002.docx]

**Aridity drives plant biogeographical sub regions in the Caatinga, the largest tropical dry forest and woodland block in South America**

Augusto C. Silva, Alexandre F. Souza

**Supporting information 1**


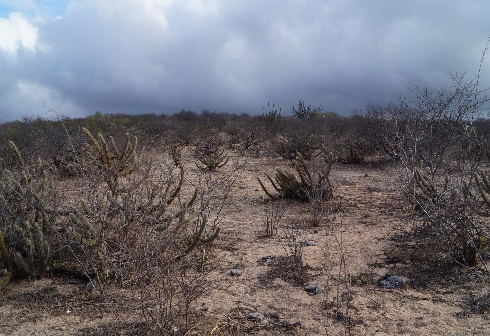

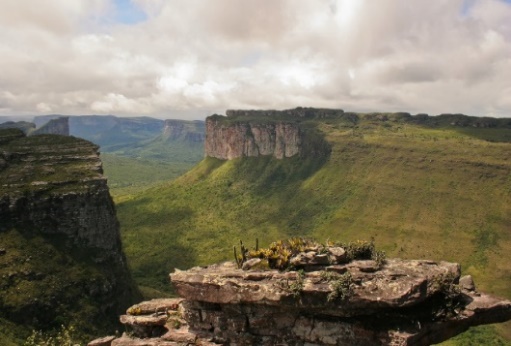

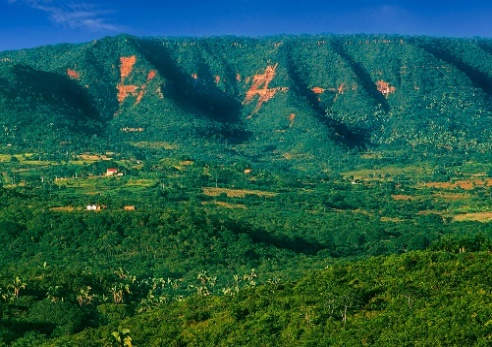

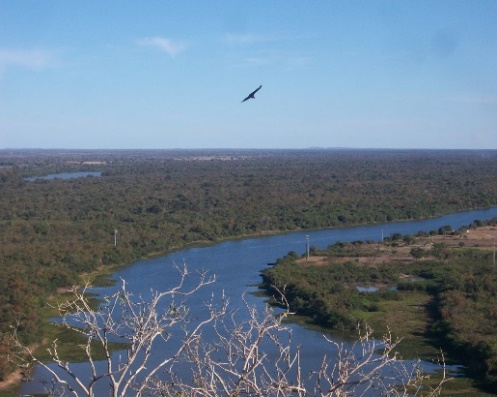

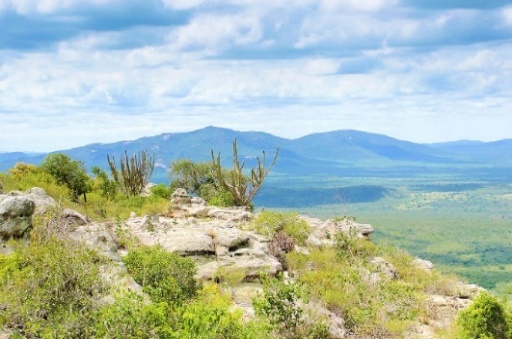

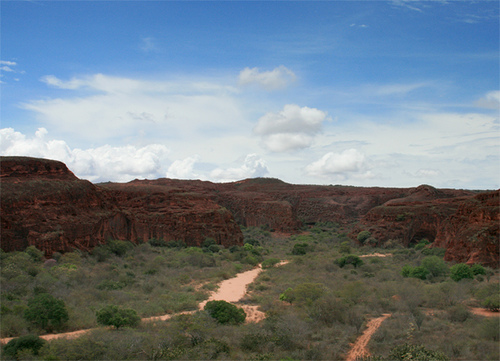

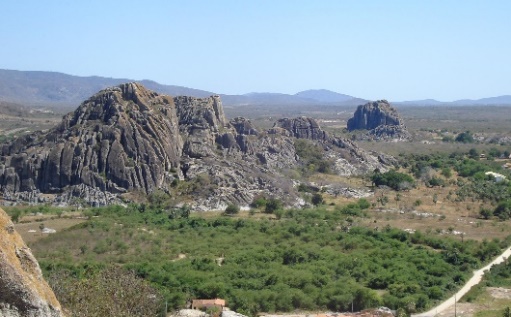

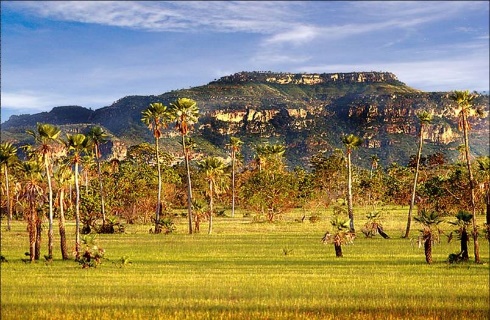

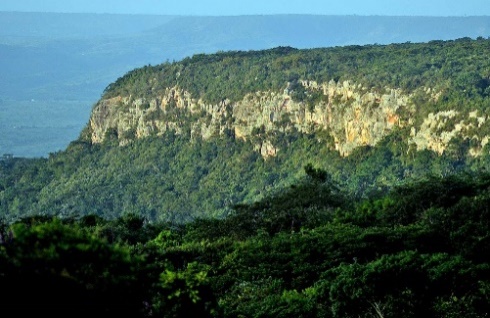


A

H

E

B

D

G

I

F

C

Examples of Caatinga vegetation physiognomies found in each of the 9 biogeographical sub regions identified in the present contribution, northeastern South America. A) Core Chapada Diamantina, B) Chapada Dimantina Periphery, C) Riverine Depressions, D) Eastern Caatinga, E) Moderate Semiarid, F) Intense Semiarid, G) Sertanejo Highlands, H) Middle São Francisco and Cearense Depression, I) Pluvial. Photo credits: Augusto C. Silva for picture F, all others https://commons.wikimedia.org.

Content

[Data analysis: dataset exploration 5](#_Toc511305582)

[**S1 Fig 1. Distribution of current environmental variables at the Caatinga biogeographical province at 2.5 arc-min (ca. 5 km2) resolution.** (a) Elevation (m) with the main mountain ranges indicated (1 – Ibiapaba highlands, 2 – Borborema highlands, 3 – Northern Sertaneja lowlands, 4 – Southern Sertaneja lowlands, 5 – Araripe highlands, 6 – Diamantina highlands). (b) Mean annual temperature (°C). (c) Mean diurnal temperature range (°C). (d) Annual rainfall (mm per year). (e) Köppen Aridity Index (note that the lowest the value the more arid the local climate). (f) Soil sand content (%). (g) Soil cation exchange capacity (cmol+/kg). (h) Human footprint. 7](#_Toc511305583)

[**S1 Fig 2. Caatinga physiognomies. Physiognomic units correspond to IBGE units re-classified according to the categories proposed by Oliveira-Filho** [10]**.** 8](#_Toc511305584)

[**S1 Table 1. General characterization of the studied localities in the Caatinga vegetation domain.** 8](#_Toc511305585)

[**S1 Fig 3. Estimated variation in the geographic distribution of abiotic variables in the Caatinga province between the last glacial maximum (ca. 22000 years before present) and the present**. (a) Historic variation in overall hydric conditions, including annual precipitation, precipitation seasonality, and precipitation across wettest/driest/warmest/coldest seasons. (b) Historical variation in overall thermal conditions, which included changes in annual mean temperature, isothermality, temperature seasonality, and temperature across warmest/coldest/wettest/driest seasons. (c) Historical variation in mean temperature. (d) Historical variation in annual rainfall. (e) Historical variation in aridity index. The variables shown in maps (c) and (d) were not used directly in the data analyses but were included here for informative purposes. 17](#_Toc511305586)

[**S1 Table 2. Selected environmental variables and their descriptive statistics.** CV = coefficient of variation. 18](#_Toc511305587)

[**S1 Fig 4. Moran’s spatial correlograms for the NMDS ordination axes using the Simpson dissimilarity matrix.** 19](#_Toc511305588)

[**S1 Fig 5. Interpolated scores of the non-metric muldimensional scaling (NMDS) ordination based on Simpson β-diversity distances**. (a) First, (b) second, and (c) third axes of the interpolated NMDS-scores. Maps drawn in 2.5 arc-min resolution. 20](#_Toc511305589)

[**S1 Fig 6. Shepard diagram for the non-metric muldimensional scaling (NMDS).** The NMDS using the Simpson dissimilarity, depicting the 3-D space of the NMDS plotted against the Simpson distance. 21](#_Toc511305590)

[**S1 Fig 7. K-means test.** (a) Variation in the optimal number of clusters (k) identified by the L-method algorithm according to increasing number of maximum k, which is the number of points in the piecewise regression. (b) Histogram for the values of optimal k selected when varying the maximum k from 4 to (n_sites_ – 1). The red bar indicates the optimal k = 9. 22](#_Toc511305591)

[**S1 Table 3. Description of Caatinga forest floristic groups**: area, number of recorded and exclusive species, as well as topographic and climatic characteristics. 23](#_Toc511305592)

[**S1 Fig 8. NMDS ordination plots in three dimensions of Caatinga biogeographical sub-regions with 260 localities**; stress values = 0.124, tr = 10000. B: 12 floristic groups; stress values = 0.116, tr = 100; relationships inferred from a classification using the UPGMA method (Fig. S2) are indicated by lines**.** 24](#_Toc511305593)

[**S1 Fig 9. NMDS ordination plots in two dimensions of the nine Caatinga biogeographical sub-regions;** stress values = 0.10, tr = 10000. The relationships depicted by the lines were inferred from a classification using the UPGMA method (Fig. 3a). 25](#_Toc511305594)

[**S1 Table 4. Shared species among Caatinga floristic groups**. Deeper grey shade indicates greater numbers of shared species, corresponding to line widths in Figure 3. 26](#_Toc511305595)

[**S1 Table 5. Comparison of classification schemes.** Explanatory potential of different Caatinga sub-region schemes and vegetation physiognomy on woody plant composition. 28](#_Toc511305596)

[**S1 Fig 10. Moran’s correlograms.** Representation the residuals of the multinomial logistic regression (MLR) using a nine-level categorical variable as dependent variable to represent the Caatinga woody plant biogeographical sub-regions. 29](#_Toc511305597)

[**S1 Table 6. Multinomial logistic regression models used to investigate the influence of current and historical environmental conditions as well as the human footprint in explaining the biogeographical sub-regions for woody plants in the Caatinga.** Spatial autocorrelation was controlled for through the inclusion of eight Moran’s Eigenvector Maps (MEMs) in all tested models (MEM1 + MEM2 + MEM3 + MEM4 + MEM5 + MEM6 + MEM8 + MEM11). Models are presented in increasing AICc order. IAC = Köppen Aridity Index, ElevCV = coefficient of variation of elevation, ElevR = elevation range, CEC = cation exchange capacity, Sand = soil sand content, HHP = Historic variation in overall hydric conditions, HHC = Historical variation in overall thermal conditions, HFP = human footprint. 30](#_Toc511305598)

[References 33](#_Toc511305599)

# Data analysis: dataset exploration

Interpolation methods depend on the degree of spatial autocorrelation of the dependent variable (the NMDS axes in this case). Moran’s I correlograms revealed spatial structure in the NMDS axes (S1.4 Fig), which were appropriate for interpolation. The interpolated NMDS axes (S1.5 Fig) should be seen as a first approximation of the floristic compositional dissimilarity in the Caatinga, based on the assumption that the rate of geographical turnover in species composition is similar among observed and interpolated sites [1]. We interpolated each NMDS axis using the inverse distance weighting technique, which employs a search window of variable size and a weighting parameter, which is the power of the inverse distance weighting function (1/d^p^_ij_; where d = distance between points i and j, p = power exponent)[2]. The greater the value of p, the less influence distant points have on the estimated value. We used p = 2 as in Moura et al. [3] whose simulations indicated that this value produced inverse distance weighting estimations highly correlated with observed values (average R^2^ = 0.84 for the three NMDS axes). Computations were performed using the idw function of the ‘gstat’ package [4].

The biogeographical regionalization procedure described in the main text identified clearly defined floristic groupings in space based on floristic affinities captured in the NMDS axes. These floristic affinities were detected without recourse to environmental variables. The relationship between floristic groups and environmental variables was then tested through multinomial logistic regression. Two alternatives were previously tried but performed poorly. The first alternative was a hierarchical cluster analysis run using the Simpson dissimilarity as a distance measure and the unweighted pair-group method with arithmetic mean (UPGMA) as the linkage method as suggested by Kreft and Jetz [1]. The analysis was run altering the order of the sites 1000 times with the recluster.node.strength function of the 'recluster' package, and summarized using a 50% majority rule consensus tree with the recluster.cons function of the same package, in order to control for ties in the data, as suggested by Dapporto *et al*. [2]. Floristic groups were defined by comparing all the possible subdivisions of the tree, using the measure of ‘explained dissimilarity’ with the recluster.expl.diss function of the 'recluster' package and using the minimal division explaining at least 90% of dissimilarity [3]. This approach, however, was very sensitive to outliers and rather than identifying spatially clearly defined clusters, recovered an unresolved tree with 38 floristic groups, many of which contained very few or even single locations making the results difficult to interpret. Furthermore, there is no established method to interpolate the groups identified with this approach to unsampled cells of the dominion in order to produce a useful map. This result agrees with Linder et al. [4], who reported poorly defined groups for sub-Saharan plant using an UPGMA-based approach.

The second alternative was a multivariate regression tree [5] relating the species presence-absence matrix to the matrix of environmental variable after removal of highly correlated (*r* ≥ 0.75) variables. Species restricted to a single locality were excluded before analysis. Results indicated that the Köppen aridity index was the only variable capable of splitting the species dataset into two groups. This model, however, left behind a large amount of floristic variation due to the exclusion of singletons, which corresponded to a large proportion of the species, and had virtually no explanatory power (Error = 0.974; CVE = 1.01), being thus unusable to interpolate group affiliations to unsampled cells in the Caatinga dominion.

**
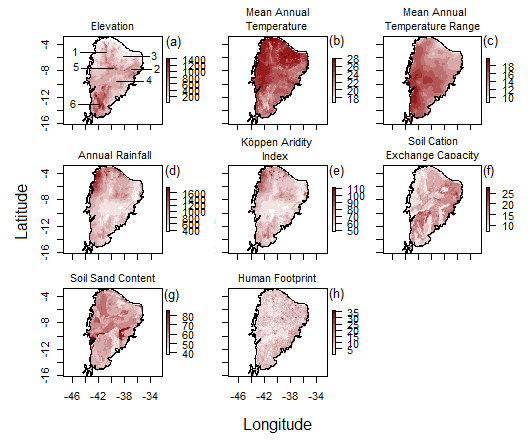
**

## S1 Fig A. Distribution of current environmental variables at the Caatinga biogeographical province at 2.5 arc-min (ca. 5 km2) resolution. (a) Elevation (m) with the main mountain ranges indicated (1 – Ibiapaba highlands, 2 – Borborema highlands, 3 – Northern Sertaneja lowlands, 4 – Southern Sertaneja lowlands, 5 – Araripe highlands, 6 – Diamantina highlands). (b) Mean annual temperature (°C). (c) Mean diurnal temperature range (°C). (d) Annual rainfall (mm per year). (e) Köppen Aridity Index (note that the lowest the value the more arid the local climate). (f) Soil sand content (%). (g) Soil cation exchange capacity (cmol+/kg). (h) Human footprint.


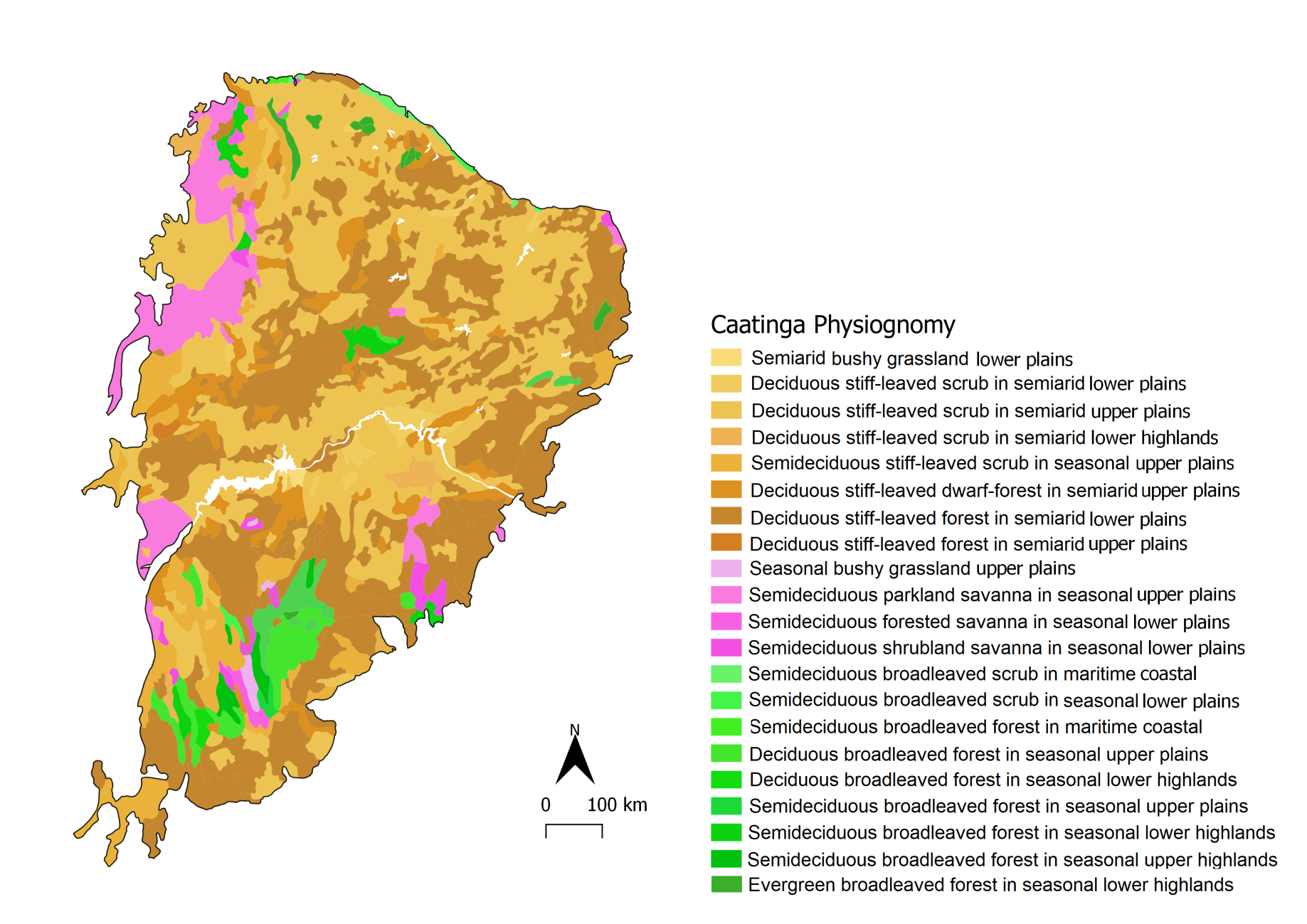


## **S1 Fig B. Caatinga physiognomies. Physiognomic units correspond to IBGE units re-classified according to the categories proposed by Oliveira-Filho** [10]**.**

## **S1 Table A. General characterization of the studied localities in the Caatinga vegetation domain.**

| Locality | Latitude | Longitude | Altitude (m) | References |
| --- | --- | --- | --- | --- |
| 1 | -8.583 | -38.033 | 800 | [11,12] |
| 2 | -7.250 | -37.378 | 1197 | [13] |
| 3 | -6.622 | -35.610 | 480 | [14] |
| 4 | -6.639 | -35.633 | 360 | [14] |
| 5 | -6.970 | -35.704 | 600 | [15,16] |
| 6 | -6.969 | -35.700 | 618 | [17] |
| 7 | -8.890 | -36.493 | 963 | [18] |
| 8 | -8.167 | -36.667 | 1098 | [19] |
| 9 | -8.341 | -36.783 | 980 | [20] |
| 10 | -8.143 | -36.373 | 642 | [21] |
| 11 | -7.833 | -38.117 | 1104 | [22] |
| 12 | -8.417 | -36.783 | 1082 | [23] |
| 13 | -8.783 | -37.050 | 798 | [19] |
| 14 | -8.600 | -38.567 | 316 | [19] |
| 15 | -4.367 | -38.917 | 171 | [24, 25] |
| 16 | -11.900 | -41.100 | 890 | [26] |
| 17 | -11.100 | -40.700 | 835 | [26] |
| 18 | -11.250 | -40.395 | 430 | [26] |
| 19 | -11.750 | -40.400 | 500 | [26] |
| 20 | -2.983 | -41.701 | 18 | [27] |
| 21 | -2.971 | -41.502 | 19 | [27] |
| 22 | -3.593 | -38.876 | 32 | [28] |
| 23 | -3.609 | -38.868 | 21 | [28] |
| 24 | -2.919 | -40.504 | 15 | [29] |
| 25 | -14.416 | -44.167 | 658 | [30] |
| 26 | -7.117 | -40.966 | 420 | [31] |
| 27 | -7.817 | -38.033 | 1100 | [32] |
| 28 | -7.867 | -38.183 | 900 | [32] |
| 29 | -8.197 | -36.392 | 972 | [33] |
| 30 | -7.967 | -38.300 | 600 | [22] |
| 31 | -9.908 | -39.489 | 250 | [34] |
| 32 | -7.837 | -37.192 | 600 | [35] |
| 33 | -9.385 | -40.508 | 369 | [12] |
| 34 | -9.256 | -40.378 | 399 | [12] |
| 35 | -8.403 | -37.358 | 600 | [36] |
| 36 | -6.810 | -36.961 | 271 | [37] |
| 37 | -8.617 | -37.917 | 860 | [38] |
| 38 | -6.703 | -37.754 | 196.5 | [39] |
| 39 | -8.238 | -35.922 | 530 | [40] |
| 40 | -6.567 | -37.278 | 200 | [41] |
| 41 | -7.270 | -36.241 | 500 | [42] |
| 42 | -7.903 | -37.152 | 621 | [43] |
| 43 | -6.881 | -35.795 | 596 | [44] |
| 44 | -8.667 | -37.600 | 600 | [45] |
| 45 | -8.583 | -37.250 | 835 | [46] |
| 46 | -8.060 | -38.719 | 495 | [47] |
| 47 | -8.312 | -38.197 | 545 | [48,49] |
| 48 | -7.288 | -37.077 | 594 | [42] |
| 49 | -7.054 | -36.362 | 519 | [42] |
| 50 | -7.385 | -36.569 | 462 | [42] |
| 51 | -7.387 | -36.535 | 449 | [42] |
| 52 | -7.434 | -36.579 | 480 | [42] |
| 53 | -7.247 | -36.487 | 490 | [42] |
| 54 | -7.066 | -36.363 | 522 | [42] |
| 55 | -7.382 | -36.535 | 462 | [42] |
| 56 | -8.533 | -38.083 | 480 | [50] |
| 57 | -8.517 | -38.283 | 460 | [50] |
| 58 | -8.164 | -37.567 | 524 | [50] |
| 59 | -8.167 | -37.550 | 520 | [50] |
| 60 | -9.149 | -40.364 | 374 | [51] |
| 61 | -7.400 | -39.333 | 900 | [52, 118] |
| 62 | -3.909 | -40.990 | 830 | [53, 118] |
| 63 | -5.141 | -40.914 | 650 | [54] |
| 64 | -12.550 | -41.417 | 800 | [55] |
| 65 | -5.519 | -40.910 | 750 | [56] |
| 66 | -5.553 | -40.926 | 780 | [56] |
| 67 | -5.586 | -40.911 | 760 | [56] |
| 68 | -6.329 | -39.377 | 230 | [57, 58] |
| 69 | -8.800 | -39.833 | 453 | [59] |
| 70 | -6.874 | -36.933 | 299 | [60] |
| 71 | -6.600 | -40.121 | 500 | [61, 118] |
| 72 | -6.352 | -39.231 | 260 | [57] |
| 73 | -8.735 | -42.520 | 600 | [62] |
| 74 | -7.816 | -40.069 | 422 | [63] |
| 75 | -4.867 | -42.067 | 100 | [64] |
| 76 | -8.500 | -38.000 | 440 | [65] |
| 77 | -8.617 | -38.283 | 375 | [65] |
| 78 | -8.100 | -37.317 | 556 | [65] |
| 79 | -7.867 | -38.750 | 480 | [66] |
| 80 | -8.800 | -39.827 | 373 | [63] |
| 81 | -9.389 | -40.503 | 379 | [63] |
| 82 | -7.250 | -35.750 | 340 | [67] |
| 83 | -7.450 | -35.650 | 224 | [67] |
| 84 | -7.370 | -36.133 | 479 | [67] |
| 85 | -7.650 | -36.217 | 527 | [67] |
| 86 | -10.182 | -37.417 | 230 | [68] |
| 87 | -10.550 | -37.636 | 204 | [68] |
| 88 | -4.970 | -39.018 | 207 | [69] |
| 89 | -6.009 | -40.294 | 400 | [70] |
| 90 | -9.775 | -37.792 | 235 | [71] |
| 91 | -9.717 | -37.817 | 280 | [71] |
| 92 | -9.617 | -37.933 | 280 | [71] |
| 93 | -9.700 | -37.900 | 230 | [71] |
| 94 | -8.366 | -36.024 | 865 | [72] |
| 95 | -7.301 | -39.303 | 412 | [73] |
| 96 | -8.950 | -36.700 | 921 | [74] |
| 97 | -8.574 | -38.546 | 319 | [75] |
| 98 | -8.568 | -38.548 | 327 | [75] |
| 99 | -7.017 | -37.400 | 300 | [76] |
| 100 | -9.079 | -44.358 | 300 | [77] |
| 101 | -9.66 | -37.789 | 174 | [78] |
| 102 | -7.49 | -36.12 | 395 | [79] |
| 103 | -9.15 | -40.36 | 390 | [80] |
| 104 | -7.47 | -36.9 | 607 | [81] |
| 105 | -7.4 | -36.53 | 450 | [81, 118] |
| 106 | -8.5 | -42.67 | 441 | [82] |
| 107 | -8.51 | -42.77 | 431 | [82] |
| 108 | -3.8 | -38.485 | 23 | [83] |
| 109 | -8.434 | -37.249 | 615 | [84] |
| 110 | -10.483 | -40.194 | 530 | [85] |
| 111 | -11.189 | -40.503 | 580 | [85] |
| 112 | -13.752 | -41.052 | 317 | [86] |
| 113 | -14.472 | -44.191 | 691 | [87] |
| 114 | -14.434 | -44.183 | 688 | [87] |
| 115 | -14.466 | -44.516 | 684 | [87] |
| 116 | -14.490 | -44.184 | 684 | [87] |
| 117 | -14.266 | -44.111 | 684 | [87] |
| 118 | -16.749 | -43.903 | 684 | [87] |
| 119 | -16.772 | -43.665 | 684 | [87] |
| 120 | -5.151 | -37.502 | 147 | [88] |
| 121 | -5.164 | -37.199 | 54 | [88] |
| 122 | -5.052 | -37.351 | 49 | [88, 118] |
| 123 | -5.273 | -37.267 | 41 | [88] |
| 124 | -6.853 | -41.470 | 495 | [89] |
| 125 | -9.51 | -41.43 | 480 | [26, 118] |
| 126 | -11.58 | -41.15 | 1027 | [26] |
| 127 | -5.54 | -37.9 | 133 | [90] |
| 128 | -11.22 | -40.51 | 492 | [26, 118] |
| 129 | -11.18 | -40.78 | 801 | [26] |
| 130 | -5.79 | -37.56 | 144 | [91] |
| 131 | -10.8 | -42.83 | 402 | [92] |
| 132 | -9.67 | -38.63 | 606 | [26] |
| 133 | -9.63 | -38.24 | 322 | [26] |
| 134 | -6.15 | -38.26 | 244 | [93] |
| 135 | -6.13 | -37.46 | 174 | [94] |
| 136 | -12.19 | -40.89 | 747 | [17] |
| 137 | -12.72 | -40.87 | 564 | [17] |
| 138 | -12.92 | -40.89 | 353 | [17] |
| 139 | -12.2 | -40.47 | 432 | [17] |
| 140 | -11.18 | -38.77 | 437 | [95] |
| 141 | -11.01 | -38.88 | 154 | [95] |
| 142 | -9.86 | -39.9 | 496 | [96] |
| 143 | -3.46 | -41.52 | 185 | [97] |
| 144 | -12.16 | -39.18 | 450 | [98] |
| 145 | -13 | -41.39 | 980 | [99] |
| 146 | -12.561 | -41.413 | 600 | [100] |
| 147 | -12.556 | -41.390 | 600 | [100] |
| 148 | -10.74 | -38.09 | 390 | [101] |
| 149 | -5.13 | -40.866 | 310 | [102] |
| 150 | -5.14 | -40.902 | 700 | [102] |
| 151 | -5.16 | -40.93 | 691 | [102] |
| 152 | -6.55 | -36.59 | 325 | [103] |
| 153 | -6.43 | -36.64 | 277 | [103] |
| 154 | -9.91 | -39 | 406 | [104] |
| 155 | -10.88 | -37.98 | 374 | [105] |
| 156 | -15.121 | -45.206 | 750 | [106] |
| 157 | -15.286 | -45.007 | 590 | [106] |
| 158 | -15.426 | -44.845 | 545 | [106] |
| 159 | -15.509 | -44.753 | 494 | [106] |
| 160 | -15.609 | -44.716 | 467 | [106] |
| 161 | -15.668 | -44.634 | 463 | [106] |
| 162 | -7.22 | -39.8 | 810 | [107] |
| 163 | -7.3 | -40.12 | 790 | [107] |
| 164 | -7.32 | -39.43 | 934 | [107] |
| 165 | -4.95 | -39.01 | 270 | [108] |
| 166 | -10.793 | -42.823 | 554 | [109] |
| 167 | -8.86 | -39 | 325 | [110] |
| 168 | -14.549 | -44.216 | 634 | [111] |
| 169 | -5.25 | -36.71 | 12 | [112] |
| 170 | -4.81 | -38.96 | 210 | [113] |
| 171 | -4.01 | -38.63 | 117 | [114] |
| 172 | -7.230 | -35.880 | 432 | [115] |
| 173 | -6.778 | -36.783 | 605 | [116] |
| 174 | -13.93 | -41.101 | 357 | [117] |
| 175 | -9.283 | -38.295 | 311 | [118] |
| 176 | -7.623 | -38.912 | 456 | [118] |
| 177 | -4.087 | -41.611 | 92 | [118] |
| 178 | -7.842 | -37.132 | 650 | [118] |
| 179 | -13.284 | -41.827 | 951 | [118] |
| 180 | -10.607 | -41.519 | 1016 | [118] |
| 181 | -4.817 | -42.121 | 120 | [118] |
| 182 | -4.166 | -40.747 | 123 | [118] |
| 183 | -8.078 | -36.658 | 574 | [118] |
| 184 | -2.919 | -40.401 | 19 | [118] |
| 185 | -6.244 | -42.852 | 113 | [118] |
| 186 | -10.597 | -36.949 | 3 | [118] |
| 187 | -10.066 | -41.663 | 458 | [118] |
| 188 | -7.007 | -40.938 | 277 | [118] |
| 189 | -5.135 | -35.642 | 6 | [118] |
| 190 | -9.470 | -40.851 | 391 | [118] |
| 191 | -11.169 | -37.827 | 171 | [118] |
| 192 | -4.834 | -42.126 | 120 | [118] |
| 193 | -9.440 | -40.918 | 397 | [118] |
| 194 | -10.600 | -41.450 | 836 | [118] |
| 195 | -7.240 | -39.414 | 419 | [118] |
| 196 | -5.554 | -42.612 | 210 | [118] |
| 197 | -9.823 | -41.795 | 415 | [118] |
| 198 | -11.62 | -41 | 897 | [118] |
| 199 | -12.622 | -41.874 | 962 | [118] |
| 200 | -13.207 | -41.958 | 1334 | [118] |
| 201 | -3.745 | -38.571 | 23 | [118] |
| 202 | -11.6 | -41.016 | 936 | [118] |
| 203 | -11.722 | -43.077 | 434 | [118] |
| 204 | -4.462 | -41.938 | 131 | [118] |
| 205 | -13.084 | -41.809 | 1338 | [118] |
| 206 | -12.493 | -41.386 | 496 | [118] |
| 207 | -12.780 | -41.344 | 444 | [118] |
| 208 | -13.058 | -41.369 | 1208 | [118] |
| 209 | -12.479 | -41.484 | 875 | [118] |
| 210 | -7.443 | -39.068 | 967 | [118] |
| 211 | -7.235 | -39.372 | 415 | [118] |
| 212 | -7.081 | -36.362 | 539 | [118] |
| 213 | -5.445 | -36.983 | 83 | [118] |
| 214 | -5.862 | -41.024 | 526 | [118] |
| 215 | -7.025 | -42.131 | 190 | [118] |
| 216 | -6.6 | -35.66 | 436 | [118] |
| 217 | -10.488 | -40.491 | 816 | [118] |
| 218 | -5.589 | -35.429 | 39 | [118] |
| 219 | -5.321 | -35.427 | 5 | [118] |
| 220 | -6.602 | -37.299 | 206 | [118] |
| 221 | -11.126 | -40.557 | 594 | [118] |
| 222 | -4.048 | -40.865 | 905 | [118] |
| 223 | -4.47 | -38.901 | 181 | [118] |
| 224 | -13.925 | -40.831 | 309 | [118] |
| 225 | -13.443 | -41.802 | 1012 | [118] |
| 226 | -9.606 | -37.918 | 204 | [118] |
| 227 | -13.826 | -40.702 | 264 | [118] |
| 228 | -12.050 | -38.597 | 232 | [118] |
| 229 | -4.829 | -42.170 | 124 | [118] |
| 230 | -7.170 | -35.853 | 603 | [118] |
| 231 | -5.195 | -37.8 | 145 | [118] |
| 232 | -9.510 | -37.890 | 219 | [118] |
| 233 | -5.569 | -42.607 | 157 | [118] |
| 234 | -4.225 | -38.698 | 90 | [118] |
| 235 | -9.996 | -37.368 | 179 | [118] |
| 236 | -12.46 | -40.998 | 623 | [118] |
| 237 | -7.040 | -38.546 | 343 | [118] |
| 238 | -5.890 | -38.621 | 122 | [118] |
| 239 | -13.449 | -41.856 | 1269 | [118] |
| 240 | -11.529 | -41.158 | 1076 | [118] |
| 241 | -12.719 | -39.719 | 285 | [118] |
| 242 | -12.713 | -39.739 | 261 | [118] |
| 243 | -12.729 | -39.69 | 607 | [118] |
| 244 | -3.73 | -40.919 | 395 | [118] |
| 245 | -9.188 | -43.442 | 701 | [118] |
| 246 | -3.722 | -38.647 | 19 | [118] |
| 247 | -12.726 | -41.396 | 746 | [118] |
| 248 | -4.835 | -42.170 | 125 | [118] |
| 249 | -4.252 | -38.938 | 873 | [118] |
| 250 | -13.518 | -41.847 | 1059 | [118] |
| 251 | -10.733 | -40.35 | 444 | [118] |
| 252 | -11.597 | -41.205 | 1093 | [118] |
| 253 | -9.923 | -37.276 | 64 | [118] |
| 254 | -7.110 | -38.615 | 407 | [118] |
| 255 | -6.162 | -37.025 | 139 | [118] |
| 256 | -9.836 | -37.716 | 203 | [118] |
| 257 | -5.239 | -37.128 | 58 | [118] |
| 258 | -4.263 | -38.933 | 865 | [118] |
| 259 | -6.197 | -37.757 | 334 | [118] |
| 260 | -5.197 | -37.361 | 23 | [118] |


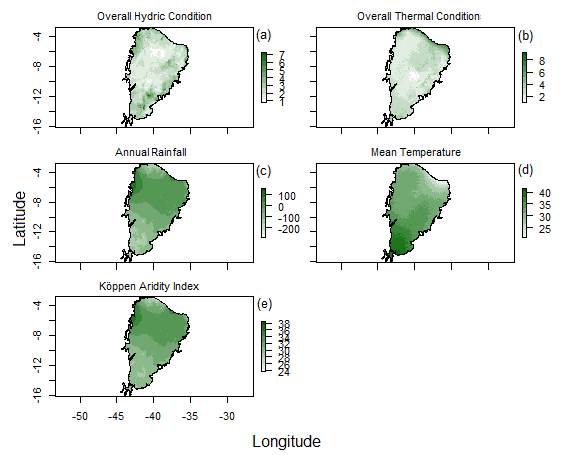


## **S1 Fig C. Estimated variation in the geographic distribution of abiotic variables in the Caatinga province between the last glacial maximum (ca. 22000 years before present) and the present**. (a) Historic variation in overall hydric conditions, including annual precipitation, precipitation seasonality, and precipitation across wettest/driest/warmest/coldest seasons. (b) Historical variation in overall thermal conditions, which included changes in annual mean temperature, isothermality, temperature seasonality, and temperature across warmest/coldest/wettest/driest seasons. (c) Historical variation in mean temperature. (d) Historical variation in annual rainfall. (e) Historical variation in aridity index. The variables shown in maps (c) and (d) were not used directly in the data analyses but were included here for informative purposes.

## **S1 Table B. Selected environmental variables and their descriptive statistics.** CV = coefficient of variation.

| Variable | Unit | Min-Max | Mean | CV (%) |
| --- | --- | --- | --- | --- |
| Elevation | m | 8.42 - 1290 | 444 | 56.89 |
| Current Aridity Index |  | 50 - 96 | 66 | 16.06 |
| Soil Cation Exchange Capacity | cmolc.Kg^-1^ | 7.63 - 22.68 | 13.75 | 23.41 |
| Soil Sand Content | % | 39.96 - 82.95 | 56.9 | 10.24 |
| Historical variation in hydric conditions^1^ | mm | 1.28 - 7.15 | 2.59 | 37.74 |
| Historical variation in thermal conditions^2^ | °C | 1.29 - 7.31 | 2.83 | 39.30 |
| Human Footprint |  | 2.19 - 33.60 | 10 | 43.57 |
| Historic Aridity Index – IAH |  | 26.4 - 36.2 | 32 | 5.48 |

1 - Difference between current and estimated last glacial maximum annual precipitation. 2 - Difference between current and estimated last glacial maximum mean temperature.


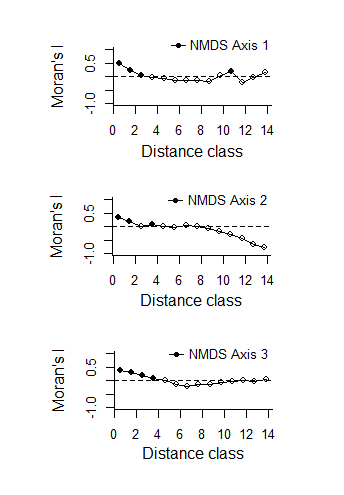


## **S1 Fig D. Moran’s spatial correlograms for the NMDS ordination axes using the Simpson dissimilarity matrix.**


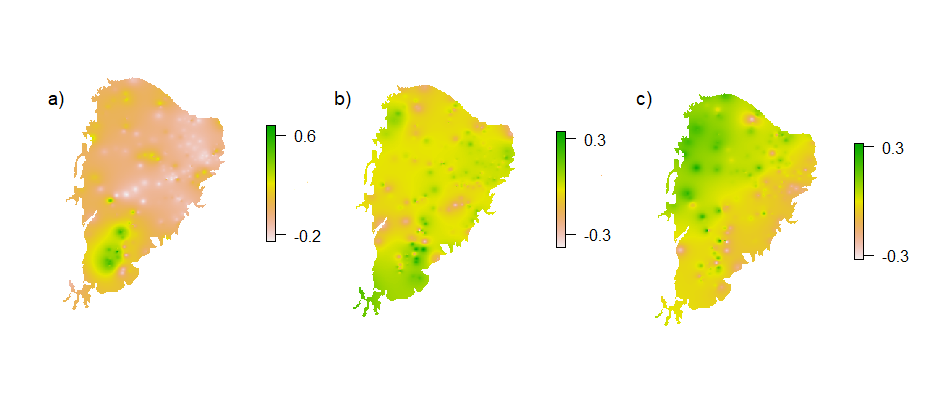

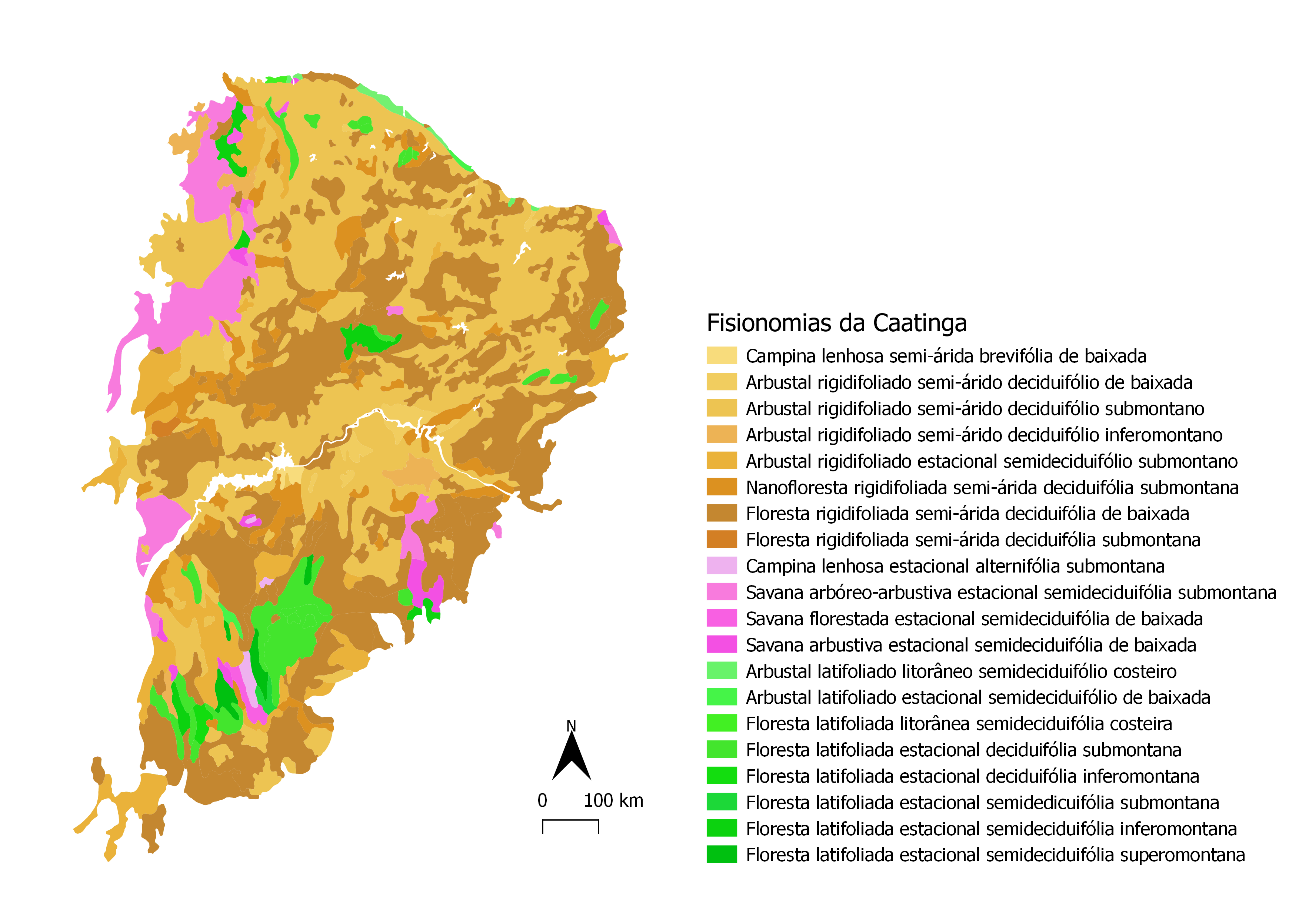


## **S1 Fig E. Interpolated scores of the non-metric muldimensional scaling (NMDS) ordination based on Simpson β-diversity distances**. (a) First, (b) second, and (c) third axes of the interpolated NMDS-scores. Maps drawn in 2.5 arc-min resolution.


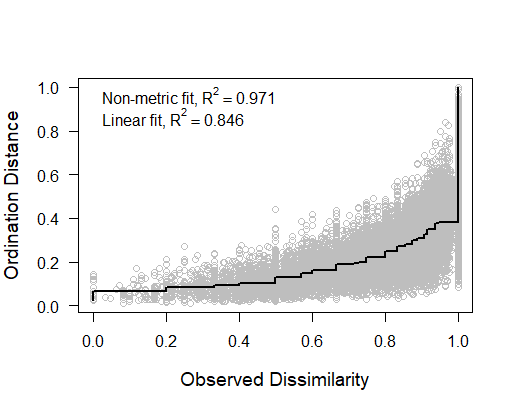


## **S1 Fig F. Shepard diagram for the non-metric muldimensional scaling (NMDS).** The NMDS using the Simpson dissimilarity, depicting the 3-D space of the NMDS plotted against the Simpson distance.


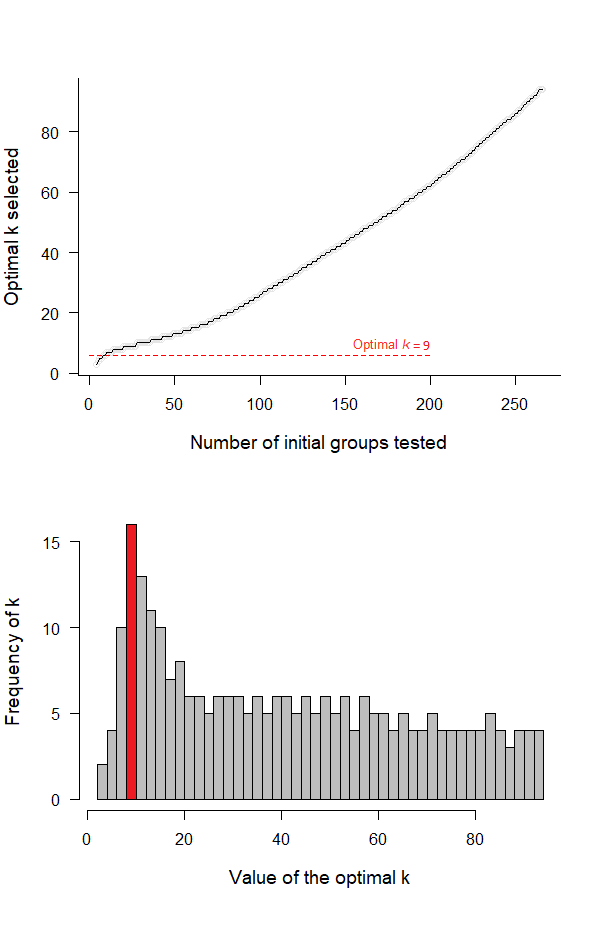


## **S1 Fig G. K-means test.** (a) Variation in the optimal number of clusters (k) identified by the L-method algorithm according to increasing number of maximum k, which is the number of points in the piecewise regression. (b) Histogram for the values of optimal k selected when varying the maximum k from 4 to (n_sites_ – 1). The red bar indicates the optimal k = 9.

| Group | Area (Km²) | Recorded Number of Species | Exclusive Species | Mean Elevation (m) | Mean Annual Temperature (°C) | Annual Rainfall (mm) | Köppen Aridity Index^1^ |
| --- | --- | --- | --- | --- | --- | --- | --- |
| 1 - Core Chapada Diamantina | 30420 | 857 | 524 | 892 | 20 | 838 | 73 |
| 2 - Chapada Diamantina Periphery | 54796 | 627 | 134 | 532 | 22 | 1056 | 78 |
| 3 – Southern Caatinga | 62971 | 418 | 118 | 452 | 23 | 890 | 71 |
| 4 - Eastern Caatinga | 67166 | 430 | 92 | 601 | 22 | 753 | 66 |
| 5 – Reconcavo | 134139 | 693 | 153 | 382 | 23 | 661 | 61 |
| 6 - São Francisco and Sertaneja Depressions | 102922 | 473 | 107 | 385 | 24 | 607 | 57 |
| 7 - Sertanejo Highlands | 161741 | 537 | 79 | 301 | 26 | 800 | 63 |
| 8 - Middle São Francisco and Cearense Depression | 116024 | 586 | 101 | 273 | 24 | 961 | 71 |
| 9 – Ibiapaba | 112862 | 517 | 103 | 327 | 25 | 1090 | 75 |

## **S1 Table C. Description of Caatinga forest floristic groups**: area, number of recorded and exclusive species, as well as topographic and climatic characteristics.

1 - The lower the value the more arid is the climate.


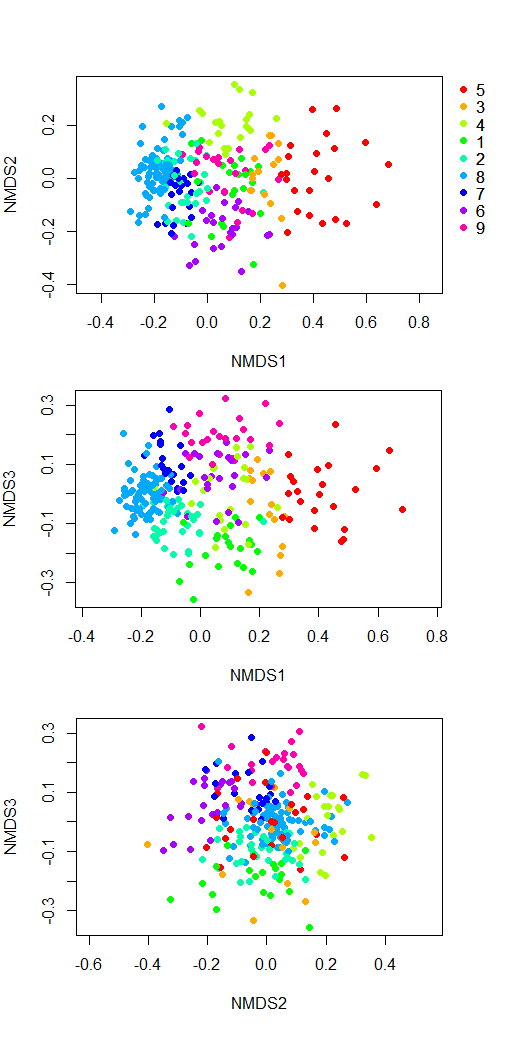


## S1 Fig H. NMDS ordination plots in three dimensions of Caatinga biogeographical sub-regions with 260 localities; stress values = 0.124, tr = 10000. B: 12 floristic groups; stress values = 0.116, tr = 100; relationships inferred from a classification using the UPGMA method (Fig. S2) are indicated by lines**.**


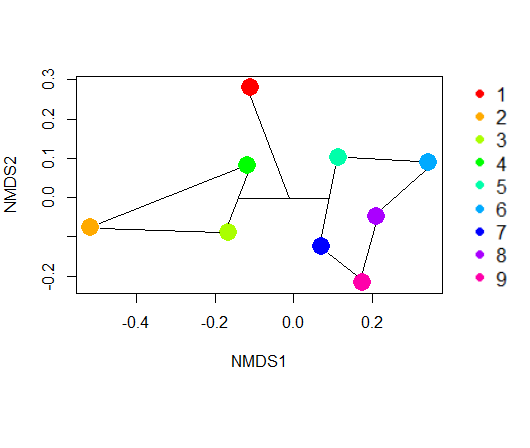


## **S1 Fig I. NMDS ordination plots in two dimensions of the nine Caatinga biogeographical sub-regions;** stress values = 0.10, tr = 10000. The relationships depicted by the lines were inferred from a classification using the UPGMA method (Fig. 3a).

## **S1 Table D. Shared species among Caatinga floristic groups**. Deeper grey shade indicates greater numbers of shared species, corresponding to line widths in Figure 3.

|  | **Core Chapada Diamantina** | **Chapada Diamantina Periphery** | **Southern Caatinga** | **Eastern Caatinga** | **Reconcavo** | **São Francisco and Sertaneja Depressions** | **Sertanejo Highlands** | **Middle São Francisco and Cearense Depressions** | **Ibiapaba** |
| --- | --- | --- | --- | --- | --- | --- | --- | --- | --- |
| Core Chapada Diamantina | 857 | 205 | 107 | 109 | 129 | 58 | 90 | 137 | 108 |
| Chapada Diamantina Periphery |  | 627 | 149 | 107 | 199 | 102 | 166 | 233 | 197 |
| Southern Caatinga |  |  | 418 | 133 | 167 | 110 | 118 | 127 | 127 |
| Eastern Caatinga |  |  |  | 430 | 214 | 107 | 138 | 132 | 119 |
| Reconcavo |  |  |  |  | 693 | 253 | 282 | 242 | 199 |
| São Francisco and Sertaneja Depressions |  |  |  |  |  | 473 | 219 | 164 | 156 |
| Sertanejo Highlands |  |  |  |  |  |  | 537 | 264 | 240 |
| Middle São Francisco and Cearense Depressions |  |  |  |  |  |  |  | 586 | 255 |
| Ibiapaba |  |  |  |  |  |  |  |  | 517 |

## **S1 Table E. Comparison of classification schemes.** Explanatory potential of different Caatinga sub-region schemes and vegetation physiognomy on woody plant composition.

| Main Factor | F | R² | P |
| --- | --- | --- | --- |
| Floristic sub-regions (this work) | 13.701 | 0.277 | 0.001 |
| Physiognomy | 2.345 | 0.126 | 0.001 |
| Floristic sub-regions : Physiognomy | 1.629 | 0.231 | 0.001 |
| Total |  | 0.403 |  |
|  |  |  |  |
| Sub-regions (Velloso *et al.* 2002) | 9.366 | 0.187 | 0.001 |
| Physiognomy | 3.039 | 0.182 | 0.001 |
| Sub-regions : Physiognomy | 1.527 | 0.187 | 0.001 |
| Total |  | 0.369 |  |
|  |  |  |  |
| Floristic sub-regions (Moro *et al.* 2016) | 7.807 | 0.184 | 0.001 |
| Physiognomy | 2.697 | 0.170 | 0.001 |
| Floristic sub-regions : Physiognomy | 1.444 | 0.171 | 0.001 |
| Total |  | 0.354 |  |


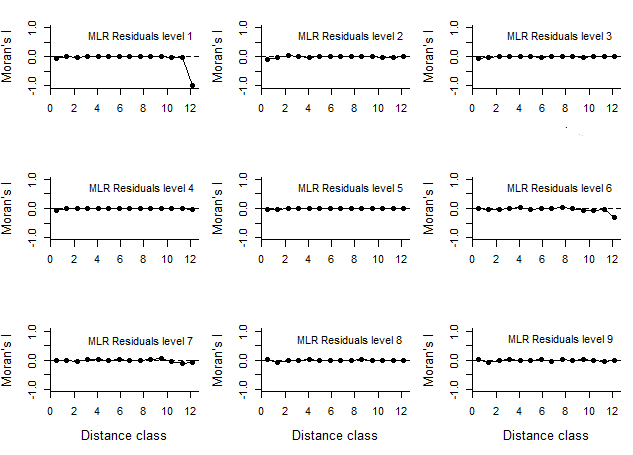


## **S1 Fig J. Moran’s correlograms.** Representation the residuals of the multinomial logistic regression (MLR) using a nine-level categorical variable as dependent variable to represent the Caatinga woody plant biogeographical sub-regions.

## **S1 Table F. Multinomial logistic regression models used to investigate the influence of current and historical environmental conditions as well as the human footprint in explaining the biogeographical sub-regions for woody plants in the Caatinga.** Spatial autocorrelation was controlled for through the inclusion of eight Moran’s Eigenvector Maps (MEMs) in all tested models (MEM1 + MEM2 + MEM3 + MEM4 + MEM5 + MEM6 + MEM8 + MEM11). Models are presented in increasing AICc order. IAC = Köppen Aridity Index, ElevCV = coefficient of variation of elevation, ElevR = elevation range, CEC = cation exchange capacity, Sand = soil sand content, HHP = Historic variation in overall hydric conditions, HHC = Historical variation in overall thermal conditions, HFP = human footprint.

| Model | Deviance | AICc | wAICc |
| --- | --- | --- | --- |
| AI (Best Model) | 0.48 | 759.0 | 0.999 |
| AI + HFP | 0.49 | 782.0 | 0.000 |
| AI + CEC + Sand | 0.51 | 810.0 | 0.000 |
| AI + ElevR + ElevCV | 0.50 | 815.0 | 0.000 |
| HHC | 0.41 | 823.0 | 0.000 |
| CEC | 0.41 | 825.0 | 0.000 |
| AI + HTC + HHC + HAI | 0.54 | 827.0 | 0.000 |
| Space only (8 MEMs) | 0.37 | 828.0 | 0.000 |
| HFP | 0.40 | 829.0 | 0.000 |
| HTC | 0.41 | 835.0 | 0.000 |
| HAI | 0.40 | 839.0 | 0.000 |
| ElevCV | 0.39 | 841.1 | 0.000 |
| Sand | 0.39 | 841.3 | 0.000 |
| AI + CEC + Sand + HFP | 0.52 | 846.1 | 0.000 |
| AI + ElevR + ElevCV + HFP | 0.52 | 846.5 | 0.000 |
| ElevR | 0.39 | 846.7 | 0.000 |
| AI + HFP + HHC + HTC + HAI | 0.55 | 863.0 | 0.000 |
| Sand + CEC + HFP | 0.45 | 864.0 | 0.000 |
| ElevR + ElevCV + HFP | 0.44 | 876.0 | 0.000 |
| HTC + HHC + HAI + HFP | 0.48 | 884.0 | 0.000 |
| AI + CEC + Sand + ElevR + ElevCV | 0.53 | 889.0 | 0.000 |
| Sand + CEC + ElevR + ElevCV | 0.46 | 905.0 | 0.000 |
| AI + CEC + Sand + HTC + HHC + HAI | 0.56 | 918.0 | 0.000 |
| AI + ElevR + ElevCV + HTC + HHC + HAI | 0.55 | 928.0 | 0.000 |
| Sand + CEC + HHC + HTC + HAI | 0.49 | 930.0 | 0.000 |
| CEC + Sand + ElevR + ElevCV + HFP | 0.48 | 936.0 | 0.000 |
| AI + CEC + Sand + ElevR + ElevCV + HFP | 0.54 | 937.0 | 0.000 |
| ElevR + ElevCV + HHC + HTC + HAI | 0.47 | 948.0 | 0.000 |
| CEC + Sand + HFP + HTC + HHC + HAI | 0.51 | 967.0 | 0.000 |
| AI + CEC + Sand + HFP + HTC + HHC + HAI | 0.57 | 971.0 | 0.000 |
| AI + ElevR + ElevCV + HFP + HTC + HHC + HAI | 0.57 | 979.0 | 0.000 |
| ElevR + ElevCV + HFP + HTC + HHC + HAI | 0.49 | 982.0 | 0.000 |
| ElevR + ElevCV + CEC + Sand + HTC + HHC + HAI | 0.50 | 1041.0 | 0.000 |
| AI + CEC + Sand + ElevCV + HFP + HTC + HHC + HAI | 0.58 | 1045.0 | 0.000 |
| AI + CEC + Sand + ElevR + HFP + HTC + HHC + HAI | 0.58 | 1047.0 | 0.000 |
| AI + CEC + ElevR + ElevCV + HFP + HTC + HHC + HAI | 0.58 | 1047.3 | 0.000 |
| AI + Sand + ElevR + ElevCV + HFP + HTC + HHC + HAI | 0.58 | 1048.0 | 0.000 |
| AI + CEC + Sand + ElevR + ElevCV + HFP + HTC + HHC | 0.57 | 1050.0 | 0.000 |
| AI + CEC + Sand + ElevR + ElevCV + HTC + HHC + HAI | 0.57 | 1056.0 | 0.000 |
| AI + CEC + Sand + ElevR + ElevCV + HFP + HHC + HAI | 0.57 | 1056.5 | 0.000 |
| AI + CEC + Sand + ElevR + ElevCV + HFP + HTC + HAI | 0.57 | 1058.0 | 0.000 |
| CEC + Sand + ElevR + ElevCV + HFP + HTC + HHC + HAI | 0.52 | 1100.0 | 0.000 |
| AI + CEC + Sand + ElevR + ElevCV + HFP + HTC + HHC + HAI | 0.59 | 1132.0 | 0.000 |

# References

1. Ferrier S. Mapping spatial pattern in biodiversity for regional conservation planning: where to from here? Syst Biol. 2002;51: 331–363. doi:10.1080/10635150252899806

2. Legendre P, Legendre LF. Numerical ecology. 3rd ed. Amsterdam: Elsevier; 2012.

3. Moura MR, Argôlo AJ, Costa HC. Historical and contemporary correlates of snake biogeographical subregions in the Atlantic Forest hotspot. J Biogeogr. 2017;44: 640–650. doi:10.1111/jbi.12900

4. Pebesma EJ. Multivariable geostatistics in S: the gstat package. Comput Geosci. 2004;30: 683–691. doi:10.1016/j.cageo.2004.03.012

5. Kreft H, Jetz W. A framework for delineating biogeographical regions based on species distributions. J Biogeogr. 2010;37: 2029–2053. doi:10.1111/j.1365-2699.2010.02375.x

6. Dapporto L, Ramazzotti M, Fattorini S, Talavera G, Vila R, Dennis RLH. Recluster: An unbiased clustering procedure for beta-diversity turnover. Ecography (Cop). 2013;36: 1070–1075. doi:10.1111/j.1600-0587.2013.00444.x

7. Holt BG, Lessard J-P, Borregaard MK, Fritz SA, Araújo MB, Dimitrov D, et al. An Update of Wallace’s Zoogeographic Regions of the World. Science (80- ). 2013;339: 74–78. doi:10.1126/science.1228282

8. Linder HP, de Klerk HM, Born J, Burgess ND, Fjeldså J, Rahbek C. The partitioning of Africa: Statistically defined biogeographical regions in sub-Saharan Africa. J Biogeogr. 2012;39: 1189–1205. doi:10.1111/j.1365-2699.2012.02728.x

9. De’ath G. Multivariate regression trees: a new technique for modeling species–environment relationships. Ecology. 2002;83: 1105–1117.

10. Oliveira-Filho AT. Classificação das fitofisionomias da américa do sul cisandina tropical e subtropical: proposta de um novo sistema – prático e flexível – ou uma injeção a mais de caos? Rodriguésia. 2009;60: 237–258.

11. Rodal MJN, Nascimento LM. Levantamento florístico da floresta serrana da reserva biológica de Serra Negra, microrregião de Itaparica, Pernambuco, Brasil. Acta Botanica Brasilica. 2002; 16: 481–500.

12. Nascimento CES, Rodal MJN, Cavalcanti AC. Phytosociology of the remaining xerophytic woodland associated to an environmental gradient at the banks of the São Francisco river - Petrolina, Pernambuco, Brazil. Revista Brasileira de Botânica. 2003; 26: 271–287.

13. Cunha MCL, Silva Júnior MC, Lima RB. Fitossociologia do estrato lenhoso de uma Floresta Estacional Semidecidual Montana na Paraíba, Brasil. Cerne, 2013; 19: 271–280.

14. Xavier KRF. Análise Floristica e Fitossociológica em dois fragmentos de floresta serrana no município de Dona Inês, Paraiba. Dissertação Programa de Pós-Graduação em Agronomia; 2009. 255.

15. Mayo SJ, Vania PB. Mata de Pau Ferro: a pilot study of the brejo forest of Paraíba, Brazil. Kew: Royal Botanic Gardens/Bentham-Moxon Trust . 1980; 29p

16. Andrade LA, Oliveira FX, Nascimento IS, Fabricante JR, Sampaio EVSB, Barbosa MRV. Análise florística e estrutural de matas ciliares ocorrentes em brejo de altitude no município de Areia , Paraíba. Revista Brasileira de Ciências Agrárias. 2006; 1: 31–40.

17. Brasil, Ministério das Minas e Energia. Projeto RADAMBRASIL folha SD. 24 Salvador; geologia, geomorfologia, pedologia, vegetação e uso potencial da terra. Rio de Janeiro. 1981.

18. Melo JIM, Rodal, MJN. Levantamento florístico de um trecho de floresta serrana no planalto de Garanhuns, Estado de Pernambuco. Acta Scientiarum - Biological Sciences. 2003; 25: 173–178

19. Moura F, Sampaio E. Flora lenhosa de uma mata serrana semidecídua em Jataúba, Pernambuco. Revista Nordestina de Biologia. 2001; 15: 77–89.

20. Pinto MSC, Sampaio EVSB, Nascimento LM. Florística e estrutura da vegetação de um brejo de altitude em Pesqueira, PE, Brasil. Revista Nordestina de BIologia. 2012; 21: 47–79.

21. Lyra A. A condição de brejo: efeito do relevo na vegetação de duas áreas do Município do Brejo de Madre de Deus, PE. Dissertação (Mestrado em Botânica). 1982; 105f. Universidade Federal Rural de Pernambuco, Recife.

22. Ferraz EMN, Rodal MJN, Sampaio EVSB, Pereira RDCA. Composição florística em trechos de vegetação de caatinga e brejo de altitude na região do Vale do Pajeú, Pernambuco. Revta brasil. Bot. 1998; 21: 7–15.

23. Correia MS. Estrutura da vegetação da mata serrana em um brejo de altitude em Pesqueira - PE. Dissertação de Mestrado. 1996. Universidade Federal de Pernambuco. Recife

24. Figueiredo MA, Barbosa M. A vegetação ea flora na serra de Baturité, Ceará. Coleção Mossoroense. 1990; 747.

25. Oliveira MLR. Composição florística e análise fitossociológica de uma área de mata úmida na serra do Baturité - CE. Monografia de Graduação. Universidade Federal do Ceará, Fortaleza 1994.

26. Brasil, Ministério das Minas e Energia. Projeto RADAMBRASIL folha SD. 24 Salvador; geologia, geomorfologia, pedologia, vegetação e uso potencial da terra. Rio de Janeiro. 1983.

27. Santos Filho F. Composição florística e estrutural da vegetação de restinga do estado do Piauí. 2009; 120p.

28. Castro ASF, Moro MF, Menezes MOT. O Complexo Vegetacional da Zona Litorânea no Ceará: Pecém, São Gonçalo do Amarante. Acta Botanica Brasilica. 2012; 26: 108–124.

29. Matias LQ, Nunes EP. Levantamento florístico da Área de Proteção Ambiental de Jericoacoara, Ceará. Acta Botanica Brasilica. 2001; 15: 35–43.

30. Santos RM, Barbosa ACMC, Almeida HS, Vieira FA, Santos PF, et al. Estrutura e florística de um remanescente de Caatinga arbórea em Juvenília, norte de Minas Gerais, Brasil. Cerne. 2011; 17: 247–258.

31. Oliveira MEA, Sampaio EVSB, Castro AAJF, Rodal MJN. Flora e Fitossosiologia de uma área de transição Carrasco-Caatinga de areia em Padre Marco, Piauí. Naturalia. 1997; 22: 131–150.

32. Ferraz EMN, Rodal MJN, Sampaio EVSB. Physiognomy and structure of vegetation along an altitudinal gradient in the semi-arid region of northeastern Brazil. Phytocoenologia. 2003; 33: 71–92.

33. Nascimento LM, Rodal MJN. Fisionomia e estrutura de uma floresta estacional montana do maciço da Borborema, Pernambuco - Brasil. Revista Brasileira de Botânica. 2008; 31: 27–39.

34. Paes MLN, Dias IFO. Plano de manejo: Estação Ecológica Raso da Catarina, IBAMA, Brasília. 2008.

35. Pereira Júnior L, Andrade AP, Araújo KD. Composição florística e fitossociológica de um fragmento de caatinga em monteiro, PB. Holos. 2012; 6: 73–87.

36. Figueiredo LS, Rodal MJN, Melo AL. Floristica e fitossociologia de uma área de Vegetação arbustiva caducifólia espinhosa no município de Buíque - PE. Naturalia. 2000; 25: 205–224.

37. Fabricante JR, Andrade LA. Análise Estrutural de um Remanescente de Caatinga no Seridó Paraibano. Oecologia Brasiliensis. 2007; 11: 341–349.

38. Rodal MJN, Andrade KVDA, Sales MF, Gomes APS. Fitossociologia do componente lenhoso de um refúgio vegetacional no município de Buíque, Pernambuco. Revista Brasileira de Biologia. 1998; 58: 517–526.

39. Dantas TVP, Nascimento-Júnior JE, Ribeiro ADS, Prata APDN. Florística e estrutura da vegetação arbustivo-arbórea das Areias Brancas do Parque Nacional Serra de Itabaiana/Sergipe, Brasil. Revista Brasileira de Botânica. 2010; 33: 575–588.

40. Alcoforado-Filho FG, Sampaio EVDSB, Rodal MJN. Florística e fitossociologia de um remanescente de vegetação caducifólia espinhosa arbórea em Caruaru, Pernambuco. Acta Botanica Brasilica. 2003; 17: 287–303.

41. Amorim IL, Sampaio EVSB, Araújo EDL. Flora e estrutura da vegetação arbustivo-arbórea de uma área de caatinga do Seridó, RN, Brasil. Acta Botanica Brasilica. 2005; 19: 615–623.

42. Lacerda AV, Nordi N, Barbosa FM, Watanabe T. Levantamento florístico do componente arbustivo-arbóreo da vegetação ciliar na bacia do rio Taperoá, PB, Brasil. Acta Botanica Brasilica. 2005; 19: 647–656.

43. Pegado CMA, Andrade LA, Félix LP, Pereira IM. Efeitos da invasão biológica de algaroba: Prosopis juliflora (Sw.) DC. sobre a composição e a estrutura do estrato arbustivo-arbóreo da caatinga no Município de Monteiro, PB, Brasil. Acta Botanica Brasilica. 2006; 20: 887–898.

44. Pereira IM, Andrade LA, Barbosa MRDV, Sampaio EVSB. Composição florística e análise fitossociológica do componente arbustivo-arbóre de um remanescente florestal no agreste paraibano. Acta Botanica Brasilica. 2002; 16: 357–369.

45. Rodal MJN, Nascimento LM, Melo AL. Composição florística de um trecho de vegetação arbustiva caducifólia, no município de Ibimirim, PE, Brasil. Acta Botanica Brasilica. 1999; 13: 15–28.

46. Gomes APDS, Rodal MJN, Melo AL. Florística e fitogeografia da vegetação arbustiva subcaducifólia da Chapada de São José, Buíque, PE, Brasil. Acta Botanica Brasilica. 2006; 20: 37–48.

47. Pinheiro K, Alves M. Espécies arbóreas de uma área de Caatinga no sertão de Pernambuco, Brasil: dados preliminares. Revista Brasileira de Biociências. 2008; 426–428.

48. Rodal MJN, Martins FR, Sampaio EVSB. Levantamento quantitativo das plantas lenhosas em trechos de vegetaçao de Caatinga em Pernambuco. Revista Caatinga, 2008; 21: 192–205.

49. Costa KC, Lima ALA, Fernandes CHM, Silva MCNA, Lins e Silva ACB, Rodal MJN. Flora vascular e formas de vida em um hectare de caatinga no Nordeste brasileiro. Revista Brasileira de Ciências Agrárias. 2009a; 4: 48–54.

50. Rodal MJN. Fitossociologia da vegetação arbustivo-arbórea em quatro áreas de Caatinga em Pernanbuco. 1992; 241p.

51. Albuquerque SG, Soares JGG, Filho JAA. Densidade de espécies arbóreas e arbustivas em vegetação de caatinga. Embrapa. 1982; 16: 1–9.

52. Costa IR, Araújo FS, Lima-Verde LW. Flora e aspectos auto-ecológicos de um encrave de cerrado na chapada do Araripe, Nordeste do Brasil. Acta Botanica Brasilica. 2004; 18: 759–770.

53. Araújo FR, Martins F, Shepherd GJ. Variações estruturais e florísticas do carrasco no planalto da Ibiapaba, estado do Ceará. Revista Brasileira de Biologia. 1999; 59: 663–678.

54. Lima JR, Sampaio EVDSB, Rodal MJN, Araújo FS. Composição florística da floresta estacional decídua montana de Serra das Almas, CE, Brasil. Acta Botanica Brasilica. 2009; 23: 756–763.

55. Ribeiro-Filho AA, Funch LS, Rodal MJN. Composição florística da floresta ciliar do rio Mandassaia, Parque Nacional da Chapada Diamantina, Bahia, Brasil. Rodriguésia. 2009; 60: 265–276.

56. Araújo FS, Sampaio EVSB, Figueiredo MA, Rodal MJN, Fernandes AG. Composição florística da vegetação de carrasco, Novo Oriente, CE. Revista Brasileira de Botânica. 1998; 21: 105–116.

57. Lima BG, Coelho MFB, Oliveira OF. Caracterização florística de duas áreas de caatinga na região centro-sul do Ceará, Brasil. Bioscience Journal. 2012; 28: 277–296.

58. Lima BG, Coelho MFB. Estrutura do componente arbustivo-arbóreo de um remanescente de caatinga no estado do Ceará, Brasil. Cerne. 2015; 21: 665–672.

59. Drumond MA, Lima PCF, Souza SM, Lima JLS. Sociabilidade das espécies florestais da Caatinga em Santa Maria da Boa Vista - PE. Centro de Pesquisa Agropecuária do Trópico Semi-Árido - CPATSA. 1982; 47–59.

60. Araújo BA, Neto JD, Alves AS, Araújo PAA. Estrutura Fitossociológica Em Uma Área De Caatinga No Seridó Paraibano. Revista Educação Agrícola Superior. 2012; 27: 25–29.

61. Lemos JR, Meguro M. Florística e fitogeografia da vegetação decidual da Esrasiltação Ecológica de Aiuaba, Ceará, Nordeste do B. Revista Braisileira de Biociências. 2010; 8: 34–43.

62. Lemos JR, Rodal MJN. Fitossociologia do componente lenhoso de um trecho da vegetação de caatinga no Parque Nacional Serra da Capivara, Piauí, Brasil. Acta Botanica Brasilica. 2002; 16: 23–42.

63. Tavares S, Paiva FAF, Tavares EJS, Carvalho GH, Lima JLS. Inventário florestal de Pernambuco: estudo preliminar das matas remanescentes dos municípios de Ouricurí, Bodocó, Santa Maria da Bôa Vista e Petrolina. Boletim de Recursos Naturais - SUDENE. 1970; 8: 149–194.

64. Farias RRS, Castro AAJF. Fitossociologia de trechos da vegetação do Complexo de Campo Maior, Campo Maior, PI, Brasil. Acta Botanica Brasilica. 2004; 18: 949–963.

65. Araújo EDL, Sampaio EVSB, Rodal MJN. Composição florística e fitossociologia de três áreas de caatinga de Pernambuco. Revista Brasileira de Biologia, 1995; 55: 595–607.

66. Tavares S, Paiva FAF, Tavares EJS, Lima JLS, Carvalho GH. Estudo preliminar das matas remanescentes do município de São José de Belmonte. Sudene. 1969b; 7: 113–139.

67. Oliveira PTB, Trovão DMBM, Carvalho ECD, Souza BC, Ferreira LMR. Florística e Fitossociologia de Quatro Remanescentes de Vegetação. Revista Caatinga. 2009; 22: 169–178.

68. Souza GV. Estrutura da vegetação da caating hipoxerófila do estado de Sergipe. 1983.

69. Tavares S, Paiva FAF, Tavares EJS, Lima JLS. Estudo Preliminar das matas remanescentes do município de Quixadá. Sudene. 1969a; 7: 93–111.

70. Tavares S, Paiva FAF, Tavares EJS, Lima JLS. Estudo preliminar das Matas remanescentes do municipio de Tauá. Sudene. 1974b; 12: 5–19.

71. Fonseca MR. Análise da vegetação arbustivo-arbórea da caatinga hiperxerófila do nordeste do estado de Sergipe. 1991.

72. Rodal MJN, Sales MF. Composição da flora vascular em um remanescente de floresta montana no semi-árido do nordeste do Brasil. Hoehnea. 2007; 34: 433–446.

73. Tavares, S., Paiva, F.A.F., Tavares, E.J. de S. & Lima, J.L.S. (1974a) Estudo preliminar das mata remanescentes do município de Barbalha. Sudene, 12, 20–46.

74. Júnior APS, Silva CIA, Rodrigues CM, Oliveira ML, Luna RB. Plano de Manejo da RPPN Reserva Natural Brajo. 2012.

75. Souza JÁN, Rodal MJN. Levantamento florístico em trecho de vegetação ripária de caatinga no rio Pajeú, Floresta/Pernambuco-Brasil. Revista Caatinga. 2010; 23: 54–62.

76. Guedes RS, Zanella FCV, Costa JEV, Santana GM, Silva JA. Caracterização florístico-fitossociológica do componente lenhoso de um trecho de caatinga no semiárido paraibano. Revista Caatinga. 2012; 25: 99–108.

77. Alves AR, Ribeiro IB, Sousa JRL, Barros SS, Sousa PS. Análise da estrutura vegetacional em uma área de transição Cerrado-Caatinga no município de Bom Jesus-PI. Revista Caatinga. 2013; 26: 99–106.

78. Ferraz RC, Mello AA, Ferreira RA, Prata APN. Levantamento fitossociológico em área de caatinga no monumento natural Grota do Angico, Sergipe, Brasil. Revista Caatinga. 2013; 26: 89–98.

79. Santos ACJ, Melo JIM. Flora vascular de uma área de caatinga no estado da paraíba - Nordeste do Brasil. Revista Caatinga. 2010; 23: 32–40.

80. Calixto Júnior JT, Drumond MA. Estrutura fitossociológica de um fragmento de caatinga senso stricto 30 anos após corte raso, Petrolina-PE, Brasil. Revista Caatinga. 2011; 24: 67–74.

81. Barbosa MRDV, Lima IB, Lima JR, Cunha JP, Agra MDF, Thomas WW. Vegetação e Flora no Cariri Paraibano. Oecologia Brasiliensis. 2007; 11: 313–322.

82. Emperaire L. Vegetation et gestion des ressources naturelles dans la Caatinga du sud-est du Paiui (Bresil). 1989.

83. Moro MF, Castro ASF, Araújo FS, Fortaleza D. Composição florística e estrutura de um fragmento de vegetação savânica sobre os tabuleiros pré-litorâneos. Rodrigésia. 2011; 62: 407–423.

84. Barbosa MD, Marangon LC, Feliciano ALP, Freire FJ, Duarte GMT. Florística e fitossociologia de espécies arbóreas e arbustivas em uma área de Caatinga em Arcoverde, PE, Brasil. Revista Árvore. 2012; 36: 851–858.

85. Ramalho IC, Andrade AP, Félix LP, Lacerda AV, Maracajá PB. Flora arbóreo-arbustiva em áreas de caatinga no semiárido baiano, Brasil. Revista Caatinga. 2009; 22: 182–190.

86. Lima PCF, Lima JLS. Composição florística e fitossociologia de uma área de caatinga em Contendas do Sincorá, Bahia, microrregião homogênea da Chapada Diamantina. Acta Botanica Brasilica. 1998; 12: 441–450.

87. Santos RM, Vieira FDA, Fagundes M, Nunes YRF, Gusmão E. Riqueza e similaridade florística de oito remanescentes florestais no norte de Minas Gerais, Brasil. Revista Árvore. 2007; 31: 135–144.

88. Dias PMS, Diodato MA, Grigio AM. Levantamento Fitossociológico De Remanescentes Florestais No Município De Mossoró-RN. Revista Caatinga. 2014; 27: 183–190.

89. Mendes MRA, Castro AAJF. Vascular flora of semi-arid region , São José do Piauí , state of Piauí , Brazil. Check List. 2010; 6: 39–44.

90. Guerra AMNM, Pessoa MF, Maracajá PB. Estudo fitossociológico em dois ambientes da caatinga localizada no assentamento Moacir Lucena, Apodi-RN – BRASIL. Revista Verde de Agroecologia e Desenvolvimento Sustentável. 2014; 9: 141–150.

91. Moreira ARP, Maracajá PB, Guerra AMNM, Filho FAZ, Pereira TFC. Composição florística e análise fitossociológica arbustivo-arbóreo no município de Caraúbas-RN. Revista Verde de Agroecologia e Desenvolvimento Sustentável. 2007; 2: 113–126.

92. Rocha PLB, Queiroz LP, Pirani JR. Plant species and habitat structure in a sand dune field in the Brazilian Caatinga: a homogeneous habitat harbouring an endemic biota. Revista Brasileira de Botanica. 2004; 27: 739–755.

93. Souza GF, Medeiros JF. Fitossociologia e florística em áreas de caatinga na microbacia hidrográfica do riacho cajazeiras - RN. GEOTemas. 2013; 3: 161–176.

94. Freitas RAC, Filho FAS, Maracajá PB, Filho ETD. Estudo florístico e fitosociológico do extrato arbustivo-arboreo de dois ambientes em Messias Targino divisa RN/PB. Revista Verde de Agroecologia e Desenvolvimento Sustentável. 2007; 2: 135–147.

95. Costa GM. Ecologia da vegetação de caatingas em diferente substratos, Bahia, Brasil. 2014.

96. Silva GA, Maia LC, Silva FSB, Lima PCF. Potencial de infectividade de fungos micorrízicos arbusculares oriundos de área de caatinga nativa e degradada por mineração , no Estado da Bahia , Brasil. Revista Brasileira de Botanica. 2001; 24: 135–143.

97. Chaves EMF. Florística e potencialidades economicas da vegetação de Carrasco no município de Cocao, Piauí, Brasil. 2005.

98. Cardoso D, França F, Novais J, Ferreira M, Santos R, et al. Composição florística e análise fitogeográfica de uma floresta semidecídua na Bahia, Brasil. Rodriguésia. 2009; 60: 1055–1076.

99. Martins CTV. Florística e estrutura de vegetações ciliares no alto da bacia hidrográfica no rio Paraguaçu, Mucugê, Bahia, Brasil. 2015.

100. Funch LS. Composição florística e fenologia de mata ciliar e mata de encosta, adjacentes ao rio Lençois, Lençois, BA. UNICAMP, Campinas - SP. 1996.

101. Ferreira EVR. Composição florística, estrutura da comunidade e síndrome de dispersão de sementes de um remanescente de Caatinga em Poço Verde - Sergipe. Dissertação de Mestrado, Pós-Graduação em Ecologia e Conservação, Universidade Federal de Sergipe. 2011.

102. Araújo FS, Costa RC, Lima JR, Vasconcelos SF, Girão LC, et al. Floristics and life-forms along a topographic gradient, To test whether the flora is organized in discrete or continuous units along a topographic gradient, central-western Ceará, Brazil. Rodriguésia. 2011; 62: 341–366.

103. Andrade LA, Fabricante JR, Oliveira FX. Invasão biológica por Prosopis juliflora (Sw.) DC.: impactos sobre a diversidade e a estrutura do componente arbustivo-arbóreo da caatinga no Estado do Rio Grande do Norte, Brasil. Acta Botanica Brasilica. 2009; 23: 935–943.

104. Guedes RR. Lista preliminar das angiospermas ocorrentes no Raso da Catarina e arredores, Bahia. Rodriguésia. 1985; 37: 5–8.

105. Nogueira Júnior FC. Estrutura e composição de uma vegetação ripária, relações dendrocronológicas e climáticas na Serra dos Macacos em Tobias Barreto, Sergipe-Brasil. Dissertação de Mestrado, Pós-Graduação em Ecologia e Conservação, Universidade Federal de Sergipe. 2011.

106. Rodrigues PMS, Azevedo IFP, Veloso MDM, Santos RM, Menino GCO, et al. Riqueza florística da vegetação ciliar do rio Pandeiros, norte de Minas Gerais. Biota. 2009; 2: 18–35.

107. Loiola MIB, Araújo FS, Lima-Verde LW, Souza SSG, Matias LQ, et al. Sociobiodiversidade na Chapada do Araripe, (ed. by U.P. de Albuquerque) and M.V. Meiado) NUPEEA, Recife. 2015.

108. Araújo FS, Oliveira RF, Lima-Verde LW. Composição, espectro biológico e síndromes de dispersão da vegetação de um inselberg no domínio da Caatinga, Ceará. Rodriguésia. 2008; 59: 659–671.

109. Rodarte ATA, Silva FO, Viana BF. A flora melitófila de uma área de dunas com vegetação de caatinga, Estado da Bahia, Nordeste do Brasil. Acta Botanica Brasilica. 2008; 22: 301–312.

110. Damascena L. Caracterização da Savana Estépica Parque no baixo médio São Francisco, Bahia, Brasil. Dissertação de Mestrado, Universidade Estadual de Feira de Santana. 2011; 127p.

111. Santos RM, Vieira FA, Morais VM, Medeiros MA. Estrutura E Florística De Um Remanescente Florestal na fazenda Ribeirão, município de Juvenília, MG, Brasil. Revista Caatinga. 2008; 24: 154–162.

112. Fortius GA, Sá IB. Prospecção botânica em área de exploração petrolífera no municipio de Pendências, RN. Embrapa. 1988; 1–11.

113. Cordeiro, A.C.L. Plano de Manejo da Reserva do Patrimônio Natural Não Me Deixes. 2012b; 110p.

114. Cordeiro, A.C. Plano de manejo da RPPN Edson Queiroz. 2012a; 105p.

115. Trovão DMBM, Freire AM, Melo JIM. Florística e fitossociologia do componente lenhoso da mata ciliar do riacho de bodocongó, semiárido paraibano. Revista Caatinga. 2010; 23: 78–86.

116. Costa TCC, Oliveira MAJ, Accioly LJO, Silva FHBB. Análise da degradação da caatinga no núcleo de desertificação do Seridó (RN/PB). Revista Brasileira de Engenharia Agrícola e Ambiental. 2009b;13: 961–974.

117. IBAMA/MMA. Informações gerais sobre a floresta nacional. Plano de Manejo: Floresta Nacional Contendas do Sincorá, v.1. 2006.

118. Herbarium data. Specieslink. <http://splink.cria.org.br/>. 2016.
