# Supplementary material for: Aridity drives plant biogeographical sub regions in the Caatinga, the largest tropical dry forest and woodland block in South America
Source: PLoS One. 2018 Apr 27;13(4):e0196130. doi: 10.1371/journal.pone.0196130 (PMC5922524; doi:10.1371/journal.pone.0196130)
Supplement: S2 Text — Table A. Woody plant species recorded after compilation of 260 inventories in the Caatinga. (DOCX) [file pone.0196130.s003.docx]

**Aridity drives plant biogeographical sub regions in the Caatinga, the largest tropical dry forest and woodland block in South America**

Augusto C. Silva, Alexandre F. Souza

**Supporting information 2**

**S2 Table A. Woody plant species recorded after compilation of 260 inventories in the Caatinga.** Species presence within each biogeographical sub-region is also shown (sub-region number as in Table S3). CCD = Core Chapada Diamantina, CDP = Chapada Dimantina Periphery, S = Southern Caatinga, EC = Eastern Caatinga, R = Reconcavo, SSD = São Francisco and Sertaneja Depressions, SH = Sertanejo Highlands, MSCD = Middle São Francisco and Cearense Depression, I = Ibiapaba.

| Botanical Family | Species | CCD | CDP | S | EC | R | SSD | SH | MSCD | I |
| --- | --- | --- | --- | --- | --- | --- | --- | --- | --- | --- |
| Fabaceae | *Abarema cochliacarpos* | 1 | 0 | 3 | 0 | 5 | 0 | 0 | 0 | 0 |
| Fabaceae | *Abarema filamentosa* | 0 | 2 | 0 | 0 | 0 | 0 | 0 | 0 | 0 |
| Fabaceae | *Abarema jupunba* | 0 | 2 | 3 | 0 | 0 | 0 | 0 | 0 | 0 |
| Malvaceae | *Abutilon pauciflorum* | 0 | 0 | 0 | 4 | 0 | 0 | 0 | 0 | 0 |
| Malvaceae | *Abutilon regnellii* | 0 | 0 | 0 | 0 | 5 | 0 | 0 | 0 | 0 |
| Malvaceae | *Abutilon woronowii* | 0 | 0 | 0 | 4 | 0 | 0 | 0 | 0 | 0 |
| Fabaceae | *Acacia farnesiana* | 0 | 2 | 0 | 0 | 5 | 0 | 7 | 0 | 9 |
| Euphorbiaceae | *Acalypha amblyodonta* | 0 | 0 | 0 | 4 | 0 | 0 | 0 | 0 | 0 |
| Euphorbiaceae | *Acalypha brasiliensis* | 0 | 0 | 0 | 4 | 5 | 0 | 0 | 0 | 0 |
| Euphorbiaceae | *Acalypha multicaulis* | 0 | 0 | 0 | 4 | 5 | 6 | 0 | 0 | 0 |
| Euphorbiaceae | *Acalypha pruriens* | 0 | 0 | 0 | 0 | 5 | 0 | 0 | 0 | 0 |
| Euphorbiaceae | *Acalypha villosa* | 0 | 2 | 0 | 0 | 0 | 0 | 0 | 0 | 0 |
| Polygalaceae | *Acanthocladus albicans* | 0 | 0 | 0 | 4 | 5 | 0 | 0 | 0 | 0 |
| Polygalaceae | *Acanthocladus dichromus* | 0 | 0 | 0 | 0 | 5 | 0 | 0 | 0 | 0 |
| Asteraceae | *Acanthospermum australe* | 1 | 0 | 0 | 0 | 0 | 0 | 0 | 0 | 0 |
| Asteraceae | *Acanthospermum hispidum* | 1 | 0 | 0 | 4 | 0 | 0 | 0 | 8 | 0 |
| Plantaginaceae | *Achetaria erecta* | 1 | 0 | 0 | 0 | 0 | 0 | 0 | 0 | 0 |
| Asteraceae | *Achyrocline satureioides* | 1 | 0 | 0 | 0 | 0 | 0 | 0 | 0 | 0 |
| Solanaceae | *Acnistus arborescens* | 0 | 2 | 0 | 4 | 5 | 0 | 0 | 0 | 0 |
| Fabaceae | *Acosmium dasycarpum* | 0 | 0 | 0 | 0 | 0 | 6 | 0 | 0 | 0 |
| Papilionoideae | *Acosmium lentiscifolium* | 0 | 0 | 3 | 0 | 0 | 0 | 0 | 0 | 0 |
| Asteraceae | *Acritopappus catolesensis* | 1 | 0 | 0 | 0 | 0 | 0 | 0 | 0 | 0 |
| Asteraceae | *Acritopappus confertus* | 1 | 0 | 0 | 0 | 0 | 0 | 7 | 0 | 0 |
| Asteraceae | *Acritopappus connatifolius* | 1 | 0 | 0 | 0 | 0 | 0 | 0 | 0 | 0 |
| Asteraceae | *Acritopappus micropappus* | 0 | 0 | 3 | 0 | 0 | 0 | 0 | 0 | 0 |
| Arecaceae | *Acrocomia aculeata* | 0 | 0 | 3 | 0 | 0 | 0 | 0 | 0 | 0 |
| Arecaceae | *Acrocomia hassleri* | 1 | 0 | 0 | 0 | 0 | 0 | 0 | 0 | 0 |
| Arecaceae | *Acrocomia intumescens* | 0 | 2 | 3 | 0 | 0 | 0 | 0 | 0 | 0 |
| Eriocaulaceae | *Actinocephalus ramosus* | 1 | 0 | 0 | 0 | 0 | 0 | 0 | 0 | 0 |
| Euphorbiaceae | *Actinostemon concolor* | 0 | 0 | 0 | 4 | 0 | 0 | 0 | 0 | 0 |
| Euphorbiaceae | *Actinostemon klotzschii* | 0 | 0 | 0 | 0 | 0 | 0 | 0 | 8 | 0 |
| Euphorbiaceae | *Actinostemon lasiocarpus* | 0 | 0 | 0 | 4 | 0 | 0 | 0 | 0 | 0 |
| Euphorbiaceae | *Actinostemon schomburgkii* | 0 | 0 | 0 | 0 | 0 | 0 | 0 | 8 | 0 |
| Bignoniaceae | *Adenocalymma involucratum* | 0 | 0 | 0 | 0 | 0 | 0 | 7 | 0 | 0 |
| Bignoniaceae | *Adenocalymma marginatum* | 0 | 0 | 0 | 0 | 0 | 0 | 0 | 8 | 0 |
| Bignoniaceae | *Adenocalymma pedunculatum* | 0 | 0 | 0 | 0 | 0 | 0 | 0 | 8 | 0 |
| Bignoniaceae | *Adenocalymma pubescens* | 0 | 0 | 0 | 0 | 0 | 0 | 7 | 0 | 0 |
| Bignoniaceae | *Adenocalymma purpurascens* | 0 | 0 | 0 | 0 | 0 | 0 | 7 | 0 | 0 |
| Lamiaceae | *Aegiphila integrifolia* | 0 | 2 | 0 | 0 | 5 | 0 | 0 | 0 | 0 |
| Lamiaceae | *Aegiphila luschnathii* | 0 | 0 | 3 | 0 | 0 | 0 | 0 | 0 | 0 |
| Lamiaceae | *Aegiphila verticillata* | 1 | 2 | 0 | 0 | 5 | 6 | 0 | 8 | 9 |
| Fabaceae | *Aeschynomene benthamii* | 0 | 2 | 0 | 0 | 0 | 0 | 0 | 0 | 0 |
| Fabaceae | *Aeschynomene brasiliana* | 0 | 0 | 0 | 0 | 0 | 0 | 0 | 8 | 0 |
| Fabaceae | *Aeschynomene elegans* | 0 | 0 | 0 | 4 | 0 | 0 | 0 | 0 | 0 |
| Fabaceae | *Aeschynomene evenia* | 0 | 2 | 0 | 0 | 0 | 0 | 0 | 0 | 0 |
| Fabaceae | *Aeschynomene histrix* | 1 | 0 | 0 | 0 | 0 | 0 | 0 | 8 | 0 |
| Fabaceae | *Aeschynomene marginata* | 0 | 0 | 0 | 0 | 0 | 6 | 0 | 8 | 0 |
| Fabaceae | *Aeschynomene martii* | 0 | 0 | 0 | 4 | 5 | 0 | 0 | 8 | 9 |
| Fabaceae | *Aeschynomene mollicula* | 0 | 0 | 0 | 0 | 5 | 0 | 0 | 0 | 0 |
| Fabaceae | *Aeschynomene monteiroi* | 0 | 0 | 0 | 0 | 0 | 6 | 0 | 0 | 0 |
| Fabaceae | *Aeschynomene paniculata* | 0 | 2 | 0 | 0 | 0 | 0 | 0 | 8 | 0 |
| Fabaceae | *Aeschynomene rudis* | 0 | 0 | 0 | 0 | 0 | 0 | 0 | 8 | 0 |
| Fabaceae | *Aeschynomene scabra* | 0 | 0 | 0 | 0 | 0 | 0 | 7 | 8 | 0 |
| Fabaceae | *Aeschynomene sensitiva* | 0 | 2 | 0 | 0 | 0 | 6 | 0 | 8 | 0 |
| Fabaceae | *Aeschynomene viscidula* | 0 | 0 | 0 | 0 | 5 | 0 | 0 | 0 | 0 |
| Ericaceae | *Agarista coriifolia* | 1 | 2 | 0 | 0 | 0 | 0 | 0 | 0 | 0 |
| Ericaceae | *Agarista oleifolia* | 1 | 0 | 0 | 0 | 0 | 0 | 0 | 0 | 0 |
| Ericaceae | *Agarista revoluta* | 0 | 0 | 0 | 4 | 0 | 0 | 0 | 0 | 0 |
| Eupatorieae | *Ageratum conyzoides* | 0 | 0 | 0 | 4 | 5 | 0 | 0 | 0 | 0 |
| Opiliaceae | *Agonandra brasiliensis* | 0 | 2 | 3 | 0 | 0 | 0 | 7 | 8 | 9 |
| Opiliaceae | *Agonandra excelsa* | 0 | 0 | 0 | 4 | 0 | 0 | 0 | 0 | 0 |
| Asteraceae | *Agrianthus empetrifolius* | 1 | 0 | 0 | 0 | 0 | 0 | 0 | 0 | 0 |
| Asteraceae | *Agrianthus leutzelburgii* | 1 | 0 | 0 | 0 | 0 | 0 | 0 | 0 | 0 |
| Asteraceae | *Agrianthus pungens* | 1 | 0 | 0 | 0 | 0 | 0 | 0 | 0 | 0 |
| Lauraceae | *Aiouea guianensis* | 0 | 2 | 0 | 0 | 0 | 0 | 0 | 0 | 0 |
| Lauraceae | *Aiouea saligna* | 0 | 2 | 0 | 0 | 0 | 0 | 0 | 0 | 0 |
| Asteraceae | *Albertinia brasiliensis* | 1 | 0 | 0 | 0 | 0 | 0 | 0 | 0 | 0 |
| Fabaceae | *Albizia chinensis* | 0 | 0 | 0 | 0 | 0 | 6 | 0 | 0 | 0 |
| Fabaceae | *Albizia inundata* | 0 | 0 | 0 | 0 | 0 | 0 | 7 | 0 | 9 |
| Fabaceae | *Albizia lebbeck* | 0 | 2 | 0 | 0 | 0 | 0 | 0 | 0 | 9 |
| Fabaceae | *Albizia multiflora* | 0 | 0 | 0 | 0 | 0 | 6 | 0 | 0 | 0 |
| Fabaceae | *Albizia niopoides* | 0 | 0 | 3 | 0 | 0 | 6 | 0 | 0 | 0 |
| Fabaceae | *Albizia pedicellaris* | 1 | 2 | 0 | 4 | 0 | 0 | 0 | 0 | 0 |
| Fabaceae | *Albizia polycephala* | 0 | 2 | 3 | 4 | 5 | 0 | 7 | 0 | 9 |
| Euphorbiaceae | *Alchornea glandulosa* | 0 | 2 | 0 | 0 | 0 | 0 | 0 | 0 | 0 |
| Euphorbiaceae | *Alchornea triplinervia* | 1 | 2 | 0 | 4 | 0 | 0 | 0 | 0 | 0 |
| Euphorbiaceae | *Aleurites moluccanus* | 0 | 2 | 0 | 0 | 5 | 0 | 0 | 0 | 0 |
| Myrtaceae | *Algrizea macrochlamys* | 1 | 0 | 0 | 0 | 0 | 0 | 0 | 0 | 0 |
| Rubiaceae | *Alibertia edulis* | 1 | 2 | 0 | 0 | 0 | 0 | 7 | 0 | 9 |
| Arecaceae | *Allagoptera arenaria* | 1 | 0 | 0 | 0 | 0 | 0 | 0 | 0 | 0 |
| Arecaceae | *Allagoptera campestris* | 1 | 0 | 0 | 0 | 0 | 0 | 0 | 0 | 0 |
| Apocynaceae | *Allamanda blanchetii* | 0 | 2 | 0 | 4 | 5 | 6 | 7 | 8 | 9 |
| Apocynaceae | *Allamanda cathartica* | 0 | 0 | 0 | 0 | 0 | 0 | 0 | 0 | 9 |
| Apocynaceae | *Allamanda puberula* | 1 | 0 | 0 | 0 | 0 | 6 | 7 | 8 | 9 |
| Sapindaceae | *Allophylus edulis* | 0 | 2 | 3 | 4 | 5 | 6 | 7 | 8 | 9 |
| Sapindaceae | *Allophylus laevigatus* | 0 | 0 | 3 | 4 | 5 | 6 | 0 | 0 | 0 |
| Sapindaceae | *Allophylus puberulus* | 0 | 2 | 0 | 4 | 0 | 0 | 0 | 0 | 0 |
| Sapindaceae | *Allophylus quercifolius* | 0 | 0 | 0 | 4 | 5 | 6 | 7 | 0 | 0 |
| Sapindaceae | *Allophylus racemosus* | 0 | 0 | 0 | 4 | 0 | 6 | 0 | 8 | 9 |
| Rutaceae | *Almeidea coerulea* | 1 | 0 | 0 | 0 | 0 | 0 | 0 | 0 | 0 |
| Verbenaceae | *Aloysia gratissima* | 1 | 0 | 0 | 0 | 0 | 0 | 0 | 0 | 0 |
| Verbenaceae | *Aloysia virgata* | 0 | 0 | 3 | 4 | 0 | 0 | 0 | 0 | 9 |
| Rubiaceae | *Alseis floribunda* | 1 | 2 | 3 | 4 | 5 | 0 | 0 | 0 | 9 |
| Rubiaceae | *Alseis latifolia* | 1 | 0 | 0 | 0 | 0 | 0 | 0 | 0 | 0 |
| Rubiaceae | *Alseis pickelii* | 0 | 2 | 0 | 0 | 5 | 0 | 0 | 8 | 0 |
| Amaranthaceae | *Alternanthera brasiliana* | 0 | 0 | 0 | 4 | 5 | 6 | 7 | 8 | 0 |
| Amaranthaceae | *Alternanthera littoralis* | 0 | 0 | 0 | 0 | 0 | 0 | 7 | 8 | 0 |
| Amaranthaceae | *Alternanthera pungens* | 0 | 0 | 0 | 0 | 0 | 0 | 0 | 8 | 0 |
| Amaranthaceae | *Alternanthera regelii* | 0 | 0 | 0 | 0 | 0 | 0 | 0 | 8 | 0 |
| Amaranthaceae | *Alternanthera sessilis* | 0 | 0 | 0 | 0 | 0 | 0 | 0 | 8 | 0 |
| Amaranthaceae | *Alternanthera tenella* | 0 | 0 | 0 | 0 | 5 | 0 | 0 | 8 | 0 |
| Rubiaceae | *Amaioua guianensis* | 0 | 2 | 3 | 0 | 0 | 0 | 0 | 0 | 0 |
| Rubiaceae | *Amaioua intermedia* | 1 | 0 | 3 | 0 | 0 | 0 | 0 | 8 | 0 |
| Amaranthaceae | *Amaranthus spinosus* | 0 | 0 | 0 | 4 | 0 | 0 | 0 | 0 | 0 |
| Lamiaceae | *Amasonia arborea* | 0 | 2 | 0 | 0 | 0 | 0 | 0 | 8 | 0 |
| Lamiaceae | *Amasonia campestris* | 0 | 0 | 0 | 0 | 0 | 0 | 0 | 8 | 0 |
| Fabaceae | *Amburana cearensis* | 0 | 0 | 3 | 0 | 5 | 6 | 7 | 8 | 9 |
| Scrophulariaceae | *Ameroglossum pernambucense* | 0 | 0 | 0 | 4 | 0 | 0 | 0 | 0 | 0 |
| Bignoniaceae | *Amphilophium crucigerum* | 0 | 0 | 0 | 0 | 0 | 0 | 7 | 0 | 9 |
| Anacardiaceae | *Anacardium humile* | 0 | 2 | 0 | 0 | 0 | 0 | 0 | 0 | 0 |
| Anacardiaceae | *Anacardium occidentale* | 0 | 2 | 3 | 4 | 5 | 6 | 7 | 8 | 9 |
| Fabaceae | *Anadenanthera colubrina* | 0 | 2 | 3 | 4 | 5 | 6 | 7 | 8 | 9 |
| Fabaceae | *Anadenanthera peregrina* | 0 | 0 | 3 | 0 | 0 | 6 | 0 | 0 | 0 |
| Annonaceae | *Anaxagorea dolichocarpa* | 1 | 2 | 0 | 0 | 0 | 0 | 0 | 0 | 0 |
| Fabaceae | *Andira anthelmia* | 0 | 0 | 3 | 0 | 0 | 0 | 0 | 0 | 0 |
| Fabaceae | *Andira anthelminthica* | 0 | 0 | 0 | 0 | 0 | 0 | 7 | 0 | 0 |
| Fabaceae | *Andira cordata* | 0 | 2 | 0 | 0 | 0 | 0 | 0 | 0 | 9 |
| Fabaceae | *Andira fraxinifolia* | 1 | 2 | 3 | 4 | 0 | 0 | 0 | 0 | 0 |
| Fabaceae | *Andira humilis* | 1 | 0 | 0 | 0 | 0 | 0 | 7 | 8 | 0 |
| Fabaceae | *Andira legalis* | 0 | 0 | 0 | 0 | 0 | 0 | 0 | 8 | 0 |
| Fabaceae | *Andira surinamensis* | 0 | 2 | 0 | 0 | 0 | 0 | 0 | 8 | 9 |
| Fabaceae | *Andira vermifuga* | 0 | 0 | 3 | 0 | 0 | 0 | 7 | 0 | 9 |
| Bignoniaceae | *Anemopaegma album* | 1 | 0 | 0 | 0 | 0 | 0 | 0 | 0 | 0 |
| Bignoniaceae | *Anemopaegma ataidei* | 0 | 0 | 0 | 0 | 0 | 0 | 7 | 0 | 0 |
| Bignoniaceae | *Anemopaegma citrinum* | 0 | 0 | 0 | 0 | 0 | 0 | 7 | 0 | 0 |
| Bignoniaceae | *Anemopaegma scabriusculum* | 1 | 0 | 0 | 0 | 0 | 0 | 0 | 0 | 0 |
| Bignoniaceae | *Anemopaegma velutinum* | 0 | 0 | 0 | 0 | 5 | 0 | 0 | 0 | 0 |
| Plantaginaceae | *Angelonia biflora* | 0 | 0 | 0 | 0 | 5 | 0 | 0 | 0 | 0 |
| Plantaginaceae | *Angelonia blanchetii* | 1 | 0 | 0 | 0 | 0 | 0 | 0 | 0 | 0 |
| Plantaginaceae | *Angelonia campestris* | 0 | 0 | 0 | 4 | 5 | 0 | 7 | 8 | 0 |
| Plantaginaceae | *Angelonia cornigera* | 1 | 0 | 0 | 0 | 5 | 0 | 0 | 0 | 0 |
| Plantaginaceae | *Angelonia tomentosa* | 1 | 0 | 0 | 0 | 0 | 0 | 0 | 0 | 0 |
| Plantaginaceae | *Angelonia verticillata* | 1 | 0 | 0 | 0 | 0 | 0 | 0 | 0 | 0 |
| Rutaceae | *Angostura bracteata* | 0 | 0 | 3 | 0 | 0 | 0 | 0 | 0 | 0 |
| Lauraceae | *Aniba firmula* | 0 | 2 | 0 | 0 | 0 | 0 | 0 | 0 | 0 |
| Acanthaceae | *Anisacanthus trilobus* | 0 | 0 | 0 | 0 | 0 | 0 | 7 | 0 | 0 |
| Convolvulaceae | *Aniseia martinicensis* | 1 | 0 | 0 | 0 | 0 | 0 | 0 | 0 | 0 |
| Annonaceae | *Annona coriacea* | 1 | 2 | 3 | 0 | 0 | 0 | 0 | 8 | 9 |
| Annonaceae | *Annona crassiflora* | 1 | 0 | 3 | 0 | 0 | 6 | 0 | 0 | 0 |
| Annonaceae | *Annona dolabripetala* | 0 | 0 | 3 | 0 | 0 | 0 | 0 | 0 | 0 |
| Annonaceae | *Annona exsucca* | 0 | 2 | 0 | 0 | 0 | 0 | 0 | 0 | 0 |
| Annonaceae | *Annona glabra* | 0 | 0 | 0 | 0 | 0 | 6 | 0 | 8 | 0 |
| Annonaceae | *Annona laurifolia* | 0 | 0 | 0 | 0 | 0 | 6 | 0 | 0 | 0 |
| Annonaceae | *Annona leptopetala* | 1 | 2 | 3 | 4 | 5 | 6 | 7 | 8 | 9 |
| Annonaceae | *Annona montana* | 0 | 2 | 3 | 0 | 0 | 0 | 7 | 0 | 0 |
| Annonaceae | *Annona muricata* | 1 | 0 | 0 | 0 | 5 | 6 | 0 | 0 | 0 |
| Annonaceae | *Annona pickelii* | 0 | 0 | 3 | 0 | 0 | 0 | 0 | 0 | 0 |
| Annonaceae | *Annona spinescens* | 0 | 0 | 0 | 0 | 5 | 6 | 0 | 8 | 0 |
| Annonaceae | *Annona squamosa* | 0 | 0 | 0 | 0 | 5 | 6 | 7 | 0 | 9 |
| Annonaceae | *Annona sylvatica* | 0 | 0 | 3 | 0 | 0 | 6 | 0 | 0 | 0 |
| Annonaceae | *Annona tomentosa* | 0 | 2 | 0 | 0 | 0 | 0 | 0 | 0 | 0 |
| Annonaceae | *Annona vepretorum* | 0 | 0 | 3 | 0 | 5 | 6 | 7 | 0 | 0 |
| Araceae | *Anthurium erskinei* | 1 | 0 | 0 | 0 | 0 | 0 | 0 | 0 | 0 |
| Araceae | *Anthurium scandens* | 1 | 0 | 3 | 0 | 0 | 0 | 0 | 0 | 0 |
| Araceae | *Anthurium zappiae* | 1 | 0 | 0 | 0 | 0 | 0 | 0 | 0 | 0 |
| Loganiaceae | *Antonia ovata* | 1 | 2 | 0 | 0 | 0 | 0 | 0 | 8 | 9 |
| Loasaceae | *Aosa rupestris* | 0 | 0 | 0 | 0 | 5 | 0 | 0 | 0 | 0 |
| Euphorbiaceae | *Aparisthmium cordatum* | 0 | 2 | 3 | 4 | 0 | 0 | 0 | 0 | 0 |
| Malvaceae | *Apeiba albiflora* | 0 | 2 | 0 | 0 | 0 | 0 | 0 | 0 | 0 |
| Malvaceae | *Apeiba tibourbou* | 0 | 2 | 3 | 0 | 0 | 0 | 0 | 0 | 0 |
| Acanthaceae | *Aphelandra nitida* | 0 | 2 | 0 | 0 | 0 | 0 | 0 | 0 | 0 |
| Anacardiaceae | *Apterokarpos gardneri* | 0 | 0 | 0 | 4 | 0 | 6 | 7 | 8 | 9 |
| Fabaceae | *Apuleia grazielana* | 0 | 0 | 0 | 0 | 0 | 0 | 0 | 8 | 0 |
| Fabaceae | *Apuleia leiocarpa* | 0 | 2 | 3 | 0 | 5 | 6 | 0 | 8 | 0 |
| Fabaceae | *Arachis dardani* | 0 | 0 | 0 | 0 | 0 | 6 | 0 | 0 | 0 |
| Araliaceae | *Aralia excelsa* | 0 | 0 | 3 | 0 | 0 | 6 | 0 | 0 | 0 |
| Araliaceae | *Aralia warmingiana* | 0 | 0 | 0 | 4 | 5 | 6 | 0 | 0 | 0 |
| Aristolochiaceae | *Aristolochia birostris* | 0 | 0 | 0 | 0 | 5 | 0 | 0 | 0 | 0 |
| Cactaceae | *Arrojadoa penicillata* | 0 | 0 | 0 | 0 | 0 | 6 | 0 | 8 | 0 |
| Cactaceae | *Arrojadoa rhodantha* | 0 | 0 | 0 | 0 | 5 | 6 | 0 | 0 | 0 |
| Moraceae | *Artocarpus heterophyllus* | 0 | 0 | 3 | 4 | 0 | 0 | 0 | 0 | 0 |
| Apocynaceae | *Asclepias candida* | 1 | 0 | 0 | 0 | 0 | 0 | 0 | 0 | 0 |
| Polygalaceae | *Asemeia martiana* | 0 | 0 | 0 | 0 | 5 | 0 | 0 | 8 | 0 |
| Polygalaceae | *Asemeia pseudohebeclada* | 0 | 2 | 0 | 0 | 0 | 0 | 7 | 0 | 0 |
| Apocynaceae | *Aspidosperma cuspa* | 0 | 0 | 3 | 0 | 0 | 6 | 7 | 8 | 9 |
| Apocynaceae | *Aspidosperma cylindrocarpon* | 0 | 0 | 3 | 0 | 0 | 6 | 0 | 0 | 0 |
| Apocynaceae | *Aspidosperma discolor* | 1 | 2 | 3 | 0 | 0 | 0 | 0 | 8 | 9 |
| Apocynaceae | *Aspidosperma eburneum* | 0 | 0 | 3 | 0 | 0 | 0 | 0 | 0 | 0 |
| Apocynaceae | *Aspidosperma illustre* | 0 | 0 | 0 | 0 | 5 | 0 | 0 | 0 | 0 |
| Apocynaceae | *Aspidosperma macrocarpon* | 0 | 0 | 3 | 0 | 0 | 0 | 0 | 8 | 0 |
| Apocynaceae | *Aspidosperma multiflorum* | 0 | 0 | 3 | 4 | 5 | 0 | 7 | 8 | 9 |
| Apocynaceae | *Aspidosperma olivaceum* | 0 | 0 | 3 | 0 | 0 | 0 | 0 | 0 | 0 |
| Apocynaceae | *Aspidosperma parvifolium* | 0 | 2 | 3 | 4 | 5 | 0 | 0 | 0 | 0 |
| Apocynaceae | *Aspidosperma polyneuron* | 0 | 0 | 3 | 4 | 0 | 6 | 0 | 0 | 0 |
| Apocynaceae | *Aspidosperma pypifolium* | 0 | 0 | 0 | 0 | 0 | 0 | 0 | 8 | 0 |
| Apocynaceae | *Aspidosperma pyricollum* | 0 | 0 | 3 | 0 | 0 | 0 | 0 | 0 | 0 |
| Apocynaceae | *Aspidosperma pyrifolium* | 0 | 0 | 3 | 4 | 5 | 6 | 7 | 0 | 9 |
| Apocynaceae | *Aspidosperma riedelii* | 0 | 0 | 0 | 0 | 5 | 0 | 7 | 8 | 9 |
| Apocynaceae | *Aspidosperma spruceanum* | 0 | 0 | 3 | 0 | 0 | 0 | 0 | 0 | 0 |
| Apocynaceae | *Aspidosperma subincanum* | 0 | 0 | 3 | 0 | 0 | 0 | 7 | 8 | 9 |
| Apocynaceae | *Aspidosperma tomentosum* | 1 | 0 | 3 | 0 | 0 | 0 | 0 | 0 | 0 |
| Apocynaceae | *Aspidosperma ulei* | 0 | 0 | 0 | 0 | 5 | 6 | 0 | 8 | 0 |
| Asteraceae | *Aspilia almasensis* | 1 | 0 | 0 | 0 | 0 | 0 | 0 | 0 | 0 |
| Asteraceae | *Aspilia foliosa* | 1 | 0 | 0 | 0 | 0 | 0 | 0 | 0 | 0 |
| Euphorbiaceae | *Astraea douradensis* | 1 | 0 | 0 | 0 | 0 | 0 | 0 | 0 | 0 |
| Euphorbiaceae | *Astraea klotzschii* | 0 | 0 | 3 | 0 | 0 | 0 | 0 | 0 | 0 |
| Euphorbiaceae | *Astraea lobata* | 1 | 2 | 0 | 4 | 0 | 0 | 7 | 0 | 0 |
| Arecaceae | *Astrocaryum campestre* | 1 | 0 | 0 | 0 | 0 | 0 | 0 | 0 | 0 |
| Arecaceae | *Astrocaryum vulgare* | 0 | 0 | 0 | 0 | 0 | 0 | 7 | 0 | 0 |
| Phyllanthaceae | *Astrocasia jacobinensis* | 0 | 0 | 0 | 4 | 0 | 0 | 0 | 0 | 0 |
| Anacardiaceae | *Astronium concinnum* | 0 | 0 | 3 | 0 | 5 | 0 | 0 | 0 | 9 |
| Anacardiaceae | *Astronium fraxinifolium* | 1 | 2 | 3 | 0 | 5 | 0 | 0 | 0 | 9 |
| Anacardiaceae | *Astronium graveolens* | 0 | 0 | 0 | 0 | 5 | 0 | 0 | 0 | 0 |
| Fabaceae | *Ateleia guaraya* | 0 | 2 | 0 | 0 | 0 | 0 | 0 | 0 | 0 |
| Fabaceae | *Ateleia ovata* | 0 | 2 | 0 | 0 | 0 | 0 | 0 | 0 | 0 |
| Solanaceae | *Athenaea micrantha* | 1 | 0 | 0 | 0 | 0 | 0 | 0 | 0 | 0 |
| Arecaceae | *Attalea oleifera* | 0 | 0 | 3 | 4 | 0 | 0 | 0 | 0 | 0 |
| Arecaceae | *Attalea speciosa* | 0 | 2 | 0 | 0 | 0 | 0 | 0 | 0 | 9 |
| Solanaceae | *Aureliana fasciculata* | 0 | 0 | 0 | 0 | 5 | 0 | 0 | 0 | 0 |
| Asteraceae | *Austroeupatorium inulifolium* | 0 | 0 | 0 | 4 | 0 | 0 | 0 | 0 | 0 |
| Oxalidaceae | *Averrhoa carambola* | 0 | 0 | 0 | 0 | 0 | 0 | 0 | 0 | 9 |
| Sapindaceae | *Averrhoidium gardnerianum* | 0 | 0 | 3 | 4 | 5 | 0 | 0 | 0 | 0 |
| Verbenaceae | *Avicennia germinans* | 0 | 0 | 0 | 0 | 0 | 0 | 7 | 0 | 0 |
| Asteraceae | *Ayapana amygdalina* | 1 | 0 | 0 | 0 | 0 | 0 | 0 | 0 | 0 |
| Asteraceae | *Ayapanopsis oblongifolia* | 1 | 0 | 0 | 0 | 0 | 0 | 0 | 0 | 0 |
| Sterculiaceae | *Ayenia blanchetiana* | 1 | 0 | 0 | 0 | 0 | 0 | 0 | 0 | 0 |
| Meliaceae | *Azadirachta indica* | 0 | 0 | 0 | 0 | 0 | 6 | 0 | 0 | 9 |
| Asteraceae | *Baccharis aphylla* | 1 | 0 | 0 | 0 | 0 | 0 | 0 | 0 | 0 |
| Asteraceae | *Baccharis cinerea* | 0 | 0 | 0 | 0 | 5 | 0 | 0 | 0 | 0 |
| Asteraceae | *Baccharis linearifolia* | 1 | 0 | 0 | 0 | 0 | 0 | 0 | 0 | 0 |
| Asteraceae | *Baccharis nitida* | 0 | 2 | 0 | 0 | 0 | 0 | 0 | 0 | 0 |
| Asteraceae | *Baccharis oxyodonta* | 0 | 0 | 0 | 4 | 0 | 0 | 0 | 0 | 0 |
| Asteraceae | *Baccharis pingraea* | 1 | 0 | 0 | 0 | 0 | 0 | 0 | 0 | 0 |
| Asteraceae | *Baccharis polygona* | 1 | 0 | 0 | 0 | 0 | 0 | 0 | 0 | 0 |
| Asteraceae | *Baccharis polyphylla* | 1 | 0 | 0 | 0 | 0 | 0 | 0 | 0 | 0 |
| Asteraceae | *Baccharis retusa* | 1 | 0 | 0 | 0 | 0 | 0 | 0 | 0 | 0 |
| Asteraceae | *Baccharis sagittalis* | 1 | 0 | 0 | 0 | 0 | 0 | 0 | 0 | 0 |
| Asteraceae | *Baccharis vulneraria* | 1 | 0 | 3 | 4 | 5 | 0 | 0 | 0 | 0 |
| Asteraceae | *Bahianthus viscosus* | 1 | 0 | 3 | 0 | 0 | 0 | 0 | 0 | 0 |
| Rutaceae | *Balfourodendron molle* | 0 | 0 | 0 | 4 | 5 | 6 | 7 | 0 | 0 |
| Rutaceae | *Balfourodendron riedelianum* | 0 | 0 | 0 | 0 | 0 | 0 | 0 | 8 | 0 |
| Salicaceae | *Banara brasiliensis* | 0 | 2 | 0 | 0 | 0 | 0 | 0 | 0 | 0 |
| Flacourtiaceae | *Banara guianensis* | 0 | 2 | 0 | 0 | 0 | 0 | 0 | 0 | 0 |
| Malpighiaceae | *Banisteriopsis angustifolia* | 1 | 0 | 0 | 0 | 0 | 0 | 0 | 0 | 0 |
| Malpighiaceae | *Banisteriopsis harleyi* | 1 | 0 | 0 | 0 | 0 | 0 | 0 | 0 | 0 |
| Malpighiaceae | *Banisteriopsis malifolia* | 1 | 2 | 0 | 0 | 0 | 0 | 0 | 8 | 0 |
| Malpighiaceae | *Banisteriopsis muricata* | 0 | 0 | 0 | 0 | 0 | 0 | 7 | 0 | 0 |
| Malpighiaceae | *Banisteriopsis nummifera* | 0 | 0 | 0 | 0 | 0 | 0 | 7 | 0 | 0 |
| Malpighiaceae | *Banisteriopsis schizoptera* | 1 | 0 | 0 | 0 | 0 | 0 | 0 | 0 | 0 |
| Malpighiaceae | *Banisteriopsis stellaris* | 1 | 2 | 0 | 0 | 5 | 0 | 0 | 8 | 0 |
| Malpighiaceae | *Banisteriopsis vernoniifolia* | 1 | 0 | 0 | 0 | 0 | 0 | 0 | 0 | 0 |
| Velloziaceae | *Barbacenia blanchetii* | 1 | 0 | 0 | 0 | 0 | 0 | 0 | 0 | 0 |
| Apocynaceae | *Barjonia chloraeifolia* | 1 | 0 | 0 | 0 | 0 | 0 | 0 | 0 | 0 |
| Malpighiaceae | *Barnebya harleyi* | 1 | 0 | 0 | 0 | 5 | 0 | 7 | 0 | 0 |
| Rubiaceae | *Bathysa australis* | 0 | 0 | 0 | 0 | 5 | 0 | 0 | 0 | 0 |
| Fabaceae | *Bauhinia aculeata* | 0 | 0 | 0 | 0 | 5 | 0 | 0 | 0 | 0 |
| Fabaceae | *Bauhinia acuruana* | 1 | 0 | 3 | 4 | 5 | 0 | 7 | 8 | 9 |
| Fabaceae | *Bauhinia brevipes* | 0 | 0 | 3 | 0 | 0 | 0 | 7 | 8 | 0 |
| Fabaceae | *Bauhinia cheilantha* | 0 | 0 | 3 | 4 | 5 | 6 | 7 | 8 | 9 |
| Fabaceae | *Bauhinia cupulata* | 0 | 0 | 0 | 0 | 0 | 0 | 0 | 0 | 9 |
| Fabaceae | *Bauhinia cuyabensis* | 0 | 0 | 0 | 0 | 0 | 0 | 7 | 0 | 0 |
| Fabaceae | *Bauhinia dubia* | 0 | 0 | 0 | 0 | 0 | 0 | 7 | 8 | 9 |
| Fabaceae | *Bauhinia dumosa* | 0 | 0 | 0 | 0 | 5 | 0 | 0 | 0 | 0 |
| Fabaceae | *Bauhinia forficata* | 0 | 2 | 3 | 0 | 5 | 6 | 7 | 0 | 0 |
| Fabaceae | *Bauhinia glabra* | 0 | 0 | 0 | 0 | 0 | 0 | 0 | 8 | 0 |
| Fabaceae | *Bauhinia hirsutiflora* | 0 | 0 | 0 | 0 | 0 | 0 | 0 | 8 | 0 |
| Fabaceae | *Bauhinia holophylla* | 1 | 0 | 0 | 0 | 0 | 0 | 0 | 0 | 0 |
| Fabaceae | *Bauhinia longifolia* | 0 | 0 | 3 | 0 | 0 | 0 | 0 | 0 | 0 |
| Fabaceae | *Bauhinia monandra* | 0 | 0 | 0 | 0 | 0 | 6 | 0 | 0 | 9 |
| Fabaceae | *Bauhinia pentandra* | 0 | 0 | 0 | 0 | 5 | 6 | 7 | 8 | 9 |
| Fabaceae | *Bauhinia pulchella* | 1 | 2 | 0 | 0 | 0 | 0 | 7 | 8 | 9 |
| Fabaceae | *Bauhinia rufa* | 0 | 0 | 3 | 4 | 0 | 0 | 0 | 0 | 0 |
| Fabaceae | *Bauhinia smilacifolia* | 0 | 0 | 0 | 0 | 0 | 0 | 0 | 8 | 0 |
| Fabaceae | *Bauhinia subclavata* | 0 | 2 | 0 | 0 | 5 | 0 | 7 | 8 | 9 |
| Fabaceae | *Bauhinia ungulata* | 0 | 2 | 0 | 0 | 0 | 6 | 7 | 8 | 9 |
| Begoniaceae | *Begonia reniformis* | 0 | 2 | 0 | 0 | 5 | 0 | 0 | 0 | 0 |
| Begoniaceae | *Begonia saxicola* | 0 | 2 | 0 | 0 | 0 | 0 | 0 | 0 | 0 |
| Begoniaceae | *Begonia ulmifolia* | 0 | 0 | 0 | 0 | 5 | 0 | 0 | 0 | 0 |
| Euphorbiaceae | *Bernardia tamanduana* | 0 | 2 | 0 | 4 | 0 | 0 | 0 | 0 | 0 |
| Rubiaceae | *Bertiera guianensis* | 0 | 2 | 0 | 0 | 0 | 0 | 0 | 0 | 0 |
| Euphorbiaceae | *Bia lessertiana* | 0 | 0 | 0 | 4 | 0 | 0 | 0 | 0 | 0 |
| Bignoniaceae | *Bignonia sciuripabulum* | 0 | 0 | 3 | 0 | 0 | 0 | 0 | 0 | 0 |
| Fabaceae | *Bionia coriacea* | 1 | 0 | 3 | 0 | 0 | 0 | 7 | 8 | 0 |
| Fabaceae | *Bionia pedicellata* | 1 | 2 | 0 | 0 | 0 | 0 | 7 | 0 | 0 |
| Asteraceae | *Bishopalea erecta* | 1 | 0 | 0 | 0 | 0 | 0 | 0 | 0 | 0 |
| Bixaceae | *Bixa orellana* | 0 | 0 | 0 | 0 | 0 | 0 | 7 | 0 | 9 |
| Asteraceae | *Blainvillea lanceolata* | 0 | 0 | 0 | 0 | 0 | 6 | 0 | 0 | 0 |
| Asteraceae | *Blanchetia heterotricha* | 0 | 0 | 0 | 4 | 0 | 0 | 0 | 0 | 0 |
| Fabaceae | *Blanchetiodendron blanchetii* | 1 | 0 | 3 | 0 | 0 | 6 | 0 | 0 | 0 |
| Myrtaceae | *Blepharocalyx salicifolius* | 1 | 0 | 3 | 0 | 0 | 0 | 0 | 0 | 0 |
| Apocynaceae | *Blepharodon pictum* | 1 | 0 | 0 | 0 | 0 | 0 | 0 | 0 | 0 |
| Fabaceae | *Bocoa decipiens* | 0 | 0 | 0 | 0 | 0 | 0 | 0 | 8 | 0 |
| Bonnetiaceae | *Bonnetia stricta* | 1 | 2 | 0 | 0 | 0 | 0 | 0 | 0 | 0 |
| Rubiaceae | *Borreria scabiosoides* | 1 | 0 | 0 | 0 | 0 | 0 | 0 | 0 | 0 |
| Rubiaceae | *Borreria verticillata* | 0 | 0 | 0 | 0 | 0 | 0 | 7 | 8 | 0 |
| Nyctaginaceae | *Bougainvillea glabra* | 0 | 0 | 0 | 0 | 0 | 0 | 0 | 0 | 9 |
| Nyctaginaceae | *Bougainvillea praecox* | 0 | 0 | 3 | 0 | 0 | 6 | 0 | 0 | 0 |
| Nyctaginaceae | *Bougainvillea spectabilis* | 0 | 0 | 0 | 4 | 0 | 0 | 0 | 0 | 0 |
| Fabaceae | *Bowdichia virgilioides* | 1 | 2 | 3 | 4 | 5 | 0 | 7 | 0 | 9 |
| Cactaceae | *Brasilicereus phaeacanthus* | 0 | 0 | 0 | 0 | 0 | 6 | 0 | 0 | 0 |
| Cactaceae | *Brasiliopuntia brasiliensis* | 0 | 0 | 0 | 4 | 5 | 6 | 0 | 0 | 0 |
| Polygalaceae | *Bredemeyera autranii* | 0 | 2 | 0 | 0 | 0 | 0 | 0 | 0 | 0 |
| Polygalaceae | *Bredemeyera brevifolia* | 1 | 2 | 0 | 0 | 0 | 0 | 7 | 8 | 9 |
| Polygalaceae | *Bredemeyera floribunda* | 0 | 2 | 0 | 0 | 0 | 0 | 7 | 0 | 0 |
| Polygalaceae | *Bredemeyera hebeclada* | 0 | 2 | 0 | 0 | 0 | 0 | 0 | 0 | 0 |
| Polygalaceae | *Bredemeyera kunthiana* | 0 | 0 | 0 | 0 | 0 | 0 | 0 | 0 | 9 |
| Polygalaceae | *Bredemeyera laurifolia* | 0 | 0 | 0 | 4 | 5 | 0 | 0 | 0 | 0 |
| Polygalaceae | *Bredemeyera velutina* | 0 | 0 | 0 | 0 | 0 | 0 | 0 | 0 | 9 |
| Malvaceae | *Briquetia spicata* | 0 | 0 | 0 | 0 | 0 | 0 | 0 | 8 | 0 |
| Moraceae | *Brosimum gaudichaudii* | 1 | 2 | 3 | 0 | 5 | 0 | 0 | 8 | 9 |
| Moraceae | *Brosimum guianense* | 0 | 2 | 3 | 0 | 0 | 0 | 0 | 0 | 0 |
| Moraceae | *Brosimum rubescens* | 0 | 0 | 0 | 0 | 5 | 0 | 0 | 0 | 0 |
| Solanaceae | *Brunfelsia cuneifolia* | 0 | 0 | 0 | 0 | 0 | 0 | 0 | 8 | 0 |
| Solanaceae | *Brunfelsia uniflora* | 1 | 2 | 0 | 4 | 5 | 0 | 7 | 8 | 0 |
| Combretaceae | *Buchenavia guianensis* | 0 | 0 | 0 | 0 | 0 | 0 | 0 | 0 | 9 |
| Combretaceae | *Buchenavia tetraphylla* | 1 | 2 | 3 | 4 | 5 | 0 | 0 | 8 | 9 |
| Combretaceae | *Buchenavia tomentosa* | 0 | 0 | 3 | 0 | 0 | 0 | 0 | 0 | 0 |
| Malpighiaceae | *Bunchosia acuminata* | 0 | 2 | 0 | 0 | 0 | 0 | 0 | 0 | 9 |
| Malpighiaceae | *Bunchosia apiculata* | 0 | 2 | 0 | 0 | 0 | 0 | 0 | 8 | 0 |
| Malpighiaceae | *Bunchosia maritima* | 0 | 0 | 0 | 0 | 5 | 0 | 0 | 0 | 0 |
| Malpighiaceae | *Bunchosia pernambucana* | 0 | 0 | 0 | 0 | 5 | 0 | 0 | 0 | 0 |
| Arecaceae | *Butia capitata* | 0 | 0 | 3 | 0 | 0 | 0 | 0 | 0 | 0 |
| Malpighiaceae | *Byrsonima bahiana* | 1 | 2 | 0 | 0 | 0 | 0 | 0 | 0 | 0 |
| Malpighiaceae | *Byrsonima blanchetiana* | 0 | 0 | 3 | 0 | 0 | 0 | 0 | 8 | 0 |
| Malpighiaceae | *Byrsonima chrysophylla* | 0 | 0 | 0 | 0 | 0 | 0 | 0 | 0 | 9 |
| Malpighiaceae | *Byrsonima coccolobifolia* | 1 | 2 | 0 | 0 | 5 | 0 | 0 | 0 | 0 |
| Malpighiaceae | *Byrsonima correifolia* | 1 | 0 | 3 | 4 | 0 | 0 | 7 | 8 | 9 |
| Malpighiaceae | *Byrsonima crassifolia* | 0 | 2 | 3 | 0 | 0 | 6 | 7 | 8 | 9 |
| Malpighiaceae | *Byrsonima crispa* | 0 | 2 | 0 | 4 | 0 | 0 | 0 | 0 | 0 |
| Malpighiaceae | *Byrsonima cydoniifolia* | 0 | 0 | 3 | 0 | 0 | 0 | 0 | 0 | 9 |
| Malpighiaceae | *Byrsonima dealbata* | 1 | 2 | 0 | 0 | 5 | 0 | 0 | 0 | 0 |
| Malpighiaceae | *Byrsonima gardneriana* | 1 | 2 | 0 | 4 | 5 | 0 | 7 | 8 | 9 |
| Malpighiaceae | *Byrsonima microphylla* | 0 | 2 | 0 | 0 | 0 | 0 | 0 | 0 | 0 |
| Malpighiaceae | *Byrsonima morii* | 1 | 0 | 0 | 0 | 0 | 0 | 0 | 0 | 0 |
| Malpighiaceae | *Byrsonima nitidifolia* | 0 | 2 | 0 | 4 | 5 | 0 | 0 | 0 | 0 |
| Malpighiaceae | *Byrsonima pachyphylla* | 0 | 0 | 3 | 0 | 0 | 0 | 0 | 0 | 0 |
| Malpighiaceae | *Byrsonima pedunculata* | 0 | 2 | 0 | 0 | 0 | 0 | 0 | 0 | 0 |
| Malpighiaceae | *Byrsonima sericea* | 1 | 2 | 3 | 4 | 5 | 0 | 7 | 8 | 9 |
| Malpighiaceae | *Byrsonima spicata* | 0 | 0 | 0 | 0 | 0 | 0 | 0 | 8 | 0 |
| Malpighiaceae | *Byrsonima stannardii* | 1 | 0 | 0 | 0 | 0 | 0 | 0 | 0 | 0 |
| Malpighiaceae | *Byrsonima stipulacea* | 0 | 0 | 0 | 4 | 0 | 0 | 0 | 0 | 9 |
| Malpighiaceae | *Byrsonima triopterifolia* | 1 | 0 | 0 | 0 | 0 | 0 | 0 | 0 | 0 |
| Malpighiaceae | *Byrsonima vacciniifolia* | 0 | 0 | 0 | 4 | 5 | 0 | 7 | 8 | 9 |
| Malpighiaceae | *Byrsonima variabilis* | 1 | 2 | 0 | 0 | 0 | 0 | 0 | 0 | 0 |
| Malpighiaceae | *Byrsonima verbascifolia* | 1 | 2 | 3 | 0 | 5 | 0 | 7 | 8 | 0 |
| Malvaceae | *Byttneria filipes* | 0 | 0 | 0 | 0 | 5 | 0 | 7 | 0 | 0 |
| Polygalaceae | *Caamembeca insignis* | 1 | 0 | 0 | 0 | 0 | 0 | 0 | 0 | 0 |
| Polygalaceae | *Caamembeca laureola* | 0 | 0 | 0 | 0 | 5 | 0 | 0 | 0 | 0 |
| Polygalaceae | *Caamembeca oxyphylla* | 1 | 0 | 3 | 0 | 0 | 0 | 0 | 0 | 0 |
| Polygalaceae | *Caamembeca spectabilis* | 0 | 2 | 0 | 4 | 5 | 0 | 0 | 8 | 0 |
| Meliaceae | *Cabralea canjerana* | 1 | 0 | 0 | 0 | 0 | 6 | 0 | 0 | 0 |
| Fabaceae | *Caesalpinia calycina* | 1 | 0 | 0 | 0 | 5 | 0 | 0 | 0 | 0 |
| Fabaceae | *Caesalpinia gardneriana* | 0 | 0 | 0 | 0 | 0 | 6 | 7 | 0 | 0 |
| Fabaceae | *Caesalpinia leiostachya* | 0 | 0 | 0 | 0 | 5 | 6 | 0 | 0 | 0 |
| Fabaceae | *Caesalpinia pluviosa* | 0 | 0 | 3 | 0 | 0 | 6 | 0 | 0 | 0 |
| Fabaceae | *Caesalpinia pulcherrima* | 0 | 0 | 0 | 0 | 0 | 0 | 0 | 0 | 9 |
| Fabaceae | *Caesalpinia pyramidalis* | 0 | 0 | 0 | 0 | 5 | 6 | 7 | 0 | 0 |
| Fabaceae | *Cajanus cajan* | 0 | 2 | 0 | 0 | 0 | 0 | 0 | 0 | 0 |
| Fabaceae | *Calliandra aeschynomenoides* | 0 | 0 | 0 | 0 | 5 | 0 | 0 | 0 | 0 |
| Fabaceae | *Calliandra asplenioides* | 1 | 0 | 0 | 0 | 0 | 0 | 0 | 0 | 0 |
| Fabaceae | *Calliandra bahiana* | 1 | 0 | 0 | 0 | 0 | 0 | 0 | 0 | 0 |
| Fabaceae | *Calliandra calycina* | 1 | 0 | 0 | 0 | 0 | 0 | 0 | 0 | 0 |
| Fabaceae | *Calliandra depauperata* | 0 | 0 | 0 | 0 | 5 | 6 | 0 | 0 | 0 |
| Fabaceae | *Calliandra erubescens* | 1 | 0 | 0 | 0 | 0 | 0 | 0 | 0 | 0 |
| Fabaceae | *Calliandra fernandesii* | 0 | 0 | 0 | 0 | 0 | 0 | 0 | 8 | 9 |
| Fabaceae | *Calliandra foliolosa* | 0 | 0 | 3 | 0 | 0 | 0 | 0 | 0 | 0 |
| Fabaceae | *Calliandra fuscipila* | 1 | 0 | 0 | 0 | 0 | 0 | 0 | 0 | 0 |
| Fabaceae | *Calliandra harrisii* | 0 | 0 | 0 | 0 | 0 | 0 | 0 | 8 | 0 |
| Fabaceae | *Calliandra higrophila* | 1 | 0 | 0 | 0 | 0 | 0 | 0 | 0 | 0 |
| Fabaceae | *Calliandra hirsuticaulis* | 1 | 0 | 0 | 0 | 0 | 0 | 0 | 0 | 0 |
| Fabaceae | *Calliandra hirtiflora* | 1 | 0 | 0 | 0 | 0 | 0 | 0 | 0 | 0 |
| Fabaceae | *Calliandra lanata* | 1 | 0 | 0 | 0 | 0 | 0 | 0 | 0 | 0 |
| Fabaceae | *Calliandra leptopoda* | 0 | 0 | 0 | 0 | 0 | 6 | 0 | 0 | 0 |
| Fabaceae | *Calliandra luetzelburgii* | 1 | 0 | 0 | 0 | 0 | 0 | 0 | 0 | 0 |
| Fabaceae | *Calliandra macrocalyx* | 0 | 0 | 0 | 0 | 0 | 6 | 7 | 8 | 0 |
| Fabaceae | *Calliandra mucugeana* | 1 | 0 | 0 | 0 | 0 | 0 | 0 | 0 | 0 |
| Fabaceae | *Calliandra paterna* | 1 | 0 | 0 | 0 | 0 | 0 | 0 | 0 | 0 |
| Fabaceae | *Calliandra renvoizeana* | 1 | 0 | 0 | 0 | 0 | 0 | 0 | 0 | 0 |
| Fabaceae | *Calliandra sessilis* | 1 | 0 | 0 | 0 | 0 | 0 | 0 | 8 | 0 |
| Fabaceae | *Calliandra spinosa* | 0 | 0 | 0 | 0 | 0 | 0 | 7 | 0 | 9 |
| Fabaceae | *Calliandra squarrosa* | 0 | 0 | 0 | 0 | 0 | 6 | 0 | 0 | 0 |
| Fabaceae | *Calliandra umbellifera* | 0 | 2 | 0 | 0 | 0 | 0 | 7 | 0 | 0 |
| Fabaceae | *Calliandra viscidula* | 1 | 0 | 0 | 0 | 0 | 0 | 0 | 0 | 0 |
| Malvaceae | *Callianthe andrade.limae* | 0 | 0 | 0 | 4 | 0 | 0 | 0 | 0 | 0 |
| Malvaceae | *Callianthe scabrida* | 0 | 2 | 0 | 4 | 0 | 0 | 0 | 0 | 0 |
| Vochysiaceae | *Callisthene fasciculata* | 0 | 0 | 3 | 4 | 0 | 6 | 7 | 0 | 9 |
| Vochysiaceae | *Callisthene major* | 0 | 0 | 3 | 0 | 0 | 0 | 0 | 0 | 0 |
| Vochysiaceae | *Callisthene microphylla* | 0 | 0 | 0 | 0 | 5 | 6 | 7 | 0 | 9 |
| Vochysiaceae | *Callisthene minor* | 0 | 0 | 0 | 0 | 0 | 6 | 7 | 8 | 9 |
| Calophyllaceae | *Calophyllum brasiliense* | 1 | 2 | 3 | 0 | 0 | 0 | 0 | 0 | 0 |
| Apocynaceae | *Calotropis procera* | 0 | 2 | 0 | 0 | 5 | 0 | 7 | 8 | 9 |
| Myrtaceae | *Calycolpus legrandii* | 0 | 2 | 0 | 0 | 0 | 0 | 0 | 0 | 0 |
| Myrtaceae | *Calyptranthes brasiliensis* | 1 | 2 | 0 | 0 | 0 | 0 | 0 | 0 | 0 |
| Myrtaceae | *Calyptranthes dardanoi* | 0 | 0 | 0 | 0 | 5 | 0 | 0 | 0 | 0 |
| Myrtaceae | *Calyptranthes grandifolia* | 1 | 0 | 0 | 0 | 0 | 0 | 0 | 0 | 0 |
| Myrtaceae | *Calyptranthes lucida* | 0 | 2 | 0 | 0 | 0 | 0 | 0 | 0 | 0 |
| Myrtaceae | *Calyptranthes multiflora* | 0 | 0 | 0 | 0 | 5 | 0 | 0 | 0 | 0 |
| Myrtaceae | *Calyptranthes pulchella* | 1 | 2 | 0 | 0 | 0 | 0 | 0 | 0 | 0 |
| Myrtaceae | *Calyptranthes rufa* | 1 | 0 | 0 | 0 | 0 | 0 | 0 | 0 | 0 |
| Malpighiaceae | *Camarea elongata* | 1 | 0 | 0 | 0 | 0 | 0 | 0 | 0 | 0 |
| Melastomataceae | *Cambessedesia cambessedesioides* | 1 | 0 | 0 | 0 | 0 | 0 | 0 | 0 | 0 |
| Melastomataceae | *Cambessedesia fasciculata* | 1 | 0 | 0 | 0 | 0 | 0 | 0 | 0 | 0 |
| Melastomataceae | *Cambessedesia hermogenesii* | 1 | 0 | 0 | 0 | 0 | 0 | 0 | 0 | 0 |
| Myrtaceae | *Campomanesia adamantium* | 0 | 2 | 0 | 0 | 0 | 0 | 0 | 0 | 0 |
| Myrtaceae | *Campomanesia aromatica* | 0 | 2 | 3 | 4 | 5 | 0 | 7 | 8 | 9 |
| Myrtaceae | *Campomanesia dichotoma* | 0 | 2 | 0 | 4 | 5 | 0 | 0 | 0 | 0 |
| Myrtaceae | *Campomanesia eugenioides* | 0 | 0 | 3 | 0 | 5 | 6 | 0 | 8 | 0 |
| Myrtaceae | *Campomanesia guaviroba* | 0 | 2 | 0 | 0 | 0 | 0 | 0 | 8 | 0 |
| Myrtaceae | *Campomanesia ilhoensis* | 0 | 0 | 0 | 4 | 5 | 0 | 7 | 0 | 0 |
| Myrtaceae | *Campomanesia lineatifolia* | 0 | 0 | 3 | 0 | 5 | 0 | 0 | 0 | 0 |
| Myrtaceae | *Campomanesia pubescens* | 0 | 0 | 0 | 0 | 0 | 6 | 0 | 0 | 0 |
| Myrtaceae | *Campomanesia sessiliflora* | 1 | 0 | 0 | 0 | 0 | 0 | 0 | 0 | 0 |
| Myrtaceae | *Campomanesia velutina* | 0 | 0 | 0 | 0 | 0 | 0 | 7 | 0 | 9 |
| Fabaceae | *Camptosema coccineum* | 1 | 0 | 0 | 0 | 0 | 0 | 0 | 0 | 0 |
| Fabaceae | *Camptosema pedicellatum* | 1 | 0 | 0 | 0 | 0 | 0 | 0 | 0 | 0 |
| Capparaceae | *Capparidastrum frondosum* | 0 | 0 | 0 | 4 | 0 | 0 | 0 | 0 | 0 |
| Solanaceae | *Capsicum caatingae* | 0 | 0 | 0 | 0 | 5 | 0 | 0 | 0 | 0 |
| Solanaceae | *Capsicum parvifolium* | 0 | 2 | 3 | 4 | 5 | 6 | 7 | 8 | 9 |
| Calophyllaceae | *Caraipa densifolia* | 0 | 0 | 3 | 0 | 0 | 0 | 0 | 0 | 0 |
| Rubiaceae | *Carapichea ipecacuanha* | 0 | 0 | 0 | 0 | 5 | 0 | 0 | 0 | 0 |
| Annonaceae | *Cardiopetalum calophyllum* | 0 | 0 | 0 | 0 | 0 | 0 | 0 | 0 | 9 |
| Sapindaceae | *Cardiospermum anomalum* | 0 | 0 | 0 | 0 | 5 | 0 | 0 | 0 | 9 |
| Sapindaceae | *Cardiospermum corindum* | 0 | 0 | 0 | 0 | 5 | 6 | 7 | 0 | 0 |
| Sapindaceae | *Cardiospermum halicacabum* | 0 | 0 | 0 | 0 | 5 | 0 | 0 | 0 | 0 |
| Sapindaceae | *Cardiospermum strictum* | 0 | 0 | 0 | 0 | 5 | 0 | 0 | 0 | 0 |
| Caricaceae | *Carica papaya* | 0 | 0 | 0 | 0 | 0 | 0 | 7 | 0 | 0 |
| Lecythidaceae | *Cariniana estrellensis* | 0 | 0 | 3 | 0 | 0 | 0 | 0 | 0 | 0 |
| Lecythidaceae | *Cariniana legalis* | 0 | 0 | 3 | 0 | 0 | 0 | 0 | 0 | 0 |
| Caryocaraceae | *Caryocar brasiliense* | 1 | 2 | 3 | 0 | 0 | 0 | 0 | 0 | 0 |
| Caryocaraceae | *Caryocar coriaceum* | 0 | 2 | 0 | 0 | 0 | 0 | 0 | 8 | 9 |
| Apocynaceae | *Cascabela thevetia* | 0 | 0 | 0 | 0 | 0 | 0 | 0 | 0 | 9 |
| Salicaceae | *Casearia aculeata* | 0 | 0 | 0 | 0 | 5 | 0 | 0 | 0 | 0 |
| Salicaceae | *Casearia arborea* | 1 | 2 | 3 | 0 | 0 | 0 | 0 | 0 | 0 |
| Salicaceae | *Casearia bahiensis* | 0 | 2 | 0 | 0 | 0 | 0 | 0 | 0 | 0 |
| Salicaceae | *Casearia commersoniana* | 1 | 2 | 0 | 0 | 0 | 0 | 0 | 0 | 0 |
| Salicaceae | *Casearia decandra* | 1 | 2 | 0 | 0 | 5 | 0 | 0 | 0 | 0 |
| Salicaceae | *Casearia eichleriana* | 1 | 0 | 0 | 0 | 0 | 0 | 0 | 0 | 0 |
| Salicaceae | *Casearia grandiflora* | 0 | 2 | 0 | 0 | 0 | 0 | 7 | 0 | 9 |
| Salicaceae | *Casearia guianensis* | 0 | 0 | 0 | 4 | 0 | 6 | 0 | 8 | 0 |
| Salicaceae | *Casearia hirsuta* | 0 | 0 | 3 | 0 | 0 | 0 | 0 | 0 | 0 |
| Salicaceae | *Casearia javitensis* | 0 | 2 | 3 | 0 | 0 | 0 | 0 | 8 | 0 |
| Salicaceae | *Casearia lasiophylla* | 0 | 0 | 0 | 0 | 0 | 0 | 0 | 8 | 9 |
| Salicaceae | *Casearia mariquitensis* | 0 | 0 | 3 | 4 | 0 | 0 | 0 | 0 | 0 |
| Salicaceae | *Casearia melliodora* | 0 | 0 | 3 | 0 | 0 | 0 | 0 | 0 | 0 |
| Salicaceae | *Casearia obliqua* | 0 | 0 | 0 | 4 | 0 | 0 | 0 | 0 | 0 |
| Salicaceae | *Casearia rupestris* | 0 | 0 | 3 | 0 | 0 | 0 | 0 | 0 | 0 |
| Salicaceae | *Casearia selloana* | 0 | 0 | 3 | 4 | 0 | 0 | 0 | 0 | 0 |
| Salicaceae | *Casearia sylvestris* | 1 | 2 | 3 | 4 | 5 | 0 | 7 | 8 | 9 |
| Salicaceae | *Casearia ulmifolia* | 0 | 0 | 3 | 0 | 0 | 0 | 0 | 0 | 9 |
| Fabaceae | *Cassia bicapsularis* | 0 | 0 | 0 | 0 | 0 | 6 | 0 | 0 | 0 |
| Fabaceae | *Cassia ferruginea* | 0 | 2 | 0 | 4 | 5 | 0 | 0 | 0 | 9 |
| Fabaceae | *Cassia leiandra* | 0 | 2 | 0 | 0 | 0 | 0 | 0 | 0 | 0 |
| Casuarinaceae | *Casuarina equisetifolia* | 0 | 0 | 0 | 0 | 0 | 0 | 0 | 0 | 9 |
| Orchidaceae | *Catasetum micranthum* | 0 | 0 | 0 | 0 | 5 | 0 | 0 | 0 | 0 |
| Malvaceae | *Cavanillesia umbellata* | 0 | 0 | 3 | 4 | 5 | 6 | 0 | 0 | 0 |
| Urticaceae | *Cecropia pachystachya* | 1 | 2 | 3 | 4 | 5 | 0 | 0 | 0 | 0 |
| Urticaceae | *Cecropia palmata* | 0 | 2 | 3 | 0 | 0 | 0 | 0 | 8 | 0 |
| Urticaceae | *Cecropia polystachya* | 0 | 0 | 0 | 0 | 0 | 0 | 0 | 0 | 9 |
| Meliaceae | *Cedrela fissilis* | 0 | 2 | 3 | 4 | 5 | 6 | 0 | 0 | 0 |
| Meliaceae | *Cedrela odorata* | 0 | 0 | 0 | 4 | 5 | 6 | 7 | 0 | 9 |
| Malvaceae | *Ceiba erianthos* | 0 | 0 | 0 | 0 | 5 | 0 | 0 | 0 | 0 |
| Malvaceae | *Ceiba glaziovii* | 0 | 0 | 3 | 4 | 5 | 6 | 7 | 0 | 0 |
| Malvaceae | *Ceiba pentandra* | 0 | 0 | 0 | 0 | 0 | 6 | 0 | 0 | 9 |
| Malvaceae | *Ceiba pubi.ora* | 0 | 0 | 3 | 0 | 0 | 6 | 0 | 0 | 0 |
| Cannabaceae | *Celtis brasiliensis* | 0 | 0 | 3 | 0 | 5 | 6 | 0 | 0 | 0 |
| Cannabaceae | *Celtis fluminensis* | 0 | 0 | 3 | 0 | 0 | 0 | 0 | 0 | 0 |
| Cannabaceae | *Celtis iguanaea* | 0 | 2 | 3 | 4 | 5 | 6 | 0 | 8 | 0 |
| Cannabaceae | *Celtis pubescens* | 0 | 0 | 3 | 0 | 0 | 0 | 0 | 0 | 0 |
| Cannabaceae | *Celtis spinosa* | 0 | 2 | 0 | 0 | 0 | 0 | 0 | 0 | 0 |
| Caesalpiniaceae | *Cenostigma gardnerianum* | 0 | 0 | 0 | 0 | 0 | 0 | 7 | 8 | 9 |
| Caesalpinaceae | *Cenostigma macrophyllum* | 0 | 0 | 0 | 0 | 0 | 0 | 0 | 8 | 9 |
| Asteraceae | *Centratherum punctatum* | 0 | 0 | 0 | 4 | 5 | 0 | 0 | 0 | 0 |
| Fabaceae | *Centrolobium robustum* | 0 | 0 | 0 | 0 | 0 | 6 | 0 | 0 | 0 |
| Fabaceae | *Centrolobium sclerophyllum* | 0 | 0 | 0 | 0 | 0 | 6 | 0 | 0 | 0 |
| Fabaceae | *Centrosema arenarium* | 0 | 0 | 0 | 4 | 0 | 0 | 0 | 0 | 0 |
| Fabaceae | *Centrosema brasilianum* | 0 | 0 | 0 | 0 | 0 | 6 | 0 | 0 | 0 |
| Fabaceae | *Centrosema coriaceum* | 0 | 0 | 0 | 0 | 0 | 0 | 0 | 0 | 9 |
| Fabaceae | *Centrosema plumieri* | 0 | 0 | 0 | 4 | 0 | 0 | 0 | 0 | 0 |
| Ochnaceae | *Cercouratea cassinifolia* | 0 | 0 | 0 | 4 | 0 | 0 | 0 | 0 | 0 |
| Cactaceae | *Cereus albicaulis* | 0 | 0 | 0 | 0 | 0 | 0 | 7 | 8 | 9 |
| Cactaceae | *Cereus jamacaru* | 0 | 0 | 3 | 4 | 5 | 6 | 7 | 8 | 9 |
| Solanaceae | *Cestrum axillare* | 0 | 2 | 3 | 0 | 0 | 0 | 0 | 8 | 0 |
| Solanaceae | *Cestrum laevigatum* | 0 | 0 | 3 | 0 | 5 | 6 | 0 | 0 | 0 |
| Solanaceae | *Cestrum obovatum* | 0 | 0 | 0 | 0 | 5 | 0 | 0 | 0 | 0 |
| Solanaceae | *Cestrum salzmannii* | 0 | 2 | 0 | 0 | 0 | 0 | 0 | 0 | 0 |
| Solanaceae | *Cestrum tenuifolium* | 0 | 2 | 0 | 0 | 0 | 0 | 0 | 0 | 0 |
| Fabaceae | *Chaetocalyx brasiliensis* | 0 | 0 | 0 | 0 | 5 | 0 | 0 | 0 | 0 |
| Fabaceae | *Chaetocalyx longiflora* | 0 | 0 | 0 | 0 | 5 | 0 | 0 | 0 | 0 |
| Fabaceae | *Chaetocalyx scandens* | 0 | 0 | 0 | 4 | 5 | 0 | 0 | 0 | 0 |
| Euphorbiaceae | *Chaetocarpus echinocarpus* | 1 | 2 | 3 | 0 | 5 | 0 | 0 | 0 | 0 |
| Melastomataceae | *Chaetostoma armatum* | 0 | 0 | 0 | 0 | 0 | 0 | 7 | 0 | 0 |
| Melastomataceae | *Chaetostoma luetzelburgii* | 1 | 0 | 0 | 0 | 0 | 0 | 0 | 0 | 0 |
| Fabaceae | *Chamaecrista acosmifolia* | 0 | 0 | 0 | 0 | 0 | 0 | 0 | 8 | 0 |
| Fabaceae | *Chamaecrista amiciella* | 0 | 0 | 0 | 0 | 0 | 0 | 7 | 0 | 0 |
| Fabaceae | *Chamaecrista apoucouita* | 0 | 0 | 3 | 0 | 5 | 0 | 0 | 0 | 0 |
| Fabaceae | *Chamaecrista axilliflora* | 1 | 0 | 0 | 0 | 0 | 0 | 0 | 0 | 0 |
| Fabaceae | *Chamaecrista bahiae* | 0 | 2 | 0 | 0 | 0 | 0 | 0 | 0 | 0 |
| Fabaceae | *Chamaecrista barbata* | 0 | 0 | 0 | 0 | 5 | 0 | 0 | 0 | 0 |
| Fabaceae | *Chamaecrista belemii* | 0 | 0 | 0 | 0 | 5 | 0 | 7 | 8 | 9 |
| Fabaceae | *Chamaecrista blanchetii* | 1 | 2 | 0 | 0 | 0 | 0 | 0 | 0 | 0 |
| Fabaceae | *Chamaecrista brachystachya* | 1 | 0 | 0 | 0 | 0 | 0 | 0 | 0 | 0 |
| Fabaceae | *Chamaecrista brevicalyx* | 0 | 0 | 0 | 0 | 5 | 0 | 0 | 0 | 9 |
| Fabaceae | *Chamaecrista chapadae* | 1 | 0 | 0 | 0 | 0 | 0 | 0 | 0 | 0 |
| Fabaceae | *Chamaecrista confertiformis* | 1 | 0 | 0 | 0 | 0 | 0 | 0 | 0 | 0 |
| Fabaceae | *Chamaecrista cytisoides* | 1 | 2 | 0 | 0 | 0 | 0 | 0 | 0 | 0 |
| Fabaceae | *Chamaecrista desvauxii* | 1 | 2 | 0 | 0 | 5 | 0 | 0 | 8 | 9 |
| Fabaceae | *Chamaecrista diphylla* | 0 | 0 | 0 | 0 | 0 | 0 | 0 | 8 | 0 |
| Fabaceae | *Chamaecrista duckeana* | 0 | 0 | 0 | 0 | 0 | 0 | 7 | 0 | 0 |
| Fabaceae | *Chamaecrista eitenorum* | 0 | 2 | 0 | 0 | 0 | 0 | 7 | 8 | 9 |
| Fabaceae | *Chamaecrista ensiformis* | 1 | 0 | 0 | 4 | 0 | 0 | 0 | 8 | 9 |
| Fabaceae | *Chamaecrista fagonioides* | 0 | 0 | 0 | 0 | 0 | 6 | 0 | 0 | 0 |
| Fabaceae | *Chamaecrista flexuosa* | 0 | 2 | 0 | 4 | 5 | 0 | 0 | 8 | 0 |
| Fabaceae | *Chamaecrista glandulosa* | 0 | 0 | 0 | 0 | 0 | 0 | 7 | 0 | 0 |
| Fabaceae | *Chamaecrista glaucofilix* | 1 | 0 | 0 | 0 | 0 | 0 | 0 | 0 | 0 |
| Fabaceae | *Chamaecrista hispidula* | 0 | 0 | 0 | 0 | 5 | 0 | 7 | 8 | 0 |
| Fabaceae | *Chamaecrista jacobinea* | 1 | 0 | 0 | 0 | 0 | 0 | 0 | 0 | 0 |
| Fabaceae | *Chamaecrista mucronata* | 1 | 0 | 0 | 0 | 0 | 0 | 0 | 0 | 0 |
| Fabaceae | *Chamaecrista nictitans* | 0 | 0 | 3 | 0 | 5 | 6 | 0 | 8 | 9 |
| Fabaceae | *Chamaecrista pascuorum* | 0 | 0 | 0 | 0 | 0 | 6 | 0 | 0 | 0 |
| Fabaceae | *Chamaecrista philippi* | 1 | 0 | 0 | 0 | 0 | 0 | 0 | 0 | 0 |
| Fabaceae | *Chamaecrista ramosa* | 1 | 0 | 0 | 4 | 5 | 0 | 7 | 8 | 0 |
| Fabaceae | *Chamaecrista repens* | 0 | 0 | 0 | 0 | 5 | 0 | 7 | 0 | 9 |
| Fabaceae | *Chamaecrista rotundifolia* | 1 | 2 | 0 | 4 | 0 | 0 | 0 | 0 | 0 |
| Fabaceae | *Chamaecrista tenuisepala* | 0 | 0 | 0 | 0 | 0 | 0 | 0 | 8 | 0 |
| Fabaceae | *Chamaecrista urophyllidia* | 1 | 0 | 0 | 0 | 0 | 0 | 0 | 0 | 0 |
| Fabaceae | *Chamaecrista viscosa* | 0 | 2 | 0 | 0 | 5 | 0 | 0 | 0 | 0 |
| Fabaceae | *Chamaecrista zygophylloides* | 0 | 0 | 0 | 0 | 0 | 0 | 7 | 8 | 0 |
| Celastraceae | *Cheiloclinium cognatum* | 0 | 0 | 3 | 0 | 0 | 0 | 0 | 0 | 0 |
| Celastraceae | *Cheiloclinium serratum* | 0 | 0 | 3 | 0 | 0 | 0 | 0 | 0 | 0 |
| Gentianaceae | *Chelonanthus purpurascens* | 1 | 0 | 0 | 0 | 0 | 0 | 0 | 0 | 0 |
| Amaranthaceae | *Chenopodium hircinum* | 0 | 0 | 0 | 4 | 0 | 0 | 0 | 0 | 0 |
| Rubiaceae | *Chiococca alba* | 1 | 2 | 3 | 4 | 5 | 0 | 0 | 8 | 9 |
| Rubiaceae | *Chiococca nitida* | 0 | 0 | 0 | 0 | 0 | 0 | 0 | 8 | 9 |
| Rubiaceae | *Chiococca plowmanii* | 1 | 0 | 0 | 0 | 0 | 0 | 0 | 0 | 0 |
| Fabaceae | *Chloroleucon acacioides* | 0 | 0 | 0 | 0 | 0 | 0 | 0 | 8 | 9 |
| Fabaceae | *Chloroleucon dumosum* | 0 | 0 | 3 | 4 | 5 | 6 | 7 | 8 | 0 |
| Fabaceae | *Chloroleucon foliolosum* | 0 | 0 | 3 | 0 | 5 | 6 | 7 | 8 | 0 |
| Fabaceae | *Chloroleucon mangense* | 0 | 0 | 0 | 0 | 0 | 6 | 7 | 0 | 0 |
| Fabaceae | *Chloroleucon tortum* | 0 | 0 | 3 | 0 | 0 | 6 | 0 | 0 | 0 |
| Rubiaceae | *Chomelia intercedens* | 0 | 0 | 0 | 0 | 0 | 0 | 0 | 0 | 9 |
| Rubiaceae | *Chomelia martiana* | 0 | 0 | 0 | 0 | 0 | 0 | 0 | 8 | 9 |
| Rubiaceae | *Chomelia obtusa* | 0 | 0 | 3 | 0 | 0 | 0 | 0 | 8 | 9 |
| Rubiaceae | *Chomelia pohliana* | 0 | 2 | 0 | 0 | 0 | 0 | 0 | 0 | 0 |
| Rubiaceae | *Chomelia ribesioides* | 0 | 0 | 0 | 0 | 0 | 0 | 0 | 0 | 9 |
| Rubiaceae | *Chomelia sericea* | 0 | 0 | 3 | 0 | 0 | 0 | 0 | 0 | 0 |
| Asteraceae | *Chresta pinnatifida* | 0 | 0 | 0 | 0 | 0 | 0 | 7 | 0 | 0 |
| Asteraceae | *Chromolaena morii* | 1 | 0 | 0 | 0 | 0 | 0 | 0 | 0 | 0 |
| Asteraceae | *Chromolaena squalida* | 1 | 0 | 0 | 0 | 0 | 0 | 0 | 0 | 0 |
| Chrysobalanaceae | *Chrysobalanus icaco* | 0 | 0 | 0 | 0 | 0 | 0 | 7 | 8 | 0 |
| Sapotaceae | *Chrysophyllum arenarium* | 0 | 2 | 0 | 0 | 0 | 0 | 0 | 8 | 9 |
| Sapotaceae | *Chrysophyllum gonocarpum* | 0 | 0 | 3 | 0 | 0 | 0 | 0 | 0 | 0 |
| Sapotaceae | *Chrysophyllum lucentifolium* | 0 | 2 | 0 | 0 | 0 | 0 | 0 | 0 | 0 |
| Sapotaceae | *Chrysophyllum marginatum* | 0 | 2 | 3 | 0 | 0 | 0 | 7 | 8 | 0 |
| Sapotaceae | *Chrysophyllum rufum* | 1 | 0 | 3 | 4 | 5 | 0 | 0 | 0 | 0 |
| Sapotaceae | *Chrysophyllum sparsiflorum* | 0 | 2 | 0 | 0 | 0 | 0 | 0 | 0 | 0 |
| Sapotaceae | *Chrysophyllum splendens* | 0 | 2 | 0 | 0 | 0 | 0 | 0 | 0 | 0 |
| Lauraceae | *Cinnamomum haussknechtii* | 1 | 0 | 0 | 0 | 0 | 0 | 0 | 0 | 0 |
| Lauraceae | *Cinnamomum tomentulosum* | 1 | 0 | 0 | 0 | 0 | 0 | 0 | 0 | 0 |
| Lauraceae | *Cinnamomum triplinerve* | 1 | 2 | 0 | 4 | 0 | 0 | 0 | 0 | 0 |
| Menispermaceae | *Cissampelos ovalifolia* | 1 | 0 | 0 | 0 | 0 | 0 | 0 | 0 | 0 |
| Vitaceae | *Cissus simsiana* | 0 | 0 | 0 | 0 | 5 | 0 | 0 | 0 | 0 |
| Vitaceae | *Cissus subrhomboidea* | 0 | 2 | 0 | 0 | 0 | 0 | 0 | 0 | 0 |
| Cardiopteridaceae | *Citronella paniculata* | 0 | 0 | 3 | 0 | 0 | 0 | 0 | 0 | 0 |
| Rutaceae | *Citrus aurantium* | 0 | 0 | 0 | 0 | 5 | 6 | 0 | 0 | 0 |
| Rutaceae | *Citrus limon* | 0 | 0 | 0 | 0 | 0 | 6 | 7 | 0 | 9 |
| Melastomataceae | *Clidemia biserrata* | 1 | 2 | 0 | 0 | 0 | 0 | 0 | 0 | 9 |
| Melastomataceae | *Clidemia capitata* | 0 | 0 | 0 | 0 | 0 | 0 | 0 | 0 | 9 |
| Melastomataceae | *Clidemia capitellata* | 0 | 0 | 0 | 4 | 0 | 0 | 0 | 0 | 0 |
| Melastomataceae | *Clidemia debilis* | 0 | 2 | 0 | 4 | 0 | 0 | 0 | 0 | 0 |
| Melastomataceae | *Clidemia dentata* | 0 | 2 | 0 | 0 | 0 | 0 | 0 | 0 | 0 |
| Melastomataceae | *Clidemia hirta* | 1 | 2 | 0 | 4 | 5 | 0 | 0 | 8 | 0 |
| Melastomataceae | *Clidemia japurensis* | 0 | 0 | 3 | 0 | 0 | 0 | 0 | 0 | 0 |
| Melastomataceae | *Clidemia sericea* | 1 | 0 | 0 | 0 | 0 | 0 | 0 | 0 | 0 |
| Melastomataceae | *Clidemia urceolata* | 1 | 2 | 0 | 0 | 0 | 0 | 7 | 0 | 0 |
| Fabaceae | *Clitoria fairchildiana* | 0 | 0 | 0 | 0 | 0 | 6 | 7 | 0 | 9 |
| Fabaceae | *Clitoria laurifolia* | 0 | 0 | 0 | 0 | 0 | 0 | 0 | 8 | 0 |
| Clusiaceae | *Clusia burle.marxii* | 1 | 0 | 0 | 0 | 0 | 0 | 0 | 0 | 0 |
| Clusiaceae | *Clusia criuva* | 1 | 0 | 3 | 0 | 0 | 0 | 0 | 0 | 9 |
| Clusiaceae | *Clusia dardanoi* | 0 | 0 | 0 | 4 | 5 | 0 | 0 | 8 | 0 |
| Clusiaceae | *Clusia intermedia* | 1 | 0 | 0 | 0 | 0 | 0 | 0 | 0 | 0 |
| Clusiaceae | *Clusia melchiorii* | 1 | 0 | 0 | 4 | 0 | 0 | 0 | 0 | 0 |
| Clusiaceae | *Clusia nemorosa* | 1 | 2 | 3 | 4 | 5 | 0 | 0 | 0 | 0 |
| Clusiaceae | *Clusia obdeltifolia* | 1 | 0 | 0 | 0 | 0 | 0 | 0 | 0 | 0 |
| Clusiaceae | *Clusia panapanari* | 0 | 0 | 0 | 0 | 0 | 6 | 0 | 0 | 9 |
| Clusiaceae | *Clusia paralicola* | 0 | 0 | 0 | 0 | 5 | 0 | 0 | 0 | 0 |
| Clusiaceae | *Clusia sellowiana* | 0 | 0 | 0 | 0 | 5 | 0 | 0 | 0 | 0 |
| Euphorbiaceae | *Cnidoscolus adenochlamys* | 0 | 0 | 0 | 0 | 5 | 0 | 0 | 0 | 0 |
| Euphorbiaceae | *Cnidoscolus bahianus* | 0 | 0 | 0 | 0 | 5 | 6 | 0 | 0 | 0 |
| Euphorbiaceae | *Cnidoscolus oligandrus* | 0 | 0 | 3 | 4 | 0 | 6 | 0 | 0 | 0 |
| Euphorbiaceae | *Cnidoscolus pubescens* | 1 | 0 | 3 | 0 | 5 | 6 | 0 | 0 | 0 |
| Euphorbiaceae | *Cnidoscolus quercifolius* | 0 | 0 | 0 | 4 | 5 | 6 | 7 | 8 | 9 |
| Euphorbiaceae | *Cnidoscolus urens* | 0 | 2 | 0 | 4 | 5 | 6 | 7 | 8 | 9 |
| Euphorbiaceae | *Cnidoscolus vitifolius* | 0 | 0 | 0 | 0 | 5 | 0 | 7 | 8 | 9 |
| Polygonaceae | *Coccoloba brasiliensis* | 1 | 0 | 0 | 0 | 0 | 0 | 0 | 0 | 0 |
| Polygonaceae | *Coccoloba declinata* | 0 | 0 | 3 | 4 | 5 | 0 | 0 | 0 | 0 |
| Polygonaceae | *Coccoloba laevis* | 0 | 2 | 0 | 0 | 5 | 0 | 0 | 8 | 0 |
| Polygonaceae | *Coccoloba latifolia* | 0 | 2 | 0 | 0 | 0 | 0 | 0 | 8 | 9 |
| Polygonaceae | *Coccoloba lucidula* | 0 | 2 | 0 | 0 | 0 | 0 | 0 | 0 | 0 |
| Polygonaceae | *Coccoloba mollis* | 0 | 0 | 3 | 4 | 5 | 0 | 0 | 0 | 9 |
| Polygonaceae | *Coccoloba oblonga* | 0 | 2 | 0 | 0 | 0 | 0 | 0 | 0 | 0 |
| Polygonaceae | *Coccoloba parimensis* | 0 | 2 | 0 | 0 | 0 | 0 | 0 | 0 | 0 |
| Polygonaceae | *Coccoloba ramosissima* | 0 | 0 | 0 | 0 | 0 | 0 | 0 | 8 | 0 |
| Polygonaceae | *Coccoloba rosea* | 0 | 2 | 0 | 0 | 5 | 0 | 0 | 0 | 0 |
| Polygonaceae | *Coccoloba schwackeana* | 0 | 0 | 3 | 0 | 5 | 6 | 0 | 0 | 0 |
| Polygonaceae | *Coccoloba tenuiflora* | 0 | 0 | 0 | 0 | 0 | 6 | 0 | 0 | 0 |
| Bixaceae | *Cochlospermum regium* | 0 | 0 | 3 | 0 | 0 | 6 | 0 | 0 | 0 |
| Bixaceae | *Cochlospermum vitifolium* | 0 | 2 | 0 | 0 | 5 | 6 | 7 | 8 | 9 |
| Arecaceae | *Cocos nucifera* | 0 | 0 | 0 | 0 | 0 | 6 | 7 | 0 | 9 |
| Rubiaceae | *Coffea arabica* | 1 | 2 | 3 | 4 | 0 | 0 | 0 | 0 | 0 |
| Cactaceae | *Coleocephalocereus goebelianus* | 0 | 0 | 0 | 0 | 0 | 6 | 0 | 0 | 0 |
| Capparaceae | *Colicodendron yco* | 0 | 0 | 3 | 4 | 5 | 6 | 7 | 0 | 0 |
| Rhamnaceae | *Colubrina cordifolia* | 1 | 2 | 0 | 4 | 0 | 0 | 0 | 8 | 9 |
| Rhamnaceae | *Colubrina glandulosa* | 0 | 0 | 0 | 0 | 0 | 0 | 7 | 0 | 0 |
| Combretaceae | *Combretum discolor* | 0 | 0 | 0 | 0 | 0 | 0 | 0 | 0 | 9 |
| Combretaceae | *Combretum duarteanum* | 0 | 2 | 3 | 0 | 0 | 6 | 0 | 0 | 9 |
| Combretaceae | *Combretum fruticosum* | 0 | 0 | 0 | 0 | 5 | 0 | 7 | 0 | 0 |
| Combretaceae | *Combretum glaucocarpum* | 0 | 2 | 0 | 0 | 0 | 6 | 7 | 8 | 9 |
| Combretaceae | *Combretum hilarianum* | 0 | 0 | 0 | 0 | 5 | 6 | 7 | 8 | 0 |
| Combretaceae | *Combretum lanceolatum* | 0 | 2 | 0 | 0 | 0 | 0 | 7 | 8 | 9 |
| Combretaceae | *Combretum laxum* | 0 | 2 | 3 | 0 | 0 | 0 | 7 | 0 | 9 |
| Combretaceae | *Combretum leprosum* | 0 | 2 | 3 | 0 | 5 | 6 | 7 | 8 | 9 |
| Combretaceae | *Combretum mellifluum* | 1 | 2 | 0 | 0 | 0 | 0 | 7 | 8 | 9 |
| Combretaceae | *Combretum monetaria* | 1 | 0 | 0 | 0 | 5 | 6 | 7 | 0 | 0 |
| Combretaceae | *Combretum pisonioides* | 0 | 0 | 0 | 0 | 5 | 6 | 0 | 0 | 9 |
| Commelinaceae | *Commelina erecta* | 0 | 0 | 0 | 0 | 5 | 0 | 0 | 0 | 0 |
| Burseraceae | *Commiphora leptophloeos* | 0 | 0 | 3 | 0 | 5 | 6 | 7 | 8 | 9 |
| Melastomataceae | *Comolia villosa* | 0 | 0 | 0 | 0 | 0 | 0 | 0 | 8 | 0 |
| Rutaceae | *Conchocarpus macrophyllus* | 0 | 0 | 3 | 0 | 0 | 0 | 0 | 0 | 0 |
| Connaraceae | *Connarus detersus* | 0 | 2 | 0 | 0 | 0 | 0 | 0 | 8 | 0 |
| Connaraceae | *Connarus rostratus* | 0 | 0 | 3 | 0 | 0 | 0 | 0 | 0 | 0 |
| Connaraceae | *Connarus suberosus* | 0 | 0 | 3 | 0 | 0 | 0 | 0 | 0 | 0 |
| Combretaceae | *Conocarpus erectus* | 0 | 0 | 0 | 0 | 0 | 0 | 7 | 8 | 0 |
| Asteraceae | *Conocliniopsis prasiifolia* | 1 | 0 | 0 | 4 | 5 | 0 | 7 | 8 | 0 |
| Rubiaceae | *Conyza bonariensis* | 0 | 0 | 0 | 4 | 0 | 0 | 0 | 0 | 0 |
| Rubiaceae | *Conyza sumatrensis* | 1 | 0 | 0 | 0 | 0 | 0 | 0 | 0 | 0 |
| Fabaceae | *Copaifera arenicola* | 0 | 0 | 0 | 0 | 5 | 0 | 7 | 8 | 0 |
| Fabaceae | *Copaifera cearensis* | 0 | 0 | 0 | 0 | 0 | 0 | 7 | 0 | 0 |
| Fabaceae | *Copaifera coriacea* | 0 | 0 | 3 | 0 | 0 | 0 | 7 | 8 | 9 |
| Fabaceae | *Copaifera duckei* | 0 | 2 | 0 | 0 | 5 | 0 | 0 | 0 | 0 |
| Fabaceae | *Copaifera elliptica* | 0 | 0 | 0 | 0 | 0 | 0 | 0 | 0 | 9 |
| Fabaceae | *Copaifera langsdorffii* | 1 | 2 | 3 | 4 | 5 | 6 | 7 | 8 | 9 |
| Fabaceae | *Copaifera lucens* | 0 | 0 | 3 | 0 | 0 | 0 | 0 | 0 | 0 |
| Fabaceae | *Copaifera luetzelburgii* | 0 | 0 | 0 | 0 | 0 | 0 | 7 | 8 | 9 |
| Fabaceae | *Copaifera martii* | 0 | 0 | 3 | 0 | 5 | 0 | 7 | 8 | 9 |
| Fabaceae | *Copaifera multijuga* | 0 | 0 | 3 | 0 | 0 | 0 | 0 | 0 | 0 |
| Fabaceae | *Copaifera oblongifolia* | 0 | 0 | 0 | 0 | 0 | 0 | 0 | 8 | 9 |
| Fabaceae | *Copaifera trapezifolia* | 0 | 2 | 0 | 0 | 0 | 0 | 0 | 0 | 0 |
| Arecaceae | *Copernicia prunifera* | 0 | 2 | 0 | 0 | 5 | 6 | 7 | 8 | 9 |
| Malvaceae | *Corchorus aestuans* | 0 | 2 | 0 | 0 | 0 | 0 | 0 | 0 | 0 |
| Malvaceae | *Corchorus hirtus* | 0 | 0 | 0 | 4 | 5 | 0 | 0 | 0 | 0 |
| Boraginaceae | *Cordia alliodora* | 0 | 0 | 3 | 0 | 5 | 6 | 0 | 0 | 0 |
| Boraginaceae | *Cordia bicolor* | 1 | 2 | 0 | 0 | 0 | 0 | 0 | 8 | 0 |
| Boraginaceae | *Cordia blanchetii* | 0 | 0 | 3 | 0 | 0 | 0 | 0 | 0 | 0 |
| Boraginaceae | *Cordia bullata* | 0 | 0 | 0 | 0 | 5 | 6 | 7 | 8 | 0 |
| Boraginaceae | *Cordia curassavica* | 0 | 2 | 0 | 0 | 5 | 6 | 0 | 0 | 0 |
| Boraginaceae | *Cordia dardanoi* | 0 | 0 | 0 | 0 | 0 | 6 | 0 | 0 | 0 |
| Boraginaceae | *Cordia glabrata* | 0 | 0 | 3 | 0 | 5 | 6 | 7 | 0 | 0 |
| Boraginaceae | *Cordia glazioviana* | 0 | 0 | 0 | 0 | 0 | 6 | 7 | 8 | 0 |
| Boraginaceae | *Cordia harleyi* | 1 | 0 | 0 | 0 | 0 | 0 | 0 | 0 | 0 |
| Boraginaceae | *Cordia incognita* | 0 | 0 | 0 | 0 | 0 | 0 | 7 | 0 | 0 |
| Boraginaceae | *Cordia insignis* | 0 | 0 | 0 | 0 | 5 | 6 | 7 | 8 | 0 |
| Boraginaceae | *Cordia latiloba* | 0 | 0 | 0 | 0 | 5 | 0 | 0 | 0 | 0 |
| Boraginaceae | *Cordia leucocephala* | 0 | 2 | 0 | 0 | 5 | 6 | 7 | 0 | 0 |
| Boraginaceae | *Cordia leucomalloides* | 0 | 0 | 0 | 0 | 0 | 0 | 0 | 8 | 0 |
| Boraginaceae | *Cordia multispicata* | 0 | 2 | 0 | 4 | 5 | 6 | 0 | 0 | 0 |
| Boraginaceae | *Cordia nivea* | 0 | 0 | 0 | 0 | 0 | 0 | 0 | 0 | 9 |
| Boraginaceae | *Cordia oncocalyx* | 0 | 0 | 3 | 0 | 5 | 6 | 7 | 8 | 9 |
| Boraginaceae | *Cordia pilosa* | 0 | 0 | 0 | 0 | 5 | 0 | 0 | 0 | 0 |
| Boraginaceae | *Cordia polycephala* | 0 | 2 | 0 | 4 | 0 | 0 | 0 | 0 | 9 |
| Boraginaceae | *Cordia rufescens* | 0 | 2 | 0 | 0 | 5 | 0 | 7 | 8 | 9 |
| Boraginaceae | *Cordia sellowiana* | 0 | 2 | 0 | 4 | 0 | 0 | 0 | 0 | 0 |
| Boraginaceae | *Cordia superba* | 0 | 0 | 3 | 4 | 0 | 0 | 0 | 8 | 0 |
| Boraginaceae | *Cordia taguahyensis* | 0 | 2 | 3 | 0 | 0 | 0 | 0 | 0 | 0 |
| Boraginaceae | *Cordia toqueve* | 0 | 2 | 0 | 0 | 0 | 0 | 0 | 0 | 0 |
| Boraginaceae | *Cordia trichotoma* | 0 | 2 | 3 | 4 | 5 | 6 | 7 | 8 | 9 |
| Rubiaceae | *Cordiera concolor* | 1 | 2 | 3 | 0 | 0 | 0 | 0 | 8 | 0 |
| Rubiaceae | *Cordiera elliptica* | 1 | 0 | 3 | 0 | 0 | 0 | 0 | 0 | 0 |
| Rubiaceae | *Cordiera humilis* | 0 | 2 | 0 | 0 | 0 | 0 | 0 | 8 | 0 |
| Rubiaceae | *Cordiera myrciifolia* | 0 | 2 | 0 | 4 | 0 | 0 | 0 | 0 | 9 |
| Rubiaceae | *Cordiera obtusa* | 0 | 0 | 3 | 0 | 0 | 0 | 0 | 0 | 0 |
| Rubiaceae | *Cordiera rigida* | 0 | 0 | 3 | 0 | 5 | 6 | 0 | 8 | 9 |
| Rubiaceae | *Cordiera sessilis* | 1 | 2 | 0 | 0 | 0 | 0 | 0 | 8 | 9 |
| Costaceae | *Costus spiralis* | 0 | 0 | 0 | 4 | 0 | 0 | 0 | 0 | 0 |
| Chrysobalanaceae | *Couepia impressa* | 0 | 0 | 3 | 0 | 0 | 0 | 0 | 0 | 0 |
| Chrysobalanaceae | *Couepia monteclarensis* | 0 | 0 | 3 | 0 | 0 | 0 | 0 | 0 | 0 |
| Chrysobalanaceae | *Couepia ovalifolia* | 0 | 2 | 0 | 0 | 0 | 0 | 0 | 0 | 0 |
| Apocynaceae | *Couma rigida* | 1 | 2 | 0 | 0 | 0 | 0 | 0 | 0 | 0 |
| Lecythidaceae | *Couroupita guianensis* | 0 | 0 | 0 | 0 | 0 | 0 | 0 | 0 | 9 |
| Fabaceae | *Coursetia rostrata* | 0 | 0 | 0 | 0 | 0 | 6 | 7 | 8 | 0 |
| Urticaceae | *Coussapoa microcephala* | 0 | 2 | 0 | 0 | 0 | 0 | 0 | 0 | 0 |
| Rubiaceae | *Coussarea capitata* | 0 | 0 | 3 | 0 | 0 | 0 | 0 | 0 | 0 |
| Rubiaceae | *Coussarea contracta* | 0 | 0 | 0 | 4 | 0 | 0 | 0 | 0 | 0 |
| Rubiaceae | *Coussarea hydrangeifolia* | 0 | 2 | 3 | 0 | 0 | 0 | 0 | 0 | 0 |
| Rubiaceae | *Coussarea leptopus* | 0 | 0 | 3 | 0 | 0 | 0 | 0 | 0 | 0 |
| Rubiaceae | *Coutarea hexandra* | 0 | 2 | 3 | 4 | 5 | 6 | 7 | 8 | 0 |
| Fabaceae | *Cranocarpus gracilis* | 0 | 0 | 0 | 0 | 0 | 0 | 7 | 0 | 9 |
| Capparaceae | *Crateva tapia* | 0 | 0 | 0 | 0 | 5 | 6 | 7 | 8 | 0 |
| Fabaceae | *Cratylia argentea* | 0 | 0 | 0 | 0 | 0 | 0 | 0 | 0 | 9 |
| Fabaceae | *Cratylia mollis* | 0 | 0 | 0 | 0 | 5 | 0 | 7 | 8 | 0 |
| Fabaceae | *Crotalaria bahiensis* | 0 | 0 | 0 | 0 | 5 | 0 | 0 | 0 | 0 |
| Fabaceae | *Crotalaria holosericea* | 0 | 2 | 0 | 4 | 5 | 6 | 7 | 0 | 0 |
| Fabaceae | *Crotalaria incana* | 0 | 0 | 0 | 0 | 0 | 0 | 0 | 8 | 0 |
| Fabaceae | *Crotalaria laeta* | 1 | 0 | 0 | 0 | 0 | 0 | 0 | 0 | 0 |
| Fabaceae | *Crotalaria maypurensis* | 0 | 2 | 0 | 0 | 0 | 0 | 0 | 0 | 0 |
| Fabaceae | *Crotalaria micans* | 1 | 0 | 0 | 0 | 0 | 0 | 0 | 0 | 0 |
| Fabaceae | *Crotalaria pallida* | 0 | 0 | 0 | 4 | 0 | 0 | 0 | 8 | 0 |
| Fabaceae | *Crotalaria pilosa* | 0 | 0 | 0 | 0 | 0 | 6 | 0 | 0 | 0 |
| Fabaceae | *Crotalaria retusa* | 0 | 0 | 0 | 0 | 0 | 6 | 0 | 8 | 9 |
| Fabaceae | *Crotalaria vitellina* | 0 | 2 | 0 | 4 | 5 | 0 | 7 | 0 | 0 |
| Euphorbiaceae | *Croton adamantinus* | 1 | 2 | 0 | 0 | 5 | 0 | 7 | 8 | 0 |
| Euphorbiaceae | *Croton adenocalyx* | 0 | 2 | 0 | 0 | 5 | 6 | 7 | 0 | 0 |
| Euphorbiaceae | *Croton adenodontus* | 0 | 0 | 0 | 0 | 5 | 0 | 0 | 8 | 0 |
| Euphorbiaceae | *Croton anisodontus* | 0 | 0 | 0 | 0 | 0 | 0 | 7 | 8 | 0 |
| Euphorbiaceae | *Croton argyroglossus* | 0 | 0 | 0 | 0 | 5 | 0 | 0 | 8 | 0 |
| Euphorbiaceae | *Croton argyrophylloides* | 0 | 2 | 3 | 0 | 5 | 6 | 7 | 8 | 9 |
| Euphorbiaceae | *Croton argyrophyllus* | 1 | 0 | 3 | 4 | 5 | 0 | 0 | 0 | 0 |
| Euphorbiaceae | *Croton betaceus* | 1 | 2 | 0 | 0 | 5 | 0 | 7 | 8 | 9 |
| Euphorbiaceae | *Croton betulaster* | 1 | 0 | 0 | 0 | 0 | 0 | 0 | 0 | 0 |
| Euphorbiaceae | *Croton blanchetianus* | 1 | 2 | 0 | 4 | 5 | 6 | 7 | 8 | 9 |
| Euphorbiaceae | *Croton campestris* | 1 | 0 | 0 | 4 | 5 | 6 | 7 | 0 | 9 |
| Euphorbiaceae | *Croton celtidifolius* | 0 | 0 | 0 | 0 | 0 | 0 | 7 | 0 | 0 |
| Euphorbiaceae | *Croton conduplicatus* | 0 | 0 | 0 | 0 | 5 | 6 | 0 | 0 | 0 |
| Euphorbiaceae | *Croton cordiifolius* | 1 | 0 | 3 | 0 | 0 | 0 | 0 | 0 | 0 |
| Euphorbiaceae | *Croton desertorum* | 1 | 0 | 0 | 0 | 0 | 0 | 0 | 0 | 0 |
| Euphorbiaceae | *Croton echioides* | 1 | 0 | 0 | 0 | 5 | 6 | 7 | 8 | 9 |
| Euphorbiaceae | *Croton erythroxyloides* | 1 | 0 | 0 | 0 | 0 | 0 | 0 | 0 | 0 |
| Euphorbiaceae | *Croton floribundus* | 0 | 2 | 3 | 4 | 0 | 0 | 7 | 0 | 0 |
| Euphorbiaceae | *Croton gardnerianus* | 0 | 0 | 0 | 0 | 0 | 0 | 0 | 0 | 9 |
| Euphorbiaceae | *Croton glandulosobracteatus* | 1 | 0 | 0 | 0 | 0 | 0 | 0 | 0 | 0 |
| Euphorbiaceae | *Croton glandulosus* | 0 | 0 | 0 | 4 | 5 | 0 | 7 | 8 | 0 |
| Euphorbiaceae | *Croton glutinosus* | 1 | 0 | 0 | 0 | 5 | 0 | 0 | 0 | 0 |
| Euphorbiaceae | *Croton goyazensis* | 0 | 0 | 0 | 0 | 0 | 0 | 0 | 0 | 9 |
| Euphorbiaceae | *Croton grewioides* | 0 | 0 | 0 | 0 | 5 | 0 | 7 | 8 | 9 |
| Euphorbiaceae | *Croton harleyi* | 0 | 0 | 0 | 0 | 0 | 6 | 0 | 8 | 9 |
| Euphorbiaceae | *Croton heliotropiifolius* | 1 | 2 | 0 | 4 | 5 | 6 | 7 | 8 | 9 |
| Euphorbiaceae | *Croton hemiargyreus* | 0 | 0 | 0 | 0 | 5 | 6 | 0 | 0 | 0 |
| Euphorbiaceae | *Croton hirtus* | 0 | 0 | 0 | 0 | 0 | 0 | 7 | 0 | 9 |
| Euphorbiaceae | *Croton imbricatus* | 0 | 0 | 0 | 0 | 0 | 0 | 7 | 0 | 0 |
| Euphorbiaceae | *Croton jacobinensis* | 0 | 2 | 0 | 0 | 0 | 6 | 0 | 8 | 9 |
| Euphorbiaceae | *Croton japirensis* | 0 | 0 | 0 | 0 | 0 | 6 | 0 | 8 | 0 |
| Euphorbiaceae | *Croton laceratoglandulosus* | 0 | 0 | 0 | 0 | 0 | 0 | 7 | 0 | 0 |
| Euphorbiaceae | *Croton limae* | 1 | 2 | 3 | 0 | 5 | 0 | 7 | 0 | 0 |
| Euphorbiaceae | *Croton linearifolius* | 0 | 0 | 0 | 0 | 0 | 6 | 0 | 0 | 0 |
| Euphorbiaceae | *Croton longibracteatus* | 1 | 0 | 0 | 0 | 0 | 0 | 0 | 0 | 0 |
| Euphorbiaceae | *Croton luetzelburgii* | 1 | 0 | 0 | 0 | 0 | 0 | 0 | 0 | 0 |
| Euphorbiaceae | *Croton lundianus* | 0 | 0 | 0 | 0 | 0 | 0 | 7 | 0 | 0 |
| Euphorbiaceae | *Croton micans* | 0 | 0 | 0 | 0 | 5 | 0 | 0 | 0 | 0 |
| Euphorbiaceae | *Croton mucronifolius* | 0 | 0 | 3 | 0 | 0 | 0 | 0 | 0 | 0 |
| Euphorbiaceae | *Croton muscicarpa* | 1 | 0 | 0 | 0 | 0 | 0 | 0 | 0 | 0 |
| Euphorbiaceae | *Croton myrsinites* | 1 | 0 | 0 | 0 | 0 | 0 | 0 | 0 | 0 |
| Euphorbiaceae | *Croton nepetifolius* | 0 | 2 | 0 | 0 | 5 | 0 | 7 | 8 | 9 |
| Euphorbiaceae | *Croton nummularius* | 1 | 0 | 0 | 0 | 0 | 0 | 0 | 0 | 0 |
| Euphorbiaceae | *Croton odontadenius* | 0 | 0 | 0 | 0 | 0 | 0 | 0 | 0 | 9 |
| Euphorbiaceae | *Croton pedicellatus* | 0 | 0 | 3 | 0 | 5 | 0 | 7 | 8 | 9 |
| Euphorbiaceae | *Croton piauhiensis* | 0 | 0 | 0 | 0 | 0 | 0 | 7 | 0 | 0 |
| Euphorbiaceae | *Croton pulegiodorus* | 0 | 0 | 0 | 0 | 0 | 6 | 0 | 8 | 0 |
| Euphorbiaceae | *Croton pulegioides* | 0 | 0 | 0 | 0 | 5 | 0 | 0 | 8 | 0 |
| Euphorbiaceae | *Croton rudolphianus* | 1 | 0 | 0 | 4 | 5 | 6 | 0 | 0 | 9 |
| Euphorbiaceae | *Croton schultesii* | 1 | 0 | 0 | 0 | 0 | 0 | 0 | 0 | 0 |
| Euphorbiaceae | *Croton sellowii* | 0 | 2 | 0 | 0 | 5 | 0 | 0 | 0 | 0 |
| Euphorbiaceae | *Croton sincorensis* | 1 | 0 | 0 | 0 | 0 | 6 | 0 | 0 | 0 |
| Euphorbiaceae | *Croton sonderianus* | 0 | 2 | 0 | 0 | 5 | 6 | 7 | 8 | 0 |
| Euphorbiaceae | *Croton tetradenius* | 0 | 0 | 3 | 0 | 5 | 0 | 0 | 0 | 0 |
| Euphorbiaceae | *Croton triangularis* | 0 | 2 | 0 | 0 | 0 | 0 | 0 | 0 | 0 |
| Euphorbiaceae | *Croton tricolor* | 0 | 2 | 0 | 0 | 5 | 0 | 0 | 8 | 0 |
| Euphorbiaceae | *Croton triqueter* | 1 | 2 | 3 | 4 | 0 | 6 | 0 | 0 | 0 |
| Euphorbiaceae | *Croton urticifolius* | 0 | 0 | 3 | 4 | 5 | 6 | 0 | 8 | 9 |
| Euphorbiaceae | *Croton urucurana* | 0 | 2 | 3 | 0 | 0 | 0 | 0 | 0 | 0 |
| Euphorbiaceae | *Croton velutinus* | 1 | 0 | 3 | 0 | 0 | 0 | 0 | 0 | 0 |
| Euphorbiaceae | *Croton virgultosus* | 0 | 0 | 0 | 0 | 5 | 0 | 7 | 0 | 0 |
| Euphorbiaceae | *Croton zehntneri* | 0 | 0 | 0 | 0 | 0 | 6 | 7 | 8 | 9 |
| Apocynaceae | *Cryptostegia grandiflora* | 0 | 0 | 0 | 0 | 0 | 0 | 0 | 8 | 0 |
| Apocynaceae | *Cryptostegia madagascariensis* | 0 | 0 | 0 | 0 | 0 | 0 | 0 | 8 | 0 |
| Sapindaceae | *Cupania impressinervia* | 1 | 2 | 3 | 4 | 5 | 0 | 0 | 0 | 0 |
| Sapindaceae | *Cupania oblongifolia* | 0 | 2 | 3 | 4 | 0 | 0 | 0 | 0 | 9 |
| Sapindaceae | *Cupania paniculata* | 1 | 0 | 0 | 0 | 0 | 0 | 0 | 0 | 0 |
| Sapindaceae | *Cupania racemosa* | 0 | 0 | 0 | 0 | 0 | 0 | 0 | 0 | 9 |
| Sapindaceae | *Cupania rigida* | 1 | 0 | 0 | 0 | 0 | 0 | 0 | 0 | 0 |
| Sapindaceae | *Cupania vernalis* | 0 | 0 | 3 | 0 | 0 | 0 | 0 | 0 | 0 |
| Lythraceae | *Cuphea campestris* | 0 | 0 | 0 | 0 | 0 | 0 | 0 | 8 | 0 |
| Lythraceae | *Cuphea ericoides* | 1 | 0 | 0 | 0 | 0 | 0 | 0 | 0 | 0 |
| Lythraceae | *Cuphea flava* | 0 | 0 | 0 | 0 | 0 | 0 | 0 | 8 | 0 |
| Lythraceae | *Cuphea glareosa* | 1 | 0 | 0 | 0 | 0 | 0 | 0 | 0 | 0 |
| Lythraceae | *Cuphea pulchra* | 0 | 0 | 3 | 0 | 0 | 0 | 7 | 0 | 0 |
| Lythraceae | *Cuphea sessilifolia* | 1 | 0 | 0 | 0 | 0 | 0 | 0 | 0 | 0 |
| Dilleniaceae | *Curatella americana* | 0 | 2 | 3 | 0 | 0 | 0 | 7 | 8 | 9 |
| Bignoniaceae | *Cuspidaria argentea* | 0 | 0 | 0 | 0 | 0 | 0 | 7 | 0 | 0 |
| Bignoniaceae | *Cuspidaria cratensis* | 0 | 0 | 0 | 0 | 0 | 0 | 7 | 0 | 0 |
| Bignoniaceae | *Cuspidaria lateriflora* | 0 | 0 | 0 | 0 | 0 | 0 | 7 | 0 | 0 |
| Bignoniaceae | *Cuspidaria pulchra* | 0 | 0 | 0 | 0 | 0 | 0 | 0 | 8 | 0 |
| Lamiaceae | *Cyanocephalus rugosus* | 1 | 0 | 0 | 0 | 0 | 0 | 0 | 0 | 0 |
| Cyatheaceae | *Cyathea delgadii* | 1 | 0 | 0 | 0 | 0 | 0 | 0 | 0 | 0 |
| Cyatheaceae | *Cyathea villosa* | 1 | 0 | 0 | 0 | 0 | 0 | 0 | 0 | 0 |
| Primulaceae | *Cybianthus brasiliensis* | 1 | 0 | 0 | 0 | 0 | 0 | 0 | 0 | 0 |
| Primulaceae | *Cybianthus detergens* | 0 | 2 | 0 | 0 | 0 | 0 | 0 | 8 | 0 |
| Primulaceae | *Cybianthus glaber* | 1 | 0 | 0 | 0 | 0 | 0 | 0 | 0 | 0 |
| Primulaceae | *Cybianthus penduliflorus* | 1 | 0 | 0 | 0 | 0 | 0 | 0 | 0 | 0 |
| Primulaceae | *Cybianthus peruvianus* | 1 | 0 | 0 | 0 | 0 | 0 | 0 | 0 | 0 |
| Bignoniaceae | *Cybistax antisyphilitica* | 0 | 0 | 3 | 0 | 0 | 0 | 0 | 0 | 9 |
| Apocynaceae | *Cynanchum myrtifolium* | 1 | 0 | 0 | 0 | 0 | 0 | 0 | 0 | 0 |
| Capparaceae | *Cynophalla flexuosa* | 0 | 0 | 3 | 4 | 5 | 6 | 7 | 8 | 9 |
| Capparaceae | *Cynophalla hastata* | 0 | 2 | 0 | 0 | 5 | 6 | 7 | 8 | 9 |
| Anacardiaceae | *Cyrtocarpa caatingae* | 0 | 0 | 3 | 0 | 5 | 0 | 0 | 0 | 0 |
| Asteraceae | *Cyrtocymura harleyi* | 1 | 0 | 0 | 0 | 5 | 0 | 0 | 0 | 0 |
| Asteraceae | *Cyrtocymura scorpioides* | 0 | 2 | 0 | 4 | 5 | 0 | 0 | 0 | 0 |
| Cleomaceae | *Dactylaena microphylla* | 1 | 0 | 0 | 0 | 0 | 0 | 0 | 0 | 0 |
| Fabaceae | *Dahlstedtia araripensis* | 0 | 2 | 0 | 0 | 5 | 0 | 7 | 8 | 9 |
| Fabaceae | *Dalbergia catingicola* | 0 | 0 | 0 | 0 | 5 | 0 | 0 | 8 | 0 |
| Fabaceae | *Dalbergia cearensis* | 0 | 0 | 3 | 0 | 5 | 6 | 7 | 8 | 9 |
| Fabaceae | *Dalbergia decipularis* | 0 | 0 | 3 | 0 | 0 | 0 | 0 | 8 | 0 |
| Fabaceae | *Dalbergia ecastaphyllum* | 0 | 0 | 0 | 0 | 0 | 0 | 0 | 8 | 0 |
| Fabaceae | *Dalbergia frutescens* | 0 | 0 | 3 | 0 | 0 | 0 | 7 | 8 | 9 |
| Fabaceae | *Dalbergia glaucescens* | 0 | 0 | 0 | 0 | 0 | 0 | 0 | 0 | 9 |
| Fabaceae | *Dalbergia miscolobium* | 1 | 2 | 0 | 0 | 0 | 0 | 0 | 0 | 0 |
| Fabaceae | *Dalbergia nigra* | 0 | 0 | 3 | 0 | 0 | 0 | 0 | 0 | 0 |
| Euphorbiaceae | *Dalechampia pentaphylla* | 0 | 0 | 0 | 4 | 0 | 0 | 0 | 0 | 0 |
| Euphorbiaceae | *Dalechampia pernambucensis* | 0 | 0 | 0 | 0 | 0 | 0 | 0 | 8 | 0 |
| Thymelaeaceae | *Daphnopsis racemosa* | 0 | 0 | 3 | 0 | 0 | 0 | 0 | 0 | 0 |
| Thymelaeaceae | *Daphnopsis utilis* | 1 | 0 | 0 | 0 | 0 | 0 | 0 | 0 | 0 |
| Asteraceae | *Dasyphyllum candolleanum* | 1 | 0 | 0 | 0 | 0 | 0 | 0 | 0 | 0 |
| Asteraceae | *Dasyphyllum leptacanthum* | 1 | 0 | 0 | 0 | 0 | 0 | 0 | 0 | 0 |
| Asteraceae | *Dasyphyllum sprengelianum* | 1 | 2 | 0 | 4 | 0 | 0 | 0 | 8 | 0 |
| Solanaceae | *Datura stramonium* | 0 | 0 | 0 | 0 | 5 | 0 | 0 | 0 | 0 |
| Dilleniaceae | *Davilla cearensis* | 0 | 0 | 0 | 0 | 0 | 0 | 0 | 0 | 9 |
| Dilleniaceae | *Davilla elliptica* | 0 | 0 | 3 | 0 | 0 | 0 | 0 | 0 | 0 |
| Dilleniaceae | *Davilla macrocarpa* | 0 | 0 | 0 | 0 | 0 | 0 | 0 | 0 | 9 |
| Rubiaceae | *Declieuxia aspalathoides* | 1 | 0 | 0 | 0 | 0 | 0 | 0 | 0 | 0 |
| Rubiaceae | *Declieuxia fruticosa* | 1 | 0 | 0 | 0 | 0 | 0 | 0 | 0 | 0 |
| Fabaceae | *Deguelia costata* | 0 | 0 | 3 | 0 | 0 | 6 | 0 | 0 | 0 |
| Fabaceae | *Deguelia nitidula* | 0 | 0 | 3 | 0 | 0 | 6 | 0 | 0 | 0 |
| Asteraceae | *Delilia biflora* | 0 | 0 | 0 | 4 | 5 | 0 | 0 | 0 | 0 |
| Fabaceae | *Delonix regia* | 0 | 0 | 0 | 0 | 0 | 0 | 0 | 0 | 9 |
| Araliaceae | *Dendropanax geniculatus* | 1 | 0 | 0 | 0 | 0 | 0 | 0 | 0 | 0 |
| Fabaceae | *Desmanthus virgatus* | 0 | 2 | 0 | 4 | 5 | 6 | 0 | 0 | 0 |
| Fabaceae | *Desmodium barbatum* | 0 | 0 | 0 | 0 | 0 | 0 | 0 | 8 | 0 |
| Fabaceae | *Desmodium distortum* | 0 | 2 | 0 | 0 | 0 | 0 | 0 | 0 | 0 |
| Fabaceae | *Desmodium glabrum* | 0 | 0 | 0 | 0 | 0 | 0 | 7 | 8 | 0 |
| Fabaceae | *Desmodium incanum* | 0 | 0 | 0 | 4 | 0 | 0 | 0 | 0 | 0 |
| Fabaceae | *Desmodium tortuosum* | 0 | 0 | 0 | 4 | 0 | 0 | 0 | 0 | 0 |
| Fabaceae | *Desmodium uncinatum* | 0 | 0 | 0 | 4 | 0 | 0 | 0 | 0 | 0 |
| Asteraceae | *Diacranthera crenata* | 0 | 0 | 0 | 4 | 0 | 0 | 0 | 0 | 0 |
| Fabaceae | *Dialium guianense* | 0 | 0 | 0 | 4 | 0 | 0 | 0 | 0 | 9 |
| Acanthaceae | *Dicliptera mucronifolia* | 0 | 0 | 0 | 0 | 0 | 0 | 0 | 0 | 9 |
| Rutaceae | *Dictyoloma vandellianum* | 1 | 0 | 0 | 0 | 0 | 0 | 0 | 0 | 0 |
| Sapindaceae | *Dilodendron bipinnatum* | 0 | 0 | 3 | 0 | 0 | 6 | 0 | 0 | 0 |
| Fabaceae | *Dimorphandra exaltata* | 0 | 0 | 3 | 0 | 0 | 0 | 0 | 0 | 0 |
| Fabaceae | *Dimorphandra gardneriana* | 1 | 2 | 3 | 0 | 0 | 0 | 0 | 8 | 9 |
| Fabaceae | *Dimorphandra mollis* | 0 | 0 | 3 | 0 | 0 | 6 | 0 | 0 | 0 |
| Fabaceae | *Dioclea grandiflora* | 0 | 0 | 0 | 0 | 5 | 6 | 7 | 0 | 9 |
| Fabaceae | *Dioclea megacarpa* | 0 | 0 | 0 | 0 | 0 | 0 | 7 | 0 | 0 |
| Fabaceae | *Dioclea violacea* | 0 | 0 | 3 | 0 | 0 | 0 | 0 | 8 | 9 |
| Rubiaceae | *Diodella gardneri* | 0 | 0 | 0 | 0 | 5 | 0 | 0 | 0 | 0 |
| Ebenaceae | *Diospyros brasiliensis* | 0 | 0 | 3 | 0 | 5 | 0 | 0 | 0 | 0 |
| Ebenaceae | *Diospyros burchellii* | 0 | 2 | 0 | 0 | 0 | 0 | 0 | 0 | 0 |
| Ebenaceae | *Diospyros coccolobifolia* | 0 | 2 | 0 | 0 | 0 | 0 | 0 | 0 | 0 |
| Ebenaceae | *Diospyros hispida* | 0 | 0 | 3 | 0 | 5 | 0 | 0 | 0 | 0 |
| Ebenaceae | *Diospyros inconstans* | 1 | 2 | 3 | 0 | 5 | 6 | 7 | 8 | 0 |
| Ebenaceae | *Diospyros sericea* | 1 | 2 | 3 | 0 | 0 | 0 | 0 | 8 | 9 |
| Malpighiaceae | *Diplopterys pubipetala* | 1 | 0 | 0 | 0 | 0 | 0 | 0 | 0 | 9 |
| Fabaceae | *Diplotropis ferruginea* | 0 | 0 | 0 | 0 | 0 | 6 | 0 | 0 | 0 |
| Fabaceae | *Diplotropis incexis* | 0 | 0 | 3 | 4 | 0 | 0 | 0 | 0 | 0 |
| Fabaceae | *Diplotropis purpurea* | 0 | 0 | 3 | 0 | 0 | 0 | 0 | 0 | 0 |
| Lythraceae | *Diplusodon parvifolius* | 1 | 0 | 0 | 0 | 0 | 0 | 0 | 0 | 0 |
| Lythraceae | *Diplusodon ulei* | 1 | 0 | 0 | 0 | 0 | 0 | 0 | 0 | 0 |
| Fabaceae | *Dipteryx alata* | 0 | 0 | 0 | 0 | 0 | 0 | 0 | 0 | 9 |
| Fabaceae | *Dipteryx odorata* | 0 | 2 | 0 | 0 | 0 | 0 | 0 | 0 | 0 |
| Fabaceae | *Diptychandra aurantiaca* | 0 | 0 | 0 | 4 | 0 | 0 | 7 | 0 | 9 |
| Apocynaceae | *Ditassa pohliana* | 0 | 2 | 0 | 0 | 0 | 0 | 0 | 0 | 0 |
| Euphorbiaceae | *Ditaxis desertorum* | 0 | 0 | 3 | 0 | 0 | 6 | 7 | 8 | 0 |
| Euphorbiaceae | *Ditaxis malpighiacea* | 0 | 0 | 0 | 0 | 5 | 6 | 7 | 8 | 0 |
| Sapindaceae | *Dodonaea viscosa* | 1 | 0 | 0 | 0 | 0 | 0 | 0 | 0 | 0 |
| Sapindaceae | *Dodonaea viscosa.1* | 1 | 2 | 0 | 0 | 5 | 0 | 0 | 0 | 0 |
| Bignoniaceae | *Dolichandra quadrivalvis* | 0 | 2 | 0 | 0 | 5 | 0 | 0 | 0 | 0 |
| Bignoniaceae | *Dolichandra unguis.cati* | 0 | 0 | 0 | 0 | 0 | 6 | 0 | 0 | 0 |
| Dilleniaceae | *Doliocarpus sellowianus* | 0 | 2 | 0 | 0 | 0 | 0 | 0 | 0 | 0 |
| Moraceae | *Dorstenia caatingae* | 1 | 0 | 0 | 0 | 0 | 0 | 0 | 0 | 0 |
| Annonaceae | *Duguetia furfuracea* | 1 | 2 | 0 | 0 | 0 | 0 | 0 | 8 | 0 |
| Annonaceae | *Duguetia gardneriana* | 0 | 2 | 0 | 0 | 0 | 0 | 0 | 0 | 0 |
| Annonaceae | *Duguetia riedeliana* | 0 | 0 | 0 | 0 | 0 | 0 | 0 | 8 | 9 |
| Annonaceae | *Duguetia rotundifolia* | 0 | 0 | 0 | 0 | 5 | 0 | 0 | 0 | 0 |
| Annonaceae | *Duguetia scottmorii* | 0 | 0 | 3 | 0 | 0 | 0 | 0 | 0 | 0 |
| Verbenaceae | *Duranta erecta* | 0 | 0 | 0 | 4 | 0 | 0 | 0 | 0 | 0 |
| Amaranthaceae | *Dysphania ambrosioides* | 0 | 2 | 0 | 4 | 0 | 0 | 7 | 0 | 0 |
| Solanaceae | *Dyssochroma viridiflora* | 1 | 0 | 0 | 0 | 0 | 0 | 0 | 0 | 0 |
| Asteraceae | *Echinocoryne holosericea* | 1 | 0 | 0 | 0 | 0 | 0 | 0 | 0 | 0 |
| Asteraceae | *Elephantopus hirtiflorus* | 0 | 0 | 0 | 0 | 0 | 0 | 0 | 8 | 0 |
| Asteraceae | *Elvira biflora* | 0 | 0 | 0 | 4 | 0 | 0 | 0 | 0 | 0 |
| Rubiaceae | *Emmeorhiza umbellata* | 1 | 0 | 0 | 4 | 0 | 0 | 0 | 0 | 0 |
| Icacinaceae | *Emmotum affine* | 1 | 2 | 0 | 0 | 0 | 0 | 0 | 0 | 0 |
| Icacinaceae | *Emmotum nitens* | 1 | 2 | 3 | 0 | 0 | 0 | 7 | 8 | 0 |
| Fabaceae | *Enterolobium contortisiliquum* | 0 | 0 | 3 | 4 | 5 | 6 | 7 | 8 | 0 |
| Fabaceae | *Enterolobium gummiferum* | 1 | 0 | 0 | 0 | 0 | 0 | 0 | 0 | 0 |
| Fabaceae | *Enterolobium timbouva* | 0 | 2 | 3 | 4 | 0 | 0 | 0 | 0 | 9 |
| Annonaceae | *Ephedranthus parviflorus* | 0 | 0 | 0 | 0 | 0 | 0 | 7 | 0 | 9 |
| Annonaceae | *Ephedranthus pisocarpus* | 0 | 0 | 0 | 0 | 0 | 0 | 0 | 8 | 9 |
| Lamiaceae | *Eplingiella fruticosa* | 0 | 0 | 0 | 0 | 5 | 6 | 0 | 0 | 0 |
| Poaceae | *Eragrostis rufescens* | 0 | 0 | 0 | 0 | 5 | 0 | 0 | 0 | 0 |
| Asteraceae | *Eremanthus arboreus* | 1 | 2 | 0 | 0 | 0 | 0 | 0 | 0 | 0 |
| Asteraceae | *Eremanthus capitatus* | 1 | 2 | 3 | 4 | 5 | 0 | 0 | 0 | 0 |
| Asteraceae | *Eremanthus cinctus* | 1 | 0 | 0 | 0 | 0 | 0 | 0 | 0 | 0 |
| Asteraceae | *Eremanthus erythropappus* | 0 | 2 | 0 | 0 | 0 | 0 | 0 | 0 | 0 |
| Asteraceae | *Eremanthus glomerulatus* | 1 | 0 | 0 | 0 | 0 | 0 | 0 | 0 | 0 |
| Asteraceae | *Eremanthus harleyi* | 1 | 0 | 0 | 0 | 0 | 0 | 0 | 0 | 0 |
| Asteraceae | *Eremanthus incanus* | 1 | 0 | 0 | 0 | 0 | 0 | 0 | 0 | 0 |
| Asteraceae | *Eremanthus pohlii* | 0 | 2 | 0 | 0 | 0 | 0 | 0 | 0 | 0 |
| Lamiaceae | *Eriope anamariae* | 1 | 0 | 0 | 0 | 0 | 0 | 0 | 0 | 0 |
| Lamiaceae | *Eriope confusa* | 1 | 0 | 0 | 0 | 0 | 0 | 0 | 0 | 0 |
| Lamiaceae | *Eriope exaltata* | 1 | 0 | 0 | 0 | 0 | 0 | 0 | 0 | 0 |
| Lamiaceae | *Eriope glandulosa* | 1 | 0 | 0 | 0 | 0 | 0 | 0 | 0 | 0 |
| Lamiaceae | *Eriope hypenioides* | 1 | 0 | 0 | 0 | 0 | 0 | 7 | 0 | 0 |
| Lamiaceae | *Eriope latifolia* | 1 | 0 | 0 | 0 | 0 | 0 | 7 | 0 | 0 |
| Lamiaceae | *Eriope luetzelburgii* | 1 | 0 | 0 | 0 | 0 | 0 | 0 | 0 | 0 |
| Lamiaceae | *Eriope montana* | 1 | 0 | 0 | 0 | 0 | 0 | 0 | 0 | 0 |
| Lamiaceae | *Eriope polyphylla* | 1 | 0 | 0 | 0 | 0 | 0 | 0 | 0 | 0 |
| Lamiaceae | *Eriope sincorana* | 1 | 0 | 0 | 0 | 0 | 0 | 0 | 0 | 0 |
| Lamiaceae | *Eriopidion strictum* | 0 | 0 | 0 | 0 | 0 | 0 | 0 | 8 | 0 |
| Fabaceae | *Eriosema glaziovii* | 0 | 0 | 0 | 0 | 0 | 6 | 0 | 0 | 0 |
| Malvaceae | *Eriotheca crenulaticalyx* | 0 | 2 | 3 | 4 | 0 | 0 | 0 | 0 | 0 |
| Malvaceae | *Eriotheca globosa* | 0 | 0 | 3 | 0 | 0 | 0 | 0 | 0 | 0 |
| Malvaceae | *Eriotheca gracilipes* | 0 | 0 | 3 | 0 | 0 | 0 | 0 | 0 | 0 |
| Malvaceae | *Eriotheca macrophylla* | 0 | 0 | 3 | 0 | 0 | 0 | 0 | 0 | 0 |
| Malvaceae | *Eriotheca pubescens* | 0 | 0 | 3 | 0 | 0 | 0 | 0 | 0 | 0 |
| Fabaceae | *Erythrina falcata* | 0 | 0 | 3 | 0 | 0 | 0 | 0 | 0 | 0 |
| Fabaceae | *Erythrina velutina* | 0 | 0 | 3 | 4 | 5 | 6 | 7 | 0 | 9 |
| Fabaceae | *Erythrina verna* | 0 | 0 | 0 | 0 | 0 | 6 | 0 | 0 | 0 |
| Erythroxylaceae | *Erythroxylum affine* | 0 | 2 | 3 | 4 | 5 | 0 | 0 | 0 | 0 |
| Erythroxylaceae | *Erythroxylum amplifolium* | 0 | 2 | 0 | 0 | 0 | 0 | 0 | 8 | 9 |
| Erythroxylaceae | *Erythroxylum barbatum* | 0 | 2 | 3 | 0 | 0 | 0 | 7 | 8 | 9 |
| Erythroxylaceae | *Erythroxylum betulaceum* | 1 | 2 | 3 | 0 | 0 | 6 | 7 | 8 | 9 |
| Erythroxylaceae | *Erythroxylum bezerrae* | 0 | 0 | 0 | 0 | 0 | 0 | 0 | 0 | 9 |
| Erythroxylaceae | *Erythroxylum caatingae* | 0 | 0 | 0 | 4 | 5 | 0 | 7 | 8 | 0 |
| Erythroxylaceae | *Erythroxylum campestre* | 0 | 2 | 0 | 0 | 0 | 0 | 0 | 0 | 0 |
| Erythroxylaceae | *Erythroxylum citrifolium* | 0 | 2 | 3 | 4 | 0 | 0 | 0 | 8 | 0 |
| Erythroxylaceae | *Erythroxylum columbinum* | 0 | 0 | 0 | 4 | 0 | 0 | 0 | 0 | 0 |
| Erythroxylaceae | *Erythroxylum cuneifolium* | 0 | 2 | 0 | 0 | 0 | 0 | 0 | 0 | 0 |
| Erythroxylaceae | *Erythroxylum deciduum* | 0 | 2 | 3 | 0 | 0 | 6 | 0 | 0 | 0 |
| Erythroxylaceae | *Erythroxylum distortum* | 0 | 0 | 0 | 0 | 5 | 0 | 0 | 0 | 0 |
| Erythroxylaceae | *Erythroxylum flaccidum* | 0 | 0 | 3 | 0 | 0 | 0 | 0 | 0 | 0 |
| Erythroxylaceae | *Erythroxylum laetevirens* | 0 | 0 | 0 | 0 | 0 | 0 | 7 | 8 | 9 |
| Erythroxylaceae | *Erythroxylum lindemanii* | 0 | 0 | 0 | 0 | 5 | 0 | 0 | 0 | 9 |
| Erythroxylaceae | *Erythroxylum loefgrenii* | 1 | 2 | 0 | 0 | 0 | 0 | 0 | 8 | 0 |
| Erythroxylaceae | *Erythroxylum macrocalyx* | 0 | 0 | 3 | 0 | 0 | 0 | 0 | 0 | 0 |
| Erythroxylaceae | *Erythroxylum macrochaetum* | 0 | 0 | 0 | 0 | 5 | 0 | 0 | 0 | 0 |
| Erythroxylaceae | *Erythroxylum maracasense* | 0 | 0 | 0 | 0 | 5 | 0 | 0 | 8 | 0 |
| Erythroxylaceae | *Erythroxylum membranaceum* | 0 | 0 | 3 | 0 | 0 | 0 | 0 | 0 | 0 |
| Erythroxylaceae | *Erythroxylum mikanii* | 0 | 2 | 0 | 0 | 0 | 0 | 0 | 0 | 0 |
| Erythroxylaceae | *Erythroxylum mucronatum* | 0 | 2 | 0 | 4 | 5 | 0 | 0 | 0 | 0 |
| Erythroxylaceae | *Erythroxylum nobile* | 0 | 2 | 3 | 0 | 0 | 0 | 0 | 0 | 0 |
| Erythroxylaceae | *Erythroxylum nordestinum* | 0 | 0 | 0 | 0 | 5 | 0 | 0 | 0 | 0 |
| Erythroxylaceae | *Erythroxylum nummularia* | 0 | 0 | 0 | 4 | 5 | 0 | 7 | 8 | 9 |
| Erythroxylaceae | *Erythroxylum passerinum* | 0 | 2 | 3 | 0 | 5 | 0 | 0 | 8 | 0 |
| Erythroxylaceae | *Erythroxylum pauferrense* | 0 | 0 | 3 | 0 | 5 | 6 | 0 | 0 | 0 |
| Erythroxylaceae | *Erythroxylum petrae.caballi* | 0 | 0 | 0 | 4 | 0 | 0 | 0 | 0 | 0 |
| Erythroxylaceae | *Erythroxylum plowmanii* | 0 | 0 | 3 | 0 | 0 | 0 | 0 | 0 | 0 |
| Erythroxylaceae | *Erythroxylum polygonoides* | 1 | 0 | 3 | 4 | 0 | 0 | 0 | 0 | 0 |
| Erythroxylaceae | *Erythroxylum pulchrum* | 0 | 0 | 3 | 4 | 0 | 0 | 0 | 0 | 0 |
| Erythroxylaceae | *Erythroxylum pungens* | 0 | 0 | 0 | 0 | 5 | 6 | 7 | 0 | 9 |
| Erythroxylaceae | *Erythroxylum revolutum* | 0 | 0 | 0 | 4 | 5 | 6 | 0 | 0 | 0 |
| Erythroxylaceae | *Erythroxylum rimosum* | 0 | 2 | 0 | 0 | 0 | 0 | 0 | 8 | 0 |
| Erythroxylaceae | *Erythroxylum rosuliferum* | 0 | 2 | 0 | 0 | 0 | 0 | 0 | 8 | 0 |
| Erythroxylaceae | *Erythroxylum simonis* | 0 | 2 | 0 | 4 | 5 | 6 | 0 | 8 | 0 |
| Erythroxylaceae | *Erythroxylum squamatum* | 0 | 2 | 0 | 0 | 0 | 0 | 0 | 0 | 0 |
| Erythroxylaceae | *Erythroxylum stipulosum* | 0 | 2 | 0 | 0 | 0 | 0 | 0 | 8 | 9 |
| Erythroxylaceae | *Erythroxylum suberosum* | 1 | 2 | 0 | 0 | 0 | 0 | 0 | 0 | 9 |
| Erythroxylaceae | *Erythroxylum subglaucescens* | 0 | 2 | 0 | 4 | 5 | 0 | 0 | 0 | 0 |
| Erythroxylaceae | *Erythroxylum subracemosum* | 0 | 0 | 0 | 4 | 0 | 0 | 7 | 0 | 0 |
| Erythroxylaceae | *Erythroxylum subrotundum* | 1 | 0 | 3 | 4 | 5 | 0 | 0 | 0 | 0 |
| Erythroxylaceae | *Erythroxylum tianguanum* | 0 | 0 | 0 | 0 | 0 | 0 | 0 | 8 | 0 |
| Erythroxylaceae | *Erythroxylum vacciniifolium* | 1 | 2 | 3 | 0 | 0 | 0 | 0 | 8 | 9 |
| Lecythidaceae | *Eschweilera ovata* | 1 | 2 | 3 | 0 | 5 | 0 | 0 | 0 | 9 |
| Rutaceae | *Esenbeckia febrifuga* | 0 | 0 | 0 | 4 | 0 | 0 | 0 | 0 | 0 |
| Rutaceae | *Esenbeckia grandiflora* | 1 | 2 | 0 | 0 | 0 | 0 | 0 | 0 | 9 |
| Rutaceae | *Esenbeckia pumila* | 0 | 0 | 0 | 0 | 0 | 0 | 0 | 8 | 0 |
| Cactaceae | *Espostoopsis dybowskii* | 0 | 0 | 0 | 0 | 0 | 6 | 0 | 0 | 0 |
| Myrtaceae | *Eugenia acutata* | 0 | 2 | 0 | 0 | 0 | 0 | 0 | 0 | 0 |
| Myrtaceae | *Eugenia adstringens* | 0 | 0 | 0 | 0 | 5 | 0 | 0 | 0 | 0 |
| Myrtaceae | *Eugenia angustissima* | 1 | 0 | 0 | 0 | 0 | 0 | 0 | 0 | 0 |
| Myrtaceae | *Eugenia astringens* | 0 | 2 | 0 | 0 | 0 | 0 | 0 | 0 | 0 |
| Mytaceae | *Eugenia aurata* | 0 | 0 | 0 | 0 | 0 | 0 | 0 | 8 | 9 |
| Myrtaceae | *Eugenia azeda* | 0 | 0 | 0 | 0 | 0 | 0 | 0 | 8 | 0 |
| Myrtaceae | *Eugenia azuruensis* | 0 | 0 | 0 | 0 | 0 | 0 | 7 | 0 | 0 |
| Myrtaceae | *Eugenia biflora* | 0 | 0 | 0 | 0 | 5 | 0 | 0 | 0 | 9 |
| Myrtaceae | *Eugenia brejoensis* | 0 | 0 | 0 | 0 | 5 | 0 | 0 | 0 | 0 |
| Myrtaceae | *Eugenia cachoeirensis* | 0 | 2 | 0 | 0 | 0 | 0 | 0 | 0 | 0 |
| Myrtaceae | *Eugenia candolleana* | 0 | 2 | 3 | 4 | 5 | 0 | 0 | 0 | 0 |
| Myrtaceae | *Eugenia casearioides* | 0 | 0 | 0 | 0 | 0 | 0 | 0 | 0 | 9 |
| Myrtaceae | *Eugenia citrifolia* | 0 | 2 | 0 | 0 | 0 | 0 | 0 | 0 | 9 |
| Myrtaceae | *Eugenia convexinervia* | 0 | 2 | 0 | 0 | 0 | 0 | 0 | 0 | 0 |
| Myrtaceae | *Eugenia copacabanensis* | 1 | 0 | 0 | 0 | 0 | 0 | 0 | 0 | 0 |
| Myrtaceae | *Eugenia crenata* | 0 | 0 | 0 | 0 | 0 | 6 | 0 | 0 | 0 |
| Myrtaceae | *Eugenia dichroma* | 0 | 2 | 0 | 0 | 0 | 0 | 0 | 0 | 0 |
| Myrtaceae | *Eugenia dictyophleba* | 0 | 0 | 0 | 0 | 5 | 0 | 0 | 8 | 0 |
| Myrtaceae | *Eugenia dysenterica* | 0 | 0 | 3 | 0 | 5 | 6 | 0 | 8 | 9 |
| Myrtaceae | *Eugenia excelsa* | 0 | 2 | 0 | 0 | 0 | 0 | 0 | 8 | 0 |
| Myrtaceae | *Eugenia flavescens* | 0 | 2 | 3 | 0 | 5 | 6 | 7 | 8 | 9 |
| Myrtaceae | *Eugenia florida* | 0 | 2 | 3 | 4 | 0 | 6 | 0 | 0 | 0 |
| Myrtaceae | *Eugenia gomesiana* | 0 | 0 | 0 | 0 | 0 | 0 | 0 | 8 | 0 |
| Myrtaceae | *Eugenia hiemalis* | 1 | 0 | 0 | 0 | 0 | 0 | 0 | 0 | 0 |
| Myrtaceae | *Eugenia hirta* | 0 | 0 | 0 | 0 | 5 | 0 | 0 | 0 | 0 |
| Myrtaceae | *Eugenia hyemalis* | 0 | 0 | 0 | 4 | 0 | 0 | 0 | 0 | 0 |
| Myrtaceae | *Eugenia ilhensis* | 0 | 0 | 0 | 0 | 5 | 0 | 0 | 0 | 0 |
| Myrtaceae | *Eugenia lambertiana* | 0 | 0 | 0 | 0 | 5 | 0 | 0 | 0 | 0 |
| Myrtaceae | *Eugenia ligustrina* | 0 | 2 | 3 | 4 | 5 | 0 | 0 | 8 | 9 |
| Myrtaceae | *Eugenia luschnathiana* | 0 | 0 | 0 | 0 | 0 | 6 | 0 | 8 | 0 |
| Myrtaceae | *Eugenia lutescens* | 0 | 0 | 0 | 0 | 0 | 0 | 0 | 8 | 0 |
| Myrtaceae | *Eugenia mimus* | 0 | 2 | 0 | 4 | 0 | 0 | 0 | 0 | 0 |
| Myrtaceae | *Eugenia modesta* | 1 | 0 | 0 | 0 | 0 | 0 | 0 | 0 | 0 |
| Myrtaceae | *Eugenia pachnantha* | 0 | 2 | 0 | 0 | 0 | 0 | 0 | 0 | 9 |
| Myrtaceae | *Eugenia personii* | 0 | 0 | 0 | 4 | 0 | 0 | 0 | 0 | 0 |
| Myrtaceae | *Eugenia pistaciifolia* | 0 | 0 | 0 | 0 | 0 | 0 | 0 | 0 | 9 |
| Myrtaceae | *Eugenia pleurantha* | 0 | 0 | 0 | 4 | 0 | 0 | 0 | 0 | 0 |
| Myrtaceae | *Eugenia pseudopsidium* | 0 | 0 | 0 | 4 | 0 | 0 | 0 | 0 | 0 |
| Myrtaceae | *Eugenia punicifolia* | 1 | 2 | 3 | 4 | 5 | 0 | 7 | 8 | 9 |
| Myrtaceae | *Eugenia pyriformis* | 0 | 0 | 0 | 0 | 5 | 0 | 0 | 8 | 9 |
| Myrtaceae | *Eugenia rosea* | 0 | 0 | 0 | 0 | 5 | 0 | 0 | 8 | 0 |
| Myrtaceae | *Eugenia schottiana* | 0 | 0 | 0 | 0 | 5 | 0 | 0 | 0 | 0 |
| Myrtaceae | *Eugenia selloi* | 0 | 0 | 0 | 0 | 5 | 0 | 0 | 0 | 0 |
| Myrtaceae | *Eugenia sellowiana* | 0 | 0 | 0 | 0 | 0 | 0 | 0 | 8 | 0 |
| Myrtaceae | *Eugenia sonderiana* | 1 | 0 | 3 | 0 | 0 | 0 | 0 | 0 | 0 |
| Myrtaceae | *Eugenia splendens* | 1 | 0 | 0 | 0 | 0 | 0 | 0 | 0 | 0 |
| Myrtaceae | *Eugenia stictopetala* | 1 | 0 | 3 | 0 | 5 | 0 | 7 | 8 | 9 |
| Myrtaceae | *Eugenia subterminalis* | 0 | 2 | 0 | 0 | 0 | 0 | 0 | 0 | 0 |
| Myrtaceae | *Eugenia tapacumensis* | 1 | 0 | 0 | 0 | 0 | 0 | 0 | 0 | 0 |
| Myrtaceae | *Eugenia uniflora* | 1 | 0 | 3 | 0 | 0 | 0 | 0 | 0 | 0 |
| Myrtaceae | *Eugenia vernicosa* | 0 | 0 | 0 | 0 | 0 | 0 | 0 | 0 | 9 |
| Myrtaceae | *Eugenia vetula* | 1 | 0 | 0 | 0 | 0 | 0 | 0 | 0 | 0 |
| Euphorbiaceae | *Euphorbia appariciana* | 1 | 0 | 0 | 0 | 0 | 0 | 0 | 0 | 0 |
| Euphorbiaceae | *Euphorbia comosa* | 0 | 2 | 0 | 4 | 5 | 0 | 7 | 0 | 9 |
| Euphorbiaceae | *Euphorbia lycioides* | 0 | 0 | 0 | 0 | 0 | 0 | 0 | 8 | 0 |
| Euphorbiaceae | *Euphorbia phosphorea* | 0 | 0 | 0 | 0 | 5 | 6 | 7 | 0 | 0 |
| Euphorbiaceae | *Euphorbia potentilloides* | 1 | 0 | 0 | 0 | 0 | 0 | 0 | 0 | 0 |
| Euphorbiaceae | *Euphorbia pulcherrima* | 0 | 0 | 0 | 4 | 0 | 0 | 0 | 0 | 0 |
| Euphorbiaceae | *Euphorbia tirucalli* | 0 | 0 | 0 | 0 | 5 | 6 | 0 | 8 | 0 |
| Proteaceae | *Euplassa legalis* | 1 | 0 | 0 | 0 | 0 | 0 | 0 | 0 | 0 |
| Proteaceae | *Euplassa rufa* | 0 | 0 | 3 | 0 | 0 | 0 | 0 | 0 | 0 |
| Boraginaceae | *Euploca polyphylla* | 0 | 0 | 0 | 0 | 0 | 0 | 0 | 8 | 0 |
| Arecaceae | *Euterpe edulis* | 1 | 0 | 0 | 0 | 0 | 0 | 0 | 0 | 0 |
| Convulvulaceae | *Evolvulus elaeagnifolius* | 0 | 0 | 0 | 0 | 5 | 0 | 7 | 0 | 0 |
| Convolvulaceae | *Evolvulus elegans* | 0 | 0 | 0 | 0 | 5 | 0 | 0 | 0 | 0 |
| Convulvulaceae | *Evolvulus frankenioides* | 0 | 0 | 0 | 0 | 5 | 0 | 0 | 0 | 0 |
| Convolvulaceae | *Evolvulus latifolius* | 0 | 0 | 0 | 0 | 5 | 0 | 0 | 0 | 0 |
| Convulculaceae | *Evolvulus macroblepharis* | 0 | 0 | 0 | 0 | 0 | 0 | 0 | 0 | 9 |
| Convolvulaceae | *Evolvulus pterocaulon* | 0 | 0 | 0 | 0 | 0 | 0 | 0 | 8 | 0 |
| Rutaceae | *Fagara tenuifolia* | 0 | 0 | 3 | 4 | 5 | 0 | 0 | 0 | 0 |
| Rubiaceae | *Faramea hyacinthina* | 1 | 2 | 3 | 4 | 5 | 0 | 0 | 0 | 0 |
| Rubiaceae | *Faramea nigrescens* | 1 | 0 | 0 | 0 | 0 | 0 | 0 | 0 | 0 |
| Rubiaceae | *Faramea nitida* | 1 | 2 | 0 | 0 | 0 | 0 | 0 | 8 | 0 |
| Moraceae | *Ficus americana* | 0 | 2 | 3 | 0 | 0 | 0 | 7 | 0 | 9 |
| Moraceae | *Ficus arpazusa* | 0 | 2 | 0 | 4 | 0 | 0 | 7 | 0 | 0 |
| Moraceae | *Ficus bahiensis* | 0 | 2 | 0 | 0 | 0 | 0 | 0 | 0 | 0 |
| Moraceae | *Ficus benjamina* | 0 | 0 | 0 | 0 | 0 | 6 | 0 | 0 | 9 |
| Moraceae | *Ficus calyptroceras* | 0 | 0 | 3 | 0 | 0 | 0 | 0 | 8 | 0 |
| Moraceae | *Ficus christianii* | 0 | 0 | 3 | 0 | 5 | 6 | 0 | 0 | 9 |
| Moraceae | *Ficus citrifolia* | 0 | 2 | 0 | 4 | 0 | 0 | 0 | 0 | 0 |
| Moraceae | *Ficus cyclophylla* | 0 | 0 | 0 | 0 | 5 | 0 | 0 | 0 | 0 |
| Moraceae | *Ficus elastica* | 0 | 0 | 0 | 0 | 0 | 0 | 0 | 0 | 9 |
| Moraceae | *Ficus elliotiana* | 0 | 2 | 0 | 0 | 5 | 0 | 0 | 0 | 0 |
| Moraceae | *Ficus enormis* | 0 | 0 | 0 | 0 | 0 | 0 | 0 | 8 | 0 |
| Moraceae | *Ficus gomelleira* | 0 | 2 | 0 | 4 | 0 | 0 | 7 | 8 | 9 |
| Moraceae | *Ficus guaranitica* | 0 | 0 | 0 | 0 | 0 | 6 | 0 | 0 | 0 |
| Moraceae | *Ficus luschnathiana* | 0 | 0 | 0 | 0 | 0 | 0 | 7 | 0 | 0 |
| Moraceae | *Ficus maxima* | 0 | 0 | 0 | 4 | 0 | 0 | 0 | 0 | 0 |
| Moraceae | *Ficus mexiae* | 0 | 0 | 0 | 0 | 5 | 0 | 0 | 0 | 0 |
| Moraceae | *Ficus nymphaeifolia* | 0 | 0 | 0 | 4 | 0 | 0 | 0 | 0 | 0 |
| Moraceae | *Ficus obtusifolia* | 0 | 0 | 3 | 0 | 5 | 0 | 0 | 0 | 0 |
| Moraceae | *Ficus pertusa* | 0 | 2 | 0 | 0 | 0 | 0 | 0 | 0 | 0 |
| Moraceae | *Ficus trigonata* | 0 | 0 | 0 | 4 | 0 | 0 | 0 | 0 | 0 |
| Moraceae | *Ficus umbellata* | 0 | 0 | 3 | 0 | 0 | 0 | 0 | 0 | 0 |
| Apocynaceae | *Forsteronia pubescens* | 0 | 2 | 0 | 0 | 0 | 0 | 0 | 0 | 0 |
| Celastraceae | *Fraunhofera multiflora* | 0 | 0 | 3 | 0 | 0 | 6 | 7 | 0 | 0 |
| Bignoniaceae | *Fridericia chica* | 0 | 0 | 0 | 0 | 0 | 0 | 7 | 0 | 9 |
| Bignoniaceae | *Fridericia dispar* | 0 | 2 | 0 | 0 | 0 | 6 | 0 | 8 | 0 |
| Bignoniaceae | *Fridericia erubescens* | 0 | 0 | 0 | 0 | 5 | 0 | 7 | 0 | 0 |
| Bignoniaceae | *Fridericia limae* | 0 | 0 | 0 | 0 | 0 | 0 | 0 | 8 | 0 |
| Bignoniaceae | *Fridericia platyphylla* | 1 | 2 | 0 | 4 | 0 | 0 | 0 | 0 | 0 |
| Bignoniaceae | *Fridericia pliciflora* | 0 | 0 | 0 | 0 | 0 | 0 | 0 | 8 | 0 |
| Fabaceae | *Galactia martii* | 1 | 0 | 0 | 0 | 0 | 0 | 0 | 0 | 0 |
| Rubiaceae | *Galianthe brasiliensis* | 1 | 0 | 0 | 0 | 0 | 0 | 0 | 0 | 0 |
| Rutaceae | *Galipea trifoliata* | 0 | 0 | 0 | 0 | 0 | 0 | 0 | 0 | 9 |
| Phytolaccaceae | *Gallesia integrifolia* | 0 | 2 | 0 | 4 | 5 | 0 | 0 | 0 | 0 |
| Malpighiaceae | *Galphimia brasiliensis* | 0 | 0 | 0 | 0 | 5 | 6 | 0 | 0 | 0 |
| Clusiaceae | *Garcinia brasiliensis* | 1 | 0 | 0 | 0 | 0 | 0 | 0 | 0 | 0 |
| Clusiaceae | *Garcinia gardneriana* | 1 | 2 | 0 | 0 | 5 | 0 | 0 | 0 | 0 |
| Malvaceae | *Gaya aurea* | 0 | 0 | 0 | 0 | 5 | 6 | 0 | 0 | 0 |
| Malvaceae | *Gaya gaudichaudiana* | 0 | 2 | 0 | 0 | 5 | 0 | 0 | 0 | 0 |
| Malvaceae | *Gaya gracilipes* | 0 | 0 | 0 | 0 | 0 | 0 | 7 | 0 | 0 |
| Malvaceae | *Gaya pilosa* | 0 | 0 | 0 | 4 | 0 | 0 | 0 | 0 | 0 |
| Ericaceae | *Gaylussacia brasiliensis* | 1 | 0 | 0 | 0 | 0 | 0 | 0 | 0 | 0 |
| Ericaceae | *Gaylussacia densa* | 1 | 0 | 0 | 0 | 0 | 0 | 0 | 0 | 0 |
| Ericaceae | *Gaylussacia harleyi* | 1 | 0 | 0 | 0 | 0 | 0 | 0 | 0 | 0 |
| Ericaceae | *Gaylussacia incana* | 1 | 0 | 0 | 0 | 0 | 0 | 0 | 0 | 0 |
| Ericaceae | *Gaylussacia reticulata* | 1 | 0 | 0 | 0 | 0 | 0 | 0 | 0 | 0 |
| Ericaceae | *Gaylussacia retusa* | 1 | 0 | 0 | 0 | 0 | 0 | 0 | 0 | 0 |
| Ericaceae | *Gaylussacia virgata* | 1 | 0 | 0 | 0 | 0 | 0 | 0 | 0 | 0 |
| Acanthaceae | *Geissomeria macrophylla* | 0 | 2 | 0 | 0 | 0 | 0 | 0 | 0 | 0 |
| Rubiaceae | *Genipa americana* | 0 | 2 | 3 | 0 | 5 | 6 | 7 | 8 | 9 |
| Fabaceae | *Geoffroea spinosa* | 0 | 0 | 0 | 0 | 5 | 6 | 0 | 0 | 0 |
| Arecaceae | *Geonoma brevispatha* | 1 | 0 | 0 | 0 | 0 | 0 | 0 | 0 | 0 |
| Arecaceae | *Geonoma pohliana* | 1 | 0 | 0 | 0 | 0 | 0 | 0 | 0 | 0 |
| Arecaceae | *Geonoma schottiana* | 1 | 0 | 0 | 0 | 0 | 0 | 0 | 0 | 0 |
| Asteraceae | *Gochnatia discoidea* | 1 | 0 | 0 | 0 | 0 | 0 | 0 | 0 | 0 |
| Asteraceae | *Gochnatia floribunda* | 1 | 0 | 0 | 0 | 0 | 0 | 0 | 0 | 0 |
| Asteraceae | *Gochnatia polymorpha* | 0 | 0 | 3 | 4 | 0 | 0 | 0 | 0 | 0 |
| Bignoniaceae | *Godmania dardanoi* | 1 | 0 | 0 | 0 | 0 | 0 | 7 | 8 | 0 |
| Amaranthaceae | *Gomphrena demissa* | 1 | 0 | 0 | 0 | 0 | 0 | 0 | 0 | 0 |
| Amaranthaceae | *Gomphrena mollis* | 1 | 0 | 0 | 0 | 0 | 0 | 0 | 0 | 0 |
| Amaranthaceae | *Gomphrena vaga* | 0 | 0 | 0 | 0 | 5 | 6 | 7 | 0 | 0 |
| Fabaceae | *Goniorrhachis marginata* | 1 | 0 | 3 | 4 | 5 | 6 | 0 | 0 | 0 |
| Theaceae | *Gordonia fruticosa* | 1 | 0 | 0 | 0 | 0 | 0 | 0 | 0 | 0 |
| Malvaceae | *Gossypium hirsutum* | 1 | 0 | 0 | 0 | 0 | 0 | 0 | 0 | 0 |
| Rhamnaceae | *Gouania latifolia* | 0 | 0 | 0 | 4 | 5 | 0 | 0 | 0 | 0 |
| Nyctaginaceae | *Guapira campestris* | 0 | 0 | 0 | 0 | 0 | 0 | 0 | 0 | 9 |
| Nyctaginaceae | *Guapira graciliflora* | 0 | 0 | 0 | 0 | 0 | 0 | 7 | 8 | 9 |
| Nyctaginaceae | *Guapira hirsuta* | 0 | 0 | 0 | 4 | 5 | 0 | 0 | 0 | 0 |
| Nyctaginaceae | *Guapira laxa* | 0 | 0 | 0 | 0 | 5 | 6 | 7 | 8 | 0 |
| Nyctaginaceae | *Guapira nitida* | 0 | 2 | 0 | 0 | 0 | 0 | 0 | 8 | 0 |
| Nyctaginaceae | *Guapira noxia* | 0 | 0 | 3 | 0 | 5 | 6 | 7 | 0 | 0 |
| Nyctaginaceae | *Guapira obtusata* | 1 | 2 | 0 | 0 | 0 | 0 | 0 | 0 | 0 |
| Nyctaginaceae | *Guapira opposita* | 1 | 2 | 3 | 4 | 5 | 6 | 7 | 0 | 9 |
| Nyctaginaceae | *Guapira pernambucensis* | 0 | 2 | 0 | 4 | 0 | 0 | 0 | 8 | 0 |
| Nyctaginaceae | *Guapira tomentosa* | 0 | 0 | 0 | 0 | 5 | 6 | 0 | 8 | 0 |
| Meliaceae | *Guarea guidonia* | 0 | 0 | 0 | 4 | 0 | 0 | 0 | 0 | 0 |
| Meliaceae | *Guarea macrophylla* | 0 | 2 | 0 | 4 | 0 | 0 | 0 | 0 | 0 |
| Annonaceae | *Guatteria pogonopus* | 0 | 2 | 0 | 0 | 0 | 0 | 0 | 0 | 0 |
| Annonaceae | *Guatteria pohliana* | 1 | 0 | 0 | 0 | 0 | 0 | 0 | 0 | 0 |
| Annonaceae | *Guatteria sellowiana* | 1 | 0 | 0 | 0 | 0 | 0 | 0 | 0 | 0 |
| Malvaceae | *Guazuma ulmifolia* | 1 | 2 | 3 | 4 | 5 | 6 | 7 | 0 | 9 |
| Rubiaceae | *Guettarda angelica* | 0 | 2 | 3 | 0 | 5 | 6 | 7 | 8 | 9 |
| Rubiaceae | *Guettarda platypoda* | 0 | 2 | 0 | 0 | 5 | 6 | 0 | 8 | 0 |
| Rubiaceae | *Guettarda sericea* | 0 | 0 | 3 | 0 | 5 | 0 | 0 | 0 | 0 |
| Rubiaceae | *Guettarda uruguensis* | 0 | 0 | 3 | 0 | 0 | 0 | 0 | 0 | 0 |
| Rubiaceae | *Guettarda viburnoides* | 0 | 2 | 0 | 0 | 5 | 0 | 0 | 8 | 9 |
| Caesalpinioideae | *Guibourtia hymenaefolia* | 0 | 0 | 3 | 0 | 0 | 0 | 0 | 0 | 0 |
| Lecythidaceae | *Gustavia augusta* | 0 | 0 | 0 | 0 | 0 | 0 | 0 | 0 | 9 |
| Asteraceae | *Gymnanthemum amygdalinum* | 0 | 2 | 0 | 0 | 0 | 0 | 0 | 0 | 0 |
| Euphorbiaceae | *Gymnanthes boticario* | 0 | 0 | 0 | 0 | 5 | 0 | 7 | 8 | 9 |
| Euphorbiaceae | *Gymnanthes hypoleuca* | 0 | 0 | 3 | 0 | 0 | 0 | 0 | 0 | 0 |
| Rubiaceae | *Hamelia patens* | 0 | 2 | 0 | 0 | 0 | 0 | 0 | 0 | 0 |
| Apocynaceae | *Hancornia speciosa* | 1 | 2 | 0 | 0 | 5 | 0 | 0 | 8 | 9 |
| Bignoniaceae | *Handroanthus chrysotrichus* | 0 | 0 | 3 | 4 | 5 | 6 | 0 | 0 | 9 |
| Bignoniaceae | *Handroanthus heptaphyllus* | 0 | 0 | 3 | 0 | 0 | 6 | 0 | 8 | 0 |
| Bignoniaceae | *Handroanthus impetiginosus* | 0 | 0 | 3 | 4 | 5 | 6 | 7 | 8 | 9 |
| Bignoniaceae | *Handroanthus ochraceus* | 0 | 2 | 3 | 4 | 0 | 6 | 0 | 8 | 0 |
| Bignoniaceae | *Handroanthus selachidentatus* | 0 | 0 | 0 | 0 | 5 | 0 | 7 | 0 | 0 |
| Bignoniaceae | *Handroanthus serratifolius* | 0 | 2 | 3 | 4 | 5 | 6 | 7 | 8 | 9 |
| Bignoniaceae | *Handroanthus spongiosus* | 0 | 0 | 3 | 0 | 0 | 6 | 0 | 0 | 0 |
| Bignoniaceae | *Handroanthus vellosoi* | 0 | 0 | 0 | 0 | 0 | 6 | 0 | 0 | 0 |
| Fabaceae | *Harpalyce brasiliana* | 0 | 2 | 0 | 0 | 0 | 0 | 0 | 8 | 9 |
| Acanthaceae | *Harpochilus neesianus* | 0 | 0 | 0 | 0 | 5 | 0 | 7 | 8 | 0 |
| Acanthaceae | *Harpochilus phaeocarpus* | 0 | 0 | 0 | 0 | 0 | 0 | 7 | 0 | 0 |
| Cactaceae | *Harrisia adscendens* | 0 | 0 | 0 | 0 | 5 | 6 | 0 | 8 | 0 |
| Chloranthaceae | *Hedyosmum brasiliense* | 1 | 0 | 0 | 0 | 0 | 0 | 0 | 0 | 0 |
| Olacaceae | *Heisteria ovata* | 0 | 0 | 0 | 0 | 0 | 0 | 0 | 0 | 9 |
| Olacaceae | *Heisteria perianthomega* | 1 | 2 | 0 | 0 | 0 | 0 | 0 | 0 | 0 |
| Heliconiaceae | *Heliconia angusta* | 0 | 0 | 0 | 4 | 0 | 0 | 0 | 0 | 0 |
| Heliconiaceae | *Heliconia psittacorum* | 0 | 0 | 0 | 4 | 0 | 0 | 0 | 0 | 0 |
| Moraceae | *Helicostylis tomentosa* | 0 | 0 | 3 | 0 | 0 | 0 | 0 | 0 | 0 |
| Malvaceae | *Helicteres baruensis* | 0 | 0 | 0 | 0 | 5 | 6 | 7 | 8 | 0 |
| Malvaceae | *Helicteres brevispira* | 1 | 0 | 0 | 4 | 0 | 6 | 0 | 0 | 0 |
| Malvaceae | *Helicteres corylifolia* | 0 | 0 | 0 | 0 | 0 | 0 | 0 | 0 | 9 |
| Malvaceae | *Helicteres eichleri* | 1 | 0 | 0 | 0 | 5 | 6 | 0 | 0 | 9 |
| Malvaceae | *Helicteres eitenii* | 0 | 0 | 0 | 0 | 0 | 0 | 0 | 8 | 0 |
| Malvaceae | *Helicteres guazumifolia* | 0 | 0 | 0 | 0 | 5 | 6 | 0 | 0 | 0 |
| Malvaceae | *Helicteres heptandra* | 0 | 2 | 0 | 0 | 0 | 6 | 7 | 8 | 9 |
| Malvaceae | *Helicteres laciniosa* | 0 | 2 | 0 | 0 | 0 | 0 | 0 | 0 | 0 |
| Malvaceae | *Helicteres lhotzkyana* | 0 | 0 | 0 | 0 | 5 | 0 | 0 | 0 | 0 |
| Malvaceae | *Helicteres macropetala* | 0 | 0 | 0 | 4 | 5 | 0 | 7 | 0 | 0 |
| Malvaceae | *Helicteres muscosa* | 0 | 0 | 0 | 0 | 0 | 0 | 7 | 8 | 9 |
| Malvaceae | *Helicteres ovata* | 0 | 0 | 0 | 0 | 5 | 0 | 0 | 0 | 0 |
| Malvaceae | *Helicteres sacarolha* | 0 | 0 | 0 | 0 | 0 | 0 | 7 | 0 | 0 |
| Malvaceae | *Helicteres velutina* | 1 | 0 | 0 | 4 | 5 | 0 | 0 | 8 | 0 |
| Malvaceae | *Helicteres vuarame* | 0 | 0 | 0 | 0 | 5 | 0 | 0 | 0 | 0 |
| Rutaceae | *Helietta apiculata* | 0 | 0 | 0 | 0 | 0 | 6 | 0 | 0 | 0 |
| Boraginaceae | *Heliotropium angiospermum* | 0 | 0 | 0 | 4 | 5 | 0 | 0 | 0 | 0 |
| Boraginaceae | *Heliotropium indicum* | 0 | 0 | 0 | 4 | 0 | 0 | 0 | 0 | 0 |
| Melastomataceae | *Henriettea succosa* | 0 | 2 | 0 | 0 | 0 | 0 | 0 | 0 | 0 |
| Malvaceae | *Herissantia crispa* | 0 | 2 | 0 | 4 | 5 | 6 | 7 | 8 | 0 |
| Malvaceae | *Herissantia tiubae* | 0 | 0 | 0 | 4 | 5 | 6 | 7 | 0 | 0 |
| Asteraceae | *Heterocoma erecta* | 1 | 0 | 0 | 0 | 0 | 0 | 0 | 0 | 0 |
| Malpighiaceae | *Heteropterys arenaria* | 0 | 0 | 0 | 0 | 0 | 0 | 0 | 0 | 9 |
| Malpighiaceae | *Heteropterys byrsonimifolia* | 0 | 0 | 3 | 0 | 0 | 0 | 0 | 0 | 0 |
| Malpighiaceae | *Heteropterys discolor* | 0 | 0 | 0 | 0 | 0 | 0 | 0 | 8 | 0 |
| Malpighiaceae | *Heteropterys eglandulosa* | 1 | 0 | 0 | 0 | 0 | 0 | 0 | 0 | 0 |
| Malpighiaceae | *Heteropterys leschenaultiana* | 0 | 0 | 0 | 4 | 0 | 0 | 0 | 0 | 0 |
| Malpighiaceae | *Heteropterys pteropetala* | 1 | 0 | 0 | 0 | 0 | 0 | 7 | 0 | 0 |
| Malpighiaceae | *Heteropterys sincorensis* | 1 | 0 | 0 | 0 | 0 | 0 | 0 | 0 | 0 |
| Malpighiaceae | *Heteropterys syringifolia* | 0 | 0 | 0 | 0 | 5 | 0 | 0 | 0 | 0 |
| Malpighiaceae | *Heteropterys trichanthera* | 0 | 0 | 0 | 0 | 0 | 0 | 7 | 8 | 9 |
| Malpighiaceae | *Heteropterys umbellata* | 0 | 0 | 0 | 0 | 0 | 0 | 0 | 8 | 0 |
| Rubiaceae | *Hexasepalum apiculatum* | 0 | 2 | 0 | 0 | 0 | 0 | 7 | 8 | 0 |
| Rubiaceae | *Hexasepalum teres* | 0 | 0 | 0 | 0 | 0 | 0 | 7 | 0 | 0 |
| Malvaceae | *Hibiscus sabdariffa* | 1 | 0 | 0 | 0 | 0 | 0 | 0 | 0 | 0 |
| Phyllanthaceae | *Hieronyma alchorneoides* | 1 | 0 | 0 | 0 | 0 | 0 | 0 | 0 | 0 |
| Rubiaceae | *Hillia parasitica* | 1 | 2 | 0 | 0 | 0 | 0 | 0 | 0 | 0 |
| Apocynaceae | *Himatanthus articulatus* | 1 | 2 | 3 | 0 | 0 | 0 | 0 | 0 | 9 |
| Apocynaceae | *Himatanthus bracteatus* | 1 | 0 | 0 | 4 | 5 | 0 | 0 | 0 | 0 |
| Apocynaceae | *Himatanthus drasticus* | 1 | 2 | 3 | 0 | 5 | 0 | 7 | 8 | 9 |
| Apocynaceae | *Himatanthus lancifolius* | 1 | 2 | 0 | 0 | 0 | 0 | 0 | 0 | 0 |
| Apocynaceae | *Himatanthus obovatus* | 0 | 0 | 3 | 0 | 0 | 0 | 0 | 0 | 0 |
| Apocynaceae | *Himatanthus phagedaenicus* | 0 | 0 | 3 | 0 | 0 | 0 | 0 | 0 | 0 |
| Chrysobalanaceae | *Hirtella ciliata* | 0 | 2 | 0 | 4 | 5 | 0 | 0 | 8 | 9 |
| Chrysobalanaceae | *Hirtella glandulosa* | 1 | 2 | 3 | 0 | 0 | 0 | 0 | 0 | 0 |
| Chrysobalanaceae | *Hirtella gracilipes* | 0 | 2 | 3 | 0 | 0 | 0 | 0 | 0 | 0 |
| Chrysobalanaceae | *Hirtella hebeclada* | 0 | 0 | 3 | 0 | 0 | 0 | 0 | 0 | 0 |
| Chrysobalanaceae | *Hirtella martiana* | 1 | 0 | 0 | 0 | 0 | 0 | 0 | 0 | 0 |
| Chrysobalanaceae | *Hirtella racemosa* | 1 | 2 | 0 | 4 | 5 | 0 | 0 | 8 | 9 |
| Asteraceae | *Hoehnephytum almasense* | 1 | 0 | 0 | 0 | 0 | 0 | 0 | 0 | 0 |
| Asteraceae | *Hoehnephytum trixoides* | 1 | 0 | 0 | 0 | 0 | 0 | 0 | 0 | 0 |
| Rutaceae | *Hortia brasiliana* | 1 | 2 | 0 | 0 | 0 | 0 | 0 | 0 | 0 |
| Humiriaceae | *Humiria balsamifera* | 1 | 2 | 0 | 0 | 0 | 0 | 0 | 8 | 9 |
| Hydroleaceae | *Hydrolea spinosa* | 0 | 2 | 0 | 0 | 0 | 0 | 7 | 8 | 0 |
| Fabaceae | *Hymenaea aurea* | 0 | 0 | 0 | 0 | 0 | 0 | 0 | 8 | 0 |
| Fabaceae | *Hymenaea courbaril* | 1 | 2 | 3 | 4 | 5 | 6 | 7 | 8 | 9 |
| Fabaceae | *Hymenaea eriogyne* | 0 | 2 | 3 | 0 | 0 | 0 | 7 | 8 | 9 |
| Fabaceae | *Hymenaea longifolia* | 0 | 0 | 0 | 0 | 0 | 0 | 7 | 0 | 9 |
| Fabaceae | *Hymenaea martiana* | 0 | 2 | 3 | 0 | 5 | 6 | 7 | 8 | 9 |
| Fabaceae | *Hymenaea rubriflora* | 0 | 0 | 0 | 0 | 0 | 0 | 0 | 8 | 0 |
| Fabaceae | *Hymenaea stigonocarpa* | 1 | 2 | 3 | 0 | 5 | 6 | 7 | 8 | 9 |
| Fabaceae | *Hymenaea velutina* | 0 | 0 | 0 | 0 | 0 | 0 | 7 | 8 | 9 |
| Fabaceae | *Hymenolobium janeirense* | 0 | 2 | 0 | 4 | 0 | 0 | 0 | 0 | 0 |
| Lamiaceae | *Hypenia salzmannii* | 0 | 0 | 0 | 0 | 5 | 0 | 7 | 0 | 0 |
| Lamiaceae | *Hyptidendron amethystoides* | 0 | 2 | 0 | 0 | 0 | 0 | 0 | 0 | 0 |
| Lamiaceae | *Hyptis atrorubens* | 0 | 0 | 0 | 0 | 0 | 0 | 0 | 0 | 9 |
| Lamiaceae | *Hyptis crenata* | 0 | 0 | 0 | 0 | 0 | 0 | 0 | 0 | 9 |
| Lamiaceae | *Hyptis dilatata* | 0 | 0 | 0 | 0 | 0 | 0 | 7 | 0 | 0 |
| Lamiaceae | *Hyptis lantanifolia* | 1 | 0 | 0 | 0 | 0 | 0 | 0 | 0 | 0 |
| Lamiaceae | *Hyptis pectinata* | 0 | 2 | 0 | 4 | 5 | 0 | 0 | 0 | 0 |
| Lamiaceae | *Hyptis recurvata* | 1 | 0 | 0 | 0 | 0 | 0 | 0 | 0 | 0 |
| Asteraceae | *Ichthyothere terminalis* | 1 | 0 | 0 | 0 | 0 | 0 | 0 | 0 | 0 |
| Aquifoliaceae | *Ilex affinis* | 1 | 0 | 0 | 0 | 0 | 0 | 0 | 0 | 0 |
| Aquifoliaceae | *Ilex brevicuspis* | 0 | 0 | 3 | 0 | 0 | 6 | 0 | 0 | 0 |
| Aquifoliaceae | *Ilex dumosa* | 1 | 0 | 0 | 0 | 0 | 0 | 0 | 0 | 0 |
| Aquifoliaceae | *Ilex lundii* | 1 | 0 | 0 | 0 | 0 | 0 | 0 | 0 | 0 |
| Aquifoliaceae | *Ilex paraguariensis* | 1 | 0 | 0 | 0 | 0 | 0 | 0 | 0 | 0 |
| Aquifoliaceae | *Ilex sapotifolia* | 0 | 2 | 0 | 0 | 0 | 0 | 0 | 0 | 0 |
| Aquifoliaceae | *Ilex subcordata* | 0 | 0 | 0 | 0 | 0 | 0 | 7 | 0 | 0 |
| Aquifoliaceae | *Ilex theezans* | 1 | 0 | 0 | 0 | 0 | 0 | 0 | 0 | 0 |
| Aquifoliaceae | *Ilex theizans* | 1 | 0 | 0 | 0 | 0 | 0 | 0 | 0 | 0 |
| Aquifoliaceae | *Ilex velutina* | 1 | 0 | 0 | 0 | 0 | 0 | 0 | 0 | 0 |
| Fabaceae | *Indigofera blanchetiana* | 0 | 0 | 0 | 0 | 0 | 6 | 0 | 0 | 9 |
| Fabaceae | *Indigofera hirsuta* | 0 | 0 | 0 | 4 | 0 | 0 | 0 | 8 | 0 |
| Fabaceae | *Indigofera lespedezioides* | 0 | 0 | 0 | 0 | 5 | 0 | 0 | 0 | 0 |
| Fabaceae | *Indigofera microcarpa* | 0 | 0 | 0 | 0 | 0 | 6 | 7 | 8 | 0 |
| Fabaceae | *Indigofera suffruticosa* | 0 | 0 | 3 | 4 | 5 | 6 | 7 | 8 | 0 |
| Fabaceae | *Inga alba* | 0 | 2 | 0 | 0 | 0 | 0 | 0 | 0 | 0 |
| Fabaceae | *Inga bollandii* | 0 | 2 | 0 | 0 | 0 | 0 | 0 | 0 | 0 |
| Fabaceae | *Inga capitata* | 0 | 2 | 3 | 4 | 5 | 0 | 0 | 0 | 0 |
| Fabaceae | *Inga cayennensis* | 0 | 2 | 0 | 0 | 0 | 0 | 0 | 0 | 0 |
| Fabaceae | *Inga ciliata* | 0 | 2 | 0 | 0 | 0 | 0 | 0 | 0 | 0 |
| Fabaceae | *Inga ingoides* | 0 | 2 | 3 | 0 | 0 | 0 | 0 | 0 | 9 |
| Fabaceae | *Inga laurina* | 0 | 2 | 3 | 0 | 5 | 0 | 7 | 0 | 9 |
| Fabaceae | *Inga marginata* | 0 | 2 | 0 | 4 | 0 | 0 | 0 | 0 | 0 |
| Fabaceae | *Inga sessilis* | 0 | 0 | 3 | 0 | 0 | 0 | 0 | 0 | 0 |
| Fabaceae | *Inga striata* | 0 | 0 | 3 | 4 | 0 | 0 | 0 | 0 | 0 |
| Fabaceae | *Inga subnuda* | 0 | 2 | 3 | 4 | 0 | 0 | 0 | 0 | 0 |
| Fabaceae | *Inga tenuis* | 0 | 2 | 0 | 0 | 0 | 0 | 0 | 0 | 0 |
| Fabaceae | *Inga thibaudiana* | 0 | 2 | 0 | 4 | 5 | 0 | 0 | 0 | 0 |
| Fabaceae | *Inga vera* | 0 | 2 | 3 | 4 | 5 | 0 | 0 | 0 | 0 |
| Convolvulaceae | *Ipomoea brasiliana* | 0 | 0 | 0 | 0 | 5 | 0 | 0 | 0 | 0 |
| Convolvulaceae | *Ipomoea carnea* | 0 | 0 | 0 | 4 | 5 | 6 | 0 | 0 | 0 |
| Convolvulaceae | *Ipomoea incarnata* | 0 | 0 | 0 | 0 | 0 | 0 | 7 | 0 | 0 |
| Rubiaceae | *Ixora bracteolaris* | 0 | 0 | 3 | 0 | 0 | 0 | 0 | 0 | 0 |
| Rubiaceae | *Ixora brevifolia* | 0 | 0 | 3 | 0 | 0 | 0 | 0 | 8 | 0 |
| Rubiaceae | *Ixora chinensis* | 0 | 0 | 0 | 0 | 0 | 0 | 0 | 0 | 9 |
| Rubiaceae | *Ixora coccinea* | 0 | 0 | 0 | 0 | 0 | 0 | 0 | 0 | 9 |
| Rubiaceae | *Ixora finlaysoniana* | 0 | 0 | 0 | 0 | 0 | 0 | 0 | 0 | 9 |
| Rubiaceae | *Ixora syringiflora* | 0 | 2 | 0 | 0 | 0 | 0 | 0 | 0 | 0 |
| Bignoniaceae | *Jacaranda brasiliana* | 0 | 0 | 3 | 0 | 5 | 0 | 7 | 0 | 9 |
| Bignoniaceae | *Jacaranda caroba* | 0 | 0 | 3 | 0 | 0 | 0 | 0 | 0 | 0 |
| Bignoniaceae | *Jacaranda grandifoliolata* | 0 | 0 | 0 | 4 | 0 | 0 | 0 | 0 | 0 |
| Bignoniaceae | *Jacaranda irwinii* | 1 | 2 | 0 | 0 | 0 | 0 | 0 | 0 | 9 |
| Bignoniaceae | *Jacaranda jasminoides* | 1 | 2 | 0 | 0 | 5 | 0 | 7 | 8 | 9 |
| Bignoniaceae | *Jacaranda mimosifolia* | 1 | 0 | 0 | 0 | 0 | 0 | 0 | 0 | 9 |
| Bignoniaceae | *Jacaranda obovata* | 0 | 2 | 0 | 0 | 0 | 0 | 0 | 0 | 0 |
| Bignoniaceae | *Jacaranda praetermissa* | 0 | 0 | 0 | 0 | 0 | 0 | 7 | 0 | 9 |
| Bignoniaceae | *Jacaranda puberula* | 1 | 0 | 0 | 0 | 0 | 0 | 0 | 0 | 0 |
| Bignoniaceae | *Jacaranda rugosa* | 0 | 0 | 0 | 4 | 5 | 0 | 0 | 0 | 0 |
| Caricaceae | *Jacaratia corumbensis* | 0 | 0 | 0 | 0 | 5 | 6 | 7 | 0 | 0 |
| Caricaceae | *Jacaratia spinosa* | 0 | 0 | 0 | 4 | 0 | 0 | 0 | 0 | 0 |
| Convolvulaceae | *Jacquemontia confusa* | 0 | 0 | 0 | 0 | 5 | 0 | 0 | 0 | 0 |
| Convolvulaceae | *Jacquemontia pentanthos* | 0 | 0 | 0 | 0 | 0 | 0 | 7 | 0 | 0 |
| Malpighiaceae | *Janusia mediterranea* | 0 | 0 | 0 | 0 | 0 | 0 | 7 | 0 | 0 |
| Euphorbiaaceae | *Jatropha curcas* | 0 | 0 | 0 | 0 | 5 | 0 | 0 | 0 | 0 |
| Euphorbiaaceae | *Jatropha gossypiifolia* | 0 | 0 | 0 | 0 | 5 | 6 | 7 | 8 | 9 |
| Euphorbiaceae | *Jatropha martiusii* | 0 | 0 | 3 | 0 | 5 | 0 | 0 | 0 | 9 |
| Euphorbiaceae | *Jatropha mollissima* | 0 | 0 | 0 | 0 | 5 | 6 | 7 | 8 | 9 |
| Euphorbiaceae | *Jatropha mutabilis* | 0 | 0 | 0 | 0 | 5 | 6 | 7 | 8 | 0 |
| Euphorbiaceae | *Jatropha ribifolia* | 0 | 0 | 0 | 0 | 5 | 6 | 7 | 8 | 0 |
| Euphorbiaceae | *Joannesia princeps* | 0 | 2 | 0 | 0 | 0 | 0 | 0 | 0 | 0 |
| Acanthaceae | *Justicia aequilabris* | 0 | 0 | 0 | 0 | 5 | 0 | 7 | 8 | 9 |
| Acanthaceae | *Justicia lepida* | 1 | 0 | 0 | 0 | 0 | 0 | 0 | 0 | 0 |
| Calophyllaceae | *Kielmeyera bifaria* | 1 | 0 | 0 | 0 | 0 | 0 | 0 | 0 | 0 |
| Calophyllaceae | *Kielmeyera coriacea* | 1 | 0 | 3 | 0 | 0 | 0 | 0 | 0 | 0 |
| Calophyllaceae | *Kielmeyera cuspidata* | 1 | 0 | 0 | 0 | 0 | 0 | 0 | 0 | 0 |
| Calophyllaceae | *Kielmeyera lathrophyton* | 1 | 0 | 0 | 0 | 0 | 0 | 0 | 0 | 0 |
| Calophyllaceae | *Kielmeyera neglecta* | 0 | 2 | 0 | 0 | 0 | 0 | 0 | 0 | 0 |
| Calophyllaceae | *Kielmeyera neriifolia* | 1 | 0 | 0 | 0 | 0 | 0 | 0 | 0 | 0 |
| Calophyllaceae | *Kielmeyera petiolaris* | 1 | 0 | 0 | 0 | 0 | 0 | 0 | 0 | 0 |
| Calophyllaceae | *Kielmeyera rubriflora* | 0 | 0 | 3 | 0 | 0 | 0 | 0 | 0 | 0 |
| Calophyllaceae | *Kielmeyera speciosa* | 0 | 0 | 3 | 0 | 0 | 0 | 0 | 0 | 0 |
| Calophyllaceae | *Kielmeyera tomentosa* | 1 | 0 | 0 | 0 | 0 | 0 | 0 | 0 | 0 |
| Asteraceae | *Koanophyllon adamantium* | 1 | 0 | 0 | 0 | 0 | 0 | 0 | 0 | 0 |
| Asteraceae | *Koanophyllon conglobatum* | 0 | 0 | 0 | 0 | 5 | 0 | 0 | 0 | 0 |
| Krameriaceae | *Krameria argentea* | 0 | 2 | 0 | 0 | 0 | 0 | 0 | 0 | 9 |
| Krameriaceae | *Krameria grandiflora* | 0 | 0 | 0 | 0 | 5 | 0 | 0 | 0 | 0 |
| Krameriaceae | *Krameria tomentosa* | 1 | 2 | 0 | 0 | 5 | 0 | 7 | 8 | 9 |
| Malvaceae | *Krapovickasia flavescens* | 0 | 0 | 0 | 0 | 0 | 6 | 0 | 0 | 0 |
| Lacistemataceae | *Lacistema robustum* | 0 | 2 | 0 | 4 | 0 | 0 | 0 | 0 | 0 |
| Rubiaceae | *Ladenbergia cujabensis* | 0 | 0 | 3 | 0 | 0 | 0 | 0 | 0 | 0 |
| Rubiaceae | *Ladenbergia hexandra* | 0 | 0 | 0 | 0 | 0 | 0 | 0 | 0 | 9 |
| Salicaceae | *Laetia americana* | 0 | 0 | 0 | 0 | 0 | 6 | 0 | 0 | 0 |
| Lythraceae | *Lafoensia glyptocarpa* | 0 | 2 | 0 | 4 | 5 | 0 | 0 | 0 | 0 |
| Lythraceae | *Lafoensia pacari* | 0 | 2 | 3 | 0 | 5 | 0 | 0 | 0 | 0 |
| Lythraceae | *Lafoensia vandelliana* | 1 | 2 | 0 | 0 | 0 | 6 | 0 | 8 | 9 |
| Combretaceae | *Laguncularia racemosa* | 0 | 0 | 0 | 0 | 0 | 0 | 7 | 8 | 0 |
| Cunoniaceae | *Lamanonia ternata* | 0 | 0 | 0 | 4 | 0 | 0 | 0 | 0 | 0 |
| Verbenaceae | *Lantana achyranthifolia* | 0 | 0 | 0 | 0 | 0 | 6 | 0 | 0 | 0 |
| Verbenaceae | *Lantana brasiliensis* | 0 | 0 | 0 | 0 | 0 | 0 | 0 | 0 | 9 |
| Verbenaceae | *Lantana caatingensis* | 1 | 0 | 0 | 0 | 5 | 0 | 0 | 8 | 0 |
| Verbenaceae | *Lantana camara* | 1 | 2 | 3 | 4 | 5 | 6 | 7 | 8 | 9 |
| Verbenaceae | *Lantana canescens* | 0 | 0 | 0 | 0 | 5 | 0 | 7 | 8 | 0 |
| Verbenaceae | *Lantana fucata* | 0 | 2 | 3 | 0 | 5 | 0 | 7 | 8 | 9 |
| Verbenaceae | *Lantana gracilis* | 1 | 0 | 0 | 0 | 0 | 0 | 0 | 0 | 0 |
| Verbenaceae | *Lantana hatschbachii* | 1 | 0 | 0 | 0 | 0 | 0 | 0 | 0 | 0 |
| Verbenaceae | *Lantana pohliana* | 0 | 0 | 0 | 0 | 0 | 0 | 7 | 0 | 0 |
| Theaceae | *Laplacea fruticosa* | 1 | 0 | 0 | 0 | 0 | 0 | 0 | 0 | 0 |
| Asteraceae | *Lasiolaena blanchetii* | 1 | 0 | 0 | 0 | 0 | 0 | 0 | 0 | 0 |
| Asteraceae | *Lasiolaena morii* | 1 | 0 | 0 | 0 | 0 | 0 | 0 | 0 | 0 |
| Melastomataceae | *Lavoisiera nervulosa* | 1 | 0 | 0 | 0 | 0 | 0 | 0 | 0 | 0 |
| Melastomataceae | *Leandra aurea* | 1 | 0 | 0 | 0 | 0 | 0 | 0 | 0 | 0 |
| Melastomataceae | *Leandra carassana* | 1 | 0 | 0 | 0 | 0 | 0 | 0 | 0 | 0 |
| Melastomataceae | *Leandra erostrata* | 1 | 0 | 0 | 0 | 0 | 0 | 0 | 0 | 0 |
| Melastomataceae | *Leandra fluminensis* | 1 | 0 | 0 | 0 | 0 | 0 | 0 | 0 | 0 |
| Melastomataceae | *Leandra melastomoides* | 1 | 0 | 0 | 0 | 0 | 0 | 0 | 0 | 0 |
| Melastomataceae | *Leandra paulina* | 1 | 0 | 0 | 0 | 0 | 0 | 0 | 0 | 0 |
| Melastomataceae | *Leandra quinquedentata* | 1 | 0 | 0 | 0 | 0 | 0 | 0 | 0 | 0 |
| Melastomataceae | *Leandra salicina* | 1 | 0 | 0 | 0 | 0 | 0 | 0 | 0 | 0 |
| Melastomataceae | *Leandra xantholasia* | 1 | 0 | 0 | 0 | 0 | 0 | 0 | 0 | 0 |
| Lecythidaceae | *Lecythis ibiriba* | 0 | 2 | 0 | 0 | 0 | 0 | 0 | 0 | 0 |
| Lecythidaceae | *Lecythis lurida* | 0 | 0 | 0 | 0 | 5 | 0 | 0 | 0 | 0 |
| Lecythidaceae | *Lecythis pisonis* | 0 | 0 | 0 | 0 | 5 | 0 | 7 | 0 | 0 |
| Eriocaulaceae | *Leiothrix angustifolia* | 1 | 0 | 0 | 0 | 0 | 0 | 0 | 0 | 0 |
| Eriocaulaceae | *Leiothrix flavescens* | 1 | 0 | 0 | 0 | 0 | 0 | 0 | 0 | 0 |
| Cactaceae | *Leocereus bahiensis* | 1 | 0 | 0 | 0 | 0 | 0 | 0 | 0 | 0 |
| Lamiaceae | *Leonotis nepetifolia* | 0 | 2 | 0 | 0 | 5 | 0 | 0 | 0 | 9 |
| Lamiaceae | *Leonurus sibiricus* | 0 | 2 | 0 | 0 | 0 | 0 | 0 | 0 | 0 |
| Asteraceae | *Lepidaploa.eriolepis* | 0 | 0 | 0 | 4 | 0 | 0 | 0 | 0 | 0 |
| Asteraceae | *Lepidaploa acutiangula* | 0 | 0 | 3 | 4 | 0 | 0 | 0 | 0 | 0 |
| Asteraceae | *Lepidaploa araripensis* | 0 | 2 | 0 | 0 | 0 | 0 | 0 | 0 | 0 |
| Asteraceae | *Lepidaploa arenaria* | 0 | 0 | 0 | 0 | 0 | 0 | 0 | 0 | 9 |
| Asteraceae | *Lepidaploa aurea* | 0 | 0 | 0 | 0 | 0 | 0 | 0 | 0 | 9 |
| Asteraceae | *Lepidaploa bahiana* | 1 | 0 | 0 | 0 | 0 | 0 | 0 | 0 | 0 |
| Asteraceae | *Lepidaploa chalybaea* | 1 | 0 | 0 | 4 | 5 | 0 | 7 | 0 | 9 |
| Asteraceae | *Lepidaploa cotoneaster* | 1 | 0 | 3 | 0 | 5 | 0 | 0 | 8 | 0 |
| Asteraceae | *Lepidaploa lilacina* | 0 | 0 | 0 | 0 | 0 | 0 | 0 | 8 | 0 |
| Asteraceae | *Lepidaploa pseudaurea* | 1 | 0 | 0 | 0 | 0 | 0 | 0 | 0 | 0 |
| Asteraceae | *Lepidaploa remotiflora* | 0 | 0 | 0 | 0 | 5 | 0 | 0 | 8 | 0 |
| Asteraceae | *Lepidaploa tombadorensis* | 1 | 0 | 0 | 0 | 0 | 0 | 0 | 0 | 0 |
| Lamiaceae | *Leptohyptis calida* | 0 | 0 | 0 | 0 | 5 | 0 | 0 | 0 | 0 |
| Lamiaceae | *Leptohyptis macrostachys* | 1 | 0 | 0 | 0 | 0 | 0 | 0 | 0 | 0 |
| Fabaceae | *Leptolobium dasycarpum* | 1 | 2 | 3 | 0 | 0 | 0 | 0 | 8 | 9 |
| Rubiaceae | *Leptoscela ruellioides* | 0 | 0 | 0 | 4 | 0 | 6 | 0 | 0 | 0 |
| Asteraceae | *Lessingianthus.rugulosus* | 0 | 0 | 0 | 0 | 0 | 0 | 0 | 0 | 9 |
| Asteraceae | *Lessingianthus farinosus* | 1 | 0 | 0 | 0 | 0 | 0 | 0 | 0 | 0 |
| Asteraceae | *Lessingianthus morii* | 0 | 0 | 3 | 0 | 0 | 0 | 0 | 0 | 0 |
| Asteraceae | *Lessingianthus obscurus* | 0 | 0 | 3 | 0 | 0 | 0 | 0 | 0 | 9 |
| Asteraceae | *Lessingianthus rosmarinifolius* | 1 | 0 | 0 | 0 | 0 | 0 | 0 | 0 | 0 |
| Fabaceae | *Leucaena leucocephala* | 0 | 0 | 0 | 0 | 0 | 0 | 7 | 0 | 0 |
| Fabaceae | *Leucochloron limae* | 0 | 0 | 3 | 0 | 0 | 6 | 0 | 0 | 0 |
| Fabaceae | *Libidibia ferrea* | 0 | 2 | 3 | 4 | 5 | 6 | 7 | 8 | 9 |
| Chrysobalanaceae | *Licania apetala* | 0 | 0 | 0 | 0 | 0 | 0 | 0 | 8 | 9 |
| Chrysobalanaceae | *Licania dealbata* | 1 | 0 | 0 | 0 | 0 | 0 | 0 | 0 | 0 |
| Chrysobalanaceae | *Licania gardneri* | 0 | 2 | 0 | 0 | 0 | 0 | 0 | 0 | 0 |
| Chrysobalanaceae | *Licania humilis* | 0 | 0 | 0 | 0 | 0 | 0 | 0 | 0 | 9 |
| Chrysobalanaceae | *Licania kunthiana* | 1 | 2 | 0 | 0 | 0 | 0 | 0 | 0 | 9 |
| Chrysobalanaceae | *Licania octandra* | 0 | 2 | 0 | 4 | 0 | 0 | 0 | 8 | 0 |
| Chrysobalanaceae | *Licania rigida* | 0 | 2 | 3 | 0 | 0 | 6 | 7 | 0 | 9 |
| Chrysobalanaceae | *Licania sclerophylla* | 0 | 0 | 0 | 0 | 0 | 0 | 7 | 0 | 0 |
| Chrysobalanaceae | *Licania tomentosa* | 0 | 0 | 3 | 0 | 0 | 0 | 0 | 8 | 9 |
| Achariaceae | *Lindackeria ovata* | 0 | 2 | 0 | 0 | 0 | 0 | 7 | 0 | 9 |
| Verbenaceae | *Lippia alba* | 1 | 0 | 0 | 0 | 5 | 6 | 0 | 0 | 0 |
| Verbenaceae | *Lippia alnifolia* | 1 | 0 | 0 | 0 | 0 | 0 | 0 | 0 | 0 |
| Verbenaceae | *Lippia gracilis* | 1 | 0 | 0 | 4 | 5 | 6 | 0 | 0 | 9 |
| Verbenaceae | *Lippia grata* | 1 | 0 | 0 | 0 | 5 | 6 | 0 | 0 | 0 |
| Verbenaceae | *Lippia insignis* | 1 | 0 | 0 | 0 | 0 | 0 | 7 | 0 | 0 |
| Verbenaceae | *Lippia lasiocalycina* | 0 | 0 | 0 | 0 | 5 | 0 | 0 | 0 | 0 |
| Verbenaceae | *Lippia macrophylla* | 0 | 2 | 0 | 4 | 5 | 0 | 0 | 0 | 0 |
| Verbenaceae | *Lippia microphylla* | 1 | 2 | 0 | 0 | 5 | 6 | 0 | 8 | 0 |
| Verbenaceae | *Lippia origanoides* | 1 | 2 | 0 | 0 | 5 | 6 | 7 | 8 | 0 |
| Verbenaceae | *Lippia rigida* | 1 | 0 | 0 | 4 | 5 | 0 | 0 | 0 | 0 |
| Verbenaceae | *Lippia sidoides* | 0 | 0 | 0 | 0 | 5 | 0 | 7 | 0 | 0 |
| Verbenaceae | *Lippia subracemosa* | 1 | 0 | 0 | 0 | 0 | 0 | 0 | 0 | 0 |
| Verbenaceae | *Lippia thymoides* | 0 | 0 | 0 | 0 | 5 | 0 | 7 | 0 | 0 |
| Anacardiaceae | *Lithrea molleoides* | 1 | 0 | 3 | 0 | 0 | 0 | 0 | 0 | 0 |
| Campanulaceae | *Lobelia organensis* | 1 | 0 | 0 | 0 | 0 | 0 | 0 | 0 | 0 |
| Campanulaceae | *Lobelia xalapensis* | 0 | 2 | 0 | 0 | 0 | 0 | 0 | 0 | 0 |
| Fabaceae | *Lonchocarpus sericeus* | 0 | 0 | 3 | 4 | 5 | 6 | 7 | 0 | 0 |
| Fabaceae | *Lonchocarpus virgilioides* | 0 | 0 | 0 | 0 | 5 | 0 | 0 | 0 | 0 |
| Onagraceae | *Ludwigia affinis* | 0 | 0 | 0 | 0 | 0 | 6 | 0 | 0 | 0 |
| Onagraceae | *Ludwigia albiflora* | 1 | 0 | 0 | 0 | 0 | 0 | 0 | 0 | 0 |
| Onagraceae | *Ludwigia elegans* | 0 | 0 | 0 | 0 | 0 | 6 | 0 | 0 | 0 |
| Onagraceae | *Ludwigia erecta* | 0 | 0 | 0 | 0 | 0 | 6 | 0 | 0 | 0 |
| Onagraceae | *Ludwigia leptocarpa* | 0 | 0 | 0 | 0 | 0 | 6 | 0 | 8 | 0 |
| Onagraceae | *Ludwigia octovalvis* | 0 | 0 | 0 | 4 | 0 | 6 | 0 | 0 | 0 |
| Onagraceae | *Ludwigia pilosa* | 0 | 0 | 0 | 0 | 5 | 0 | 0 | 0 | 0 |
| Onagraceae | *Ludwigia rigida* | 1 | 0 | 0 | 0 | 0 | 0 | 0 | 0 | 0 |
| Malvaceae | *Luehea candicans* | 0 | 0 | 3 | 0 | 0 | 0 | 7 | 8 | 9 |
| Malvaceae | *Luehea divaricata* | 0 | 2 | 3 | 0 | 0 | 6 | 0 | 0 | 0 |
| Malvaceae | *Luehea grandiflora* | 1 | 0 | 3 | 4 | 5 | 0 | 0 | 0 | 0 |
| Malvaceae | *Luehea ochrophylla* | 0 | 0 | 3 | 0 | 0 | 0 | 0 | 0 | 0 |
| Malvaceae | *Luehea paniculata* | 1 | 0 | 3 | 0 | 0 | 6 | 0 | 0 | 9 |
| Malvaceae | *Luehea speciosa* | 0 | 0 | 0 | 0 | 0 | 0 | 0 | 0 | 9 |
| Fabaceae | *Luetzelburgia auriculata* | 0 | 0 | 0 | 0 | 0 | 6 | 7 | 8 | 9 |
| Fabaceae | *Luetzelburgia bahiensis* | 0 | 0 | 0 | 0 | 0 | 6 | 7 | 0 | 0 |
| Fabaceae | *Luetzelburgia purpurea* | 0 | 0 | 0 | 0 | 5 | 0 | 0 | 0 | 0 |
| Bignoniaceae | *Lundia corymbifera* | 0 | 0 | 3 | 0 | 0 | 0 | 0 | 0 | 0 |
| Ochnaceae | *Luxemburgia diciliata* | 1 | 0 | 0 | 0 | 0 | 0 | 0 | 0 | 0 |
| Asteraceae | *Lychnophora granmogolensis* | 1 | 0 | 0 | 0 | 0 | 0 | 0 | 0 | 0 |
| Asteraceae | *Lychnophora phylicifolia* | 1 | 0 | 0 | 0 | 0 | 0 | 0 | 0 | 0 |
| Asteraceae | *Lychnophora regis* | 1 | 0 | 0 | 0 | 0 | 0 | 0 | 0 | 0 |
| Asteraceae | *Lychnophora salicifolia* | 1 | 0 | 0 | 0 | 0 | 0 | 0 | 0 | 0 |
| Asteraceae | *Lychnophora santosii* | 1 | 0 | 0 | 0 | 0 | 0 | 0 | 0 | 0 |
| Asteraceae | *Lychnophora triflora* | 1 | 0 | 0 | 0 | 0 | 0 | 0 | 0 | 0 |
| Solanaceae | *Lycium martii* | 0 | 0 | 0 | 0 | 5 | 0 | 0 | 0 | 0 |
| Euphorbiaceae | *Mabea fistulifera* | 0 | 2 | 0 | 0 | 0 | 0 | 0 | 0 | 0 |
| Euphorbiaceae | *Mabea occidentalis* | 0 | 0 | 0 | 4 | 0 | 0 | 0 | 0 | 0 |
| Melastomataceae | *Macairea radula* | 1 | 0 | 3 | 0 | 0 | 0 | 0 | 8 | 0 |
| Fabaceae | *Machaerium aculeatum* | 0 | 2 | 3 | 0 | 0 | 6 | 0 | 0 | 0 |
| Fabaceae | *Machaerium acutifolium* | 0 | 2 | 3 | 4 | 0 | 6 | 7 | 8 | 9 |
| Fabaceae | *Machaerium amplum* | 0 | 2 | 0 | 0 | 0 | 0 | 0 | 8 | 0 |
| Fabaceae | *Machaerium brasiliense* | 0 | 0 | 3 | 0 | 0 | 6 | 0 | 0 | 0 |
| Fabaceae | *Machaerium .oridum* | 0 | 0 | 0 | 0 | 0 | 6 | 0 | 0 | 0 |
| Fabaceae | *Machaerium hirtum* | 0 | 2 | 3 | 4 | 5 | 6 | 7 | 0 | 0 |
| Fabaceae | *Machaerium oblongifolium* | 0 | 0 | 0 | 4 | 0 | 0 | 0 | 0 | 0 |
| Fabaceae | *Machaerium opacum* | 1 | 0 | 3 | 0 | 0 | 6 | 0 | 0 | 0 |
| Fabaceae | *Machaerium salzmannii* | 0 | 0 | 3 | 0 | 0 | 0 | 0 | 0 | 0 |
| Fabaceae | *Machaerium scleroxylon* | 0 | 0 | 3 | 0 | 0 | 6 | 0 | 0 | 0 |
| Fabaceae | *Machaerium stipitatum* | 0 | 0 | 0 | 0 | 0 | 0 | 0 | 8 | 9 |
| Fabaceae | *Machaerium vestitum* | 0 | 0 | 0 | 0 | 0 | 0 | 0 | 8 | 9 |
| Fabaceae | *Machaerium villosum* | 0 | 0 | 3 | 0 | 0 | 6 | 0 | 0 | 0 |
| Rubiaceae | *Machaonia acuminata* | 1 | 0 | 3 | 0 | 5 | 6 | 0 | 0 | 0 |
| Moraceae | *Maclura tinctoria* | 0 | 2 | 3 | 4 | 5 | 6 | 7 | 8 | 9 |
| Fabaceae | *Macrolobium latifolium* | 0 | 2 | 0 | 0 | 0 | 0 | 0 | 0 | 0 |
| Monimiaceae | *Macropeplus ligustrinus* | 1 | 0 | 0 | 0 | 0 | 0 | 0 | 0 | 0 |
| Fabaceae | *Macroptilium bracteatum* | 0 | 2 | 0 | 4 | 0 | 0 | 7 | 0 | 0 |
| Fabaceae | *Macroptilium lathyroides* | 0 | 0 | 0 | 4 | 0 | 0 | 0 | 0 | 0 |
| Salicaceae | *Macrothumia kuhlmannii* | 0 | 0 | 3 | 0 | 0 | 0 | 0 | 0 | 0 |
| Sapindaceae | *Magonia pubescens* | 0 | 2 | 3 | 0 | 0 | 6 | 7 | 8 | 9 |
| Apocynaceae | *Malouetia cestroides* | 0 | 0 | 3 | 0 | 0 | 0 | 0 | 0 | 0 |
| Malpighiaceae | *Malpighia emarginata* | 0 | 0 | 0 | 0 | 0 | 6 | 0 | 0 | 0 |
| Malvaceae | *Malvastrum americanum* | 0 | 2 | 0 | 0 | 0 | 0 | 0 | 0 | 0 |
| Malvaceae | *Malvastrum coromandelianum* | 0 | 2 | 0 | 0 | 5 | 0 | 0 | 0 | 0 |
| Malvaceae | *Malvastrum tomentosum* | 0 | 0 | 0 | 0 | 0 | 6 | 7 | 0 | 0 |
| Apocynaceae | *Mandevilla alexicaca* | 1 | 0 | 0 | 0 | 0 | 0 | 0 | 0 | 0 |
| Apocynaceae | *Mandevilla bahiensis* | 1 | 0 | 0 | 0 | 0 | 0 | 0 | 0 | 0 |
| Apocynaceae | *Mandevilla dardanoi* | 0 | 0 | 0 | 4 | 0 | 0 | 0 | 0 | 0 |
| Apocynaceae | *Mandevilla emarginata* | 1 | 0 | 0 | 0 | 0 | 0 | 0 | 0 | 0 |
| Apocynaceae | *Mandevilla funiformis* | 0 | 0 | 0 | 0 | 5 | 0 | 0 | 0 | 0 |
| Apocynaceae | *Mandevilla illustris* | 1 | 0 | 0 | 0 | 0 | 0 | 0 | 0 | 0 |
| Apocynaceae | *Mandevilla moricandiana* | 1 | 0 | 0 | 0 | 0 | 0 | 0 | 0 | 0 |
| Apocynaceae | *Mandevilla sancta* | 1 | 0 | 0 | 0 | 0 | 0 | 0 | 0 | 0 |
| Rubiaceae | *Manettia cordifolia* | 1 | 0 | 0 | 4 | 0 | 0 | 0 | 0 | 0 |
| Anacardiaceae | *Mangifera indica* | 0 | 2 | 3 | 0 | 0 | 0 | 0 | 0 | 9 |
| Euphorbiaceae | *Manihot anomala* | 0 | 0 | 0 | 0 | 5 | 0 | 7 | 0 | 9 |
| Euphorbiaceae | *Manihot brachyandra* | 0 | 0 | 0 | 0 | 0 | 0 | 0 | 0 | 9 |
| Euphorbiaceae | *Manihot brachyloba* | 0 | 0 | 0 | 0 | 0 | 0 | 0 | 8 | 0 |
| Euphorbiaceae | *Manihot caerulescens* | 1 | 2 | 0 | 0 | 0 | 0 | 7 | 8 | 9 |
| Euphorbiaceae | *Manihot carthagenensis* | 0 | 0 | 3 | 0 | 5 | 6 | 7 | 8 | 9 |
| Euphorbiaceae | *Manihot catingae* | 0 | 0 | 0 | 0 | 0 | 6 | 0 | 0 | 0 |
| Euphorbiaceae | *Manihot dichotoma* | 0 | 0 | 0 | 0 | 5 | 6 | 7 | 8 | 9 |
| Euphorbiaceae | *Manihot epruinosa* | 0 | 0 | 3 | 0 | 0 | 0 | 0 | 0 | 0 |
| Euphorbiaceae | *Manihot heptaphylla* | 0 | 0 | 0 | 0 | 0 | 0 | 0 | 8 | 0 |
| Euphorbiaceae | *Manihot jacobinensis* | 1 | 0 | 0 | 0 | 0 | 0 | 0 | 0 | 0 |
| Euphorbiaceae | *Manihot palmata* | 0 | 2 | 0 | 0 | 0 | 6 | 0 | 0 | 9 |
| Euphorbiaceae | *Manihot pseudoglaziovii* | 0 | 0 | 0 | 0 | 0 | 6 | 0 | 0 | 0 |
| Euphorbiaceae | *Manihot reniformis* | 1 | 0 | 0 | 0 | 0 | 0 | 0 | 0 | 0 |
| Euphorbiaceae | *Manihot tripartita* | 0 | 0 | 0 | 0 | 0 | 0 | 0 | 8 | 0 |
| Euphorbiaceae | *Manihot tristis* | 0 | 0 | 0 | 0 | 0 | 0 | 0 | 8 | 0 |
| Sapotaceae | *Manilkara longifolia* | 0 | 2 | 0 | 0 | 0 | 0 | 0 | 0 | 0 |
| Sapotaceae | *Manilkara rufula* | 0 | 2 | 0 | 4 | 5 | 0 | 0 | 0 | 0 |
| Sapotaceae | *Manilkara salzmannii* | 0 | 2 | 0 | 4 | 5 | 0 | 0 | 8 | 0 |
| Sapotaceae | *Manilkara subsericea* | 0 | 0 | 0 | 0 | 0 | 6 | 0 | 0 | 0 |
| Sapotaceae | *Manilkara triflora* | 0 | 2 | 0 | 0 | 0 | 0 | 0 | 8 | 9 |
| Sapotaceae | *Manilkara zapota* | 0 | 0 | 0 | 0 | 0 | 0 | 0 | 0 | 9 |
| Euphorbiaceae | *Maprounea guianensis* | 1 | 2 | 3 | 4 | 5 | 6 | 7 | 8 | 9 |
| Melastomataceae | *Marcetia bahiensis* | 1 | 0 | 0 | 0 | 0 | 0 | 0 | 0 | 0 |
| Melastomataceae | *Marcetia canescens* | 1 | 0 | 0 | 0 | 0 | 0 | 0 | 0 | 0 |
| Melastomataceae | *Marcetia ericoides* | 1 | 0 | 0 | 0 | 0 | 0 | 0 | 0 | 0 |
| Melastomataceae | *Marcetia formosa* | 1 | 0 | 0 | 0 | 0 | 0 | 0 | 0 | 0 |
| Melastomataceae | *Marcetia grandiflora* | 1 | 0 | 0 | 0 | 0 | 0 | 0 | 0 | 0 |
| Melastomataceae | *Marcetia harleyi* | 1 | 0 | 0 | 0 | 0 | 0 | 0 | 0 | 0 |
| Melastomataceae | *Marcetia latifolia* | 1 | 0 | 0 | 0 | 0 | 0 | 0 | 0 | 0 |
| Melastomataceae | *Marcetia macrophylla* | 1 | 0 | 0 | 0 | 0 | 0 | 0 | 0 | 0 |
| Melastomataceae | *Marcetia taxifolia* | 1 | 0 | 0 | 0 | 0 | 0 | 0 | 0 | 0 |
| Melastomataceae | *Marcetia velutina* | 1 | 0 | 0 | 0 | 0 | 0 | 0 | 0 | 0 |
| Melastomataceae | *Marcetia viscida* | 1 | 0 | 0 | 0 | 0 | 0 | 0 | 0 | 0 |
| Phyllanthaceae | *Margaritaria nobilis* | 0 | 2 | 0 | 0 | 5 | 0 | 0 | 0 | 0 |
| Rubiaceae | *Margaritopsis astrellantha* | 0 | 0 | 3 | 0 | 0 | 0 | 0 | 0 | 0 |
| Rubiaceae | *Margaritopsis carrascoana* | 0 | 2 | 3 | 0 | 0 | 0 | 0 | 8 | 0 |
| Rubiaceae | *Margaritopsis chaenotricha* | 1 | 2 | 0 | 4 | 0 | 0 | 0 | 0 | 0 |
| Solanaceae | *Markea longipes* | 0 | 0 | 0 | 0 | 5 | 0 | 0 | 0 | 0 |
| Myrtaceae | *Marlierea clausseniana* | 0 | 2 | 0 | 4 | 0 | 0 | 0 | 0 | 0 |
| Myrtaceae | *Marlierea eugenioides* | 0 | 2 | 0 | 0 | 0 | 0 | 0 | 0 | 0 |
| Myrtaceae | *Marlierea excoriata* | 0 | 0 | 3 | 4 | 0 | 0 | 0 | 0 | 0 |
| Myrtaceae | *Marlierea ferruginea* | 0 | 0 | 0 | 0 | 0 | 0 | 0 | 8 | 0 |
| Myrtaceae | *Marlierea laevigata* | 1 | 0 | 0 | 0 | 0 | 0 | 0 | 0 | 0 |
| Myrtaceae | *Marlierea luschnathiana* | 1 | 0 | 0 | 0 | 0 | 0 | 0 | 0 | 0 |
| Myrtaceae | *Marlierea neuwiedeana* | 0 | 2 | 0 | 0 | 0 | 0 | 0 | 0 | 0 |
| Apocynaceae | *Marsdenia altissima* | 0 | 0 | 0 | 0 | 5 | 0 | 0 | 8 | 0 |
| Apocynaceae | *Marsdenia caatingae* | 0 | 0 | 0 | 4 | 0 | 0 | 0 | 0 | 0 |
| Lamiaceae | *Marsypianthes chamaedrys* | 0 | 0 | 0 | 0 | 5 | 0 | 7 | 8 | 0 |
| Lamiaceae | *Martianthus stachydifolius* | 0 | 0 | 0 | 0 | 0 | 6 | 0 | 0 | 0 |
| Fabaceae | *Martiodendron mediterraneum* | 0 | 0 | 0 | 0 | 0 | 0 | 7 | 0 | 9 |
| Malpighiaceae | *Mascagnia sepium* | 0 | 0 | 0 | 0 | 0 | 0 | 7 | 0 | 0 |
| Sapindaceae | *Matayba guianensis* | 1 | 2 | 3 | 0 | 0 | 0 | 0 | 8 | 9 |
| Sapindaceae | *Matayba heterophylla* | 1 | 0 | 0 | 0 | 0 | 0 | 0 | 0 | 0 |
| Arecaceae | *Mauritia flexuosa* | 0 | 0 | 3 | 0 | 0 | 0 | 0 | 0 | 0 |
| Arecaceae | *Mauritiella armata* | 0 | 0 | 3 | 0 | 0 | 0 | 0 | 0 | 0 |
| Celastraceae | *Maytenus acanthophylla* | 0 | 0 | 0 | 4 | 5 | 0 | 0 | 0 | 0 |
| Celastraceae | *Maytenus catingarum* | 1 | 2 | 0 | 0 | 0 | 0 | 0 | 0 | 9 |
| Celastraceae | *Maytenus distichophylla* | 0 | 2 | 3 | 4 | 5 | 0 | 0 | 8 | 0 |
| Celastraceae | *Maytenus erythroxyla* | 0 | 2 | 3 | 4 | 0 | 0 | 0 | 8 | 0 |
| Celastraceae | *Maytenus evonymoides* | 0 | 0 | 0 | 0 | 5 | 0 | 0 | 0 | 0 |
| Celastraceae | *Maytenus floribunda* | 0 | 0 | 3 | 0 | 0 | 0 | 0 | 0 | 0 |
| Celastraceae | *Maytenus gonoclada* | 1 | 0 | 0 | 0 | 0 | 0 | 0 | 8 | 0 |
| Celastraceae | *Maytenus imbricata* | 1 | 0 | 0 | 4 | 5 | 0 | 7 | 0 | 0 |
| Celastraceae | *Maytenus mucugensis* | 1 | 0 | 0 | 0 | 0 | 0 | 0 | 0 | 0 |
| Celastraceae | *Maytenus obtusifolia* | 0 | 2 | 3 | 0 | 5 | 0 | 7 | 8 | 0 |
| Celastraceae | *Maytenus opaca* | 1 | 2 | 0 | 0 | 0 | 0 | 0 | 0 | 0 |
| Celastraceae | *Maytenus patens* | 0 | 0 | 3 | 0 | 5 | 0 | 0 | 0 | 0 |
| Celastraceae | *Maytenus pruinosa* | 0 | 0 | 0 | 0 | 0 | 6 | 0 | 0 | 0 |
| Celastraceae | *Maytenus quadrangulata* | 0 | 0 | 0 | 4 | 0 | 0 | 0 | 0 | 0 |
| Celastraceae | *Maytenus rigida* | 0 | 0 | 3 | 4 | 5 | 6 | 7 | 8 | 0 |
| Celastraceae | *Maytenus robusta* | 0 | 2 | 3 | 0 | 0 | 0 | 0 | 0 | 0 |
| Malpighiaceae | *Mcvaughia bahiana* | 0 | 0 | 0 | 0 | 5 | 0 | 0 | 0 | 0 |
| Lamiaceae | *Medusantha martiusii* | 0 | 2 | 0 | 4 | 5 | 0 | 7 | 8 | 0 |
| Asteraceae | *Melampodium divaricatum* | 0 | 0 | 0 | 0 | 0 | 0 | 0 | 8 | 0 |
| Fabaceae | *Melanoxylon brauna* | 0 | 2 | 3 | 0 | 0 | 0 | 0 | 8 | 0 |
| Meliaceae | *Melia azedarach* | 0 | 0 | 0 | 0 | 5 | 0 | 0 | 0 | 0 |
| Lamiaceae | *Melissa officinalis* | 0 | 0 | 0 | 0 | 5 | 0 | 0 | 0 | 0 |
| Cactaceae | *Melocactus bahiensis* | 0 | 0 | 0 | 0 | 5 | 6 | 0 | 0 | 0 |
| Cactaceae | *Melocactus violaceus* | 0 | 0 | 0 | 0 | 0 | 6 | 0 | 0 | 0 |
| Cactaceae | *Melocactus zehntneri* | 0 | 0 | 0 | 0 | 5 | 6 | 7 | 0 | 0 |
| Malvaceae | *Melochia arenosa* | 0 | 0 | 0 | 0 | 0 | 0 | 0 | 0 | 9 |
| Malvaceae | *Melochia betonicifolia* | 0 | 0 | 0 | 0 | 5 | 6 | 0 | 8 | 0 |
| Malvaceae | *Melochia pyramidata* | 0 | 0 | 0 | 0 | 0 | 6 | 0 | 0 | 0 |
| Malvaceae | *Melochia rotundifolia* | 0 | 0 | 0 | 4 | 0 | 0 | 0 | 0 | 0 |
| Malvaceae | *Melochia tomentosa* | 0 | 0 | 0 | 4 | 5 | 6 | 7 | 8 | 0 |
| Loasaceae | *Mentzelia aspera* | 0 | 0 | 0 | 0 | 0 | 6 | 0 | 0 | 0 |
| Poaceae | *Merostachys fischeriana* | 0 | 2 | 0 | 0 | 0 | 0 | 0 | 0 | 0 |
| Convolvulaceae | *Merremia cissoides* | 0 | 0 | 0 | 0 | 0 | 0 | 7 | 0 | 0 |
| Lamiaceae | *Mesosphaerum irwinii* | 1 | 0 | 0 | 0 | 0 | 0 | 0 | 0 | 0 |
| Lamiaceae | *Mesosphaerum sidifolium* | 0 | 0 | 0 | 4 | 0 | 0 | 0 | 0 | 0 |
| Lamiaceae | *Mesosphaerum suaveolens* | 0 | 0 | 0 | 0 | 0 | 0 | 0 | 8 | 0 |
| Apocynaceae | *Metastelma harleyi* | 1 | 0 | 0 | 0 | 0 | 0 | 0 | 0 | 0 |
| Rutaceae | *Metrodorea concinna* | 1 | 0 | 0 | 0 | 0 | 0 | 0 | 0 | 0 |
| Solanaceae | *Metternichia princeps* | 0 | 0 | 3 | 4 | 0 | 0 | 0 | 0 | 0 |
| Lauraceae | *Mezilaurus revolutifolia* | 0 | 2 | 0 | 0 | 0 | 0 | 0 | 0 | 0 |
| Melastomataceae | *Miconia affinis* | 0 | 2 | 0 | 0 | 0 | 0 | 0 | 0 | 0 |
| Melastomataceae | *Miconia alata* | 0 | 2 | 0 | 0 | 0 | 0 | 0 | 0 | 0 |
| Melastomataceae | *Miconia albicans* | 1 | 2 | 3 | 0 | 5 | 0 | 0 | 0 | 9 |
| Melastomataceae | *Miconia alborufescens* | 1 | 2 | 0 | 0 | 0 | 0 | 0 | 0 | 0 |
| Melastomataceae | *Miconia amacurensis* | 0 | 2 | 0 | 0 | 0 | 0 | 0 | 0 | 0 |
| Melastomataceae | *Miconia amoena* | 0 | 2 | 0 | 0 | 0 | 0 | 0 | 0 | 0 |
| Melastomataceae | *Miconia budlejoides* | 0 | 0 | 3 | 0 | 0 | 0 | 0 | 0 | 0 |
| Melastomataceae | *Miconia calvescens* | 0 | 0 | 0 | 4 | 0 | 0 | 0 | 0 | 0 |
| Melastomataceae | *Miconia caudigera* | 1 | 2 | 0 | 4 | 0 | 0 | 0 | 0 | 0 |
| Melastomataceae | *Miconia chartacea* | 1 | 2 | 0 | 0 | 0 | 0 | 0 | 0 | 0 |
| Melastomataceae | *Miconia ciliata* | 1 | 2 | 0 | 4 | 5 | 0 | 0 | 8 | 9 |
| Melastomataceae | *Miconia cinnamomifolia* | 0 | 0 | 3 | 0 | 0 | 0 | 0 | 0 | 0 |
| Melastomataceae | *Miconia cuspidata* | 0 | 0 | 0 | 4 | 0 | 0 | 0 | 0 | 0 |
| Melastomataceae | *Miconia cyathanthera* | 1 | 0 | 0 | 0 | 0 | 0 | 0 | 0 | 0 |
| Melastomataceae | *Miconia dodecandra* | 1 | 2 | 0 | 4 | 0 | 0 | 0 | 0 | 0 |
| Melastomataceae | *Miconia elegans* | 1 | 0 | 0 | 0 | 0 | 0 | 0 | 0 | 0 |
| Melastomataceae | *Miconia falconi* | 0 | 0 | 0 | 4 | 0 | 0 | 0 | 0 | 0 |
| Melastomataceae | *Miconia flammea* | 1 | 0 | 0 | 0 | 0 | 0 | 0 | 0 | 0 |
| Melastomataceae | *Miconia holosericea* | 1 | 2 | 0 | 0 | 0 | 0 | 0 | 0 | 0 |
| Melastomataceae | *Miconia hypoleuca* | 0 | 2 | 0 | 0 | 0 | 0 | 0 | 0 | 0 |
| Melastomataceae | *Miconia ibaguensis* | 0 | 2 | 0 | 0 | 0 | 0 | 0 | 0 | 0 |
| Melastomataceae | *Miconia leandroides* | 0 | 0 | 0 | 4 | 0 | 0 | 0 | 0 | 0 |
| Melastomataceae | *Miconia lepidota* | 0 | 0 | 3 | 0 | 0 | 0 | 0 | 0 | 0 |
| Melastomataceae | *Miconia ligustroides* | 1 | 2 | 3 | 0 | 0 | 0 | 0 | 0 | 0 |
| Melastomataceae | *Miconia minutiflora* | 0 | 2 | 0 | 4 | 5 | 0 | 0 | 0 | 0 |
| Melastomataceae | *Miconia mirabilis* | 0 | 0 | 0 | 4 | 0 | 0 | 0 | 0 | 0 |
| Melastomataceae | *Miconia nervosa* | 0 | 2 | 0 | 4 | 0 | 0 | 0 | 0 | 0 |
| Melastomataceae | *Miconia nordestina* | 0 | 2 | 0 | 0 | 0 | 0 | 0 | 0 | 0 |
| Melastomataceae | *Miconia pepericarpa* | 1 | 0 | 0 | 0 | 0 | 0 | 0 | 0 | 0 |
| Melastomataceae | *Miconia prasina* | 1 | 2 | 3 | 4 | 0 | 0 | 0 | 0 | 0 |
| Melastomataceae | *Miconia pyrifolia* | 0 | 2 | 0 | 0 | 0 | 0 | 0 | 0 | 0 |
| Melastomataceae | *Miconia racemosa* | 0 | 0 | 0 | 0 | 0 | 0 | 0 | 8 | 0 |
| Melastomataceae | *Miconia rimalis* | 1 | 0 | 0 | 0 | 0 | 0 | 0 | 0 | 0 |
| Melastomataceae | *Miconia rubiginosa* | 1 | 0 | 3 | 0 | 0 | 0 | 0 | 0 | 0 |
| Melastomataceae | *Miconia sclerophylla* | 1 | 0 | 0 | 0 | 0 | 0 | 0 | 0 | 0 |
| Melastomataceae | *Miconia stenostachya* | 0 | 2 | 0 | 0 | 0 | 0 | 0 | 0 | 0 |
| Melastomataceae | *Miconia theizans* | 1 | 2 | 0 | 0 | 0 | 0 | 0 | 0 | 0 |
| Melastomataceae | *Microlicia amblysepala* | 1 | 0 | 0 | 0 | 0 | 0 | 0 | 0 | 0 |
| Melastomataceae | *Microlicia baccharoides* | 1 | 0 | 0 | 0 | 0 | 0 | 0 | 0 | 0 |
| Melastomataceae | *Microlicia balsamifera* | 1 | 0 | 0 | 0 | 0 | 0 | 0 | 0 | 0 |
| Melastomataceae | *Microlicia chrysantha* | 1 | 0 | 0 | 0 | 0 | 0 | 0 | 0 | 0 |
| Melastomataceae | *Microlicia cordata* | 1 | 0 | 0 | 0 | 0 | 0 | 0 | 0 | 0 |
| Melastomataceae | *Microlicia fasciculata* | 1 | 0 | 0 | 0 | 0 | 0 | 0 | 0 | 0 |
| Melastomataceae | *Microlicia fulva* | 1 | 0 | 0 | 0 | 0 | 0 | 0 | 0 | 0 |
| Melastomataceae | *Microlicia hatschbachii* | 1 | 0 | 0 | 0 | 0 | 0 | 0 | 0 | 0 |
| Melastomataceae | *Microlicia hirta* | 1 | 0 | 0 | 0 | 0 | 0 | 0 | 0 | 0 |
| Melastomataceae | *Microlicia isostemon* | 1 | 0 | 0 | 0 | 0 | 0 | 0 | 0 | 0 |
| Melastomataceae | *Microlicia longisepala* | 1 | 0 | 0 | 0 | 0 | 0 | 0 | 0 | 0 |
| Melastomataceae | *Microlicia luetzelburgii* | 1 | 0 | 0 | 0 | 0 | 0 | 0 | 0 | 0 |
| Melastomataceae | *Microlicia lutea* | 1 | 0 | 0 | 0 | 0 | 0 | 0 | 0 | 0 |
| Melastomataceae | *Microlicia monticola* | 1 | 0 | 0 | 0 | 0 | 0 | 0 | 0 | 0 |
| Melastomataceae | *Microlicia morii* | 1 | 0 | 0 | 0 | 0 | 0 | 0 | 0 | 0 |
| Melastomataceae | *Microlicia mucugensis* | 1 | 0 | 0 | 0 | 0 | 0 | 0 | 0 | 0 |
| Melastomataceae | *Microlicia petasensis* | 1 | 0 | 0 | 0 | 0 | 0 | 0 | 0 | 0 |
| Melastomataceae | *Microlicia semiriana* | 1 | 0 | 0 | 0 | 0 | 0 | 0 | 0 | 0 |
| Melastomataceae | *Microlicia subsetosa* | 1 | 0 | 0 | 0 | 0 | 0 | 0 | 0 | 0 |
| Melastomataceae | *Microlicia torrendii* | 1 | 0 | 0 | 0 | 0 | 0 | 0 | 0 | 0 |
| Melastomataceae | *Microlicia viminalis* | 1 | 0 | 0 | 0 | 0 | 0 | 0 | 0 | 0 |
| Sapotaceae | *Micropholis emarginata* | 1 | 0 | 0 | 0 | 0 | 0 | 0 | 0 | 0 |
| Sapotaceae | *Micropholis gardneriana* | 0 | 2 | 0 | 0 | 0 | 0 | 0 | 0 | 0 |
| Sapotaceae | *Micropholis gnaphaloclados* | 1 | 0 | 0 | 0 | 0 | 0 | 0 | 0 | 0 |
| Euphorbiaceae | *Microstachys corniculata* | 1 | 2 | 0 | 0 | 0 | 0 | 0 | 8 | 0 |
| Euphorbiaceae | *Microstachys daphnoides* | 1 | 0 | 0 | 0 | 0 | 0 | 0 | 8 | 0 |
| Euphorbiaceae | *Microstachys ditassoides* | 1 | 0 | 0 | 0 | 0 | 0 | 0 | 0 | 0 |
| Euphorbiaceae | *Microstachys heterodoxa* | 1 | 0 | 0 | 0 | 0 | 0 | 0 | 0 | 0 |
| Euphorbiaceae | *Microstachys hispida* | 1 | 0 | 0 | 4 | 0 | 0 | 0 | 0 | 0 |
| Euphorbiaceae | *Microstachys marginata* | 1 | 0 | 3 | 0 | 0 | 0 | 0 | 0 | 0 |
| Euphorbiaceae | *Microstachys serrulata* | 1 | 0 | 0 | 0 | 0 | 0 | 0 | 0 | 0 |
| Euphorbiaceae | *Microstachys uleana* | 1 | 0 | 0 | 0 | 0 | 0 | 0 | 0 | 0 |
| Phytolaccaceae | *Microtea glochidiata* | 0 | 0 | 0 | 4 | 5 | 0 | 0 | 0 | 0 |
| Asteraceae | *Mikania luetzelburgii* | 1 | 0 | 0 | 0 | 0 | 0 | 0 | 0 | 0 |
| Fabaceae | *Mimosa acutistipula* | 0 | 0 | 0 | 0 | 5 | 6 | 7 | 8 | 9 |
| Fabaceae | *Mimosa adenocarpa* | 1 | 0 | 0 | 0 | 0 | 0 | 0 | 0 | 0 |
| Fabaceae | *Mimosa adenophylla* | 0 | 0 | 0 | 0 | 5 | 6 | 0 | 8 | 0 |
| Fabaceae | *Mimosa arenosa* | 0 | 2 | 3 | 4 | 5 | 6 | 7 | 8 | 0 |
| Fabaceae | *Mimosa artemisiana* | 0 | 0 | 0 | 0 | 0 | 0 | 0 | 0 | 9 |
| Fabaceae | *Mimosa aurivillus* | 1 | 0 | 0 | 0 | 0 | 0 | 0 | 0 | 0 |
| Fabaceae | *Mimosa bimucronata* | 0 | 0 | 0 | 0 | 5 | 0 | 0 | 0 | 0 |
| Fabaceae | *Mimosa blanchetii* | 1 | 0 | 0 | 0 | 0 | 0 | 0 | 0 | 0 |
| Fabaceae | *Mimosa borboremae* | 0 | 0 | 0 | 0 | 0 | 6 | 0 | 0 | 0 |
| Fabaceae | *Mimosa brevipinna* | 0 | 0 | 0 | 0 | 5 | 0 | 0 | 0 | 0 |
| Fabaceae | *Mimosa caesalpiniifolia* | 0 | 2 | 3 | 0 | 5 | 6 | 7 | 8 | 9 |
| Fabaceae | *Mimosa camporum* | 0 | 2 | 0 | 0 | 5 | 0 | 0 | 0 | 0 |
| Fabaceae | *Mimosa candollei* | 0 | 0 | 0 | 4 | 5 | 0 | 7 | 0 | 9 |
| Fabaceae | *Mimosa cordistipula* | 1 | 0 | 0 | 0 | 0 | 0 | 0 | 0 | 0 |
| Fabaceae | *Mimosa debilis* | 0 | 0 | 0 | 4 | 0 | 0 | 0 | 0 | 0 |
| Fabaceae | *Mimosa gemmulata* | 0 | 0 | 0 | 0 | 5 | 0 | 0 | 0 | 0 |
| Fabaceae | *Mimosa guaranitica* | 0 | 0 | 0 | 0 | 5 | 0 | 0 | 0 | 0 |
| Fabaceae | *Mimosa hexandra* | 0 | 0 | 0 | 0 | 0 | 6 | 0 | 0 | 0 |
| Fabaceae | *Mimosa hirsutissima* | 0 | 0 | 0 | 0 | 0 | 0 | 0 | 8 | 0 |
| Fabaceae | *Mimosa hypoglauca* | 0 | 0 | 0 | 0 | 0 | 0 | 0 | 0 | 9 |
| Fabaceae | *Mimosa invisa* | 0 | 0 | 0 | 0 | 0 | 0 | 7 | 8 | 9 |
| Fabaceae | *Mimosa irrigua* | 1 | 0 | 0 | 0 | 0 | 0 | 0 | 0 | 0 |
| Fabaceae | *Mimosa lepidophora* | 0 | 0 | 0 | 0 | 0 | 0 | 0 | 8 | 0 |
| Fabaceae | *Mimosa lewisii* | 1 | 0 | 0 | 4 | 5 | 0 | 7 | 0 | 0 |
| Fabaceae | *Mimosa misera* | 0 | 0 | 0 | 0 | 5 | 0 | 7 | 8 | 0 |
| Fabaceae | *Mimosa nothopteris* | 0 | 0 | 0 | 0 | 0 | 0 | 0 | 0 | 9 |
| Fabaceae | *Mimosa ophthalmocentra* | 0 | 0 | 3 | 4 | 5 | 6 | 7 | 8 | 0 |
| Fabaceae | *Mimosa paraibana* | 0 | 2 | 0 | 0 | 0 | 6 | 7 | 8 | 0 |
| Fabaceae | *Mimosa pellita* | 0 | 2 | 0 | 0 | 0 | 0 | 0 | 8 | 0 |
| Fabaceae | *Mimosa pigra* | 0 | 0 | 0 | 4 | 5 | 6 | 0 | 0 | 9 |
| Fabaceae | *Mimosa pteridifolia* | 0 | 0 | 0 | 0 | 0 | 0 | 0 | 8 | 0 |
| Fabaceae | *Mimosa pudica* | 0 | 0 | 0 | 4 | 0 | 0 | 0 | 0 | 0 |
| Fabaceae | *Mimosa quadrivalvis* | 0 | 0 | 0 | 0 | 0 | 6 | 0 | 0 | 0 |
| Fabaceae | *Mimosa sensitiva* | 0 | 0 | 0 | 4 | 5 | 6 | 7 | 8 | 9 |
| Fabaceae | *Mimosa setosa* | 1 | 0 | 0 | 0 | 0 | 0 | 0 | 0 | 0 |
| Fabaceae | *Mimosa somnians* | 0 | 2 | 0 | 0 | 0 | 0 | 0 | 8 | 9 |
| Fabaceae | *Mimosa tenuiflora* | 0 | 0 | 3 | 4 | 5 | 6 | 7 | 8 | 9 |
| Fabaceae | *Mimosa ursina* | 1 | 0 | 0 | 0 | 0 | 6 | 7 | 0 | 0 |
| Fabaceae | *Mimosa verrucosa* | 1 | 2 | 0 | 0 | 5 | 0 | 7 | 8 | 9 |
| Fabaceae | *Mimosa xiquexiquensis* | 0 | 0 | 0 | 0 | 0 | 0 | 0 | 8 | 0 |
| Nyctaginaceae | *Mirabilis jalapa* | 0 | 0 | 0 | 0 | 5 | 0 | 0 | 0 | 0 |
| Rubiaceae | *Mitracarpus frigidus* | 0 | 0 | 0 | 0 | 5 | 0 | 7 | 0 | 0 |
| Rubiaceae | *Mitracarpus hirtus* | 0 | 0 | 3 | 0 | 0 | 0 | 0 | 0 | 0 |
| Rubiaceae | *Mitracarpus salzmannianus* | 1 | 2 | 0 | 0 | 0 | 0 | 0 | 8 | 0 |
| Fabaceae | *Moldenhawera blanchetiana* | 0 | 2 | 0 | 0 | 0 | 0 | 0 | 0 | 0 |
| Fabaceae | *Moldenhawera brasiliensis* | 0 | 2 | 0 | 0 | 0 | 0 | 0 | 0 | 0 |
| Molluginaceae | *Mollugo verticillata* | 0 | 0 | 0 | 0 | 5 | 0 | 0 | 0 | 0 |
| Rubiaceae | *Molopanthera paniculata* | 0 | 0 | 3 | 4 | 0 | 0 | 0 | 0 | 0 |
| Capparaceae | *Monilicarpa brasiliana* | 0 | 0 | 3 | 4 | 0 | 0 | 0 | 0 | 0 |
| Polygalaceae | *Monnina insignis* | 0 | 0 | 0 | 0 | 0 | 6 | 0 | 0 | 0 |
| Asteraceae | *Moquinia racemosa* | 1 | 0 | 0 | 0 | 0 | 0 | 0 | 0 | 0 |
| Asteraceae | *Moquiniastrum blanchetianum* | 1 | 2 | 0 | 0 | 0 | 0 | 0 | 0 | 0 |
| Asteraceae | *Moquiniastrum oligocephalum* | 1 | 2 | 3 | 4 | 5 | 0 | 0 | 8 | 0 |
| Asteraceae | *Moquiniastrum paniculatum* | 1 | 0 | 0 | 0 | 0 | 0 | 0 | 0 | 0 |
| Rubiaceae | *Morinda citrifolia* | 0 | 0 | 0 | 0 | 0 | 0 | 7 | 0 | 0 |
| Moringaceae | *Moringa oleifera* | 0 | 0 | 0 | 0 | 0 | 0 | 0 | 0 | 9 |
| Melastomataceae | *Mouriri arborea* | 0 | 0 | 3 | 0 | 0 | 0 | 0 | 0 | 0 |
| Melastomataceae | *Mouriri cearensis* | 0 | 2 | 0 | 0 | 0 | 0 | 0 | 8 | 9 |
| Melastomataceae | *Mouriri elliptica* | 0 | 0 | 0 | 0 | 0 | 0 | 0 | 0 | 9 |
| Melastomataceae | *Mouriri guianensis* | 0 | 2 | 0 | 0 | 0 | 0 | 0 | 0 | 9 |
| Melastomataceae | *Mouriri nigra* | 0 | 0 | 0 | 0 | 5 | 0 | 0 | 0 | 0 |
| Melastomataceae | *Mouriri pusa* | 0 | 0 | 3 | 0 | 5 | 0 | 7 | 8 | 9 |
| Fabaceae | *Muellera campestris* | 0 | 0 | 3 | 0 | 0 | 6 | 0 | 0 | 0 |
| Fabaceae | *Muellera obtusus* | 0 | 0 | 0 | 0 | 0 | 6 | 0 | 0 | 0 |
| Rutaceae | *Murraya paniculata* | 0 | 0 | 0 | 0 | 0 | 0 | 0 | 0 | 9 |
| Anacardiaceae | *Myracrodruon urundeuva* | 0 | 2 | 3 | 4 | 5 | 6 | 7 | 8 | 9 |
| Myrtaceae | *Myrceugenia alpigena* | 1 | 0 | 0 | 0 | 0 | 0 | 0 | 0 | 0 |
| Myrtaceae | *Myrcia almasensis* | 1 | 0 | 0 | 0 | 0 | 0 | 0 | 0 | 0 |
| Myrtaceae | *Myrcia amazonica* | 1 | 2 | 0 | 4 | 0 | 0 | 0 | 0 | 0 |
| Myrtaceae | *Myrcia anacardiifolia* | 1 | 0 | 0 | 0 | 0 | 0 | 0 | 0 | 0 |
| Myrtaceae | *Myrcia bergiana* | 1 | 2 | 0 | 0 | 0 | 0 | 0 | 0 | 0 |
| Myrtaceae | *Myrcia blanchetiana* | 1 | 2 | 0 | 0 | 0 | 0 | 0 | 0 | 0 |
| Myrtaceae | *Myrcia brasiliensis* | 0 | 2 | 0 | 0 | 0 | 0 | 0 | 0 | 0 |
| Myrtaceae | *Myrcia bullata* | 0 | 0 | 0 | 0 | 5 | 0 | 0 | 0 | 0 |
| Myrtaceae | *Myrcia calyptranthoides* | 0 | 2 | 0 | 0 | 0 | 0 | 0 | 0 | 0 |
| Myrtaceae | *Myrcia cymosa* | 0 | 2 | 0 | 0 | 0 | 0 | 0 | 0 | 0 |
| Myrtaceae | *Myrcia decorticans* | 0 | 2 | 0 | 0 | 5 | 0 | 0 | 0 | 0 |
| Myrtaceae | *Myrcia densa* | 1 | 0 | 0 | 0 | 0 | 0 | 0 | 0 | 0 |
| Myrtaceae | *Myrcia eriocalyx* | 1 | 0 | 0 | 0 | 0 | 0 | 0 | 0 | 0 |
| Myrtaceae | *Myrcia eximia* | 0 | 2 | 0 | 0 | 0 | 0 | 0 | 0 | 0 |
| Myrtaceae | *Myrcia felisberti* | 0 | 2 | 0 | 0 | 0 | 0 | 0 | 0 | 0 |
| Myrtaceae | *Myrcia glauca* | 0 | 0 | 0 | 0 | 0 | 0 | 0 | 0 | 9 |
| Myrtaceae | *Myrcia guianensis* | 1 | 2 | 3 | 4 | 5 | 0 | 0 | 8 | 9 |
| Myrtaceae | *Myrcia hartwegiana* | 1 | 0 | 0 | 0 | 0 | 0 | 0 | 0 | 0 |
| Myrtaceae | *Myrcia hebepetala* | 0 | 0 | 0 | 0 | 0 | 6 | 0 | 0 | 0 |
| Myrtaceae | *Myrcia hirtiflora* | 0 | 2 | 0 | 0 | 0 | 0 | 0 | 0 | 0 |
| Myrtaceae | *Myrcia jacobinensis* | 1 | 0 | 0 | 0 | 0 | 0 | 0 | 0 | 0 |
| Myrtaceae | *Myrcia laruotteana* | 0 | 0 | 0 | 0 | 5 | 0 | 0 | 0 | 0 |
| Myrtaceae | *Myrcia littoralis* | 1 | 0 | 0 | 0 | 0 | 0 | 0 | 0 | 0 |
| Myrtaceae | *Myrcia mischophylla* | 1 | 0 | 0 | 0 | 0 | 0 | 0 | 0 | 0 |
| Myrtaceae | *Myrcia multiflora* | 1 | 2 | 0 | 4 | 5 | 0 | 0 | 8 | 9 |
| Myrtaceae | *Myrcia mutabilis* | 1 | 0 | 0 | 0 | 0 | 0 | 0 | 0 | 0 |
| Myrtaceae | *Myrcia myrcioides* | 0 | 0 | 0 | 4 | 0 | 0 | 0 | 0 | 0 |
| Myrtaceae | *Myrcia oblongata* | 0 | 0 | 0 | 4 | 0 | 0 | 0 | 0 | 0 |
| Myrtaceae | *Myrcia polyantha* | 0 | 2 | 0 | 0 | 5 | 0 | 0 | 0 | 0 |
| Myrtaceae | *Myrcia pubescens* | 1 | 0 | 0 | 0 | 0 | 0 | 0 | 0 | 0 |
| Myrtaceae | *Myrcia racemosa* | 0 | 2 | 0 | 0 | 0 | 0 | 0 | 0 | 0 |
| Myrtaceae | *Myrcia reticulosa* | 1 | 0 | 0 | 0 | 0 | 0 | 0 | 0 | 0 |
| Myrtaceae | *Myrcia rosangelae* | 0 | 0 | 0 | 0 | 5 | 0 | 0 | 0 | 0 |
| Myrtaceae | *Myrcia saxatilis* | 0 | 0 | 0 | 0 | 0 | 0 | 0 | 0 | 9 |
| Myrtaceae | *Myrcia splendens* | 1 | 2 | 3 | 4 | 5 | 0 | 0 | 8 | 9 |
| Myrtaceae | *Myrcia sylvatica* | 1 | 2 | 3 | 4 | 5 | 0 | 0 | 0 | 0 |
| Myrtaceae | *Myrcia tomentosa* | 1 | 2 | 3 | 4 | 5 | 0 | 0 | 0 | 9 |
| Myrtaceae | *Myrcia undulata* | 0 | 0 | 3 | 0 | 0 | 0 | 0 | 0 | 0 |
| Myrtaceae | *Myrcia venulosa* | 1 | 0 | 0 | 0 | 0 | 0 | 0 | 0 | 0 |
| Myrtaceae | *Myrcia verrucosa* | 0 | 2 | 0 | 0 | 0 | 0 | 0 | 0 | 0 |
| Myrtaceae | *Myrcia vestita* | 1 | 2 | 0 | 0 | 0 | 0 | 0 | 0 | 0 |
| Myrtaceae | *Myrcia vittoriana* | 0 | 2 | 0 | 0 | 0 | 0 | 0 | 0 | 0 |
| Myrtaceae | *Myrcianthes cisplatensis* | 0 | 0 | 0 | 0 | 0 | 0 | 0 | 0 | 9 |
| Myrtaceae | *Myrciaria cuspidata* | 1 | 2 | 0 | 0 | 0 | 0 | 0 | 8 | 9 |
| Myrtaceae | *Myrciaria ferruginea* | 0 | 2 | 0 | 0 | 0 | 0 | 0 | 8 | 0 |
| Myrtaceae | *Myrciaria floribunda* | 1 | 2 | 3 | 0 | 5 | 0 | 7 | 0 | 0 |
| Myrtaceae | *Myrciaria guaquiea* | 1 | 0 | 0 | 0 | 0 | 0 | 0 | 0 | 0 |
| Myrtaceae | *Myrciaria pilosa* | 1 | 0 | 0 | 0 | 0 | 0 | 0 | 0 | 0 |
| Myrtaceae | *Myrciaria tenella* | 0 | 0 | 0 | 0 | 0 | 0 | 0 | 8 | 0 |
| Boraginaceae | *Myriopus rubicundus* | 0 | 0 | 0 | 0 | 5 | 6 | 0 | 0 | 0 |
| Fabaceae | *Myrocarpus fastigiatus* | 0 | 0 | 3 | 0 | 0 | 6 | 0 | 0 | 0 |
| Fabaceae | *Myroxylon balsamum* | 0 | 0 | 0 | 4 | 0 | 0 | 0 | 0 | 0 |
| Fabaceae | *Myroxylon peruiferum* | 0 | 2 | 0 | 4 | 5 | 6 | 0 | 0 | 0 |
| Primulaceae | *Myrsine coriacea* | 0 | 2 | 3 | 0 | 0 | 0 | 0 | 0 | 0 |
| Primulaceae | *Myrsine gardneriana* | 0 | 0 | 0 | 4 | 0 | 0 | 0 | 0 | 0 |
| Primulaceae | *Myrsine guianensis* | 1 | 2 | 3 | 4 | 5 | 0 | 0 | 0 | 0 |
| Primulaceae | *Myrsine monticola* | 1 | 0 | 0 | 0 | 0 | 0 | 0 | 0 | 0 |
| Primulaceae | *Myrsine umbellata* | 1 | 2 | 3 | 0 | 0 | 0 | 0 | 0 | 0 |
| Primulaceae | *Myrsine venosa* | 1 | 2 | 0 | 0 | 0 | 0 | 0 | 0 | 0 |
| Velloziaceae | *Nanuza plicata* | 0 | 0 | 0 | 4 | 0 | 0 | 0 | 8 | 0 |
| Lauraceae | *Nectandra cuspidata* | 0 | 2 | 0 | 4 | 0 | 0 | 0 | 0 | 0 |
| Lauraceae | *Nectandra membranacea* | 0 | 0 | 3 | 0 | 0 | 0 | 0 | 0 | 0 |
| Lauraceae | *Nectandra nitidula* | 0 | 0 | 3 | 0 | 5 | 6 | 0 | 0 | 0 |
| Nyctaginaceae | *Neea obovata* | 0 | 2 | 0 | 0 | 0 | 0 | 0 | 8 | 0 |
| Capparaceae | *Neocalyptrocalyx grandipetala* | 0 | 0 | 3 | 0 | 0 | 0 | 0 | 0 | 0 |
| Capparaceae | *Neocalyptrocalyx longifolium* | 0 | 0 | 3 | 4 | 5 | 6 | 7 | 0 | 9 |
| Capparaceae | *Neocalyptrocalyx nectareus* | 0 | 0 | 3 | 0 | 0 | 0 | 0 | 0 | 0 |
| Bignoniaceae | *Neojobertia candolleana* | 0 | 0 | 0 | 0 | 0 | 6 | 7 | 0 | 0 |
| Fabaceae | *Neptunia plena* | 0 | 0 | 0 | 0 | 0 | 6 | 0 | 8 | 0 |
| Apocynaceae | *Nerium oleander* | 0 | 0 | 0 | 0 | 0 | 0 | 7 | 0 | 9 |
| Solanaceae | *Nicotiana glauca* | 0 | 0 | 0 | 0 | 5 | 6 | 7 | 0 | 0 |
| Cactaceae | *Nopalea cochenillifera* | 0 | 0 | 0 | 0 | 0 | 0 | 0 | 0 | 9 |
| Marcgraviaceae | *Norantea guianensis* | 1 | 2 | 0 | 0 | 0 | 0 | 0 | 0 | 0 |
| Lamiaceae | *Ocimum gratissimum* | 0 | 2 | 0 | 0 | 0 | 0 | 0 | 0 | 0 |
| Lauraceae | *Ocotea aciphylla* | 1 | 0 | 0 | 0 | 0 | 0 | 0 | 0 | 0 |
| Lauraceae | *Ocotea bicolor* | 0 | 2 | 0 | 0 | 0 | 0 | 0 | 0 | 0 |
| Lauraceae | *Ocotea brachybotrya* | 1 | 0 | 0 | 0 | 0 | 0 | 0 | 0 | 0 |
| Lauraceae | *Ocotea bracteosa* | 0 | 0 | 0 | 4 | 0 | 0 | 0 | 0 | 9 |
| Lauraceae | *Ocotea canaliculata* | 0 | 2 | 0 | 0 | 0 | 0 | 0 | 8 | 9 |
| Lauraceae | *Ocotea cernua* | 0 | 0 | 3 | 0 | 0 | 0 | 0 | 0 | 0 |
| Lauraceae | *Ocotea complicata* | 0 | 0 | 3 | 0 | 0 | 0 | 0 | 0 | 0 |
| Lauraceae | *Ocotea corymbosa* | 0 | 0 | 3 | 0 | 0 | 0 | 0 | 0 | 0 |
| Lauraceae | *Ocotea duckei* | 0 | 2 | 3 | 4 | 5 | 0 | 0 | 8 | 0 |
| Lauraceae | *Ocotea elegans* | 0 | 2 | 0 | 0 | 0 | 0 | 0 | 0 | 0 |
| Lauraceae | *Ocotea fasciculata* | 0 | 0 | 0 | 0 | 0 | 0 | 0 | 0 | 9 |
| Lauraceae | *Ocotea gardneri* | 0 | 0 | 0 | 4 | 0 | 0 | 0 | 0 | 0 |
| Lauraceae | *Ocotea glaucina* | 1 | 0 | 0 | 0 | 0 | 0 | 0 | 0 | 0 |
| Lauraceae | *Ocotea glomerata* | 1 | 2 | 3 | 4 | 5 | 0 | 0 | 0 | 0 |
| Lauraceae | *Ocotea indecora* | 0 | 0 | 3 | 0 | 0 | 0 | 0 | 0 | 0 |
| Lauraceae | *Ocotea lancifolia* | 1 | 0 | 0 | 0 | 0 | 0 | 0 | 0 | 0 |
| Lauraceae | *Ocotea limae* | 0 | 0 | 0 | 4 | 0 | 0 | 0 | 0 | 0 |
| Lauraceae | *Ocotea longifolia* | 0 | 0 | 0 | 4 | 0 | 0 | 0 | 0 | 0 |
| Lauraceae | *Ocotea macropoda* | 0 | 0 | 0 | 4 | 0 | 0 | 0 | 0 | 0 |
| Lauraceae | *Ocotea nitida* | 0 | 2 | 3 | 0 | 0 | 0 | 0 | 8 | 0 |
| Lauraceae | *Ocotea notata* | 1 | 2 | 3 | 0 | 0 | 0 | 0 | 0 | 0 |
| Lauraceae | *Ocotea odorifera* | 0 | 0 | 0 | 4 | 0 | 0 | 0 | 0 | 0 |
| Lauraceae | *Ocotea oppositifolia* | 1 | 0 | 0 | 0 | 0 | 0 | 0 | 0 | 0 |
| Lauraceae | *Ocotea percoriacea* | 1 | 0 | 0 | 0 | 0 | 0 | 0 | 0 | 0 |
| Lauraceae | *Ocotea pomaderroides* | 1 | 0 | 0 | 0 | 0 | 0 | 0 | 0 | 0 |
| Lauraceae | *Ocotea prolifera* | 0 | 2 | 3 | 0 | 0 | 0 | 0 | 0 | 0 |
| Lauraceae | *Ocotea puberula* | 1 | 2 | 0 | 0 | 0 | 0 | 0 | 0 | 0 |
| Lauraceae | *Ocotea spixiana* | 1 | 0 | 0 | 0 | 0 | 0 | 0 | 0 | 0 |
| Lauraceae | *Ocotea tristis* | 1 | 0 | 0 | 0 | 0 | 0 | 0 | 0 | 0 |
| Lauraceae | *Ocotea vegrandis* | 1 | 0 | 0 | 0 | 0 | 0 | 0 | 0 | 0 |
| Lauraceae | *Ocotea velloziana* | 1 | 0 | 0 | 0 | 0 | 0 | 0 | 0 | 0 |
| Lauraceae | *Ocotea velutina* | 0 | 0 | 3 | 0 | 0 | 0 | 0 | 0 | 0 |
| Lauraceae | *Ocotea xanthocalyx* | 0 | 0 | 0 | 4 | 5 | 0 | 0 | 0 | 0 |
| Apocynaceae | *Odontadenia lutea* | 0 | 0 | 0 | 0 | 0 | 0 | 0 | 8 | 0 |
| Lamiaceae | *Oocephalus crassifolius* | 1 | 0 | 0 | 0 | 0 | 0 | 0 | 0 | 0 |
| Lamiaceae | *Oocephalus hagei* | 1 | 0 | 0 | 0 | 0 | 0 | 0 | 0 | 0 |
| Lamiaceae | *Oocephalus nubicola* | 1 | 0 | 0 | 0 | 0 | 0 | 0 | 0 | 0 |
| Convolvulaceae | *Operculina macrocarpa* | 0 | 0 | 0 | 0 | 5 | 0 | 0 | 0 | 0 |
| Cactaceae | *Opuntia dillenii* | 0 | 0 | 0 | 0 | 0 | 0 | 0 | 0 | 9 |
| Cactaceae | *Opuntia ficus.indica* | 0 | 0 | 0 | 0 | 0 | 0 | 7 | 0 | 0 |
| Araliaceae | *Oreopanax capitatus* | 0 | 2 | 0 | 0 | 0 | 0 | 0 | 0 | 0 |
| Fabaceae | *Ormosia arborea* | 0 | 2 | 0 | 0 | 0 | 0 | 0 | 0 | 0 |
| Fabaceae | *Ormosia fastigiata* | 0 | 0 | 0 | 4 | 0 | 0 | 0 | 8 | 9 |
| Melastomataceae | *Ossaea amygdaloides* | 0 | 2 | 0 | 0 | 0 | 0 | 0 | 0 | 0 |
| Melastomataceae | *Ossaea congestiflora* | 1 | 0 | 0 | 0 | 0 | 0 | 0 | 0 | 0 |
| Melastomataceae | *Ossaea consimilis* | 0 | 0 | 3 | 0 | 0 | 0 | 0 | 0 | 0 |
| Ochnaceae | *Ouratea blanchetiana* | 0 | 0 | 0 | 4 | 0 | 0 | 0 | 0 | 0 |
| Ochnaceae | *Ouratea castaneifolia* | 1 | 2 | 3 | 0 | 0 | 0 | 0 | 8 | 9 |
| Ochnaceae | *Ouratea cearensis* | 0 | 0 | 0 | 0 | 0 | 0 | 0 | 0 | 9 |
| Ochnaceae | *Ouratea crassifolia* | 0 | 0 | 0 | 4 | 0 | 0 | 0 | 0 | 0 |
| Ochnaceae | *Ouratea cuspidata* | 0 | 0 | 0 | 0 | 5 | 0 | 0 | 0 | 0 |
| Ochnaceae | *Ouratea fieldingiana* | 0 | 2 | 0 | 0 | 0 | 0 | 0 | 8 | 0 |
| Ochnaceae | *Ouratea floribunda* | 1 | 0 | 0 | 0 | 0 | 0 | 0 | 0 | 0 |
| Ochnaceae | *Ouratea glaucescens* | 0 | 0 | 0 | 0 | 0 | 0 | 0 | 8 | 0 |
| Ochnaceae | *Ouratea hexasperma* | 0 | 2 | 0 | 0 | 5 | 0 | 0 | 0 | 9 |
| Ochnaceae | *Ouratea hilaireana* | 0 | 0 | 0 | 0 | 0 | 0 | 0 | 0 | 9 |
| Ochnaceae | *Ouratea parviflora* | 1 | 2 | 0 | 0 | 0 | 0 | 0 | 0 | 0 |
| Ochnaceae | *Ouratea parvifolia* | 0 | 2 | 0 | 4 | 5 | 0 | 0 | 0 | 9 |
| Ochnaceae | *Ouratea salicifolia* | 0 | 2 | 0 | 0 | 0 | 0 | 0 | 0 | 0 |
| Ochnaceae | *Ouratea semiserrata* | 1 | 0 | 0 | 0 | 0 | 0 | 0 | 0 | 0 |
| Ochnaceae | *Ouratea subscandens* | 0 | 0 | 0 | 4 | 0 | 0 | 0 | 0 | 0 |
| Ochnaceae | *Ouratea xerophila* | 0 | 0 | 0 | 0 | 0 | 0 | 0 | 0 | 9 |
| Oxalidaceae | *Oxalis divaricata* | 0 | 0 | 0 | 0 | 0 | 0 | 0 | 8 | 0 |
| Oxalidaceae | *Oxalis frutescens* | 0 | 0 | 0 | 0 | 0 | 6 | 0 | 0 | 0 |
| Oxalidaceae | *Oxalis glaucescens* | 0 | 0 | 0 | 4 | 0 | 0 | 0 | 0 | 0 |
| Oxalidaceae | *Oxalis psoraleoides* | 0 | 0 | 0 | 0 | 5 | 6 | 7 | 0 | 0 |
| Annonaceae | *Oxandra reticulata* | 0 | 0 | 0 | 0 | 5 | 0 | 0 | 0 | 0 |
| Annonaceae | *Oxandra sessiliflora* | 0 | 2 | 0 | 0 | 0 | 0 | 0 | 0 | 9 |
| Malvaceae | *Pachira aquatica* | 0 | 0 | 0 | 0 | 0 | 0 | 0 | 0 | 9 |
| Malvaceae | *Pachira endecaphylla* | 0 | 0 | 0 | 0 | 0 | 6 | 0 | 8 | 0 |
| Malvaceae | *Pachira retusa* | 0 | 0 | 0 | 0 | 0 | 0 | 0 | 8 | 0 |
| Malvaceae | *Pachira stenopetala* | 0 | 0 | 0 | 0 | 0 | 0 | 0 | 8 | 0 |
| Eriocaulaceae | *Paepalanthus carvalhoi* | 1 | 0 | 0 | 0 | 0 | 0 | 0 | 0 | 0 |
| Eriocaulaceae | *Paepalanthus pulchellus* | 1 | 0 | 0 | 0 | 0 | 0 | 0 | 0 | 0 |
| Eriocaulaceae | *Paepalanthus pulvinatus* | 1 | 0 | 0 | 0 | 0 | 0 | 0 | 0 | 0 |
| Eriocaulaceae | *Paepalanthus sessiliflorus* | 1 | 0 | 0 | 0 | 0 | 0 | 0 | 0 | 0 |
| Eriocaulaceae | *Paepalanthus spathulatus* | 1 | 0 | 0 | 0 | 0 | 0 | 0 | 0 | 0 |
| Eriocaulaceae | *Paepalanthus tortilis* | 1 | 0 | 0 | 0 | 0 | 0 | 0 | 0 | 0 |
| Eriocaulaceae | *Paepalanthus trichophyllus* | 1 | 0 | 0 | 0 | 0 | 0 | 0 | 0 | 0 |
| Gesneriaceae | *Paliavana tenuiflora* | 1 | 0 | 0 | 4 | 0 | 0 | 0 | 0 | 0 |
| Rubiaceae | *Palicourea blanchetiana* | 0 | 2 | 0 | 0 | 0 | 0 | 0 | 0 | 0 |
| Rubiaceae | *Palicourea crocea* | 1 | 0 | 3 | 0 | 0 | 0 | 0 | 0 | 0 |
| Rubiaceae | *Palicourea guianensis* | 0 | 2 | 3 | 0 | 0 | 0 | 0 | 0 | 0 |
| Rubiaceae | *Palicourea marcgravii* | 1 | 2 | 0 | 0 | 0 | 0 | 0 | 0 | 0 |
| Rubiaceae | *Palicourea rigida* | 1 | 2 | 0 | 0 | 0 | 0 | 0 | 0 | 0 |
| Rubiaceae | *Palicourea sclerophylla* | 1 | 0 | 0 | 0 | 0 | 0 | 0 | 0 | 0 |
| Rubiaceae | *Palicourea vacillans* | 1 | 0 | 0 | 0 | 0 | 0 | 0 | 0 | 0 |
| Proteaceae | *Panopsis rubescens* | 0 | 2 | 0 | 0 | 0 | 0 | 0 | 0 | 0 |
| Asteraceae | *Paralychnophora bicolor* | 1 | 0 | 0 | 0 | 0 | 0 | 0 | 0 | 0 |
| Asteraceae | *Paralychnophora harleyi* | 1 | 0 | 0 | 0 | 0 | 0 | 0 | 0 | 0 |
| Asteraceae | *Paralychnophora reflexoauriculata* | 1 | 0 | 3 | 4 | 0 | 0 | 0 | 0 | 0 |
| Fabaceae | *Parapiptadenia bahiana* | 0 | 0 | 0 | 4 | 0 | 0 | 0 | 0 | 0 |
| Fabaceae | *Parapiptadenia blanchetii* | 0 | 0 | 0 | 4 | 5 | 0 | 0 | 0 | 9 |
| Fabaceae | *Parapiptadenia zehntneri* | 0 | 0 | 3 | 4 | 5 | 6 | 7 | 0 | 0 |
| Chrysobalanaceae | *Parinari campestris* | 0 | 0 | 0 | 0 | 0 | 0 | 0 | 8 | 9 |
| Fabaceae | *Parkia platycephala* | 0 | 2 | 3 | 0 | 5 | 0 | 7 | 8 | 9 |
| Fabaceae | *Parkinsonia aculeata* | 0 | 0 | 0 | 0 | 5 | 6 | 7 | 0 | 9 |
| Passifloraceae | *Passiflora cincinnata* | 0 | 0 | 0 | 0 | 0 | 6 | 7 | 0 | 0 |
| Passifloraceae | *Passiflora foetida* | 0 | 0 | 0 | 0 | 5 | 0 | 0 | 0 | 0 |
| Passifloraceae | *Passiflora luetzelburgii* | 0 | 0 | 0 | 0 | 5 | 0 | 0 | 0 | 0 |
| Sapindaceae | *Paullinia elegans* | 0 | 0 | 0 | 0 | 0 | 0 | 0 | 0 | 9 |
| Sapindaceae | *Paullinia pinnata* | 1 | 0 | 3 | 0 | 5 | 0 | 0 | 0 | 0 |
| Sapindaceae | *Paullinia racemosa* | 0 | 0 | 0 | 0 | 5 | 0 | 0 | 0 | 0 |
| Malvaceae | *Pavonia blanchetiana* | 0 | 0 | 0 | 4 | 5 | 0 | 7 | 8 | 0 |
| Malvaceae | *Pavonia cancellata* | 1 | 2 | 0 | 4 | 0 | 6 | 0 | 0 | 0 |
| Malvaceae | *Pavonia candida* | 1 | 0 | 0 | 0 | 0 | 0 | 0 | 0 | 0 |
| Malvaceae | *Pavonia glazioviana* | 1 | 0 | 0 | 4 | 5 | 6 | 7 | 8 | 9 |
| Malvaceae | *Pavonia harleyi* | 1 | 0 | 0 | 0 | 0 | 0 | 0 | 0 | 0 |
| Malvaceae | *Pavonia humifusa* | 0 | 0 | 0 | 0 | 5 | 0 | 7 | 0 | 0 |
| Malvaceae | *Pavonia luetzelburgii* | 1 | 0 | 0 | 0 | 0 | 0 | 0 | 0 | 0 |
| Malvaceae | *Pavonia macrostyla* | 1 | 0 | 0 | 0 | 0 | 0 | 0 | 0 | 0 |
| Malvaceae | *Pavonia malacophylla* | 1 | 2 | 0 | 0 | 0 | 0 | 0 | 0 | 0 |
| Malvaceae | *Pavonia sidifolia* | 0 | 0 | 0 | 0 | 0 | 0 | 0 | 8 | 0 |
| Malvaceae | *Pavonia varians* | 0 | 0 | 0 | 0 | 5 | 0 | 0 | 8 | 0 |
| Malvaceae | *Pavonia vinosa* | 0 | 2 | 0 | 0 | 0 | 0 | 0 | 8 | 0 |
| Violaceae | *Paypayrola blanchetiana* | 0 | 2 | 0 | 0 | 0 | 0 | 0 | 0 | 0 |
| Malpighiaceae | *Peixotoa hispidula* | 0 | 0 | 0 | 0 | 0 | 0 | 7 | 0 | 0 |
| Malpighiaceae | *Peixotoa jussieuana* | 1 | 0 | 0 | 0 | 0 | 0 | 7 | 0 | 9 |
| Malvaceae | *Peltaea obsita* | 1 | 0 | 0 | 0 | 0 | 0 | 0 | 0 | 0 |
| Malvaceae | *Peltaea polymorpha* | 1 | 0 | 0 | 0 | 0 | 0 | 0 | 0 | 0 |
| Malvaceae | *Peltaea trinervis* | 0 | 2 | 0 | 0 | 0 | 0 | 0 | 8 | 0 |
| Fabaceae | *Peltogyne chrysopis* | 0 | 2 | 0 | 0 | 0 | 0 | 0 | 0 | 0 |
| Fabaceae | *Peltogyne confertiflora* | 0 | 0 | 0 | 0 | 0 | 6 | 7 | 8 | 9 |
| Fabaceae | *Peltogyne discolor* | 0 | 2 | 0 | 0 | 0 | 0 | 0 | 0 | 0 |
| Fabaceae | *Peltogyne parvifolia* | 0 | 0 | 0 | 0 | 0 | 0 | 0 | 8 | 0 |
| Fabaceae | *Peltogyne pauciflora* | 0 | 0 | 0 | 0 | 5 | 0 | 7 | 8 | 0 |
| Fabaceae | *Peltophorum dubium* | 1 | 0 | 3 | 4 | 5 | 6 | 0 | 0 | 0 |
| Piperaceae | *Peperomia diamantinensis* | 1 | 0 | 0 | 0 | 0 | 0 | 0 | 0 | 0 |
| Piperaceae | *Peperomia obtusifolia* | 1 | 0 | 0 | 0 | 0 | 0 | 0 | 0 | 0 |
| Piperaceae | *Peperomia tetraphylla* | 1 | 0 | 0 | 0 | 0 | 0 | 0 | 0 | 0 |
| Apocynaceae | *Peplonia bradeana* | 0 | 0 | 3 | 0 | 0 | 0 | 0 | 0 | 0 |
| Euphorbiaceae | *Pera glabrata* | 0 | 2 | 3 | 0 | 5 | 0 | 0 | 8 | 0 |
| Euphorbiaceae | *Pera heteranthera* | 0 | 2 | 0 | 0 | 0 | 0 | 0 | 0 | 0 |
| Rubiaceae | *Perama harleyi* | 1 | 0 | 0 | 0 | 0 | 0 | 0 | 0 | 0 |
| Cactaceae | *Pereskia bahiensis* | 0 | 0 | 0 | 0 | 0 | 6 | 0 | 8 | 0 |
| Cactaceae | *Pereskia grandiflora* | 0 | 0 | 0 | 0 | 0 | 6 | 0 | 0 | 0 |
| Cactaceae | *Pereskia grandifolia* | 0 | 0 | 0 | 0 | 0 | 0 | 0 | 0 | 9 |
| Fabaceae | *Periandra mediterranea* | 1 | 2 | 0 | 4 | 5 | 0 | 7 | 8 | 0 |
| Lauraceae | *Persea americana* | 0 | 0 | 0 | 0 | 0 | 6 | 0 | 0 | 0 |
| Lauraceae | *Persea aurata* | 1 | 0 | 0 | 0 | 0 | 0 | 0 | 0 | 0 |
| Lauraceae | *Persea major* | 1 | 0 | 0 | 0 | 0 | 0 | 0 | 0 | 0 |
| Araceae | *Philodendron pachyphyllum* | 1 | 0 | 0 | 0 | 0 | 0 | 0 | 0 | 0 |
| Euphorbiaceae | *Philyra brasiliensis* | 0 | 0 | 0 | 4 | 0 | 0 | 0 | 0 | 0 |
| Santalaceae | *Phoradendron liga* | 0 | 2 | 0 | 0 | 0 | 0 | 0 | 0 | 0 |
| Phyllanthaceae | *Phyllanthus acuminatus* | 0 | 0 | 0 | 4 | 0 | 0 | 7 | 0 | 0 |
| Phyllanthaceae | *Phyllanthus angustissimus* | 1 | 0 | 0 | 0 | 0 | 0 | 0 | 0 | 0 |
| Phyllanthaceae | *Phyllanthus brasiliensis* | 0 | 0 | 0 | 0 | 0 | 0 | 7 | 0 | 0 |
| Phyllanthaceae | *Phyllanthus chacoensis* | 0 | 0 | 0 | 0 | 5 | 0 | 0 | 0 | 0 |
| Phyllanthaceae | *Phyllanthus flagelliformis* | 1 | 2 | 0 | 0 | 0 | 0 | 0 | 0 | 0 |
| Phyllanthaceae | *Phyllanthus heteradenius* | 0 | 0 | 0 | 0 | 5 | 0 | 0 | 0 | 0 |
| Phyllanthaceae | *Phyllanthus klotzschianus* | 1 | 0 | 0 | 0 | 0 | 0 | 0 | 0 | 0 |
| Phyllanthaceae | *Phyllanthus niruri* | 0 | 0 | 0 | 0 | 0 | 0 | 7 | 8 | 0 |
| Phyllanthaceae | *Phyllanthus ternellus* | 0 | 0 | 0 | 0 | 5 | 0 | 0 | 0 | 0 |
| Ulmaceae | *Phyllostylon brasiliense* | 0 | 0 | 0 | 0 | 0 | 6 | 0 | 0 | 0 |
| Solanaceae | *Physalis alkekengi* | 0 | 0 | 0 | 4 | 0 | 0 | 0 | 0 | 0 |
| Solanaceae | *Physalis angulata* | 0 | 0 | 0 | 0 | 0 | 0 | 7 | 0 | 0 |
| Solanaceae | *Physalis pubescens* | 0 | 0 | 0 | 0 | 5 | 0 | 0 | 0 | 0 |
| Orobanchaceae | *Physocalyx scaberrimus* | 1 | 0 | 0 | 0 | 0 | 0 | 0 | 0 | 0 |
| Phytolaccaceae | *Phytolacca thyrsiflora* | 1 | 0 | 0 | 0 | 0 | 0 | 0 | 0 | 0 |
| Picramniaceae | *Picramnia andrade.limae* | 0 | 0 | 0 | 4 | 0 | 0 | 0 | 0 | 0 |
| Picramniaceae | *Picramnia bahiensis* | 0 | 0 | 0 | 0 | 5 | 0 | 0 | 0 | 0 |
| Picramniaceae | *Picramnia gardneri* | 0 | 2 | 0 | 0 | 0 | 0 | 0 | 0 | 0 |
| Picramniaceae | *Picramnia ramiflora* | 0 | 2 | 0 | 0 | 0 | 0 | 0 | 0 | 0 |
| Simaroubaceae | *Picrasma crenata* | 0 | 0 | 3 | 4 | 0 | 0 | 0 | 0 | 0 |
| Urticaceae | *Pilea hyalina* | 0 | 0 | 0 | 4 | 0 | 0 | 0 | 0 | 0 |
| Rutaceae | *Pilocarpus jaborandi* | 0 | 0 | 0 | 0 | 0 | 0 | 0 | 8 | 9 |
| Rutaceae | *Pilocarpus spicatus* | 0 | 0 | 0 | 0 | 0 | 0 | 7 | 0 | 9 |
| Rutaceae | *Pilocarpus trachylophus* | 0 | 0 | 0 | 0 | 0 | 0 | 0 | 8 | 0 |
| Cactaceae | *Pilosocereus arrabidae* | 0 | 0 | 0 | 0 | 0 | 0 | 0 | 0 | 9 |
| Cactaceae | *Pilosocereus catingicola* | 0 | 0 | 0 | 0 | 5 | 0 | 0 | 8 | 0 |
| Cactaceae | *Pilosocereus chrysostele* | 0 | 0 | 0 | 0 | 0 | 6 | 7 | 0 | 0 |
| Cactaceae | *Pilosocereus flavipulvinatus* | 0 | 0 | 0 | 0 | 0 | 0 | 0 | 0 | 9 |
| Cactaceae | *Pilosocereus glaucescens* | 0 | 0 | 0 | 0 | 0 | 6 | 0 | 0 | 0 |
| Cactaceae | *Pilosocereus gounellei* | 0 | 0 | 0 | 4 | 5 | 6 | 7 | 8 | 9 |
| Cactaceae | *Pilosocereus magnificus* | 0 | 0 | 0 | 0 | 0 | 6 | 0 | 0 | 0 |
| Cactaceae | *Pilosocereus pachycladus* | 0 | 0 | 0 | 4 | 5 | 6 | 7 | 0 | 0 |
| Cactaceae | *Pilosocereus pentaedrophorus* | 0 | 0 | 0 | 4 | 0 | 0 | 0 | 0 | 9 |
| Cactaceae | *Pilosocereus piauhyensis* | 0 | 0 | 0 | 0 | 5 | 6 | 7 | 0 | 0 |
| Cactaceae | *Pilosocereus tuberculatus* | 0 | 0 | 0 | 0 | 5 | 6 | 7 | 8 | 0 |
| Piperaceae | *Piper aduncum* | 0 | 0 | 0 | 4 | 0 | 0 | 0 | 0 | 0 |
| Piperaceae | *Piper amalago* | 1 | 2 | 0 | 0 | 0 | 0 | 0 | 0 | 0 |
| Piperaceae | *Piper arboreum* | 0 | 2 | 3 | 4 | 0 | 0 | 0 | 0 | 0 |
| Piperaceae | *Piper boucheanum* | 0 | 2 | 0 | 0 | 0 | 0 | 0 | 0 | 0 |
| Piperaceae | *Piper caldense* | 0 | 2 | 0 | 0 | 0 | 0 | 0 | 0 | 0 |
| Piperaceae | *Piper dilatatum* | 0 | 2 | 0 | 0 | 0 | 0 | 0 | 0 | 0 |
| Piperaceae | *Piper hispidum* | 0 | 2 | 0 | 0 | 0 | 0 | 0 | 0 | 0 |
| Piperaceae | *Piper lhotzkyanum* | 1 | 0 | 0 | 0 | 0 | 0 | 0 | 0 | 0 |
| Piperaceae | *Piper marginatum* | 0 | 2 | 0 | 0 | 0 | 0 | 0 | 0 | 0 |
| Piperaceae | *Piper rhododendrifolium* | 0 | 2 | 0 | 0 | 0 | 0 | 0 | 0 | 0 |
| Piperaceae | *Piper richardiifolium* | 0 | 0 | 0 | 0 | 0 | 0 | 0 | 8 | 0 |
| Piperaceae | *Piper tectoniifolium* | 1 | 0 | 0 | 0 | 0 | 0 | 0 | 0 | 0 |
| Piperaceae | *Piper tuberculatum* | 0 | 2 | 0 | 0 | 0 | 0 | 0 | 0 | 9 |
| Fabaceae | *Piptadenia gonoacantha* | 0 | 0 | 0 | 0 | 0 | 6 | 0 | 0 | 0 |
| Fabaceae | *Piptadenia obliqua* | 0 | 0 | 3 | 4 | 5 | 6 | 7 | 8 | 9 |
| Fabaceae | *Piptadenia stipulacea* | 0 | 2 | 0 | 4 | 5 | 6 | 7 | 8 | 9 |
| Fabaceae | *Piptadenia viridiflora* | 0 | 0 | 3 | 4 | 5 | 6 | 7 | 0 | 0 |
| Asteraceae | *Piptocarpha leprosa* | 0 | 0 | 3 | 0 | 0 | 0 | 0 | 0 | 0 |
| Asteraceae | *Piptocarpha rotundifolia* | 0 | 0 | 0 | 0 | 5 | 0 | 0 | 0 | 0 |
| Passifloraceae | *Piraqueta sidifolia* | 0 | 0 | 0 | 0 | 0 | 0 | 7 | 0 | 0 |
| Passifloraceae | *Piriqueta cistoides* | 0 | 0 | 0 | 0 | 0 | 0 | 7 | 0 | 0 |
| Passifloraceae | *Piriqueta dentata* | 1 | 0 | 0 | 0 | 0 | 0 | 0 | 0 | 0 |
| Passifloraceae | *Piriqueta duarteana* | 0 | 0 | 0 | 4 | 5 | 0 | 7 | 8 | 0 |
| Passifloraceae | *Piriqueta flammea* | 1 | 0 | 0 | 0 | 0 | 0 | 0 | 0 | 0 |
| Passifloraceae | *Piriqueta guianensis* | 0 | 0 | 0 | 0 | 5 | 0 | 0 | 0 | 0 |
| Passifloraceae | *Piriqueta sarae* | 1 | 0 | 0 | 0 | 0 | 0 | 0 | 0 | 0 |
| Passifloraceae | *Piriqueta sidifolia* | 0 | 0 | 0 | 0 | 0 | 0 | 0 | 8 | 0 |
| Fabaceae | *Pithecellobium diversifolium* | 0 | 2 | 0 | 0 | 5 | 6 | 7 | 0 | 0 |
| Fabaceae | *Pithecellobium dulce* | 0 | 0 | 0 | 0 | 5 | 6 | 7 | 0 | 9 |
| Fabaceae | *Pithecellobium roseum* | 0 | 0 | 0 | 0 | 5 | 6 | 0 | 0 | 0 |
| Asteraceae | *Pithecoseris pacourinoides* | 1 | 0 | 0 | 0 | 0 | 0 | 0 | 0 | 0 |
| Fabaceae | *Pityrocarpa moniliformis* | 1 | 2 | 3 | 0 | 5 | 6 | 7 | 8 | 9 |
| Fabaceae | *Plathymenia reticulata* | 1 | 2 | 3 | 0 | 5 | 0 | 0 | 8 | 9 |
| Fabaceae | *Platymiscium blanchetii* | 0 | 0 | 3 | 0 | 0 | 6 | 7 | 0 | 0 |
| Fabaceae | *Platymiscium floribundum* | 1 | 2 | 3 | 4 | 5 | 6 | 7 | 8 | 0 |
| Fabaceae | *Platypodium elegans* | 0 | 0 | 3 | 4 | 0 | 0 | 7 | 8 | 9 |
| Celastraceae | *Plenckia populnea* | 1 | 0 | 0 | 0 | 0 | 0 | 0 | 0 | 0 |
| Lythraceae | *Pleurophora anomala* | 0 | 0 | 0 | 0 | 0 | 6 | 7 | 0 | 9 |
| Myrtaceae | *Plinia cauliflora* | 0 | 0 | 3 | 4 | 5 | 0 | 0 | 0 | 0 |
| Plumbaginaceae | *Plumbago scandens* | 0 | 0 | 0 | 0 | 5 | 6 | 7 | 0 | 0 |
| Apocynaceae | *Plumeria rubra* | 0 | 0 | 0 | 0 | 0 | 6 | 0 | 0 | 9 |
| Podocarpaceae | *Podocarpus lambertii* | 1 | 0 | 0 | 0 | 0 | 0 | 0 | 0 | 0 |
| Podocarpaceae | *Podocarpus sellowii* | 1 | 0 | 0 | 0 | 0 | 0 | 0 | 0 | 0 |
| Fabaceae | *Poecilanthe falcata* | 0 | 0 | 3 | 4 | 5 | 0 | 0 | 0 | 0 |
| Fabaceae | *Poecilanthe grandiflora* | 0 | 0 | 0 | 0 | 0 | 0 | 7 | 0 | 0 |
| Fabaceae | *Poecilanthe subcordata* | 0 | 0 | 0 | 0 | 0 | 6 | 0 | 0 | 0 |
| Fabaceae | *Poecilanthe ulei* | 0 | 0 | 3 | 4 | 5 | 0 | 7 | 0 | 0 |
| Fabaceae | *Poeppigia procera* | 0 | 2 | 3 | 0 | 5 | 6 | 7 | 8 | 9 |
| Euphorbiaceae | *Pogonophora schomburgkiana* | 1 | 2 | 3 | 4 | 5 | 0 | 0 | 0 | 0 |
| Fabaceae | *Poincianella bracteosa* | 0 | 0 | 0 | 0 | 5 | 6 | 7 | 8 | 9 |
| Fabaceae | *Poincianella gardneriana* | 0 | 0 | 0 | 0 | 0 | 6 | 7 | 0 | 0 |
| Fabaceae | *Poincianella laxiflora* | 0 | 0 | 0 | 0 | 0 | 6 | 0 | 0 | 0 |
| Fabaceae | *Poincianella microphylla* | 0 | 0 | 3 | 4 | 5 | 6 | 7 | 0 | 0 |
| Fabaceae | *Poincianella pluviosa* | 0 | 0 | 0 | 0 | 0 | 6 | 0 | 0 | 0 |
| Fabaceae | *Poincianella pyramidalis* | 0 | 0 | 0 | 4 | 5 | 6 | 7 | 8 | 9 |
| Fabaceae | *Poiretia bahiana* | 1 | 0 | 0 | 0 | 0 | 0 | 0 | 0 | 0 |
| Polygalaceae | *Polygala boliviensis* | 0 | 2 | 0 | 0 | 0 | 0 | 0 | 0 | 0 |
| Polygalaceae | *Polygala cuspidata* | 1 | 0 | 0 | 0 | 0 | 0 | 0 | 0 | 0 |
| Polygalaceae | *Polygala gracilis* | 0 | 0 | 0 | 0 | 0 | 0 | 7 | 0 | 0 |
| Polygalaceae | *Polygala paniculata* | 0 | 2 | 0 | 0 | 0 | 0 | 0 | 0 | 0 |
| Polygalaceae | *Polygala poaya* | 1 | 0 | 0 | 0 | 0 | 0 | 0 | 0 | 0 |
| Polygalaceae | *Polygala sincorensis* | 1 | 0 | 0 | 0 | 0 | 0 | 0 | 0 | 0 |
| Polygalaceae | *Polygala trichosperma* | 1 | 0 | 0 | 0 | 0 | 0 | 0 | 8 | 0 |
| Polygonaceae | *Polygonum hydropiperoides* | 1 | 0 | 0 | 0 | 0 | 0 | 0 | 0 | 0 |
| Violaceae | *Pombalia glauca* | 0 | 0 | 0 | 4 | 0 | 0 | 0 | 0 | 0 |
| Portulacaceae | *Portulaca elatior* | 0 | 0 | 0 | 0 | 5 | 0 | 0 | 0 | 0 |
| Portulacaceae | *Portulaca hirsutissima* | 0 | 0 | 0 | 0 | 5 | 0 | 7 | 0 | 0 |
| Portulacaceae | *Portulaca mucronata* | 0 | 0 | 0 | 0 | 0 | 0 | 7 | 0 | 0 |
| Portulacaceae | *Portulaca oleracea* | 0 | 0 | 0 | 0 | 5 | 0 | 0 | 0 | 0 |
| Portulacaceae | *Portulaca umbraticola* | 0 | 0 | 0 | 0 | 0 | 0 | 7 | 0 | 0 |
| Rubiaceae | *Posoqueria latifolia* | 1 | 2 | 0 | 0 | 0 | 0 | 0 | 0 | 0 |
| Sapotaceae | *Pouteria andarahiensis* | 1 | 0 | 0 | 0 | 0 | 0 | 0 | 0 | 0 |
| Sapotaceae | *Pouteria caimito* | 1 | 0 | 0 | 0 | 0 | 0 | 0 | 0 | 0 |
| Sapotaceae | *Pouteria furcata* | 0 | 0 | 0 | 0 | 0 | 0 | 0 | 8 | 0 |
| Sapotaceae | *Pouteria gardneri* | 0 | 0 | 3 | 4 | 5 | 0 | 0 | 0 | 0 |
| Sapotaceae | *Pouteria gardneriana* | 0 | 0 | 3 | 0 | 5 | 6 | 7 | 8 | 9 |
| Sapotaceae | *Pouteria glomerata* | 0 | 2 | 0 | 0 | 0 | 0 | 0 | 0 | 0 |
| Sapotaceae | *Pouteria grandiflora* | 0 | 2 | 0 | 0 | 5 | 0 | 0 | 0 | 0 |
| Sapotaceae | *Pouteria grandis* | 0 | 2 | 0 | 0 | 0 | 0 | 0 | 0 | 0 |
| Sapotaceae | *Pouteria macrophylla* | 0 | 2 | 0 | 0 | 0 | 0 | 7 | 0 | 0 |
| Sapotaceae | *Pouteria procera* | 0 | 0 | 3 | 0 | 0 | 0 | 0 | 0 | 0 |
| Sapotaceae | *Pouteria ramiflora* | 1 | 2 | 3 | 0 | 5 | 0 | 7 | 8 | 9 |
| Sapotaceae | *Pouteria reticulata* | 1 | 0 | 0 | 0 | 0 | 0 | 0 | 0 | 9 |
| Sapotaceae | *Pouteria subsessilifolia* | 1 | 0 | 0 | 0 | 0 | 0 | 0 | 0 | 0 |
| Sapotaceae | *Pouteria torta* | 1 | 2 | 3 | 0 | 0 | 0 | 0 | 0 | 0 |
| Sapotaceae | *Pouteria venosa* | 0 | 0 | 3 | 0 | 0 | 0 | 0 | 0 | 0 |
| Gentianaceae | *Prepusa montana* | 1 | 0 | 0 | 0 | 0 | 0 | 0 | 0 | 0 |
| Celastraceae | *Pristimera sclerophylla* | 0 | 0 | 0 | 0 | 0 | 0 | 0 | 8 | 0 |
| Verbenaceae | *Priva bahiensis* | 1 | 0 | 0 | 0 | 0 | 0 | 0 | 0 | 0 |
| Salicaceae | *Prockia crucis* | 0 | 2 | 3 | 4 | 5 | 6 | 7 | 8 | 0 |
| Fabaceae | *Prosopis juliflora* | 0 | 0 | 0 | 0 | 0 | 6 | 0 | 0 | 0 |
| Fabaceae | *Prosopis pallida* | 0 | 0 | 0 | 0 | 5 | 6 | 7 | 8 | 0 |
| Burseraceae | *Protium aracouchini* | 0 | 2 | 0 | 0 | 0 | 0 | 0 | 0 | 0 |
| Burseraceae | *Protium bahianum* | 0 | 2 | 3 | 0 | 0 | 0 | 0 | 0 | 0 |
| Burseraceae | *Protium heptaphyllum* | 1 | 2 | 3 | 4 | 5 | 0 | 0 | 8 | 9 |
| Burseraceae | *Protium tenuifolium* | 0 | 2 | 0 | 0 | 0 | 0 | 0 | 0 | 0 |
| Burseraceae | *Protium warmingianum* | 0 | 0 | 3 | 0 | 0 | 0 | 0 | 0 | 0 |
| Rosaceae | *Prunus myrtifolia* | 1 | 2 | 0 | 0 | 0 | 0 | 0 | 0 | 0 |
| Acanthaceae | *Pseuderanthemum carruthersii* | 0 | 0 | 0 | 0 | 0 | 0 | 7 | 0 | 0 |
| Malvaceae | *Pseudobombax campestre* | 1 | 0 | 0 | 0 | 0 | 0 | 0 | 0 | 0 |
| Malvaceae | *Pseudobombax grandiflorum* | 0 | 0 | 0 | 4 | 0 | 6 | 0 | 0 | 0 |
| Malvaceae | *Pseudobombax marginatum* | 0 | 0 | 3 | 4 | 5 | 6 | 7 | 0 | 9 |
| Malvaceae | *Pseudobombax parvifolium* | 0 | 0 | 0 | 0 | 5 | 0 | 0 | 0 | 0 |
| Malvaceae | *Pseudobombax simplicifolium* | 0 | 0 | 3 | 0 | 5 | 6 | 7 | 0 | 0 |
| Malvaceae | *Pseudobombax tomentosum* | 0 | 0 | 3 | 0 | 0 | 0 | 0 | 0 | 0 |
| Asteraceae | *Pseudobrickellia angustissima* | 1 | 0 | 0 | 0 | 0 | 0 | 0 | 0 | 0 |
| Asteraceae | *Pseudobrickellia brasiliensis* | 1 | 0 | 0 | 0 | 0 | 0 | 0 | 0 | 0 |
| Fabaceae | *Pseudopiptadenia bahiana* | 0 | 0 | 3 | 0 | 0 | 0 | 0 | 0 | 0 |
| Fabaceae | *Pseudopiptadenia brenanii* | 0 | 0 | 0 | 0 | 0 | 0 | 7 | 0 | 0 |
| Fabaceae | *Pseudopiptadenia contorta* | 0 | 0 | 3 | 0 | 0 | 0 | 0 | 0 | 0 |
| Asteraceae | *Pseudostifftia kingii* | 1 | 0 | 0 | 0 | 0 | 0 | 0 | 0 | 0 |
| Myrtaceae | *Psidium acutangulum* | 0 | 0 | 0 | 0 | 5 | 0 | 0 | 0 | 0 |
| Myrtaceae | *Psidium appendiculatum* | 1 | 0 | 0 | 0 | 5 | 0 | 0 | 8 | 0 |
| Myrtaceae | *Psidium australe* | 1 | 2 | 0 | 0 | 0 | 0 | 0 | 0 | 0 |
| Myrtaceae | *Psidium bahianum* | 0 | 2 | 0 | 0 | 0 | 0 | 0 | 0 | 0 |
| Myrtaceae | *Psidium brownianum* | 1 | 0 | 0 | 0 | 0 | 0 | 0 | 0 | 0 |
| Myrtaceae | *Psidium cauliflorum* | 0 | 0 | 0 | 4 | 0 | 0 | 0 | 0 | 0 |
| Myrtaceae | *Psidium decussatum* | 0 | 0 | 0 | 0 | 0 | 6 | 0 | 0 | 0 |
| Myrtaceae | *Psidium ganevii* | 1 | 0 | 0 | 0 | 0 | 0 | 0 | 8 | 0 |
| Myrtaceae | *Psidium grandifolium* | 1 | 0 | 0 | 0 | 0 | 0 | 0 | 0 | 0 |
| Myrtaceae | *Psidium guajava* | 0 | 0 | 3 | 0 | 0 | 6 | 7 | 0 | 9 |
| Myrtaceae | *Psidium guineense* | 1 | 2 | 3 | 4 | 5 | 0 | 0 | 8 | 0 |
| Myrtaceae | *Psidium guyanense* | 0 | 2 | 0 | 0 | 0 | 0 | 0 | 0 | 0 |
| Myrtaceae | *Psidium hians* | 0 | 0 | 0 | 0 | 0 | 0 | 0 | 0 | 9 |
| Myrtaceae | *Psidium laruotteanum* | 0 | 2 | 0 | 0 | 0 | 0 | 0 | 0 | 0 |
| Myrtaceae | *Psidium myrsinites* | 1 | 2 | 0 | 0 | 5 | 0 | 0 | 0 | 9 |
| Myrtaceae | *Psidium myrtoides* | 0 | 2 | 3 | 0 | 5 | 0 | 0 | 0 | 9 |
| Myrtaceae | *Psidium oligospermum* | 0 | 2 | 0 | 4 | 5 | 0 | 0 | 8 | 0 |
| Myrtaceae | *Psidium riparium* | 0 | 0 | 0 | 0 | 5 | 0 | 0 | 0 | 0 |
| Myrtaceae | *Psidium rufum* | 1 | 0 | 3 | 0 | 0 | 0 | 0 | 8 | 0 |
| Myrtaceae | *Psidium salutare* | 1 | 2 | 3 | 0 | 5 | 0 | 0 | 0 | 0 |
| Myrtaceae | *Psidium sartorianum* | 0 | 2 | 0 | 0 | 5 | 0 | 7 | 8 | 9 |
| Myrtaceae | *Psidium schenckianum* | 1 | 0 | 3 | 0 | 5 | 6 | 0 | 0 | 0 |
| Loranthaceae | *Psittacanthus calyculatus* | 0 | 0 | 0 | 0 | 5 | 0 | 0 | 0 | 0 |
| Loranthaceae | *Psittacanthus cordatus* | 0 | 0 | 0 | 0 | 0 | 0 | 7 | 8 | 0 |
| Rubiaceae | *Psychotria bahiensis* | 0 | 2 | 3 | 4 | 0 | 0 | 0 | 0 | 0 |
| Rubiaceae | *Psychotria bracteocardia* | 0 | 2 | 0 | 0 | 5 | 0 | 0 | 0 | 0 |
| Rubiaceae | *Psychotria capitata* | 0 | 0 | 0 | 0 | 5 | 0 | 0 | 0 | 0 |
| Rubiaceae | *Psychotria carthagenensis* | 1 | 2 | 3 | 0 | 0 | 0 | 0 | 0 | 0 |
| Rubiaceae | *Psychotria colorata* | 0 | 2 | 0 | 0 | 5 | 0 | 0 | 0 | 0 |
| Rubiaceae | *Psychotria cuspidata* | 0 | 0 | 0 | 0 | 5 | 0 | 0 | 0 | 0 |
| Rubiaceae | *Psychotria deflexa* | 0 | 2 | 0 | 0 | 0 | 0 | 0 | 0 | 0 |
| Rubiaceae | *Psychotria hoffmannseggiana* | 0 | 2 | 3 | 0 | 0 | 0 | 0 | 0 | 0 |
| Rubiaceae | *Psychotria jambosioides* | 0 | 2 | 0 | 0 | 0 | 0 | 0 | 0 | 0 |
| Rubiaceae | *Psychotria leiocarpa* | 1 | 0 | 0 | 4 | 5 | 0 | 0 | 0 | 0 |
| Rubiaceae | *Psychotria mapourioides* | 0 | 2 | 0 | 0 | 0 | 0 | 0 | 0 | 0 |
| Rubiaceae | *Psychotria minutiflora* | 0 | 2 | 0 | 0 | 0 | 0 | 0 | 0 | 0 |
| Rubiaceae | *Psychotria stachyoides* | 1 | 2 | 3 | 0 | 0 | 0 | 0 | 0 | 0 |
| Rubiaceae | *Psychotria subtriflora* | 0 | 0 | 3 | 0 | 0 | 0 | 0 | 0 | 0 |
| Rubiaceae | *Psychotria vellosiana* | 0 | 2 | 3 | 4 | 0 | 0 | 0 | 0 | 0 |
| Fabaceae | *Pterocarpus monophyllus* | 0 | 0 | 0 | 0 | 0 | 0 | 0 | 8 | 0 |
| Fabaceae | *Pterocarpus rohrii* | 0 | 2 | 3 | 0 | 0 | 0 | 0 | 0 | 0 |
| Fabaceae | *Pterocarpus villosus* | 1 | 0 | 0 | 0 | 0 | 0 | 7 | 8 | 0 |
| Fabaceae | *Pterocarpus violaceus* | 0 | 2 | 3 | 0 | 0 | 6 | 0 | 0 | 0 |
| Fabaceae | *Pterocarpus zehntneri* | 0 | 0 | 3 | 0 | 0 | 0 | 0 | 8 | 0 |
| Fabaceae | *Pterodon abruptus* | 0 | 0 | 0 | 0 | 0 | 6 | 7 | 8 | 9 |
| Fabaceae | *Pterodon emarginatus* | 0 | 0 | 3 | 0 | 0 | 0 | 7 | 8 | 0 |
| Fabaceae | *Pterodon pubescens* | 0 | 0 | 3 | 0 | 0 | 0 | 0 | 0 | 0 |
| Fabaceae | *Pterogyne nitens* | 1 | 0 | 3 | 0 | 5 | 6 | 7 | 8 | 0 |
| Melastomataceae | *Pterolepis cataphracta* | 1 | 0 | 0 | 0 | 0 | 0 | 0 | 0 | 0 |
| Melastomataceae | *Pterolepis parnassifolia* | 1 | 0 | 0 | 0 | 0 | 0 | 0 | 0 | 0 |
| Melastomataceae | *Pterolepis parnassiifolia* | 1 | 0 | 0 | 0 | 0 | 0 | 0 | 0 | 0 |
| Malpighiaceae | *Ptilochaeta bahiensis* | 0 | 0 | 3 | 0 | 5 | 0 | 7 | 0 | 0 |
| Lythraceae | *Punica granatum* | 0 | 0 | 0 | 0 | 5 | 6 | 0 | 0 | 0 |
| Vochysiaceae | *Qualea cordata* | 1 | 0 | 0 | 0 | 0 | 0 | 0 | 0 | 0 |
| Vochysiaceae | *Qualea cryptantha* | 1 | 2 | 0 | 0 | 0 | 0 | 0 | 0 | 0 |
| Vochysiaceae | *Qualea grandiflora* | 0 | 2 | 3 | 0 | 0 | 0 | 0 | 0 | 9 |
| Vochysiaceae | *Qualea multiflora* | 0 | 0 | 3 | 0 | 0 | 0 | 0 | 0 | 0 |
| Vochysiaceae | *Qualea parviflora* | 0 | 2 | 3 | 0 | 0 | 0 | 7 | 8 | 9 |
| Malvaceae | *Quararibea floribunda* | 0 | 0 | 3 | 0 | 0 | 0 | 0 | 0 | 0 |
| Malvaceae | *Quararibea penduliflora* | 0 | 0 | 3 | 0 | 0 | 0 | 0 | 0 | 0 |
| Amaranthaceae | *Quaternella ephedroides* | 1 | 0 | 0 | 0 | 0 | 0 | 0 | 0 | 0 |
| Rubiaceae | *Randia armata* | 1 | 2 | 3 | 4 | 5 | 6 | 7 | 8 | 9 |
| Rubiaceae | *Randia calycina* | 0 | 2 | 0 | 0 | 0 | 0 | 7 | 0 | 0 |
| Apocynaceae | *Rauvolfia ligustrina* | 0 | 2 | 0 | 0 | 0 | 0 | 0 | 0 | 0 |
| Apocynaceae | *Rauvolfia paucifolia* | 0 | 2 | 0 | 0 | 0 | 0 | 0 | 8 | 0 |
| Rubiaceae | *Retiniphyllum laxiflorum* | 1 | 0 | 0 | 0 | 0 | 0 | 0 | 0 | 0 |
| Rhamnaceae | *Rhamnidium elaeocarpum* | 0 | 0 | 3 | 0 | 5 | 6 | 0 | 0 | 0 |
| Rhamnaceae | *Rhamnidium molle* | 0 | 0 | 0 | 0 | 5 | 6 | 0 | 0 | 0 |
| Rhamnaceae | *Rhamnus sphaerosperma* | 1 | 0 | 0 | 0 | 0 | 0 | 0 | 0 | 0 |
| Rhizophoraceae | *Rhizophora mangle* | 0 | 0 | 0 | 0 | 0 | 0 | 7 | 0 | 0 |
| Fabaceae | *Rhynchosia minima* | 1 | 0 | 0 | 0 | 0 | 0 | 0 | 0 | 0 |
| Fabaceae | *Rhynchosia phaseoloides* | 1 | 0 | 0 | 0 | 0 | 0 | 0 | 0 | 0 |
| Fabaceae | *Rhynchosia reticulata* | 0 | 0 | 0 | 4 | 0 | 0 | 0 | 0 | 0 |
| Rubiaceae | *Richardia grandiflora* | 0 | 0 | 0 | 0 | 0 | 0 | 7 | 8 | 0 |
| Phyllanthaceae | *Richeria grandis* | 1 | 2 | 0 | 0 | 0 | 0 | 0 | 0 | 0 |
| Asteraceae | *Richterago discoidea* | 1 | 0 | 0 | 0 | 0 | 0 | 0 | 0 | 0 |
| Euphorbiaceae | *Ricinus communis* | 0 | 2 | 0 | 0 | 5 | 0 | 7 | 0 | 0 |
| Phyllanthaceae | *Rivina humilis* | 0 | 0 | 0 | 4 | 5 | 0 | 7 | 0 | 0 |
| Asteraceae | *Rolandra fruticosa* | 0 | 0 | 0 | 0 | 5 | 0 | 0 | 0 | 0 |
| Proteaceae | *Roupala caparoensis* | 0 | 0 | 3 | 0 | 0 | 0 | 0 | 0 | 0 |
| Proteaceae | *Roupala consimilis* | 0 | 2 | 0 | 0 | 0 | 0 | 0 | 0 | 0 |
| Proteaceae | *Roupala montana* | 1 | 2 | 3 | 0 | 5 | 0 | 0 | 8 | 0 |
| Proteaceae | *Roupala paulensis* | 0 | 2 | 0 | 4 | 5 | 0 | 0 | 0 | 0 |
| Connaraceae | *Rourea induta* | 0 | 0 | 0 | 0 | 0 | 0 | 0 | 8 | 0 |
| Connaraceae | *Rourea martiana* | 0 | 0 | 0 | 0 | 5 | 0 | 0 | 0 | 0 |
| Rosaceae | *Rubus brasiliensis* | 1 | 0 | 0 | 0 | 0 | 0 | 0 | 0 | 0 |
| Rosaceae | *Rubus imperialis* | 0 | 0 | 0 | 4 | 0 | 0 | 0 | 0 | 0 |
| Rubiaceae | *Rudgea irregularis* | 1 | 0 | 0 | 0 | 0 | 0 | 0 | 0 | 0 |
| Rubiaceae | *Rudgea jacobinensis* | 1 | 2 | 0 | 4 | 5 | 0 | 0 | 0 | 0 |
| Rubiaceae | *Rudgea japurensis* | 0 | 0 | 3 | 0 | 0 | 0 | 0 | 0 | 0 |
| Rubiaceae | *Rudgea jasminoides* | 0 | 0 | 3 | 4 | 0 | 0 | 0 | 0 | 0 |
| Rubiaceae | *Rudgea obesiflora* | 0 | 0 | 0 | 0 | 0 | 0 | 0 | 8 | 0 |
| Rubiaceae | *Rudgea obtusa* | 0 | 0 | 0 | 0 | 0 | 0 | 0 | 8 | 0 |
| Acanthaceae | *Ruellia asperula* | 0 | 0 | 3 | 4 | 5 | 6 | 7 | 8 | 0 |
| Acanthaceae | *Ruellia bahiensis* | 0 | 0 | 0 | 0 | 5 | 0 | 7 | 8 | 9 |
| Acanthaceae | *Ruellia devosiana* | 0 | 0 | 0 | 4 | 0 | 0 | 0 | 0 | 0 |
| Acanthaceae | *Ruellia geminiflora* | 0 | 0 | 0 | 4 | 5 | 0 | 0 | 8 | 0 |
| Acanthaceae | *Ruellia inundata* | 0 | 0 | 0 | 0 | 0 | 0 | 0 | 8 | 0 |
| Acanthaceae | *Ruellia paniculata* | 0 | 2 | 0 | 4 | 5 | 6 | 7 | 8 | 9 |
| Acanthaceae | *Ruellia villosa* | 1 | 0 | 0 | 0 | 0 | 0 | 0 | 0 | 0 |
| Polygonaceae | *Ruprechtia apetala* | 0 | 0 | 0 | 0 | 0 | 6 | 0 | 0 | 0 |
| Polygonaceae | *Ruprechtia laxiflora* | 0 | 0 | 0 | 0 | 5 | 6 | 0 | 0 | 0 |
| Polygonaceae | *Ruprechtia ramiflora* | 0 | 0 | 0 | 0 | 0 | 0 | 0 | 8 | 0 |
| Polygonaceae | *Rupreschtia apetala* | 0 | 0 | 0 | 0 | 5 | 0 | 0 | 0 | 0 |
| Plantaginaceae | *Russelia equisetiformis* | 0 | 0 | 0 | 0 | 0 | 0 | 0 | 0 | 9 |
| Arecaceae | *Sabal maritima* | 0 | 0 | 0 | 0 | 0 | 0 | 0 | 0 | 9 |
| Humiriaceae | *Sacoglottis guianensis* | 0 | 0 | 0 | 0 | 0 | 0 | 0 | 8 | 0 |
| Humiriaceae | *Sacoglottis mattogrossensis* | 0 | 0 | 0 | 0 | 0 | 0 | 0 | 8 | 0 |
| Celastraceae | *Salacia crassifolia* | 1 | 0 | 0 | 0 | 0 | 0 | 0 | 0 | 0 |
| Celastraceae | *Salacia elliptica* | 0 | 0 | 3 | 0 | 0 | 0 | 0 | 0 | 9 |
| Vochysiaceae | *Salvertia convallariodora* | 0 | 0 | 0 | 0 | 0 | 0 | 0 | 0 | 9 |
| Rubiaceae | *Salzmannia nitida* | 0 | 2 | 0 | 0 | 5 | 0 | 0 | 0 | 0 |
| Fabaceae | *Samanea inopinata* | 0 | 0 | 3 | 0 | 0 | 0 | 0 | 0 | 0 |
| Fabaceae | *Samanea tubulosa* | 0 | 2 | 3 | 0 | 0 | 0 | 0 | 0 | 9 |
| Sapindaceae | *Sapindus saponaria* | 0 | 0 | 0 | 0 | 5 | 6 | 7 | 0 | 9 |
| Euphorbiaceae | *Sapium argutum* | 0 | 0 | 0 | 0 | 5 | 6 | 7 | 8 | 9 |
| Euphorbiaceae | *Sapium glandulosum* | 1 | 0 | 3 | 4 | 5 | 6 | 7 | 8 | 9 |
| Euphorbiaceae | *Sapium obovatum* | 0 | 0 | 0 | 0 | 0 | 0 | 7 | 0 | 0 |
| Euphorbiaceae | *Sapium sebiferum* | 0 | 0 | 0 | 0 | 0 | 0 | 7 | 0 | 0 |
| Ochnaceae | *Sauvagesia insignis* | 1 | 0 | 0 | 0 | 0 | 0 | 0 | 0 | 0 |
| Ochnaceae | *Sauvagesia paniculata* | 1 | 0 | 0 | 0 | 0 | 0 | 0 | 0 | 0 |
| Ochnaceae | *Sauvagesia semicylindrifolia* | 1 | 0 | 0 | 0 | 0 | 0 | 0 | 0 | 0 |
| Phyllanthaceae | *Savia dictyocarpa* | 0 | 0 | 3 | 0 | 5 | 6 | 0 | 0 | 0 |
| Phyllanthaceae | *Savia sessiliflora* | 0 | 0 | 0 | 4 | 5 | 6 | 0 | 0 | 0 |
| Araliaceae | *Schefflera macrocarpa* | 1 | 2 | 0 | 0 | 0 | 0 | 0 | 0 | 0 |
| Araliaceae | *Schefflera morototoni* | 0 | 2 | 3 | 4 | 0 | 0 | 0 | 0 | 0 |
| Araliaceae | *Schefflera vinosa* | 1 | 0 | 0 | 0 | 0 | 0 | 0 | 0 | 0 |
| Anacardiaceae | *Schinopsis brasiliensis* | 0 | 0 | 3 | 4 | 5 | 6 | 7 | 0 | 9 |
| Anacardiaceae | *Schinus terebinthifolius* | 0 | 2 | 0 | 0 | 5 | 0 | 0 | 0 | 0 |
| Schoepfiaceae | *Schoepfia brasiliensis* | 0 | 2 | 3 | 4 | 5 | 0 | 0 | 8 | 0 |
| Gentianaceae | *Schultesia pachyphylla* | 1 | 0 | 0 | 0 | 0 | 0 | 0 | 0 | 0 |
| Marcgraviaceae | *Schwartzia adamantium* | 0 | 2 | 0 | 0 | 5 | 0 | 0 | 0 | 0 |
| Marcgraviaceae | *Schwartzia brasiliensis* | 0 | 0 | 3 | 0 | 5 | 0 | 0 | 0 | 0 |
| Solanaceae | *Schwenckia americana* | 0 | 2 | 0 | 0 | 0 | 0 | 7 | 0 | 0 |
| Fabaceae | *Sclerolobium densiflorum* | 0 | 0 | 3 | 0 | 0 | 0 | 0 | 0 | 0 |
| Fabaceae | *Sclerolobium hypoleucum* | 0 | 0 | 0 | 0 | 0 | 0 | 0 | 8 | 0 |
| Fabaceae | *Sclerolobium paniculatum* | 0 | 0 | 3 | 0 | 0 | 0 | 0 | 8 | 9 |
| Fabaceae | *Sclerolobium pilgerianum* | 0 | 0 | 3 | 0 | 0 | 0 | 0 | 0 | 0 |
| Plantaginaceae | *Scoparia dulcis* | 0 | 0 | 0 | 4 | 5 | 0 | 0 | 0 | 0 |
| Euphorbiaceae | *Sebastiania brasiliensis* | 0 | 2 | 3 | 4 | 5 | 0 | 7 | 0 | 0 |
| Euphorbiaceae | *Sebastiania brevifolia* | 1 | 2 | 0 | 4 | 0 | 0 | 7 | 8 | 0 |
| Euphorbiaceae | *Sebastiania jacobinensis* | 0 | 0 | 0 | 4 | 0 | 0 | 0 | 0 | 0 |
| Euphorbiaceae | *Sebastiania larensis* | 0 | 0 | 0 | 0 | 0 | 0 | 7 | 8 | 0 |
| Euphorbiaceae | *Sebastiania macrocarpa* | 0 | 0 | 0 | 0 | 5 | 6 | 0 | 0 | 0 |
| Euphorbiaceae | *Sebastiania potamophila* | 0 | 0 | 0 | 0 | 5 | 0 | 0 | 0 | 0 |
| Euphorbiaceae | *Sebastiania schottiana* | 0 | 0 | 0 | 0 | 5 | 0 | 0 | 0 | 0 |
| Apocynaceae | *Secondatia densiflora* | 0 | 0 | 0 | 0 | 0 | 0 | 0 | 0 | 9 |
| Apocynaceae | *Secondatia floribunda* | 0 | 2 | 0 | 0 | 0 | 0 | 0 | 8 | 0 |
| Polygalaceae | *Securidaca coriacea* | 0 | 0 | 0 | 4 | 0 | 0 | 0 | 0 | 0 |
| Polygalaceae | *Securidaca diversifolia* | 0 | 0 | 0 | 0 | 5 | 0 | 0 | 0 | 0 |
| Selaginellaceae | *Selaginella convoluta* | 0 | 0 | 0 | 0 | 5 | 0 | 0 | 0 | 0 |
| Asteraceae | *Semiria viscosa* | 1 | 0 | 0 | 0 | 0 | 0 | 0 | 0 | 0 |
| Euphorbiaceae | *Senefeldera verticillata* | 0 | 0 | 3 | 0 | 0 | 0 | 0 | 0 | 0 |
| Fabaceae | *Senegalia bahiensis* | 0 | 0 | 3 | 4 | 5 | 6 | 7 | 0 | 9 |
| Fabaceae | *Senegalia giganticarpa* | 0 | 2 | 0 | 0 | 0 | 6 | 0 | 0 | 0 |
| Fabaceae | *Senegalia globosa* | 0 | 0 | 0 | 0 | 0 | 0 | 7 | 0 | 0 |
| Fabaceae | *Senegalia langsdorffii* | 1 | 2 | 3 | 0 | 5 | 0 | 7 | 8 | 9 |
| Fabaceae | *Senegalia lasiophylla* | 0 | 0 | 0 | 0 | 0 | 0 | 7 | 0 | 0 |
| Fabaceae | *Senegalia martii* | 0 | 0 | 3 | 0 | 0 | 0 | 0 | 0 | 0 |
| Fabaceae | *Senegalia paniculata* | 0 | 0 | 0 | 0 | 5 | 0 | 0 | 0 | 0 |
| Fabaceae | *Senegalia piauhiensis* | 0 | 0 | 0 | 0 | 5 | 6 | 7 | 0 | 9 |
| Fabaceae | *Senegalia polyphylla* | 0 | 2 | 3 | 4 | 5 | 6 | 7 | 8 | 9 |
| Fabaceae | *Senegalia riparia* | 0 | 0 | 3 | 0 | 5 | 0 | 7 | 0 | 9 |
| Fabaceae | *Senegalia tenuifolia* | 1 | 2 | 0 | 4 | 5 | 6 | 7 | 8 | 9 |
| Fabaceae | *Senna acuruensis* | 1 | 0 | 3 | 0 | 5 | 0 | 7 | 8 | 9 |
| Fabaceae | *Senna alata* | 0 | 0 | 0 | 0 | 5 | 0 | 7 | 8 | 0 |
| Fabaceae | *Senna angulata* | 0 | 0 | 0 | 0 | 0 | 0 | 7 | 0 | 0 |
| Fabaceae | *Senna aurantia* | 0 | 0 | 0 | 0 | 0 | 0 | 7 | 8 | 9 |
| Fabaceae | *Senna aversiflora* | 0 | 0 | 0 | 4 | 0 | 0 | 0 | 0 | 0 |
| Fabaceae | *Senna bicapsularis* | 0 | 0 | 0 | 0 | 0 | 6 | 0 | 0 | 0 |
| Fabaceae | *Senna bracteosa* | 0 | 0 | 0 | 0 | 5 | 0 | 0 | 0 | 0 |
| Fabaceae | *Senna cana* | 1 | 2 | 0 | 4 | 5 | 6 | 0 | 0 | 0 |
| Fabaceae | *Senna catingae* | 0 | 0 | 0 | 0 | 0 | 6 | 0 | 0 | 0 |
| Fabaceae | *Senna cearensis* | 0 | 2 | 0 | 0 | 0 | 0 | 7 | 8 | 9 |
| Fabaceae | *Senna gardneri* | 0 | 0 | 0 | 4 | 0 | 0 | 7 | 8 | 9 |
| Fabaceae | *Senna georgica* | 0 | 2 | 3 | 4 | 0 | 6 | 0 | 0 | 9 |
| Fabaceae | *Senna lechriosperma* | 0 | 0 | 0 | 4 | 0 | 0 | 0 | 0 | 9 |
| Fabaceae | *Senna macranthera* | 1 | 2 | 3 | 4 | 5 | 6 | 7 | 8 | 9 |
| Fabaceae | *Senna martiana* | 0 | 0 | 0 | 0 | 0 | 6 | 7 | 0 | 0 |
| Fabaceae | *Senna multijuga* | 0 | 0 | 3 | 0 | 0 | 0 | 0 | 0 | 0 |
| Fabaceae | *Senna obtusifolia* | 0 | 0 | 0 | 4 | 0 | 0 | 7 | 8 | 0 |
| Fabaceae | *Senna occidentalis* | 0 | 0 | 0 | 4 | 5 | 6 | 7 | 0 | 9 |
| Fabaceae | *Senna pendula* | 0 | 2 | 0 | 0 | 5 | 0 | 0 | 0 | 0 |
| Fabaceae | *Senna phlebadenia* | 0 | 2 | 0 | 0 | 5 | 0 | 0 | 0 | 0 |
| Fabaceae | *Senna pilifera* | 0 | 2 | 0 | 0 | 0 | 0 | 0 | 8 | 0 |
| Fabaceae | *Senna quinquangulata* | 0 | 2 | 0 | 0 | 0 | 0 | 0 | 0 | 0 |
| Fabaceae | *Senna rizzinii* | 0 | 2 | 3 | 4 | 5 | 6 | 7 | 8 | 9 |
| Fabaceae | *Senna rugosa* | 1 | 2 | 0 | 0 | 0 | 0 | 0 | 8 | 9 |
| Fabaceae | *Senna siamea* | 0 | 0 | 0 | 0 | 0 | 0 | 7 | 0 | 0 |
| Fabaceae | *Senna silvestris* | 1 | 0 | 0 | 0 | 0 | 0 | 0 | 8 | 0 |
| Fabaceae | *Senna spectabilis* | 0 | 0 | 3 | 4 | 5 | 6 | 7 | 0 | 0 |
| Fabaceae | *Senna splendida* | 0 | 2 | 0 | 4 | 5 | 0 | 7 | 8 | 9 |
| Fabaceae | *Senna trachypus* | 0 | 2 | 0 | 0 | 5 | 0 | 7 | 8 | 9 |
| Fabaceae | *Senna tropica* | 0 | 0 | 0 | 0 | 0 | 0 | 7 | 0 | 0 |
| Fabaceae | *Senna uniflora* | 1 | 0 | 0 | 0 | 5 | 6 | 7 | 0 | 0 |
| Fabaceae | *Senna velutina* | 0 | 0 | 0 | 0 | 0 | 0 | 0 | 0 | 9 |
| Sapindaceae | *Serjania caracasana* | 0 | 0 | 0 | 0 | 0 | 0 | 0 | 0 | 9 |
| Sapindaceae | *Serjania glabrata* | 0 | 0 | 3 | 0 | 0 | 0 | 0 | 0 | 0 |
| Sapindaceae | *Serjania pernambucensis* | 1 | 0 | 0 | 0 | 0 | 0 | 0 | 0 | 0 |
| Fabaceae | *Sesbania exasperata* | 0 | 0 | 0 | 0 | 5 | 6 | 0 | 0 | 0 |
| Fabaceae | *Sesbania virgata* | 0 | 0 | 0 | 0 | 5 | 0 | 0 | 0 | 0 |
| Malvaceae | *Sida angustissima* | 0 | 2 | 0 | 0 | 0 | 0 | 0 | 0 | 0 |
| Malvaceae | *Sida ciliaris* | 0 | 0 | 0 | 0 | 0 | 0 | 7 | 8 | 9 |
| Malvaceae | *Sida cordifolia* | 0 | 2 | 0 | 4 | 5 | 6 | 7 | 0 | 0 |
| Malvaceae | *Sida galheirensis* | 0 | 0 | 0 | 4 | 5 | 6 | 7 | 8 | 9 |
| Malvaceae | *Sida glomerata* | 0 | 2 | 0 | 4 | 5 | 0 | 7 | 0 | 9 |
| Malvaceae | *Sida linifolia* | 1 | 2 | 0 | 0 | 0 | 0 | 0 | 8 | 0 |
| Malvaceae | *Sida rhombifolia* | 0 | 0 | 0 | 4 | 0 | 0 | 0 | 8 | 0 |
| Malvaceae | *Sida rubifolia* | 0 | 0 | 0 | 0 | 5 | 0 | 0 | 0 | 0 |
| Malvaceae | *Sida salviifolia* | 0 | 0 | 0 | 0 | 0 | 0 | 0 | 8 | 0 |
| Malvaceae | *Sida spinosa* | 0 | 0 | 0 | 4 | 5 | 0 | 0 | 8 | 0 |
| Malvaceae | *Sida tuberculata* | 1 | 0 | 0 | 0 | 0 | 0 | 0 | 0 | 0 |
| Malvaceae | *Sida ulei* | 0 | 2 | 0 | 4 | 5 | 0 | 7 | 8 | 0 |
| Malvaceae | *Sida urens* | 0 | 2 | 0 | 0 | 0 | 0 | 7 | 0 | 0 |
| Malvaceae | *Sidastrum micranthum* | 0 | 2 | 0 | 4 | 0 | 6 | 7 | 0 | 0 |
| Malvaceae | *Sidastrum multiflorum* | 0 | 2 | 0 | 4 | 5 | 0 | 0 | 0 | 0 |
| Malvaceae | *Sidastrum paniculatum* | 0 | 0 | 0 | 4 | 5 | 0 | 0 | 0 | 0 |
| Sapotaceae | *Sideroxylon obtusifolium* | 0 | 0 | 3 | 0 | 5 | 6 | 7 | 8 | 9 |
| Rutaceae | *Sigmatanthus trifoliatus* | 0 | 0 | 0 | 0 | 0 | 6 | 0 | 8 | 0 |
| Simaroubaceae | *Simaba amara* | 1 | 0 | 0 | 0 | 0 | 0 | 0 | 0 | 0 |
| Simaroubaceae | *Simaba cedron* | 0 | 0 | 0 | 0 | 0 | 0 | 0 | 8 | 9 |
| Simaroubaceae | *Simaba ferruginea* | 0 | 0 | 0 | 0 | 5 | 0 | 7 | 8 | 0 |
| Simaroubaceae | *Simaba floribunda* | 0 | 0 | 0 | 4 | 5 | 0 | 0 | 0 | 0 |
| Simaroubaceae | *Simaba guianensis* | 0 | 0 | 0 | 0 | 0 | 0 | 0 | 0 | 9 |
| Simaroubaceae | *Simaba maiana* | 0 | 0 | 0 | 0 | 0 | 0 | 0 | 0 | 9 |
| Simaroubaceae | *Simaba trichilioides* | 0 | 2 | 0 | 0 | 0 | 0 | 0 | 8 | 9 |
| Simaroubaceae | *Simarouba amara* | 1 | 2 | 3 | 4 | 0 | 0 | 0 | 8 | 9 |
| Simaroubaceae | *Simarouba versicolor* | 1 | 2 | 3 | 0 | 0 | 0 | 7 | 8 | 9 |
| Rubiaceae | *Simira gardneriana* | 0 | 0 | 0 | 0 | 0 | 6 | 7 | 0 | 0 |
| Siparunaceae | *Siparuna guianensis* | 1 | 2 | 3 | 4 | 0 | 0 | 0 | 0 | 0 |
| Campanulaceae | *Siphocampylus imbricatus* | 1 | 0 | 0 | 0 | 0 | 0 | 0 | 0 | 0 |
| Myrtaceae | *Siphoneugena dussii* | 1 | 0 | 0 | 0 | 0 | 0 | 0 | 0 | 0 |
| Elaeocarpaceae | *Sloanea garckeana* | 0 | 2 | 0 | 0 | 0 | 0 | 0 | 0 | 0 |
| Elaeocarpaceae | *Sloanea guianensis* | 1 | 2 | 0 | 4 | 0 | 0 | 0 | 0 | 0 |
| Elaeocarpaceae | *Sloanea monosperma* | 0 | 0 | 3 | 0 | 0 | 0 | 0 | 0 | 0 |
| Elaeocarpaceae | *Sloanea obtusifolia* | 0 | 2 | 0 | 4 | 0 | 0 | 0 | 0 | 0 |
| Elaeocarpaceae | *Sloanea parviflora* | 0 | 2 | 0 | 0 | 0 | 0 | 0 | 0 | 0 |
| Elaeocarpaceae | *Sloanea pubescens* | 0 | 0 | 3 | 0 | 0 | 0 | 0 | 0 | 0 |
| Elaeocarpaceae | *Sloanea stipitata* | 0 | 0 | 3 | 0 | 0 | 0 | 0 | 0 | 0 |
| Smilacaceae | *Smilax campestris* | 1 | 0 | 0 | 0 | 0 | 0 | 0 | 0 | 0 |
| Smilacaceae | *Smilax elastica* | 1 | 0 | 0 | 0 | 0 | 0 | 0 | 0 | 0 |
| Solanaceae | *Solanum absconditum* | 0 | 2 | 0 | 0 | 0 | 0 | 7 | 8 | 0 |
| Solanaceae | *Solanum acerifolium* | 0 | 2 | 0 | 0 | 0 | 0 | 0 | 0 | 0 |
| Solanaceae | *Solanum aculeatissimum* | 0 | 0 | 0 | 0 | 5 | 0 | 0 | 0 | 0 |
| Solanaceae | *Solanum agrarium* | 0 | 0 | 0 | 0 | 0 | 6 | 7 | 0 | 0 |
| Solanaceae | *Solanum americanum* | 0 | 0 | 0 | 0 | 5 | 0 | 0 | 0 | 9 |
| Solanaceae | *Solanum asperum* | 0 | 2 | 0 | 4 | 0 | 0 | 7 | 8 | 9 |
| Solanaceae | *Solanum buddleiifolium* | 1 | 0 | 0 | 0 | 0 | 0 | 0 | 0 | 0 |
| Solanaceae | *Solanum caavurana* | 0 | 2 | 0 | 4 | 5 | 0 | 0 | 8 | 0 |
| Solanaceae | *Solanum campaniforme* | 0 | 2 | 0 | 0 | 0 | 0 | 0 | 0 | 0 |
| Solanaceae | *Solanum capsicoides* | 0 | 0 | 0 | 4 | 0 | 0 | 0 | 0 | 0 |
| Solanaceae | *Solanum crinitum* | 1 | 0 | 0 | 0 | 0 | 0 | 7 | 8 | 9 |
| Solanaceae | *Solanum decompositiflorum* | 1 | 0 | 0 | 0 | 0 | 0 | 0 | 0 | 0 |
| Solanaceae | *Solanum depauperatum* | 0 | 0 | 0 | 4 | 0 | 0 | 0 | 8 | 0 |
| Solanaceae | *Solanum diamantinense* | 1 | 0 | 0 | 0 | 0 | 0 | 0 | 0 | 0 |
| Solanaceae | *Solanum didymum* | 0 | 2 | 0 | 0 | 0 | 0 | 0 | 0 | 0 |
| Solanaceae | *Solanum evonymoides* | 0 | 0 | 0 | 0 | 5 | 0 | 0 | 0 | 0 |
| Solanaceae | *Solanum flaccidum* | 0 | 0 | 0 | 0 | 5 | 0 | 0 | 0 | 0 |
| Solanaceae | *Solanum gardneri* | 0 | 0 | 0 | 0 | 5 | 0 | 0 | 0 | 0 |
| Solanaceae | *Solanum hirtellum* | 0 | 0 | 0 | 0 | 5 | 0 | 0 | 0 | 0 |
| Solanaceae | *Solanum jabrense* | 0 | 0 | 0 | 0 | 0 | 6 | 0 | 0 | 0 |
| Solanaceae | *Solanum jamaicense* | 0 | 2 | 0 | 0 | 0 | 0 | 0 | 0 | 0 |
| Solanaceae | *Solanum jussiaei* | 0 | 2 | 0 | 0 | 0 | 0 | 0 | 0 | 0 |
| Solanaceae | *Solanum lycocarpum* | 1 | 0 | 3 | 0 | 0 | 0 | 0 | 0 | 0 |
| Solanaceae | *Solanum megalonyx* | 1 | 2 | 3 | 0 | 5 | 0 | 0 | 0 | 0 |
| Solanaceae | *Solanum melissarum* | 0 | 2 | 0 | 0 | 0 | 0 | 0 | 0 | 0 |
| Solanaceae | *Solanum ovum.fringillae* | 0 | 0 | 0 | 0 | 5 | 0 | 0 | 0 | 0 |
| Solanaceae | *Solanum paludosum* | 0 | 2 | 0 | 0 | 5 | 0 | 7 | 8 | 0 |
| Solanaceae | *Solanum paniculatum* | 1 | 2 | 3 | 4 | 5 | 6 | 7 | 8 | 9 |
| Solanaceae | *Solanum polytrichum* | 0 | 2 | 3 | 0 | 0 | 0 | 0 | 0 | 0 |
| Solanaceae | *Solanum pseudocapsicum* | 0 | 0 | 3 | 0 | 0 | 0 | 0 | 0 | 0 |
| Solanaceae | *Solanum pseudoquina* | 0 | 0 | 0 | 0 | 5 | 0 | 0 | 0 | 0 |
| Solanaceae | *Solanum rhytidoandrum* | 0 | 2 | 0 | 4 | 5 | 6 | 7 | 8 | 9 |
| Solanaceae | *Solanum robustum* | 0 | 2 | 0 | 0 | 0 | 0 | 0 | 0 | 0 |
| Solanaceae | *Solanum sellowianum* | 1 | 0 | 0 | 0 | 0 | 0 | 0 | 0 | 0 |
| Solanaceae | *Solanum sisymbriifolium* | 0 | 0 | 0 | 0 | 0 | 6 | 0 | 0 | 9 |
| Solanaceae | *Solanum stenandrum* | 1 | 0 | 0 | 0 | 0 | 0 | 0 | 0 | 0 |
| Solanaceae | *Solanum stipulaceum* | 1 | 2 | 3 | 4 | 5 | 0 | 7 | 8 | 0 |
| Solanaceae | *Solanum sycocarpum* | 0 | 2 | 0 | 0 | 0 | 0 | 0 | 0 | 0 |
| Solanaceae | *Solanum thomasiifolium* | 1 | 0 | 3 | 0 | 5 | 0 | 0 | 0 | 0 |
| Moraceae | *Sorocea bonplandii* | 0 | 0 | 0 | 4 | 0 | 0 | 0 | 0 | 0 |
| Moraceae | *Sorocea hilarii* | 0 | 0 | 3 | 0 | 0 | 0 | 0 | 0 | 0 |
| Hernandiaceae | *Sparattanthelium botocudorum* | 0 | 2 | 0 | 0 | 5 | 0 | 0 | 0 | 0 |
| Bignoniaceae | *Sparattosperma leucanthum* | 0 | 0 | 3 | 0 | 0 | 0 | 0 | 0 | 0 |
| Bignoniaceae | *Spathodea campanulata* | 0 | 0 | 0 | 0 | 0 | 0 | 0 | 0 | 9 |
| Loganiaceae | *Spigelia cremnophila* | 1 | 0 | 0 | 0 | 0 | 0 | 0 | 0 | 0 |
| Loganiaceae | *Spigelia flava* | 1 | 0 | 0 | 0 | 0 | 0 | 0 | 0 | 0 |
| Loganiaceae | *Spigelia pulchella* | 1 | 0 | 0 | 0 | 0 | 0 | 0 | 0 | 0 |
| Rutaceae | *Spiranthera odoratissima* | 1 | 0 | 0 | 0 | 0 | 0 | 0 | 0 | 0 |
| Anacardiaceae | *Spondias bahiensis* | 0 | 0 | 0 | 0 | 5 | 0 | 0 | 0 | 0 |
| Anacardiaceae | *Spondias dulcis* | 0 | 0 | 0 | 0 | 0 | 6 | 0 | 0 | 0 |
| Anacardiaceae | *Spondias mombin* | 0 | 0 | 3 | 0 | 5 | 6 | 7 | 0 | 0 |
| Anacardiaceae | *Spondias purpurea* | 0 | 0 | 0 | 0 | 0 | 6 | 0 | 0 | 0 |
| Anacardiaceae | *Spondias tuberosa* | 0 | 0 | 3 | 0 | 5 | 6 | 7 | 0 | 9 |
| Anacardiaceae | *Spondias venulosa* | 0 | 0 | 0 | 4 | 0 | 6 | 0 | 0 | 0 |
| Rubiaceae | *Stachyarrhena spicata* | 0 | 0 | 0 | 0 | 0 | 0 | 0 | 0 | 9 |
| Verbenaceae | *Stachytarpheta cayennensis* | 0 | 0 | 0 | 0 | 0 | 0 | 7 | 0 | 0 |
| Verbenaceae | *Stachytarpheta cearensis* | 0 | 2 | 0 | 0 | 0 | 0 | 0 | 0 | 0 |
| Verbenaceae | *Stachytarpheta coccinea* | 0 | 0 | 0 | 0 | 0 | 6 | 7 | 8 | 0 |
| Verbenaceae | *Stachytarpheta crassifolia* | 1 | 0 | 0 | 0 | 0 | 0 | 0 | 0 | 0 |
| Verbenaceae | *Stachytarpheta froesii* | 1 | 0 | 0 | 0 | 0 | 0 | 0 | 0 | 0 |
| Verbenaceae | *Stachytarpheta hatschbachii* | 0 | 0 | 0 | 0 | 0 | 0 | 0 | 0 | 9 |
| Verbenaceae | *Stachytarpheta hispida* | 1 | 0 | 0 | 0 | 0 | 0 | 0 | 0 | 0 |
| Verbenaceae | *Stachytarpheta lactea* | 1 | 0 | 0 | 0 | 0 | 0 | 0 | 0 | 0 |
| Verbenaceae | *Stachytarpheta maximiliani* | 0 | 0 | 0 | 0 | 0 | 0 | 0 | 0 | 9 |
| Verbenaceae | *Stachytarpheta microphylla* | 0 | 0 | 0 | 0 | 0 | 6 | 7 | 0 | 0 |
| Verbenaceae | *Stachytarpheta radlkoferiana* | 1 | 0 | 0 | 0 | 0 | 0 | 0 | 0 | 0 |
| Verbenaceae | *Stachytarpheta tuberculata* | 1 | 0 | 0 | 0 | 0 | 0 | 0 | 0 | 0 |
| Plantaginaceae | *Stemodia foliosa* | 0 | 0 | 0 | 4 | 0 | 0 | 7 | 0 | 0 |
| Plantaginaceae | *Stemodia harleyi* | 1 | 0 | 0 | 0 | 0 | 0 | 0 | 0 | 0 |
| Plantaginaceae | *Stemodia maritima* | 0 | 0 | 0 | 0 | 0 | 0 | 7 | 0 | 0 |
| Cactaceae | *Stephanocereus leucostele* | 0 | 0 | 0 | 0 | 0 | 6 | 0 | 0 | 0 |
| Cactaceae | *Stephanocereus luetzelburgii* | 1 | 0 | 0 | 0 | 0 | 0 | 0 | 0 | 0 |
| Malvaceae | *Sterculia striata* | 0 | 2 | 0 | 0 | 0 | 6 | 0 | 8 | 9 |
| Asteraceae | *Stevia morii* | 1 | 0 | 0 | 0 | 0 | 0 | 0 | 0 | 0 |
| Malpighiaceae | *Stigmaphyllon blanchetii* | 0 | 0 | 0 | 0 | 5 | 0 | 0 | 0 | 0 |
| Malpighiaceae | *Stigmaphyllon paralias* | 1 | 0 | 3 | 4 | 5 | 0 | 0 | 8 | 0 |
| Euphorbiaceae | *Stillingia dichotoma* | 1 | 0 | 0 | 0 | 0 | 0 | 0 | 0 | 0 |
| Euphorbiaceae | *Stillingia saxatilis* | 1 | 0 | 0 | 0 | 0 | 0 | 0 | 0 | 0 |
| Euphorbiaceae | *Stillingia trapezoidea* | 0 | 0 | 0 | 0 | 5 | 0 | 0 | 8 | 9 |
| Euphorbiaceae | *Stillingia uleana* | 1 | 0 | 0 | 0 | 0 | 0 | 0 | 8 | 0 |
| Asteraceae | *Stilpnopappus pratensis* | 1 | 0 | 0 | 0 | 0 | 0 | 7 | 0 | 0 |
| Asteraceae | *Stilpnopappus scaposus* | 1 | 0 | 0 | 0 | 0 | 0 | 0 | 0 | 0 |
| Asteraceae | *Stilpnopappus tomentosus* | 1 | 0 | 0 | 0 | 0 | 0 | 0 | 0 | 0 |
| Asteraceae | *Stilpnopappus trichospiroides* | 1 | 0 | 0 | 0 | 0 | 6 | 0 | 0 | 0 |
| Bignoniaceae | *Stizophyllum perforatum* | 0 | 0 | 3 | 0 | 0 | 0 | 0 | 0 | 0 |
| Marantaceae | *Stromanthe tonckat* | 0 | 0 | 0 | 4 | 0 | 0 | 0 | 0 | 0 |
| Loranthaceae | *Struthanthus marginatus* | 1 | 0 | 0 | 0 | 0 | 0 | 0 | 0 | 0 |
| Loganiaceae | *Strychnos parviflora* | 0 | 0 | 0 | 0 | 0 | 0 | 0 | 8 | 0 |
| Loganiaceae | *Strychnos parvifolia* | 0 | 0 | 3 | 0 | 5 | 0 | 7 | 8 | 0 |
| Loganiaceae | *Strychnos rubiginosa* | 0 | 0 | 0 | 0 | 5 | 0 | 7 | 8 | 9 |
| Fabaceae | *Stryphnodendron adstringens* | 0 | 0 | 3 | 0 | 0 | 0 | 0 | 0 | 0 |
| Fabaceae | *Stryphnodendron barbatimam* | 0 | 2 | 0 | 0 | 0 | 0 | 0 | 0 | 0 |
| Fabaceae | *Stryphnodendron coriaceum* | 0 | 2 | 0 | 0 | 0 | 0 | 0 | 0 | 9 |
| Fabaceae | *Stryphnodendron guianense* | 0 | 2 | 0 | 0 | 0 | 0 | 0 | 0 | 0 |
| Fabaceae | *Stryphnodendron pulcherrimum* | 0 | 2 | 0 | 4 | 5 | 0 | 0 | 0 | 0 |
| Fabaceae | *Stryphnodendron rotundifolium* | 1 | 2 | 0 | 0 | 0 | 0 | 0 | 0 | 0 |
| Fabaceae | *Stylosanthes angustifolia* | 0 | 0 | 0 | 0 | 0 | 0 | 0 | 8 | 0 |
| Fabaceae | *Stylosanthes capitata* | 0 | 2 | 0 | 0 | 0 | 0 | 0 | 8 | 0 |
| Fabaceae | *Stylosanthes guianensis* | 0 | 2 | 0 | 0 | 0 | 0 | 0 | 0 | 0 |
| Fabaceae | *Stylosanthes humilis* | 0 | 0 | 0 | 0 | 0 | 0 | 0 | 8 | 0 |
| Fabaceae | *Stylosanthes macrocephala* | 0 | 0 | 0 | 0 | 0 | 0 | 0 | 8 | 0 |
| Fabaceae | *Stylosanthes pilosa* | 0 | 0 | 0 | 0 | 0 | 0 | 7 | 0 | 0 |
| Fabaceae | *Stylosanthes scabra* | 0 | 2 | 0 | 0 | 5 | 0 | 0 | 0 | 0 |
| Fabaceae | *Stylosanthes viscosa* | 0 | 2 | 0 | 0 | 0 | 6 | 7 | 8 | 0 |
| Asteraceae | *Stylotrichium rotundifolium* | 1 | 0 | 0 | 0 | 0 | 0 | 0 | 0 | 0 |
| Styracaceae | *Styrax camporum* | 1 | 2 | 0 | 4 | 0 | 0 | 0 | 0 | 0 |
| Styracaceae | *Styrax ferrugineus* | 0 | 2 | 0 | 0 | 0 | 0 | 0 | 0 | 0 |
| Styracaceae | *Styrax martii* | 1 | 0 | 0 | 0 | 0 | 0 | 0 | 0 | 0 |
| Styracaceae | *Styrax pedicellatus* | 1 | 0 | 0 | 0 | 0 | 0 | 0 | 0 | 0 |
| Fabaceae | *Swartzia acutifolia* | 1 | 0 | 0 | 0 | 0 | 0 | 0 | 0 | 0 |
| Fabaceae | *Swartzia apetala* | 0 | 2 | 3 | 0 | 5 | 0 | 0 | 0 | 0 |
| Fabaceae | *Swartzia bahiensis* | 1 | 0 | 0 | 0 | 0 | 0 | 0 | 0 | 0 |
| Fabaceae | *Swartzia flaemingii* | 0 | 2 | 3 | 4 | 0 | 0 | 7 | 8 | 9 |
| Fabaceae | *Swartzia grandifolia* | 0 | 0 | 0 | 0 | 0 | 0 | 0 | 0 | 9 |
| Fabaceae | *Swartzia langsdorffii* | 0 | 0 | 3 | 0 | 0 | 0 | 0 | 0 | 0 |
| Fabaceae | *Swartzia macrostachya* | 1 | 0 | 0 | 4 | 0 | 0 | 0 | 0 | 0 |
| Fabaceae | *Swartzia oblata* | 0 | 0 | 3 | 0 | 0 | 0 | 0 | 0 | 0 |
| Fabaceae | *Swartzia pickelii* | 0 | 0 | 0 | 4 | 0 | 0 | 0 | 0 | 0 |
| Fabaceae | *Swartzia polita* | 0 | 2 | 0 | 0 | 0 | 0 | 0 | 0 | 0 |
| Fabaceae | *Swartzia simplex* | 0 | 0 | 3 | 0 | 0 | 0 | 0 | 0 | 9 |
| Arecaceae | *Syagrus cearensis* | 0 | 0 | 0 | 0 | 5 | 6 | 0 | 0 | 0 |
| Arecaceae | *Syagrus coronata* | 1 | 0 | 3 | 4 | 5 | 6 | 0 | 0 | 9 |
| Arecaceae | *Syagrus flexuosa* | 1 | 0 | 0 | 0 | 0 | 0 | 0 | 0 | 0 |
| Arecaceae | *Syagrus harleyi* | 1 | 0 | 0 | 0 | 0 | 0 | 0 | 0 | 0 |
| Arecaceae | *Syagrus microphylla* | 1 | 0 | 0 | 0 | 0 | 0 | 0 | 0 | 0 |
| Arecaceae | *Syagrus oleracea* | 0 | 0 | 3 | 0 | 5 | 6 | 0 | 0 | 0 |
| Arecaceae | *Syagrus vagans* | 0 | 0 | 0 | 0 | 5 | 0 | 0 | 0 | 0 |
| Clusiaceae | *Symphonia globulifera* | 0 | 2 | 0 | 0 | 0 | 0 | 0 | 0 | 0 |
| Symplocaceae | *Symplocos celastrinea* | 1 | 0 | 0 | 0 | 0 | 0 | 0 | 0 | 0 |
| Symplocaceae | *Symplocos lanceolata* | 1 | 0 | 0 | 0 | 0 | 0 | 0 | 0 | 0 |
| Symplocaceae | *Symplocos nitens* | 1 | 2 | 0 | 0 | 0 | 0 | 0 | 0 | 0 |
| Myrtaceae | *Syzygium cumini* | 0 | 2 | 0 | 0 | 0 | 0 | 0 | 8 | 9 |
| Myrtaceae | *Syzygium jambos* | 1 | 2 | 0 | 4 | 0 | 0 | 0 | 0 | 0 |
| Bignoniaceae | *Tabebuia aurea* | 0 | 2 | 3 | 0 | 5 | 6 | 7 | 8 | 0 |
| Bignoniaceae | *Tabebuia ochracea* | 0 | 2 | 3 | 4 | 0 | 0 | 7 | 0 | 9 |
| Bignoniaceae | *Tabebuia reticulata* | 0 | 0 | 3 | 0 | 0 | 0 | 0 | 0 | 0 |
| Bignoniaceae | *Tabebuia rosea* | 0 | 0 | 0 | 0 | 0 | 0 | 0 | 0 | 9 |
| Bignoniaceae | *Tabebuia roseoalba* | 0 | 0 | 3 | 4 | 5 | 6 | 0 | 8 | 0 |
| Apocynaceae | *Tabernaemontana catharinensis* | 0 | 2 | 0 | 0 | 0 | 0 | 7 | 8 | 9 |
| Apocynaceae | *Tabernaemontana flavicans* | 0 | 2 | 0 | 0 | 0 | 0 | 0 | 0 | 0 |
| Apocynaceae | *Tabernaemontana hystrix* | 0 | 0 | 0 | 0 | 0 | 0 | 0 | 0 | 9 |
| Apocynaceae | *Tabernaemontana laeta* | 1 | 0 | 0 | 0 | 0 | 0 | 0 | 8 | 9 |
| Apocynaceae | *Tabernaemontana linkii* | 0 | 0 | 0 | 0 | 0 | 0 | 0 | 8 | 0 |
| Apocynaceae | *Tabernaemontana salzmannii* | 0 | 2 | 0 | 0 | 0 | 0 | 0 | 0 | 0 |
| Apocynaceae | *Tabernaemontana solanifolia* | 1 | 0 | 3 | 0 | 0 | 0 | 0 | 0 | 0 |
| Fabaceae | *Tachigali densiflora* | 0 | 2 | 0 | 0 | 5 | 0 | 0 | 0 | 0 |
| Fabaceae | *Tachigali paniculata* | 1 | 0 | 3 | 0 | 0 | 0 | 0 | 0 | 0 |
| Fabaceae | *Tachigali subvelutina* | 1 | 0 | 0 | 0 | 0 | 0 | 0 | 0 | 0 |
| Fabaceae | *Tachigali vulgaris* | 1 | 0 | 0 | 0 | 0 | 0 | 0 | 8 | 9 |
| Cactaceae | *Tacinga funalis* | 0 | 0 | 0 | 0 | 0 | 6 | 0 | 0 | 0 |
| Cactaceae | *Tacinga inamoena* | 0 | 0 | 0 | 0 | 5 | 6 | 7 | 8 | 0 |
| Cactaceae | *Tacinga palmadora* | 0 | 0 | 0 | 0 | 5 | 6 | 0 | 0 | 9 |
| Asteraceae | *Tagetes minuta* | 1 | 0 | 0 | 0 | 0 | 0 | 0 | 0 | 0 |
| Talinaceae | *Talinum fruticosum* | 0 | 0 | 0 | 4 | 0 | 0 | 0 | 0 | 0 |
| Malvaceae | *Talipariti tiliaceum* | 0 | 0 | 0 | 0 | 0 | 6 | 0 | 0 | 0 |
| Sapindaceae | *Talisia esculenta* | 0 | 2 | 3 | 4 | 5 | 6 | 7 | 8 | 9 |
| Fabaceae | *Tamarindus indica* | 0 | 0 | 0 | 0 | 0 | 0 | 7 | 0 | 9 |
| Verbenaceae | *Tamonea curassavica* | 1 | 0 | 0 | 0 | 0 | 0 | 0 | 0 | 0 |
| Verbenaceae | *Tamonea spicata* | 1 | 0 | 0 | 0 | 0 | 0 | 0 | 0 | 0 |
| Anacardiaceae | *Tapirira guianensis* | 1 | 2 | 3 | 4 | 5 | 0 | 0 | 8 | 9 |
| Anacardiaceae | *Tapirira obtusa* | 1 | 2 | 3 | 0 | 0 | 0 | 0 | 0 | 0 |
| Cleomaceae | *Tarenaya spinosa* | 0 | 0 | 0 | 4 | 5 | 6 | 7 | 8 | 0 |
| Bignoniaceae | *Tecoma stans* | 0 | 0 | 0 | 0 | 0 | 6 | 0 | 0 | 9 |
| Fabaceae | *Tephrosia cinerea* | 0 | 0 | 0 | 0 | 0 | 6 | 0 | 0 | 0 |
| Fabaceae | *Tephrosia egregia* | 0 | 0 | 0 | 0 | 0 | 6 | 0 | 8 | 0 |
| Fabaceae | *Tephrosia purpurea* | 0 | 0 | 0 | 0 | 0 | 0 | 7 | 8 | 0 |
| Combretaceae | *Terminalia actinophylla* | 0 | 0 | 3 | 0 | 0 | 0 | 7 | 0 | 9 |
| Combretaceae | *Terminalia argentea* | 0 | 0 | 3 | 0 | 0 | 0 | 0 | 0 | 0 |
| Combretaceae | *Terminalia brasiliensis* | 0 | 2 | 3 | 0 | 5 | 0 | 0 | 0 | 9 |
| Combretaceae | *Terminalia catappa* | 0 | 0 | 0 | 0 | 0 | 6 | 7 | 0 | 9 |
| Combretaceae | *Terminalia fagifolia* | 0 | 0 | 3 | 4 | 5 | 6 | 7 | 0 | 9 |
| Combretaceae | *Terminalia glabrescens* | 1 | 0 | 3 | 0 | 0 | 0 | 7 | 0 | 9 |
| Combretaceae | *Terminalia januarensis* | 0 | 0 | 3 | 0 | 0 | 0 | 0 | 0 | 0 |
| Combretaceae | *Terminalia phaeocarpa* | 0 | 0 | 3 | 0 | 0 | 0 | 0 | 0 | 0 |
| Pentaphylacaceae | *Ternstroemia alnifolia* | 1 | 0 | 0 | 0 | 0 | 0 | 0 | 0 | 0 |
| Pentaphylacaceae | *Ternstroemia brasiliensis* | 1 | 0 | 0 | 0 | 0 | 0 | 0 | 0 | 0 |
| Pentaphylacaceae | *Ternstroemia candolleana* | 1 | 2 | 0 | 0 | 0 | 0 | 0 | 0 | 0 |
| Pentaphylacaceae | *Ternstroemia carnosa* | 1 | 0 | 0 | 0 | 0 | 0 | 0 | 0 | 0 |
| Dilleniaceae | *Tetracera breyniana* | 0 | 0 | 0 | 0 | 0 | 0 | 0 | 0 | 9 |
| Lamiaceae | *Tetradenia riparia* | 0 | 0 | 0 | 0 | 0 | 0 | 0 | 0 | 9 |
| Malpighiaceae | *Tetrapterys cardiophylla* | 1 | 0 | 0 | 0 | 0 | 0 | 0 | 0 | 0 |
| Malpighiaceae | *Tetrapterys discolor* | 0 | 0 | 0 | 4 | 0 | 0 | 0 | 0 | 0 |
| Malpighiaceae | *Tetrapterys ramiflora* | 1 | 0 | 0 | 0 | 0 | 0 | 0 | 0 | 0 |
| Malvaceae | *Thespesia populnea* | 0 | 0 | 0 | 0 | 0 | 6 | 0 | 0 | 9 |
| Combretaceae | *Thiloa glaucocarpa* | 0 | 0 | 0 | 0 | 5 | 6 | 7 | 8 | 9 |
| Malpighiaceae | *Thryallis longifolia* | 0 | 0 | 0 | 4 | 5 | 0 | 0 | 0 | 0 |
| Anacardiaceae | *Thyrsodium spruceanum* | 0 | 2 | 3 | 4 | 5 | 0 | 0 | 0 | 9 |
| Melastomataceae | *Tibouchina barnebyana* | 1 | 0 | 0 | 0 | 0 | 0 | 0 | 0 | 0 |
| Melastomataceae | *Tibouchina blanchetiana* | 1 | 0 | 0 | 0 | 0 | 0 | 0 | 0 | 0 |
| Melastomataceae | *Tibouchina caatingae* | 0 | 0 | 0 | 0 | 5 | 0 | 0 | 0 | 0 |
| Melastomataceae | *Tibouchina candolleana* | 1 | 0 | 0 | 0 | 0 | 0 | 0 | 0 | 0 |
| Melastomataceae | *Tibouchina carvalhoi* | 1 | 0 | 0 | 0 | 0 | 0 | 0 | 0 | 0 |
| Melastomataceae | *Tibouchina clavata* | 1 | 0 | 0 | 0 | 0 | 0 | 0 | 0 | 0 |
| Melastomataceae | *Tibouchina clinopodifolia* | 0 | 0 | 0 | 0 | 0 | 0 | 0 | 0 | 9 |
| Melastomataceae | *Tibouchina comosa* | 1 | 0 | 0 | 0 | 0 | 0 | 0 | 0 | 0 |
| Melastomataceae | *Tibouchina fissinervia* | 1 | 0 | 0 | 0 | 0 | 0 | 0 | 0 | 0 |
| Melastomataceae | *Tibouchina gardneri* | 0 | 2 | 0 | 0 | 0 | 0 | 0 | 0 | 0 |
| Melastomataceae | *Tibouchina heteromalla* | 1 | 2 | 0 | 4 | 0 | 0 | 0 | 0 | 0 |
| Melastomataceae | *Tibouchina lithophila* | 0 | 0 | 0 | 4 | 5 | 0 | 0 | 0 | 0 |
| Melastomataceae | *Tibouchina macrochiton* | 1 | 0 | 0 | 0 | 0 | 0 | 0 | 0 | 0 |
| Melastomataceae | *Tibouchina multiflora* | 0 | 0 | 0 | 4 | 5 | 0 | 0 | 0 | 0 |
| Melastomataceae | *Tibouchina oreophila* | 1 | 0 | 0 | 0 | 0 | 0 | 0 | 0 | 0 |
| Melastomataceae | *Tibouchina paulo.alvinii* | 0 | 0 | 0 | 0 | 5 | 0 | 0 | 0 | 0 |
| Melastomataceae | *Tibouchina pereirae* | 1 | 0 | 0 | 0 | 0 | 0 | 0 | 0 | 0 |
| Melastomataceae | *Tibouchina urceolaris* | 1 | 0 | 0 | 0 | 0 | 0 | 0 | 0 | 0 |
| Melastomataceae | *Tibouchina velutina* | 0 | 0 | 0 | 0 | 0 | 0 | 0 | 8 | 0 |
| Melastomataceae | *Tibouchinopsis glutinosa* | 1 | 0 | 0 | 0 | 0 | 0 | 0 | 0 | 0 |
| Asteraceae | *Tilesia baccata* | 0 | 2 | 3 | 4 | 5 | 0 | 7 | 8 | 9 |
| Rubiaceae | *Tocoyena bullata* | 0 | 0 | 0 | 0 | 5 | 6 | 0 | 8 | 0 |
| Rubiaceae | *Tocoyena formosa* | 1 | 2 | 3 | 4 | 5 | 6 | 7 | 8 | 9 |
| Rubiaceae | *Tocoyena hispidula* | 0 | 0 | 0 | 0 | 0 | 0 | 0 | 0 | 9 |
| Rubiaceae | *Tocoyena sellowiana* | 0 | 2 | 0 | 0 | 0 | 0 | 7 | 8 | 0 |
| Celastraceae | *Tontelea miersii* | 0 | 0 | 3 | 0 | 0 | 0 | 0 | 0 | 0 |
| Boraginaceae | *Tournefortia paniculata* | 0 | 0 | 0 | 0 | 0 | 6 | 0 | 0 | 0 |
| Boraginaceae | *Tournefortia rubicunda* | 0 | 0 | 0 | 0 | 5 | 6 | 7 | 8 | 0 |
| Boraginaceae | *Tournefortia salicifolia* | 0 | 0 | 0 | 0 | 5 | 0 | 0 | 0 | 0 |
| Boraginaceae | *Tournefortia volubilis* | 0 | 0 | 0 | 0 | 5 | 0 | 0 | 0 | 0 |
| Clusiaceae | *Tovomita choisyana* | 0 | 2 | 0 | 0 | 0 | 0 | 0 | 0 | 0 |
| Cannabaceae | *Trema micrantha* | 1 | 2 | 0 | 4 | 5 | 0 | 7 | 8 | 9 |
| Melastomataceae | *Trembleya parviflora* | 1 | 0 | 0 | 0 | 0 | 0 | 0 | 0 | 0 |
| Meliaceae | *Trichilia casaretti* | 0 | 0 | 3 | 0 | 0 | 0 | 0 | 0 | 0 |
| Meliaceae | *Trichilia elegans* | 1 | 2 | 3 | 0 | 0 | 0 | 0 | 8 | 9 |
| Meliaceae | *Trichilia glabra* | 0 | 2 | 0 | 0 | 5 | 0 | 0 | 0 | 0 |
| Meliaceae | *Trichilia hirta* | 0 | 0 | 3 | 4 | 5 | 6 | 0 | 0 | 9 |
| Meliaceae | *Trichilia lepidota* | 0 | 0 | 3 | 0 | 0 | 0 | 0 | 0 | 0 |
| Meliaceae | *Trichilia pseudostipularis* | 0 | 2 | 3 | 0 | 0 | 0 | 0 | 0 | 0 |
| Meliaceae | *Trichilia ramalhoi* | 0 | 2 | 3 | 0 | 0 | 0 | 0 | 0 | 0 |
| Meliaceae | *Trichilia silvatica* | 0 | 0 | 3 | 0 | 0 | 0 | 0 | 0 | 0 |
| Asteraceae | *Trichogonia campestris* | 0 | 0 | 0 | 0 | 0 | 0 | 7 | 0 | 0 |
| Trigoniaceae | *Trigonia nivea* | 1 | 0 | 0 | 4 | 5 | 0 | 7 | 0 | 9 |
| Polygonaceae | *Triplaris gardneriana* | 0 | 0 | 3 | 4 | 5 | 6 | 7 | 8 | 9 |
| Fabaceae | *Trischidium decipiens* | 0 | 0 | 0 | 0 | 0 | 0 | 0 | 8 | 0 |
| Fabaceae | *Trischidium molle* | 0 | 0 | 0 | 4 | 5 | 6 | 7 | 8 | 0 |
| Malvaceae | *Triumfetta althaeoides* | 0 | 0 | 0 | 4 | 0 | 0 | 0 | 0 | 0 |
| Malvaceae | *Triumfetta rhomboidea* | 0 | 2 | 0 | 0 | 0 | 0 | 0 | 8 | 0 |
| Malvaceae | *Triumfetta semitriloba* | 0 | 2 | 0 | 4 | 0 | 0 | 0 | 0 | 0 |
| Asteraceae | *Trixis antimenorrhoea* | 0 | 2 | 0 | 4 | 0 | 0 | 0 | 0 | 0 |
| Asteraceae | *Trixis calycina* | 0 | 0 | 0 | 0 | 0 | 0 | 7 | 0 | 0 |
| Asteraceae | *Trixis divaricata* | 0 | 2 | 0 | 0 | 5 | 0 | 0 | 0 | 0 |
| Asteraceae | *Trixis vauthieri* | 1 | 0 | 0 | 0 | 5 | 0 | 0 | 0 | 0 |
| Turneraceae | *Turnera bahiensis* | 1 | 0 | 0 | 0 | 0 | 0 | 0 | 8 | 0 |
| Turneraceae | *Turnera blanchetiana* | 0 | 2 | 0 | 0 | 0 | 0 | 7 | 8 | 9 |
| Turneraceae | *Turnera calyptrocarpa* | 0 | 0 | 0 | 0 | 5 | 0 | 7 | 8 | 0 |
| Turneraceae | *Turnera cearensis* | 0 | 2 | 0 | 0 | 5 | 6 | 0 | 8 | 0 |
| Turneraceae | *Turnera chamaedrifolia* | 0 | 2 | 0 | 4 | 0 | 0 | 7 | 8 | 0 |
| Turneraceae | *Turnera coerulea* | 0 | 0 | 0 | 0 | 0 | 0 | 7 | 8 | 0 |
| Turneraceae | *Turnera diffusa* | 1 | 0 | 0 | 4 | 5 | 0 | 0 | 8 | 0 |
| Turneraceae | *Turnera harleyi* | 0 | 0 | 0 | 0 | 0 | 0 | 7 | 0 | 0 |
| Turneraceae | *Turnera hermannioides* | 0 | 0 | 0 | 0 | 0 | 6 | 0 | 0 | 0 |
| Turneraceae | *Turnera macrophylla* | 0 | 0 | 0 | 0 | 0 | 6 | 0 | 0 | 0 |
| Turneraceae | *Turnera melochioides* | 1 | 2 | 0 | 0 | 0 | 0 | 7 | 8 | 0 |
| Turneraceae | *Turnera opifera* | 0 | 0 | 0 | 0 | 0 | 0 | 0 | 8 | 0 |
| Turneraceae | *Turnera simulans* | 1 | 0 | 0 | 0 | 0 | 0 | 0 | 0 | 0 |
| Turneraceae | *Turnera subulata* | 0 | 2 | 0 | 0 | 5 | 0 | 7 | 8 | 0 |
| Malvaceae | *Urena lobata* | 0 | 2 | 0 | 0 | 0 | 0 | 0 | 8 | 0 |
| Urticaceae | *Urera baccifera* | 0 | 0 | 0 | 4 | 5 | 0 | 0 | 0 | 0 |
| Urticaceae | *Urera nitida* | 0 | 2 | 0 | 0 | 0 | 0 | 0 | 0 | 0 |
| Sapindaceae | *Urvillea ulmacea* | 0 | 0 | 0 | 0 | 5 | 0 | 0 | 0 | 0 |
| Fabaceae | *Vachellia farnesiana* | 0 | 0 | 0 | 0 | 5 | 6 | 7 | 0 | 0 |
| Humiriaceae | *Vantanea compacta* | 1 | 2 | 0 | 0 | 0 | 0 | 0 | 0 | 0 |
| Humiriaceae | *Vantanea obovata* | 1 | 2 | 0 | 0 | 0 | 0 | 0 | 0 | 0 |
| Boraginaceae | *Varronia curassavica* | 0 | 2 | 0 | 4 | 5 | 0 | 0 | 0 | 0 |
| Boraginaceae | *Varronia dardani* | 0 | 0 | 0 | 0 | 0 | 6 | 0 | 0 | 0 |
| Boraginaceae | *Varronia globosa* | 0 | 0 | 3 | 4 | 5 | 6 | 7 | 8 | 0 |
| Boraginaceae | *Varronia leucocephala* | 0 | 2 | 0 | 0 | 5 | 6 | 7 | 0 | 0 |
| Boraginaceae | *Varronia leucomalloides* | 0 | 0 | 0 | 0 | 5 | 0 | 0 | 8 | 9 |
| Boraginaceae | *Varronia polycephala* | 0 | 0 | 0 | 4 | 0 | 6 | 0 | 0 | 0 |
| Caricaceae | *Vasconcellea quercifolia* | 0 | 0 | 0 | 0 | 5 | 0 | 0 | 0 | 0 |
| Fabaceae | *Vatairea macrocarpa* | 0 | 2 | 3 | 0 | 5 | 0 | 0 | 8 | 9 |
| Velloziaceae | *Vellozia canelinha* | 1 | 0 | 0 | 0 | 0 | 0 | 0 | 0 | 0 |
| Velloziaceae | *Vellozia dasypus* | 1 | 0 | 0 | 0 | 0 | 0 | 0 | 0 | 0 |
| Velloziaceae | *Vellozia hemisphaerica* | 1 | 0 | 0 | 0 | 0 | 0 | 0 | 0 | 0 |
| Velloziaceae | *Vellozia punctulata* | 1 | 0 | 0 | 0 | 0 | 0 | 0 | 0 | 0 |
| Velloziaceae | *Vellozia seubertiana* | 1 | 0 | 0 | 0 | 0 | 0 | 0 | 0 | 0 |
| Velloziaceae | *Vellozia sincorana* | 1 | 0 | 0 | 0 | 0 | 0 | 0 | 0 | 0 |
| Asteraceae | *Verbesina diversifolia* | 0 | 2 | 3 | 4 | 0 | 0 | 0 | 0 | 0 |
| Asteraceae | *Verbesina glabrata* | 1 | 0 | 0 | 0 | 0 | 0 | 0 | 0 | 0 |
| Asteraceae | *Verbesina luetzelburgii* | 1 | 0 | 0 | 0 | 0 | 0 | 0 | 0 | 0 |
| Asteraceae | *Verbesina macrophylla* | 1 | 0 | 3 | 4 | 5 | 0 | 0 | 0 | 0 |
| Asteraceae | *Vernonanthura brasiliana* | 0 | 0 | 3 | 4 | 0 | 0 | 7 | 0 | 9 |
| Asteraceae | *Vernonanthura fagifolia* | 1 | 0 | 0 | 0 | 0 | 0 | 0 | 0 | 0 |
| Asteraceae | *Vernonanthura ferruginea* | 1 | 0 | 0 | 0 | 0 | 0 | 0 | 0 | 9 |
| Asteraceae | *Vernonanthura phosphorica* | 0 | 0 | 3 | 4 | 0 | 0 | 0 | 0 | 0 |
| Asteraceae | *Vernonanthura polyanthes* | 1 | 0 | 0 | 0 | 0 | 0 | 0 | 0 | 0 |
| Asteraceae | *Vernonanthura subverticillata* | 0 | 0 | 3 | 0 | 0 | 0 | 0 | 0 | 0 |
| Asteraceae | *Vernonia arborescens* | 0 | 0 | 0 | 0 | 0 | 0 | 7 | 0 | 0 |
| Asteraceae | *Vernonia scabra* | 0 | 0 | 3 | 4 | 0 | 0 | 0 | 0 | 0 |
| Malpighiaceae | *Verrucularia glaucophylla* | 1 | 0 | 0 | 0 | 0 | 0 | 0 | 0 | 0 |
| Myristicaceae | *Virola gardneri* | 0 | 0 | 3 | 4 | 0 | 0 | 0 | 0 | 0 |
| Myristicaceae | *Virola surinamensis* | 0 | 0 | 0 | 0 | 0 | 0 | 0 | 0 | 9 |
| Hypericaceae | *Vismia guianensis* | 1 | 2 | 3 | 4 | 5 | 0 | 0 | 8 | 9 |
| Hypericaceae | *Vismia micrantha* | 0 | 0 | 0 | 0 | 0 | 0 | 0 | 0 | 9 |
| Verbenaceae | *Vitex calothyrsa* | 0 | 0 | 0 | 0 | 0 | 0 | 0 | 0 | 9 |
| Verbenaceae | *Vitex cuspidata* | 0 | 0 | 0 | 0 | 0 | 0 | 7 | 0 | 0 |
| Verbenaceae | *Vitex cymosa* | 0 | 2 | 3 | 0 | 0 | 6 | 0 | 0 | 9 |
| Verbenaceae | *Vitex gardneriana* | 0 | 0 | 0 | 0 | 0 | 6 | 0 | 0 | 0 |
| Verbenaceae | *Vitex hypoleuca* | 0 | 2 | 0 | 0 | 0 | 0 | 0 | 0 | 0 |
| Verbenaceae | *Vitex mombassae* | 0 | 2 | 0 | 0 | 0 | 0 | 0 | 0 | 0 |
| Verbenaceae | *Vitex panshiniana* | 0 | 2 | 0 | 0 | 0 | 0 | 0 | 0 | 0 |
| Verbenaceae | *Vitex polygama* | 0 | 2 | 0 | 0 | 5 | 6 | 0 | 0 | 0 |
| Verbenaceae | *Vitex rufescens* | 1 | 2 | 3 | 4 | 5 | 0 | 0 | 0 | 0 |
| Verbenaceae | *Vitex schaueriana* | 0 | 2 | 0 | 0 | 0 | 6 | 0 | 0 | 9 |
| Vochysiaceae | *Vochysia acuminata* | 1 | 0 | 0 | 0 | 0 | 0 | 0 | 0 | 0 |
| Vochysiaceae | *Vochysia emarginata* | 0 | 0 | 0 | 0 | 0 | 0 | 0 | 0 | 0 |
| Vochysiaceae | *Vochysia lucida* | 0 | 2 | 0 | 0 | 0 | 0 | 0 | 0 | 0 |
| Vochysiaceae | *Vochysia martiana* | 0 | 0 | 0 | 0 | 0 | 0 | 0 | 0 | 0 |
| Vochysiaceae | *Vochysia oblongifolia* | 0 | 0 | 0 | 4 | 0 | 0 | 0 | 0 | 0 |
| Vochysiaceae | *Vochysia obovata* | 1 | 0 | 0 | 0 | 0 | 0 | 0 | 0 | 0 |
| Vochysiaceae | *Vochysia oppugnata* | 1 | 0 | 0 | 0 | 0 | 0 | 0 | 0 | 0 |
| Vochysiaceae | *Vochysia pyramidalis* | 1 | 2 | 0 | 0 | 0 | 0 | 0 | 8 | 0 |
| Vochysiaceae | *Vochysia rufa* | 0 | 0 | 3 | 0 | 0 | 0 | 0 | 0 | 0 |
| Vochysiaceae | *Vochysia thyrsoidea* | 1 | 0 | 0 | 0 | 0 | 0 | 0 | 0 | 0 |
| Vochysiaceae | *Vochysia tucanorum* | 1 | 0 | 3 | 0 | 0 | 0 | 0 | 0 | 0 |
| Malvaceae | *Waltheria albicans* | 0 | 0 | 0 | 0 | 0 | 6 | 0 | 0 | 0 |
| Malvaceae | *Waltheria brachypetala* | 0 | 0 | 0 | 0 | 0 | 0 | 7 | 8 | 0 |
| Malvaceae | *Waltheria cinerescens* | 1 | 2 | 0 | 0 | 0 | 0 | 0 | 0 | 0 |
| Malvaceae | *Waltheria communis* | 0 | 0 | 0 | 0 | 5 | 0 | 0 | 8 | 0 |
| Malvaceae | *Waltheria ferruginea* | 0 | 0 | 0 | 0 | 5 | 6 | 7 | 8 | 9 |
| Malvaceae | *Waltheria indica* | 1 | 2 | 0 | 4 | 5 | 6 | 7 | 8 | 9 |
| Malvaceae | *Waltheria macropoda* | 0 | 0 | 0 | 0 | 0 | 6 | 7 | 0 | 0 |
| Malvaceae | *Waltheria operculata* | 0 | 0 | 0 | 0 | 0 | 0 | 7 | 0 | 0 |
| Malvaceae | *Waltheria paniculata* | 0 | 0 | 0 | 0 | 0 | 6 | 0 | 0 | 0 |
| Malvaceae | *Waltheria viscosissima* | 0 | 2 | 0 | 0 | 0 | 0 | 0 | 8 | 0 |
| Rubiaceae | *Warszewiczia schwackei* | 0 | 0 | 0 | 0 | 0 | 0 | 0 | 8 | 0 |
| Asteraceae | *Wedelia alagoensis* | 0 | 0 | 0 | 4 | 5 | 0 | 0 | 0 | 0 |
| Asteraceae | *Wedelia fruticosa* | 0 | 2 | 0 | 0 | 0 | 0 | 0 | 8 | 9 |
| Asteraceae | *Wedelia goyazensis* | 0 | 0 | 0 | 0 | 0 | 0 | 7 | 0 | 0 |
| Asteraceae | *Wedelia hispidula* | 0 | 0 | 0 | 0 | 5 | 0 | 0 | 0 | 0 |
| Asteraceae | *Wedelia hookeriana* | 0 | 0 | 0 | 0 | 5 | 0 | 7 | 0 | 0 |
| Asteraceae | *Wedelia villosa* | 0 | 2 | 0 | 0 | 5 | 6 | 7 | 8 | 9 |
| Cunoniaceae | *Weinmannia paulliniifolia* | 1 | 0 | 0 | 0 | 0 | 0 | 0 | 0 | 0 |
| Malvaceae | *Wissadula amplissima* | 0 | 2 | 0 | 0 | 0 | 0 | 7 | 0 | 0 |
| Malvaceae | *Wissadula caribaea* | 0 | 0 | 0 | 0 | 0 | 0 | 0 | 0 | 9 |
| Malvaceae | *Wissadula contracta* | 0 | 2 | 0 | 4 | 5 | 0 | 0 | 0 | 0 |
| Malvaceae | *Wissadula periplocifolia* | 0 | 0 | 0 | 0 | 0 | 6 | 0 | 0 | 9 |
| Asteraceae | *Wunderlichia cruelsiana* | 1 | 0 | 0 | 0 | 0 | 0 | 0 | 0 | 0 |
| Ximeniaceae | *Ximenia americana* | 0 | 2 | 3 | 4 | 5 | 6 | 7 | 8 | 9 |
| Ximeniaceae | *Ximenia coriacea* | 1 | 0 | 0 | 0 | 0 | 6 | 0 | 0 | 0 |
| Ximeniaceae | *Ximenia intermedia* | 0 | 0 | 0 | 0 | 5 | 0 | 0 | 0 | 0 |
| Bignoniaceae | *Xylophragma harleyi* | 0 | 0 | 0 | 0 | 0 | 0 | 0 | 0 | 0 |
| Annonaceae | *Xylopia aromatica* | 0 | 0 | 3 | 0 | 0 | 6 | 0 | 0 | 0 |
| Annonaceae | *Xylopia frutescens* | 0 | 2 | 3 | 0 | 5 | 0 | 0 | 0 | 0 |
| Annonaceae | *Xylopia laevigata* | 1 | 2 | 3 | 0 | 5 | 0 | 0 | 8 | 0 |
| Annonaceae | *Xylopia sericea* | 0 | 2 | 3 | 0 | 0 | 0 | 0 | 8 | 9 |
| Salicaceae | *Xylosma ciliatifolia* | 0 | 0 | 3 | 4 | 0 | 0 | 7 | 8 | 9 |
| Rutaceae | *Zanthoxylum acuminatum* | 0 | 0 | 0 | 4 | 0 | 0 | 0 | 0 | 0 |
| Rutaceae | *Zanthoxylum caribaeum* | 0 | 0 | 0 | 0 | 5 | 0 | 0 | 0 | 0 |
| Rutaceae | *Zanthoxylum fagara* | 0 | 0 | 0 | 4 | 0 | 6 | 0 | 0 | 0 |
| Rutaceae | *Zanthoxylum gardneri* | 0 | 2 | 0 | 0 | 5 | 0 | 0 | 8 | 0 |
| Rutaceae | *Zanthoxylum hamadryadicum* | 0 | 0 | 0 | 0 | 0 | 0 | 7 | 8 | 9 |
| Rutaceae | *Zanthoxylum monogynum* | 0 | 0 | 3 | 0 | 0 | 0 | 0 | 0 | 0 |
| Rutaceae | *Zanthoxylum petiolare* | 0 | 0 | 0 | 4 | 5 | 0 | 0 | 0 | 0 |
| Rutaceae | *Zanthoxylum rhoifolium* | 1 | 2 | 3 | 4 | 5 | 6 | 7 | 8 | 0 |
| Rutaceae | *Zanthoxylum riedelianum* | 0 | 0 | 3 | 0 | 0 | 6 | 0 | 0 | 0 |
| Rutaceae | *Zanthoxylum stelligerum* | 1 | 0 | 0 | 4 | 5 | 0 | 7 | 8 | 9 |
| Rutaceae | *Zanthoxylum syncarpum* | 0 | 0 | 0 | 0 | 0 | 0 | 0 | 8 | 9 |
| Rutaceae | *Zanthoxylum tingoassuiba* | 0 | 0 | 3 | 0 | 0 | 0 | 0 | 0 | 0 |
| Fabaceae | *Zapoteca portoricensis* | 0 | 0 | 0 | 4 | 0 | 0 | 0 | 0 | 0 |
| Bignoniaceae | *Zeyheria montana* | 1 | 0 | 0 | 0 | 0 | 0 | 0 | 0 | 0 |
| Bignoniaceae | *Zeyheria tuberculosa* | 1 | 0 | 3 | 0 | 5 | 6 | 7 | 8 | 0 |
| Rhamnaceae | *Ziziphus cotinifolia* | 1 | 0 | 3 | 0 | 0 | 6 | 7 | 0 | 0 |
| Rhamnaceae | *Ziziphus joazeiro* | 0 | 0 | 3 | 4 | 5 | 6 | 7 | 8 | 0 |
| Rhamnaceae | *Ziziphus platyphylla* | 0 | 0 | 0 | 0 | 5 | 6 | 0 | 8 | 9 |
| Rhamnaceae | *Ziziphus undulata* | 0 | 0 | 3 | 0 | 5 | 0 | 0 | 0 | 0 |
| Fabaceae | *Zollernia ilicifolia* | 0 | 0 | 3 | 4 | 5 | 0 | 0 | 0 | 0 |
| Fabaceae | *Zollernia paraensis* | 0 | 0 | 0 | 0 | 0 | 6 | 0 | 0 | 0 |
| Fabaceae | *Zornia brasiliensis* | 0 | 2 | 0 | 0 | 0 | 0 | 7 | 0 | 0 |
| Fabaceae | *Zornia diphylla* | 1 | 0 | 0 | 0 | 5 | 0 | 0 | 0 | 0 |
| Fabaceae | *Zornia echinocarpa* | 0 | 0 | 0 | 0 | 5 | 0 | 0 | 0 | 0 |
| Fabaceae | *Zornia flemmingioides* | 1 | 0 | 0 | 0 | 0 | 0 | 0 | 0 | 0 |
| Fabaceae | *Zornia latifolia* | 0 | 0 | 0 | 0 | 0 | 0 | 0 | 8 | 0 |
| Fabaceae | *Zornia myriadena* | 0 | 0 | 0 | 0 | 5 | 0 | 0 | 0 | 0 |
| Fabaceae | *Zornia orbiculata* | 0 | 0 | 0 | 0 | 0 | 0 | 7 | 0 | 0 |
| Fabaceae | *Zornia sericea* | 0 | 0 | 0 | 0 | 0 | 0 | 7 | 0 | 0 |
| Fabaceae | *Zygia latifolia* | 0 | 0 | 3 | 0 | 0 | 0 | 0 | 0 | 0 |
